# Strongly Polarized π-Extended 1,4-Dihydropyrrolo[3,2-*b*]pyrroles Fused with Tetrazolo[1,5-*a*]quinolines

**DOI:** 10.1021/acs.joc.3c02916

**Published:** 2024-03-26

**Authors:** Mohammad B. Teimouri, Irena Deperasińska, Matt Rammo, Marzena Banasiewicz, Charles W. Stark, Łukasz Dobrzycki, Michał K. Cyrański, Aleksander Rebane, Daniel T. Gryko

**Affiliations:** † Institute of Organic Chemistry, Polish Academy of Sciences, Kasprzaka 44/52, Warsaw 01-224, Poland; ‡ Faculty of Chemistry, Kharazmi University, Mofateh Ave, Tehran 15719-14911, Iran; § Institute of Physics of Polish Academy of Sciences, Polish Academy of Sciences, Al. Lotników 32/46, Warsaw 02-668, Poland; ∥ National Institute for Chemical Physics and Biophysics, Akadeemia tee 23, Tallinn 12618, Estonia; ⊥ Faculty of Chemistry, University of Warsaw, Pasteura 1, Warsaw 02-093, Poland; # Department of Physics, Montana State University, Bozeman, Montana 59717, United States

## Abstract

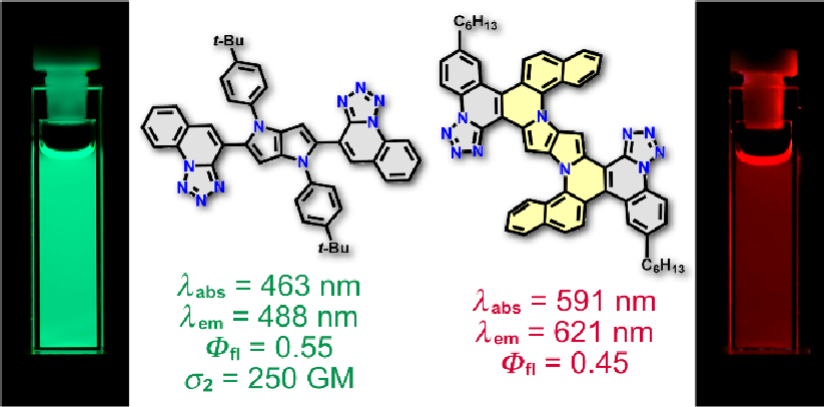

A straightforward route to 1,4-dihydropyrrolo[3,2-*b*]pyrroles comprised of two electron-withdrawing quinoline or tetrazolo[1,5-*a*]quinoline scaffolds has been developed. The versatile multicomponent reaction affording 1,4-dihydropyrrolo[3,2-*b*]pyrroles combined with intramolecular direct arylation enables assembly of these products in just three steps from anilines with overall yields exceeding 30%. The planarized, ladder-type heteroacenes possess up to 14 conjugated rings. These nominally quadrupolar materials exhibit efficient fluorescence with wavelengths spanning most of the visible spectrum from green–yellow for the dyes possessing biaryl bridges and orange–red for the fully fused systems. In many cases, the fluorescence quantum yields are large, the solvatofluorochromic effects are strong, and the fluorescence is maintained even in crystalline state. Analysis of the electronic structure of these molecular architectures using quantum chemical methods suggests that the character and position of the flanking heterocycle determine the shape of HOMO and LUMO and their extension to *N*-aryl substituents, influencing the values of molar absorption coefficient. An experimental study of the two-photon absorption (2PA) properties has revealed that it occurs in the 700–800 nm range with apparent deviation from the Laporte parity selection rule, which may be attributed to Hertzberg–Teller contribution to vibronically allowed 2PA transition.

## Introduction

Modern chemistry and materials science of complex conjugated functional dyes often implies controlled modulation of the optoelectronic properties achieved by fine-tuning the π-system. Extending the π-conjugation by linking two or more unsaturated hydrocarbons at the periphery of a conjugated architecture is a powerful method for targeted modification of the available π-cloud, leading to changes in the local or global delocalization character.^1^ The π-electron distribution within a molecule can also be manipulated using both simple substituted aryl groups and exotic heteroaromatic motifs.^2^

The 1,4-dihydropyrrolo[3,2-*b*]pyrrole (DHPP) motif, comprised of two fused pyrrole units in a centrosymmetric orientation, is not only the strongest known electron donor among 10π-electron heteropentalenes but can also be obtained through an easily accessible one-pot synthesis.^3^ The available facile synthesis of tetraaryl-1,4-dihydropyrrolo[3,2-*b*]pyrroles (TAPPs), combined with promising photophysical properties, is making TAPP scaffold-based organic chromophores attractive for many research applications.^4^

Over the past decade, the versatility of 1,4-dihydropyrrolo[3,2-*b*]pyrroles has been demonstrated in an expansive range of areas of research including two-photon absorption (2PA),^5^ ground- and excited-state symmetry breaking,^6^ solvatofluorochromism,^7^ tunable single-molecule conductance,^8^ targeting the mitochondrial TET1 protein,^9^ direct solvent probing via H-bonding interactions,^10^ photochromic analysis of halocarbons,^11^ high-performance organic field-effect transistors,^12^ dye-sensitized solar cells,^13^ and many others.^14−22^

The exceptional feature of DHPPs is that a convenient one-pot synthesis enables the assembly of the heterocyclic scaffold possessing four arenes linked via biaryl linkages.^23,24^ The variety of these arenes is quite vast especially considering that by adding just one more synthetic step, e.g., direct arylation, the otherwise weakly coupled dye can be efficiently transformed into a fused planar compound possessing 8, 10, or 12 conjugated aromatic rings.^25^ These advantages provide organic chemists a unique and versatile toolbox that currently has few if any analogs, at least in the area of centrosymmetric quadrupolar dyes.

Tetrazole is a five-membered, fully conjugated nitrogen-rich 6π-azaheterocycle consisting of a single carbon and four consecutive nitrogen atoms,^26^ which render the system very electron deficient. Interestingly, tetrazole has the highest nitrogen content among the stable five-membered heterocycles. Despite the high nitrogen content, tetrazole and most of its derivatives remain relatively stable under the influence of heat or microwave radiation.^27^ Furthermore, the related compounds are able to withstand a wide range of chemical environments such as strongly acidic and basic media, alkylating agents, dienophiles, as well as oxidizing and reducing conditions.^27^ Even though tetrazoles^28^ and their annulated derivatives have received considerable attention over the past years in fields of medicine, agriculture, and material science, it is still surprising that applications of tetrazoloquinoline-containing compounds as functional dyes have been rarely investigated to date.^29^

Our objective is to use unique features of TAPPs’ chemistry for the synthesis of large, structurally unique, heavily *N*-doped nanographenes, possessing tetrazole scaffolds. We reasoned that incorporation of moderately electron-withdrawing moieties such as tetrazolo[1,5-*a*]quinoline and quinoline at strongly conjugated positions 2 and 5 can be utilized to endow resulting dyes with desired photophysical properties.

## Results and Discussion

### Design and Synthesis

The synthesis of centrosymmetric quadrupolar TAPPs possessing fused tetrazole moieties starts from the preparation of the corresponding aldehyde. We were attracted by the exceptional simplicity of the Meth-Cohn synthesis of 2-chloro-3-formylquinolines, which enables the assembly of building blocks possessing the exact functionality required (in two steps from commercial arylamines).^30^ The tetrazolo[1,5-*a*]quinolines precursors were obtained from the corresponding 2-chloroquinolines and NaN_3_ via a general method involving the two-step process of S_N_Ar/ring–chain azido-tautomerization (see Supporting Information for details).^31^

**Scheme 1:**
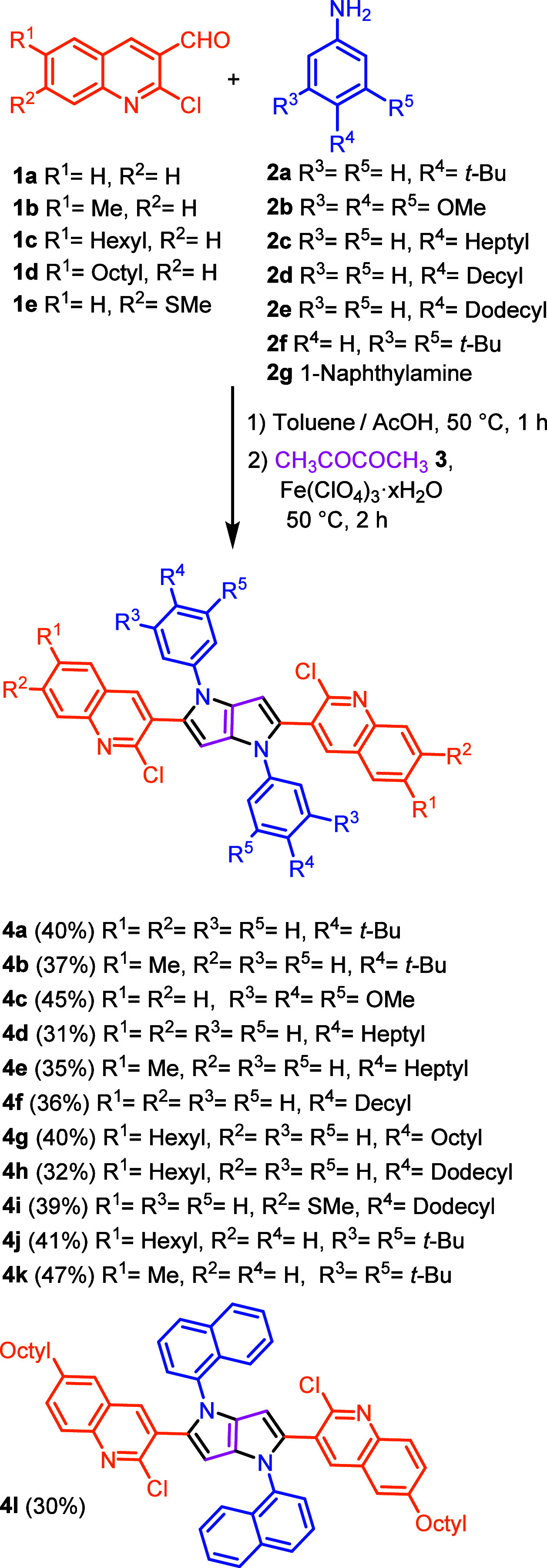
Synthesis of 2,5-Bis(2-chloroquinoline)-1,4-diaryl pyrrolo[3,2-*b*]pyrroles (CQPPs)

**Scheme 2:**
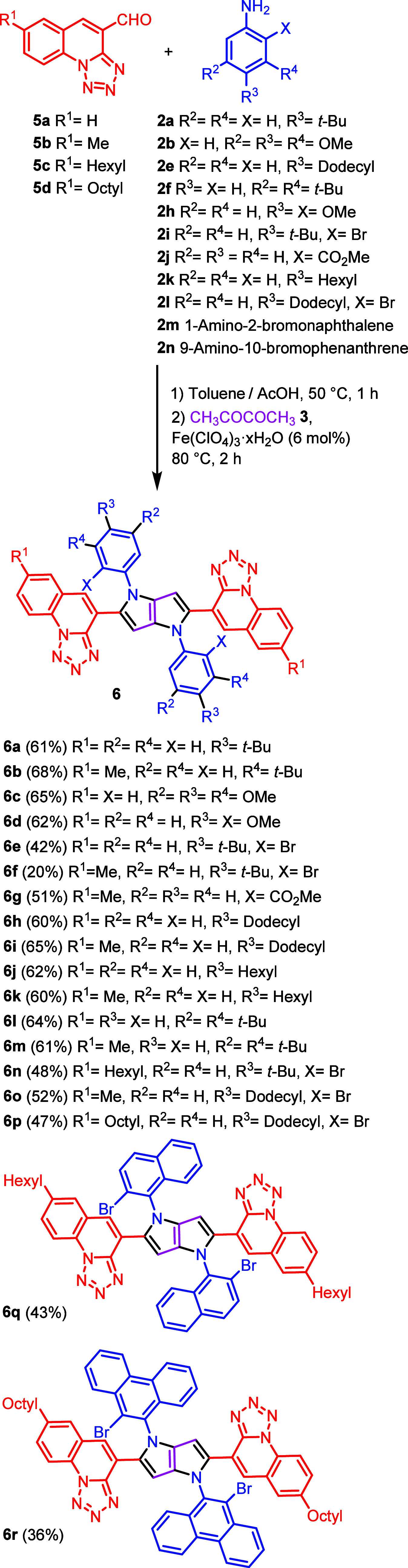
Synthesis of 2,5-Bis(tetrazoloquinoline)-1,4-diaryl pyrrolo[3,2-*b*]pyrroles (TQPPs)

**Figure 1:**
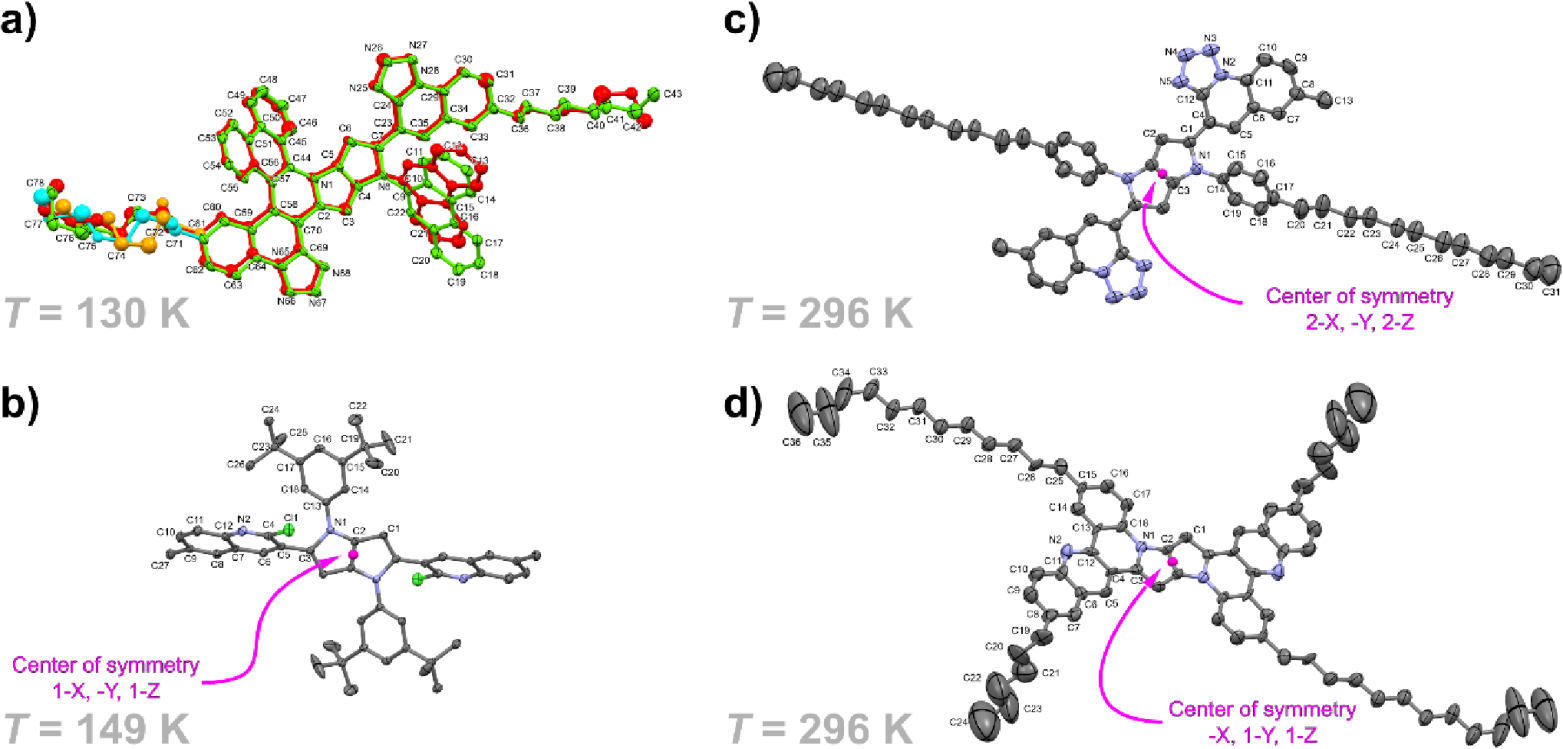
X-ray crystal structures of dyes **11d** (a), **4k** (b), **6j** (c), and **10a** (d). OTRTEP plot of molecules drawn at 50% probability level. Hydrogen molecules omitted for clarity.

Having 2-chloro-3-formylquinolines (**1a**–**e**) and tetrazolo[1,5-*a*]quinoline-4-carbaldehyde (**5a**–**d**) in hand, we subjected them to our multicomponent reaction with primary aromatic amines **2a**–**f** and butane-2,3-dione (**3**), resulting in the formation of the corresponding 2,5-bis(2-chloroquinoline)-1,4-diaryl-pyrrolo[3,2-*b*]pyrroles (CQPPs) **4a**–**4l** and 2,5-bis(tetrazoloquinoline)-1,4-diaryl-pyrrolo[3,2-*b*]pyrroles (TQPPs) (**6a**–**6q**) in moderate to good yields (Schemes 1 and 2). Furthermore, the molecular structures of **4k** and **6j** were confirmed by X-ray crystallography (Figure 1b,c). The DHPP core in **4k** is perfectly planar, and the torsional angles between the peripheral substituted benzene rings attached to nitrogen atoms and DHPP core are 42° (Figure 1b). Chloroquinoline moieties attached to C-2 and C-5 atoms are both twisted at 51°. On the other hand, the dihedral angle between tetrazoloquinolinyl substituent and the DHPP core in **6j** is only 27° (Figure 1c). Noteworthy, this angle is only 35° for unsubstituted benzene in the ground state.^3,4^

**Scheme 3:**
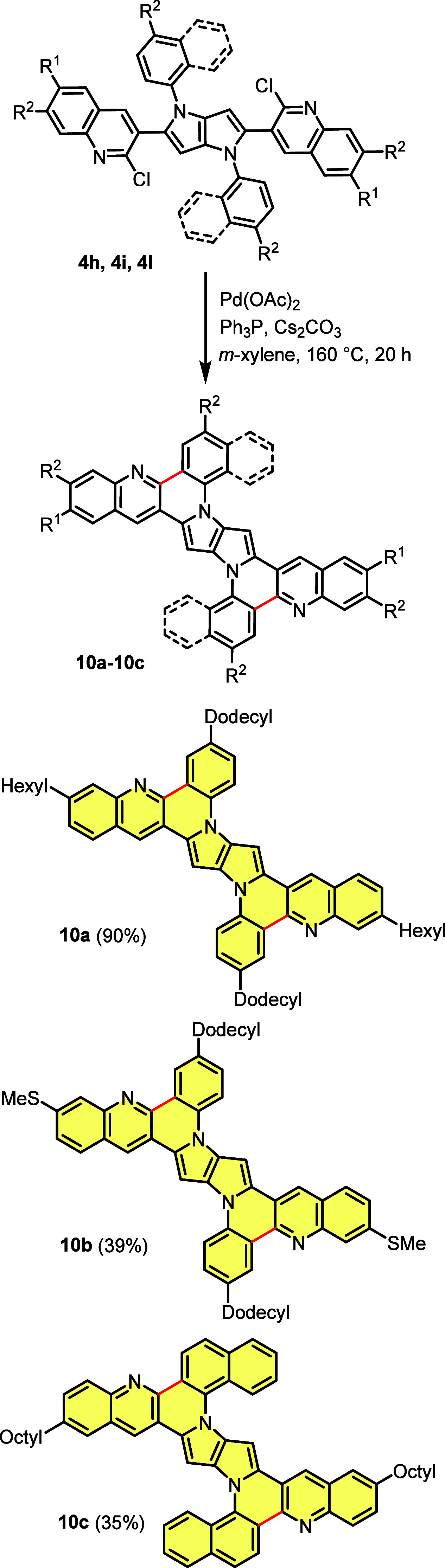
Synthesis of Ladder-Type Quinoline-Fused Pyrrolo[3,2-*b*]pyrroles (QFPPs) via Intramolecular Double Direct Arylation

Having a CQPP family with chlorine atoms and benzene rings strategically placed in respect to the central DHPP core in hand, precursors **4h**, **4i,** and **4l** were transformed in good yields into a ladder-type quinoline-fused pyrrolo[3,2-*b*]pyrroles (QFPPs) **10a**–**10c** utilizing double intramolecular direct arylation (Scheme 3). Unambiguous evidence for the proposed structure of **10** was finally obtained by single crystal X-ray diffraction analysis in the case of **10a** (Figure S3).

**Scheme 4:**
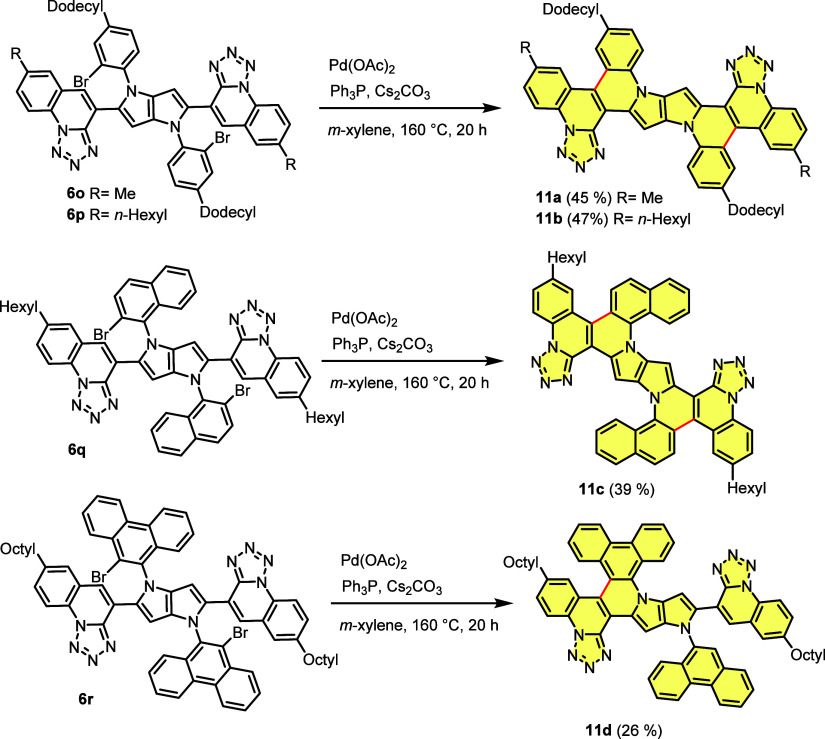
Synthesis of Pyrrolopyrrole-Containing Multiple Azahelicene **11a**–**11d** via Intramolecular Palladium-Catalyzed Direct Arylation

The analogous concept has been employed to obtain centrosymmetric, planarized tetrazoloquinoline-fused pyrrolo[3,2-*b*]pyrroles (TQFPPs). The pivotal difference in these two approaches is that in the case of quinoline-fused dyes, halogen atoms were placed on substituents at positions 2 and 5, whereas for tetrazoloquinoline-fused TAPPs, *N*-aryl substituents possessed bromine atoms. Dyes **6o** and **6p** were transformed into TQFPPs **11a** and **11b** via double intramolecular palladium-catalyzed direct arylation. High yields achieved for all these transformations encouraged us to attempt the synthesis of new TQFPPs incorporating [*n*]helicenes with hope to obtain the molecules with interesting photophysical properties. To achieve this goal, the chosen building blocks were 1-amino-2-bromonaphthalene and 9-amino-10-bromophenanthrene as the arylamine components and tetrazolo[1,5-*a*]quinoline-4-carbaldehyde bearing hexyl or octyl substituents as the aldehyde. Consequently, the brominated TQPPs **6q** and **6r** were prepared using appropriate starting materials using our optimized three-component condensation (Scheme 4).

The 2-fold cyclization of TQPP **6q** was carried out to give the symmetric helicene **11c**, including four [4]azasubhelicenes and two [5]azasubhelicenes in 39% yield. When TQPP **6r** was subjected to selective intramolecular direct arylation, to our surprise, the desired cyclization took place only on one side of the substrate, whereas debromination via a protodepalladation mechanism occurred on the other side, giving an unsymmetrical pyrrolopyrrole-containing triple azahelicene **11d** (Figure 1a) isolated in 26% yield (Scheme 4).

### Photophysical Results

**Figure 2:**
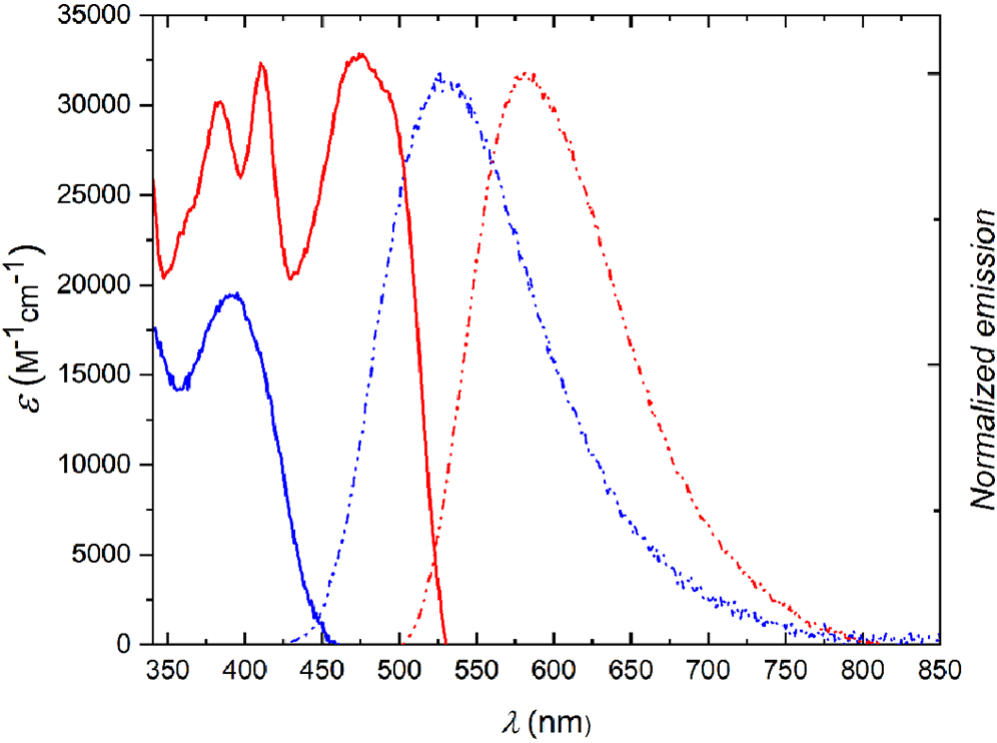
Molar extinction spectrum (solid line) and normalized fluorescence emission spectrum (dashed-dotted line) of compounds **4h** (blue) and **10a** (red) in DCM.

**Figure 3:**
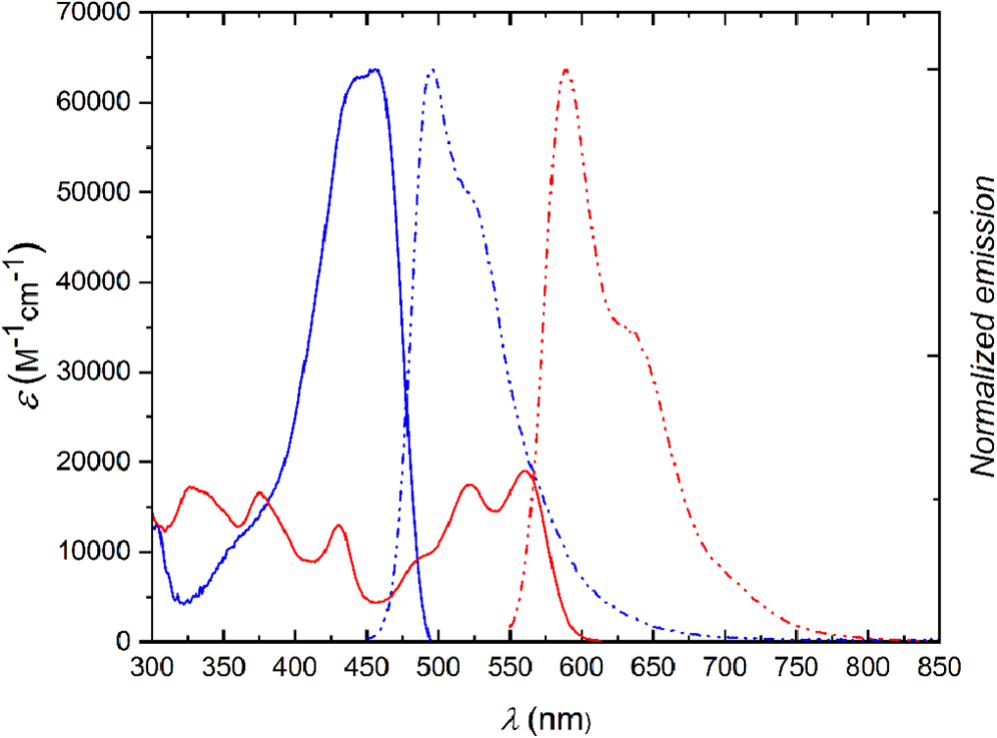
Molar extinction spectrum (solid line) and normalized fluorescence emission spectrum (dashed-dotted line) of compounds **6i** (blue) and **11a** (red) in DCM.

Spectroscopic properties of all prepared dyes were measured in toluene (if solubility allowed) (dielectric constant ε = 2.38) and dichloromethane (DCM, ε = 8.93). Spectroscopic properties of representative dyes **4a**, **4g**, **6a**, **6g**, **6r**, **10a**, **10c**, **11a**, **11c,** and **11d** are shown in Figures 2 and 3 and summarized in Table 1 (see Table S1 for comprehensive results). The absorption spectra of CQPPs (dyes **4a**–**4k**) are dominated by a strong band at around 390 nm, with the peak extinction coefficient values in the range ε = 15000–30000 M^–1^·cm^–1^. Comparison of the peak absorption wavelength of CQPPs with that of structurally related 2,5-bis(quinolin-2-yl)pyrrolo[3,2-*b*]pyrroles^25b^ shows that in toluene the former dyes absorb wavelengths ca. 30–50 nm lower. This is attributed to the presence of chloro-substituent located *ortho*- to DHPP core, which, for steric reasons (confirmed by X-ray, Figure 1b), effectively disturbs ground state electronic coupling between the core and the periphery of the molecule. On the other hand, the absorption maxima of CQPPs are bathochromically shifted by ca. 30 nm compared to those of 2,5-bis(naphth-2-yl)pyrrolo[3,2-*b*]pyrroles,^24b^ suggesting that the electron-withdrawing character of quinoline substituents is, nevertheless, clearly manifested. This behavior is even more pronounced for tetrazole-bearing TQPPs, which typically exhibit absorption maxima at 455–464 nm (dyes **6a**–**6q**), with a vibronic shoulder at ca. 20 nm lower. The only exception from this pattern is dye **6r**, for which the vibronic band is stronger than the origin band. The redshift observed for TQPPs was expected due to the significant increase in acceptor character of the substituents at positions 2 and 5 from weakly to strongly electron withdrawing. Additionally, the lack of an *ortho*-chloro substituent allows better planarization/electronic coupling in the ground state, which is confirmed by X-ray studies (Figure 1a). TQPPs also exhibit much higher extinction coefficients and fluorescence quantum yields (Φ_fl_) than CQPPs, despite the presence of heavy atoms and nitrogen-rich aromatic rings, which often lead to very efficient intersystem crossing and quenching of fluorescence.^32^

**Table 1:** Spectroscopic Properties of Compounds **4a**, **4g**, **6a**, **6g**, **6r**, **10a**, **10c**, **11a**, **11c,** and **11d** Obtained in Toluene and DCM

	in DCM					in toluene				
entry	λ_max (Ab)_ (nm)	ε@λ_max_ (M^–1^ cm^–1^)	λ_max (Em)_ (nm)	Stokes shift (cm^–1^)	Φ_fl_	λ_max (Ab)_ (nm)	ε@λ_max_ (M^–1^cm^–1^)	λ_max (Em)_ (nm)	Stokes shift (cm^–1^)	Φ_fl_
**4a**	397	14700	540	6700	0.13	399	15100	480	4200	0.19
**4g**	390	20800	533	6900	0.15	398	19800	472	3900	0.19
**6a**	456	55400	495	1700	0.56	463	52100	488	1100	0.55
**6g**	458	58100	501	1900	0.55	459	-a	494	1500	0.51
**6r**	434	32500	486	2500	0.13	460	34600	480	900	0.16
**10a**	474	32800	581	3900	0.64	501	36900	532	1200	0.52
**10c**	483	31600	589	3700	0.26	485	33600	554	2600	0.49
**11a**	561	19100	590	900	1.0	563	40600	586	700	0.79
**11c**	590	13000	622	900	0.27	591	38100	621	800	0.45
**11d**	505	16600	611	3400	0.21	509	19700	591	2700	0.16

a Solubility was too low to reliably measure ε.

Needless to say, rigid and planar QFPPs and **11a**–**c** display remarkably different characteristics compared to parent TAPP molecules. A significant ≈100 nm bathochromic shift of absorption were observed for dye **10a** (when compared to **4h**), as well as for dye **11a** (when compared to **6i**) (Figures 2 and 3). Further consequences of planarization were larger Φ_fl_ (reaching 100% in DCM in the case of dye **11a**) and smaller Stokes shifts observed for fused dyes, which are results of more restricted molecular motion comparing to the parent dyes. As expected, the properties of monocoupling product **11d** stand in the middle between those of substrates and the bis-coupled products.

As far as the solid-state emission is concerned, both groups of examined quadrupolar dyes, **4** and **6**, exhibit strikingly different properties. The analysis of solid-state spectra reveals that in the case of **4d**, **4h,** and **4i**, absorption is only slightly bathochromically shifted, i.e., to 425 nm, whereas emission is hypsochromically shifted (450–475 nm) compared to data collected in toluene. The only exception from this trend is found in the case of **4k** ( = 515 nm), and it is presumably related to the presence of sterically encumbered substituents (four *tert*-butyl groups), which impact packing. On the other hand, dyes **6a**, **6e**, **6i,** and **6o** do have bathochromically shifted both absorption and emission to 525 and 575 nm, respectively (see Supporting Information for details). For all these dyes, the fluorescence intensity is moderate ranging from 2.9% to 20%.

### Two-Photon Absorption

**Figure 4:**
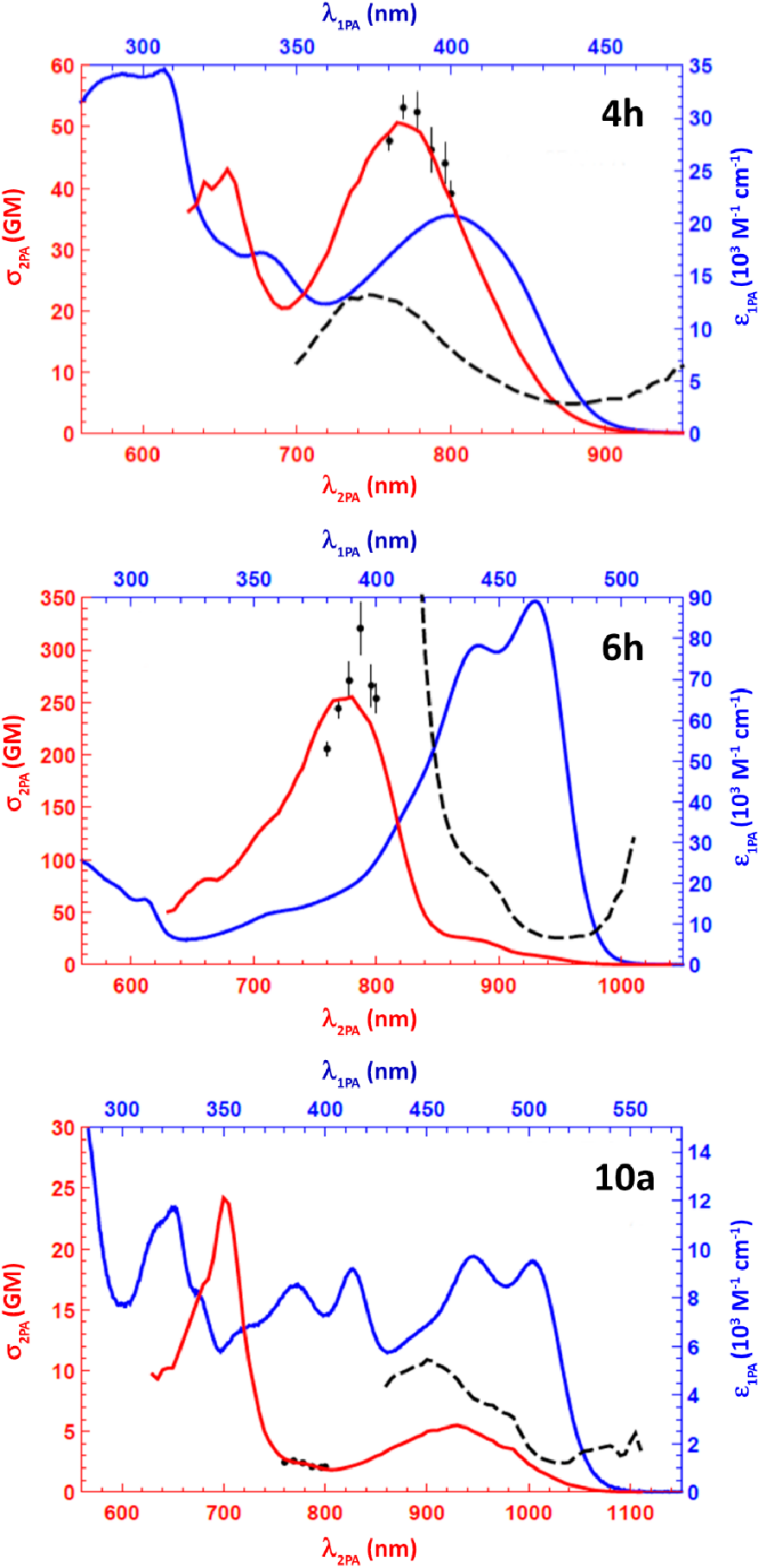
2PA cross-section spectra of **4h**, **6h,** and **10a** in toluene solution in GM (red line, left vertical and lower horizontal scale). Black symbols—absolute 2PA cross sections measured at select wavelengths. Blue line—molar extinction spectra in toluene (blue line, upper horizontal axis). Dashed black line—ratio between the 2PA and 1PA spectral profiles (in arbitrary units).

Figure 4 presents the two-photon absorption (2PA) cross-section spectra in GM units (1 GM = 10^–50^ cm^4^ s^–1^ photon) of representative dyes **4h**, **6h,** and **10a** in toluene measured with 120 fs laser pulses tuned in the wavelength range λ_2PA_= 630–1040 nm (red line, lower horizontal scale) and through the fluorescence excitation method, which consists of scaling the reference-corrected 2PA spectral profiles according to absolute 2PA cross-section values determined at select wavelengths (black symbols). The linear absorption spectra of **4h**, **6h,** and **10a** in toluene are shown for comparison on a 2× expanded wavelength scale (blue line, upper horizontal axis).

In all three dyes, the 2PA shows distinct features with the maximum cross-section values, σ_2PA_ = 50 GM (770 nm) and 43 GM (660 nm) in **4h**, σ_2PA_ = 250 GM (775 nm) in **6h**, and σ_2PA_ = 5 GM (930 nm) and 25 GM (700 nm) in **10a**. The experimental observation that the TQPP **6h** shows almost 1 order of magnitude larger peak σ_2PA_ value compared to the CQPP and QFPP-type compounds correlates well with a stronger-acting electron-withdrawing ability of the attached tetrazole moiety. As a further testimony to the quadrupolar nature of these chromophores, none of the 2PA spectral profiles coincide with underlying 1PA transition profiles. Nevertheless, the ratio of σ_2PA_ vs ε_1PA_ (dashed black lines) remains finite even for the longest wavelength part of the spectra. Such apparent deviation from the Laporte parity selection rule has been previously observed for a number of nominally inversion-symmetric diketopyrrolopyrroles^33^ and is most likely due to Hertzberg–Teller contribution to vibronically allowed 2PA transition.^22^ It is worth to point out that much larger values of σ_2PA_ were reached for various strongly pyrrole derivatives bearing strongly polarized donor–acceptor structure.^34^

### Computational Studies

DFT and TDDFT/M06/6-31G(d,p) calculations with the optimization of molecular structures in the ground electronic state S_0_ and the lowest electronic excited state S_1_ were performed for the description of the spectroscopic properties of molecules **4**, **6**, **10**, and **11** (for results, see Table 2). These dyes have been chosen since they constitute model dyes for the four groups of compounds studied in this project. The calculations also included the molecule **10-x**–regioisomer **10**, which is topologically similar to **11**. The M06 calculation method was chosen as it best reproduces the experimental results (Table S7 and Figure S7). Calculations were performed using the Gaussian 16 package.^35^ The effect of solvent was described in the PCM procedure. The SOC elements were also calculated using the Orca program.^36^

**Table 2:** TDDFT/M06/6-31G(d,p) Calculated Energies and Oscillator Strengths of the Absorption S_0_ → S_i_ and Fluorescence S_1_ → S_0_ Transitions

		absorption		fluorescence	
comp.	solvent	(nm)	*f*	(nm)	*f*
**4**	toluene	422	0.742	483	1.024
	DCM	424	0.815	492	1.289
**6**	toluene	471	1.706	531	1.930
	DCM	478	1.798	551	2.060
**10**	toluene	479	0.965	546	1.045
	DCM	489	1.203	563	1.286
**11**	toluene	550	0.849	642	0.876
	DCM	560	1.021	661	1.041
**(10-x)**	toluene	502	0.593	588	0.627
	DCM	510	0.748	603	0.783

Table 2 contains the calculated energies and oscillator strengths for the absorption (S_0_ → S_1_) and fluorescence (S_1_ → S_0_), characterizing structures optimized in the S_0_ and S_1_ states. Two of the considered molecules, **4** and **6**, are nonplanar systems. The angle between the planes of the peripheral arenes possessing electron-withdrawing character and the DHPP center in **4**, optimized in the S_0_ ground state, is 43°, and is 20° in the case of compound **6**. In the S_1_ excited state, the angle values slightly decrease to 32° and 18°, respectively. The planes between *N*-aryl substituents also form large angles with the DHPP plane: 43° and 45° in the S_0_ and S_1_ states of molecule **4**, and 48° and 49° in the case of **6**, respectively, which is corroborated by X-ray data (Figure 1).

The computational results, given in Table 2, capture the experimental facts, such as the red shifts of spectra of dye **6** relative to TAPP **4** and of dye **11** relative to TAPP **10** (i.e., in the context of the changing the acceptor), as well as **10** relative to **4** and **11** relative to **6** (i.e., in context fused vs nonfused system).

**Figure 5:**
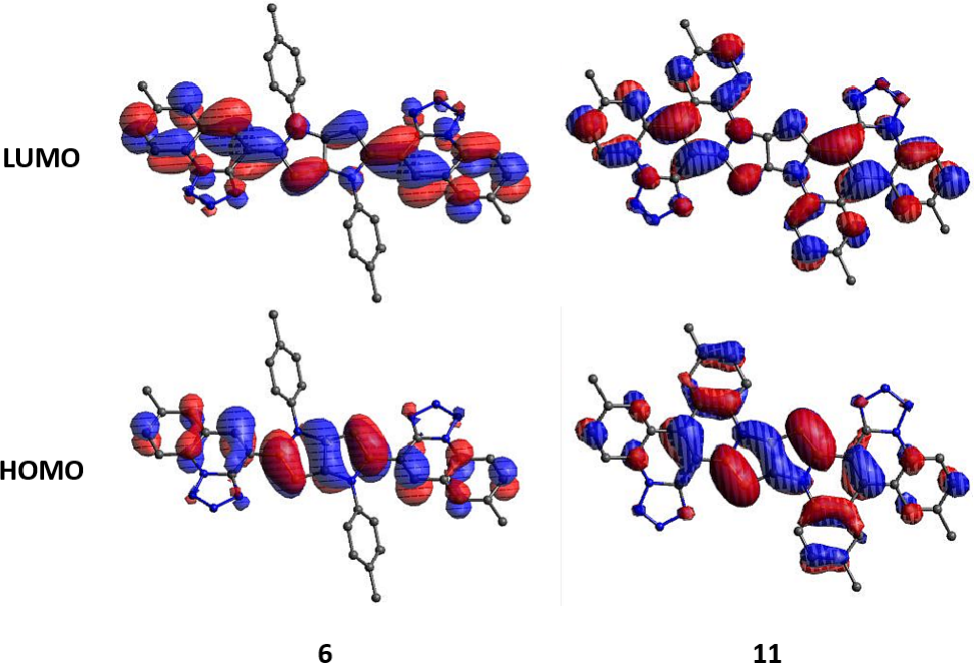
Shape of HOMO and LUMO orbitals of investigated dyes. Similarity and differences between HOMO and LUMO orbitals of nonfused and fused dyes, **6** and **11** – orbitals of **11** are spreading to *N*-aryl substituents.

The calculations reveal that electronic transitions between the S_0_ and S_1_ states in all tested compounds are described by the electronic configuration {HOMO, LUMO}, where HOMO and LUMO have a specific structure, resembling “exciplex” EDA systems. Meaning, the HOMO orbital is created from the HOMO of the DHPP donor with an admixture of HOMO orbitals of both acceptors, and the LUMO orbitals are created from a combination of LUMO orbitals of both acceptors with an admixture of the donor’s LUMO orbital (see Figures 5 and S8; the orbitals of individual elements are shown in Table S8). Interestingly, formation of fully π-conjugated systems **10** and **11** is accompanied by spreading both HOMO and LUMO into *N*-aryl substituents (see Figure 5).

Therefore, the electronic transitions between the S_0_ and S_1_ states in all the tested compounds are qualitatively of the same nature: a multicenter CT transition with a charge shift between the central DHPP scaffold and two symmetrically arranged acceptor moieties at the periphery. Referring to the symbolic record of the CT transition energy *E*_CT_ = *I*_D_ – *E*_A_ + *E*_int_(D,A,R), it implies that the quantitative differences in the spectroscopic properties of these molecules are dictated by the interplay of several factors: differences in the electron affinity of acceptors and differences in interaction energy, resulting from variability in the mutual orientation of the donor and acceptors and the role played by the *N*-aryl substituents. The red shifts of the absorption and fluorescence spectra of **6** relative to **4** (and **11** relative to dye **10**) are in correspondence with the change in the electron affinity of the acceptor (≈1800 cm^–1^, Table S8).

**Figure 6:**
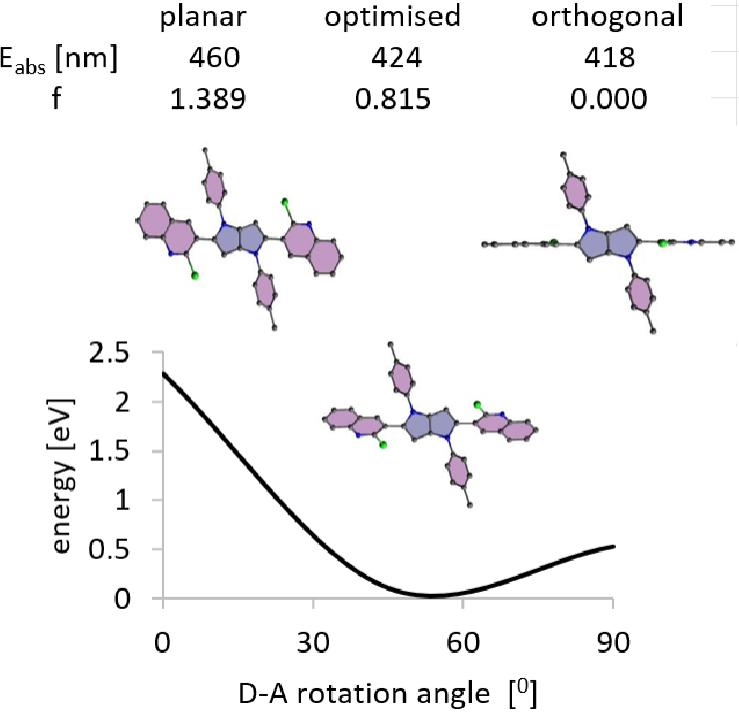
Potential energy curve of molecule **4** in S_0_ state as a function of changes in the mutual orientation of the planes of the DHPP donor and acceptors. At the top, the absorption energy values together with the oscillator strengths, corresponding to the minimum geometry and extreme cases of coplanar and orthogonal donor and acceptor planes.

However, differences in the electron affinity of the acceptor are not the only reason for differences in spectroscopic properties of the tested molecules. In the case of such complex molecules, with the possibility of rotation of the components around the bonds connecting them, an important factor influencing the spectroscopic properties of these compounds is the mutual orientation of the planes of the individual elements. An interesting observation can be derived from probing the changes in the energy and the oscillator strength of the absorption depending on the mutual orientation of the donor and acceptors using compound **4** as an example (Figure 6). According to the results of the optimization of the molecular structure, the energy minimum for this compound corresponds to the geometry in which the angle between the planes of acceptors and donor is 43°. That means that in this geometry, a balance is achieved between the stabilizing role of interactions between donor and acceptors and the destabilizing role of steric interactions. This dye absorbs in DCM at 424 nm with an oscillator strength of 0.815 (Table 2). Both quantities change when moving away from equilibrium by changing the donor–acceptor angle (see Figure 6). In the case of orthogonality of the donor and acceptor planes (the system practically is not stabilized by the interactions between them), the absorption transition would be at higher energy at 418 nm with zero oscillator strength. In turn, in the planar case, the interaction of the donor with the acceptors is the strongest, which is reflected in the absorption at 460 nm with *f* = 1.389. The planar arrangement is destabilized by strong steric interactions (see the potential energy curve in Figure 6). Collectively, these results highlight that those attractive spectroscopic properties, such as low transition energy and high oscillator strength should emerge upon the formation of a fused system, i.e., **10**. The photophysical data gathered in Tables 1 and S7 corroborate this prediction.

The values of absorption energy and oscillator strength for compound **10** (Table 2) are close to those predicted based on calculations for the planarized version of **4** (Figure 6). This is not true, however, for the pair **6** and **11**. Despite the red shift of **11** relative to **6**, the oscillator strength of the transition between S_0_ and S_1_ in **11** is *f* = 1.02 and is smaller than the oscillator strength in **6** (*f* = 1.79). These computational predictions are fully corroborated by comparison of experimental molar absorption coefficients (Tables 1 and S7). Therefore, when creating a fused system, an additional circumstance appears that affects its spectroscopic properties.

**Figure 7:**
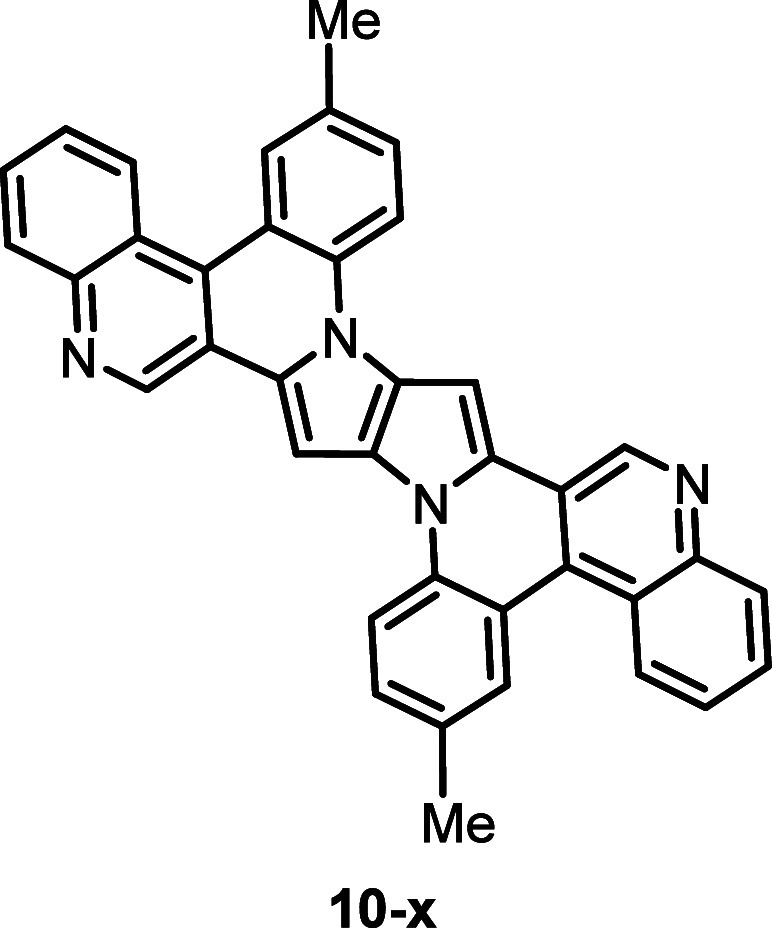
Structure of dye 10-x designed for computational purpose.

The oscillator strength is a quantity strongly determined by the shape of the molecular orbitals. Therefore, the reasons for such discrepancies can be sought in differences in the extension of the charge distribution to atoms of moieties originating from electron-withdrawing moieties, which occurs in fused dyes. To confirm this, calculations were performed for the compound **10-x** (Figure 7), which is an isomer of **10** with a geometric structure analogous to **11** (for comparison of the both structures see Table S3).The calculated oscillator strength (*f* = 0.748) for fused system in geometry (**10-x)** is smaller than oscillator strength (*f* = 0.815) for nonfused molecule **4** and smaller than *f* = 1.203 for fused **10.** Therefore, the relationships between the transition energies and oscillator strengths of **10-x** and **4** are similar as the relations between **11** with **6** (see Table 2). Comparison of the orbitals **10** and **10-x** is given in Table S9 and indicates that the orbitals of **10-x** are more spread to the *N*-aryl substituents than the orbital of **10**.

## Conclusions

We have provided experimental evidence that the efficient synthesis of quinoline derivatives enables not only preparation of 1,4-dihydropyrrrolo[3,2-*b*]pyrroles possessing planarized structures based on tetrazole but also previously inaccessible large planar *N*-doped nanographenes based on quinolines. The emission range of these prepared centrosymmetric, polarized TAPPs can be modulated from green to yellow, orange, and red, and their efficiency are typically moderate to high. Planarization of the chromophore structure results in considerable red shift of the emission wavelength (quinoline-derived dyes show strong yellow fluorescence and dyes possessing two tetrazoloquinoline units exhibit red emission). The two-photon absorption spectrum follows, in most cases, the Laporte rule, which is in accord with the inversion-symmetric structure, with the peak cross-section values reaching σ_2PA_ = 250 GM in the case of TAPP possessing two tetrazoloquinoline substituents at positions 2 and 5. Critically, subtle changes in geometry of these π-extended dyes have a profound effect on their photophysics. Computational studies suggest that intersystem crossing yield is responsible for variation for modulation of fluorescence intensity, thus confirming the notion that planarization of the geometry of polarized centrosymmetric dyes affords control over the molecular excited state. The change in the absorption strength between weakly coupled (biaryl bridges) and fused quadrupolar, centrosymmetric dyes is different for quinoline derivatives and for tetrazoloquinoline derivatives. In the latter case, the molar absorption coefficient actually decreases after planarization of the entire chromophore. The computational investigation enabled us to postulate that this striking difference has its origin in different orientation of the electron-deficient heterocyclic moiety, which causes the spread of the localization of both HOMO and LUMO on *N*-aryl moiety leading in turn to decrease in molar absorptivity. The relative position of singlet and triplet excited states plays a dominant role in determining the fate of the molecules after excitation. In particular, it is responsible for relatively small fluorescence quantum yield for bis(quinolinyl)dyes. Collectively, this work demonstrates that a tetrazole scaffold can be efficiently incorporated into the structure of quadrupolar, centrosymmetric functional dyes furnishing the latter with outstanding photophysical characteristics.

## Experimental Section

### General Information

All chemicals were bought from Sigma-Aldrich, TCI, Ambeed, and AlfaAesar and were used as received unless otherwise noted. All solvents used for reactions were analysis grade and were used without further purification. 2-Chloroquinoline-3-carbaldehyde derivatives **1**,^37^ tetrazolo[1,5-*a*]quinoline-4-carbaldehyde **5**,^38^ 2-bromo-4-dodecylaniline,^39^ and 9-amino-10-bromophenanthrene^40^ were synthesized according to literature procedures. All reactions requiring heating were carried out using an oil bath. Reaction progress was monitored by thin layer chromatography (TLC), which was performed on aluminum foil plates, covered with Silica gel 60 F254 (Merck). The identity and purity of prepared compounds were proved by ^1^H NMR and ^13^C NMR spectroscopy, as well as by MS spectrometry (via APCI-MS or EI-MS). NMR spectra were measured on Varian 500 MHz and Varian 600 MHz instruments. Chemical shifts for ^1^H NMR are expressed in parts per million (ppm) relative to tetramethylsilane (δ 0.00 ppm), CDCl_3_ (δ 7.26 ppm), CD_2_Cl_2_ (δ 5.32 ppm), tetrachloroethane-[D_2_] (δ 5.91 ppm), C_6_D_6_ (δ 7.16 ppm), and THF-[D_8_] (δ 1.73 and 3.58 ppm). Chemical shifts for^13^C NMR are expressed in ppm relative to CDCl_3_ (δ 77.2 ppm), CD_2_Cl_2_ (δ 54.0 ppm), tetrachloroethane-[D_2_] (δ 73.7 ppm), C_6_D_6_ (δ 128.4 ppm), and THF-[D_8_] (δ 25.4 and 67.6 ppm). Data are reported as follows: chemical shift, multiplicity (s = singlet, d = doublet, dd = doublet of doublets, *t* = triplet, td = triplet of doublets, q = quartet, quint = quintet, sex = sextet, br. s = broad singlet, m = multiplet), coupling constant (in Hz), and integration. Because of the very low solubility of compounds **6a**, **6c**–**6e**, **6g,** and **10a**, their ^13^C NMR spectra have been measured at high temperature in tetrachloroethane-[D_2_]. Compounds **6f** and **10b** are not sufficiently soluble in common NMR solvents such as CDCl_3_, acetone-[D_6_], DMSO-[D_6_], CD_3_CN, methanol-[D_4_], and tetrachloroethane-[D_2_] (even after heating) to allow for measurement of ^13^C NMR spectra.

All melting points for crystalline products were measured with automated melting point apparatus EZ-MELT and were given without correction.

Spectrophotometric grade solvents were used without further purification. All photophysical studies were performed with freshly prepared, air equilibrated solutions at room temperature. Steady-state fluorescence measurements were performed in standard 1 cm quartz cuvettes with dilute solutions (10^–6^ M, optical density <0.1) to minimize inner filter effects and/or aggregation. Absorption spectra were measured using a UV–vis Shimadzu UV-3600i Plus spectrophotometer. The calculation of molar absorption coefficient was conducted from Beer–Lambert’s law equation. Emission spectra were measured using Edinburgh Instruments FS5 spectrofluorometer equipped with photomultiplier Hamamatsu R123456. Fluorescence quantum yields (Φ) were calculated from the equation:
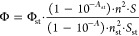
1

where *A* denotes absorbance, *n* is a refractive index of a solvent, and *S* is an integrated fluorescence intensity.

### Typical Procedure for the Synthesis of 3,3′-(1,4-Bis(4-(*tert*-butyl)phenyl)-1,4-dihydropyrrolo[3,2-*b*]pyrrole-2,5-diyl)bis(2-chloroquinoline) (4a)

Glacial acetic acid (2 mL), toluene (2 mL), 2-chloroquinoline-3-carbaldehyde (383 mg, 2 mmol, 2 equiv), and 4-(*tert*-butyl)aniline (299 mg, 2 mmol, 2 equiv) were placed in a 50 mL round-bottom flask equipped with a magnetic stir bar. The mixture reacted at 50 °C for 1 h. After that time, Fe(ClO_4_)_3_·*x*H_2_O (22 mg, 6 mol %) was added, followed by butane-2,3-dione (87 μL, 1 mmol, 1 equiv). The resulting mixture was stirred at 50 °C (oil bath) in an open flask under air for 2 h. Next, the heater was removed, and 5 mL of methanol was added to the reaction mixture, and the resulting mixture was stirred for 10 min. The precipitate was filtered, washed with methanol (3 mL) and diethyl ether (5 mL), and recrystallized from a dichloromethane/hexane mixture and dried under vacuum affording 278 mg (40%) of pure product **4a** as a yellow solid.

The procedure for the synthesis of compounds **4b**–**4l** is similar to that of compound **4a**.

#### 3,3′-(1,4-Bis(4-(*tert*-butyl)phenyl)-1,4-dihydropyrrolo[3,2-*b*]pyrrole-2,5-diyl)bis(2-chloroquinoline) (4a)

Yellow solid (278 mg, 40%); m.p.: 333–334 °C; ^1^H NMR (600 MHz, CDCl_3_) δ 8.11 (d, *J* = 0.9 Hz, 2H), 8.03 (td, *J* = 8.8, 0.9 Hz, 2H), 7.74–7.70 (m, 4H), 7.55–7.52 (m, 2H), 7.28 (d, *J* = 9.0 Hz, 4H), 7.18 (d, *J* = 9.0 Hz, 4H), 6.59 (s, 2H), 1.26 (s, 18H); ^13^C{^1^H} NMR (151 MHz, CDCl_3_) δ 150.5, 148.7, 146.4, 140.2, 136.9, 130.9, 130.6, 130.5, 128.2, 127.5, 127.5, 127.3, 126.8, 126.2, 123.7, 97.6, 34.5, 31.3; HRMS (APCI): *m*/*z* calculated for C_44_H_39_Cl_2_N_4_: 693.2552 [M + H]^+^; found: 693.2549.

#### 3,3′-(1,4-Bis(4-(*tert*-butyl)phenyl)-1,4-dihydropyrrolo[3,2-*b*]pyrrole-2,5-diyl)bis(2-chloro-6-methylquinoline) (4b)

Yellow solid (267 mg, 37%); m.p.: 324–325 °C; ^1^H NMR (600 MHz, CDCl_3_) δ 8.02 (s, 2H), 7.90 (d, *J* = 8.6 Hz, 2H), 7.54 (dd, *J* = 8.6, 1.9 Hz, 2H), 7.47 (s, 2H), 7.27 (d, *J* = 8.7 Hz, 4H), 7.18 (d, *J* = 8.6 Hz, 4H), 6.57 (s, 2H), 2.50 (s, 6H), 1.26 (s, 18H); ^13^C{^1^H} NMR (151 MHz, CDCl_3_) δ 149.6, 148.6, 145.2, 139.6, 137.3, 137.0, 132.7, 130.8, 130.8, 127.9, 127.4, 126.9, 126.3, 126.2, 123.6, 97.5, 34.4, 31.3, 21.6; HRMS (APCI): *m*/*z* calculated for C_46_H_43_Cl_2_N_4_: 721.2859 [M + H]^+^; found: 721.2862.

#### 3,3′-(1,4-Bis(3,4,5-trimethoxyphenyl)-1,4-dihydropyrrolo[3,2-*b*]pyrrole-2,5-diyl)bis(2-chloroquinoline) (4c)

Yellow solid (343 mg, 45%); m.p.: 330–331 °C; ^1^H NMR (600 MHz, CDCl_3_) δ 8.14 (s, 2H), 8.04 (d, *J* = 8.6 Hz, 2H), 7.76–7.73 (m, 4H), 7.57 (t, *J* = 7.5 Hz, 2H), 6.60 (s, 2H), 6.50 (s, 4H), 3.80 (s, 6H), 3.58 (s, 12H); ^13^C{^1^H} NMR (126 MHz, CDCl_3_) δ 153.5, 150.6, 146.6, 140.3, 136.1, 135.1, 130.9, 130.9, 130.4, 128.3, 127.5, 127.5, 127.4, 126.6, 101.9, 97.4, 61.0, 56.1; HRMS (APCI): *m*/*z* calculated for C_42_H_35_Cl_2_N_4_O_6_: 761.1934 [M + H]^+^; found: 761.1932.

#### 3,3′-(1,4-Bis(4-heptylphenyl)-1,4-dihydropyrrolo[3,2-*b*]pyrrole-2,5-diyl)bis(2-chloroquinoline) (4d)

Off-white solid (241 mg, 31%); m.p.: 206–207 °C; ^1^H NMR (500 MHz, CDCl_3_) δ 8.09 (s, 2H), 8.04 (d, *J* = 8.5 Hz, 2H), 7.73–7.70 (m, 4H), 7.54 (t, *J* = 7.5 Hz, 2H), 7.17 (d, *J* = 8.0 Hz, 4H), 7.08 (d, *J* = 8.0 Hz, 4H), 6.61 (s, 2H), 2.54 (t, *J* = 7.8 Hz, 4H), 1.59–1.53 (m, 4H), 1.30–1.22 (m, 16H), 0.87 (t, *J* = 6.9 Hz, 6H); ^13^C{^1^H} NMR (126 MHz, CDCl_3_) δ 150.4, 146.4, 140.7, 140.2, 137.1, 130.9, 130.7, 130.5, 129.2, 128.2, 127.5, 127.4, 127.3, 126.8, 124.1, 97.5, 35.4, 31.8, 31.2, 29.2, 29.1, 22.6, 14.1; HRMS (APCI): *m*/*z* calculated for C_50_H_51_Cl_2_N_4_: 777.3491 [M + H]^+^; found: 777.3497.

#### 3,3′-(1,4-Bis(4-heptylphenyl)-1,4-dihydropyrrolo[3,2-*b*]pyrrole-2,5-diyl)bis(2-chloro-6-methylquinoline) (4e)

Off-white solid (282 mg, 35%); m.p.: 253–254 °C; ^1^H NMR (600 MHz, CDCl_3_) δ 7.99 (s, 2H), 7.93 (d, *J* = 8.6 Hz, 2H), 7.55 (dd, *J* = 8.6, 1.9 Hz, 2H), 7.46 (s, 2H), 7.17 (d, *J* = 8.1 Hz, 4H), 7.08 (d, *J* = 8.1 Hz, 4H), 6.59 (s, 2H), 2.54 (t, *J* = 7.8 Hz, 4H), 2.51 (s, 6H), 1.58–1.55 (m, 4H), 1.30–1.23 (m, 16H), 0.86 (t, *J* = 6.8 Hz, 6H); ^13^C{^1^H} NMR (126 MHz, CDCl_3_) δ 149.5, 145.0, 140.6, 139.7, 137.4, 137.1, 132.8, 130.8, 130.8, 129.2, 127.8, 127.3, 126.8, 126.3, 124.1, 97.4, 35.4, 31.8, 31.2, 29.2, 29.1, 22.6, 21.6, 14.1; HRMS (APCI): *m*/*z* calculated for C_52_H_55_Cl_2_N_4_: 805.3804 [M + H]^+^; found: 805.3807.

#### 3,3′-(1,4-Bis(4-decylphenyl)-1,4-dihydropyrrolo[3,2-*b*]pyrrole-2,5-diyl)bis(2-chloroquinoline) (4f)

Off-white solid (311 mg, 36%); m.p.: 203–204 °C; ^1^H NMR (500 MHz, CDCl_3_) δ 8.08 (s, 2H), 8.02 (d, *J* = 8.2 Hz, 2H), 7.73–7.70 (m, 4H), 7.54 (t, *J* = 7.4 Hz, 2H), 7.17 (d, *J* = 8.0 Hz, 4H), 7.08 (d, *J* = 8.0 Hz, 4H), 6.61 (s, 2H), 2.54 (t, *J* = 7.8 Hz, 4H), 1.59–1.53 (m, 4H), 1.30–1.24 (m, 28H), 0.87 (t, *J* = 6.8 Hz, 6H); ^13^C{^1^H} NMR (126 MHz, CDCl_3_) δ 150.5, 146.5, 140.6, 140.2, 137.1, 130.9, 130.7, 130.5, 129.2, 128.3, 127.5, 127.4, 127.2, 126.8, 124.1, 97.4, 35.4, 31.9, 31.2, 29.6, 29.6, 29.5, 29.3, 29.3, 22.7, 14.1; HRMS (APCI): *m*/*z* calculated for C_56_H_63_Cl_2_N_4_: 861.4430 [M + H]^+^; found: 861.4433.

#### 3,3′-(1,4-Bis(4-octylphenyl)-1,4-dihydropyrrolo[3,2-*b*]pyrrole-2,5-diyl)bis(2-chloro-6-hexylquinoline) (4g)

Yellow solid (390 mg, 40%); m.p.: 121–122 °C; ^1^H NMR (500 MHz, CDCl_3_) δ 8.02 (s, 2H), 7.91 (d, *J* = 8.6 Hz, 2H), 7.56 (dd, *J* = 8.7, 2.0 Hz, 2H), 7.46 (d, *J* = 1.9 Hz, 2H), 7.17 (d, *J* = 8.2 Hz, 4H), 7.08 (d, *J* = 8.1 Hz, 4H), 6.58 (s, 2H), 2.76 (t, *J* = 7.8 Hz, 4H), 2.54 (t, *J* = 7.8 Hz, 4H), 1.60–1.53 (m, 4H), 1.71–1.66 (m, 4H), 1.37–1.24 (m, 32H), 0.91–0.85 (m, 12H); ^13^C{^1^H} NMR (126 MHz, CDCl_3_) δ 149.6, 145.4, 142.2, 140.5, 139.7, 137.2, 132.0, 130.9, 130.8, 129.2, 128.0, 127.3, 126.8, 125.7, 124.0, 97.3, 35.9, 35.4, 31.9, 31.7, 31.2, 31.1, 29.4, 29.3, 29.2, 29.0, 22.6 (2 signals), 14.1 (2 signals); HRMS (APCI): *m*/*z* calculated for C_64_H_79_Cl_2_N_4_: 973.5682 [M + H]^+^; found: 973.5696.

#### 3,3′-(1,4-Bis(4-octylphenyl)-1,4-dihydropyrrolo[3,2-*b*]pyrrole-2,5-diyl)bis(2-chloro-6-hexylquinoline) (4h)

Pale yellow solid (348 mg, 32%); m.p.: 108–109 °C; ^1^H NMR (600 MHz, CDCl_3_) δ 8.01 (s, 2H), 7.91 (d, *J* = 8.6 Hz, 2H), 7.55 (d, *J* = 8.6 Hz, 2H), 7.45 (s, 2H), 7.16 (d, *J* = 7.9 Hz, 4H), 7.06 (d, *J* = 7.9 Hz, 4H), 6.57 (s, 2H), 2.75 (t, *J* = 7.7 Hz, 4H), 2.53 (t, *J* = 7.9 Hz, 4H), 1.70–1.65 (m, 4H), 1.56–1.52 (m, 4H), 1.36–1.23 (m, 48H), 0.98–0.84 (m, 12H); ^13^C{^1^H} NMR (151 MHz, CDCl_3_) δ 149.6, 145.3, 142.3, 140.5, 139.7, 137.2, 132.0, 130.8, 130.8, 129.2, 127.9, 127.3, 126.8, 125.7, 124.0, 97.3, 35.9, 35.4, 31.9, 31.7, 31.2, 31.1, 29.6, 29.6, 29.6, 29.4, 29.3, 29.3, 29.0, 22.7, 22.6, 14.1 (2 signals); HRMS (APCI): *m*/*z* calculated for C_72_H_95_Cl_2_N_4_: 1085.6934 [M + H]^+^; found: 1085.6943.

#### 3,3′-(1,4-Bis(4-dodecylphenyl)-1,4-dihydropyrrolo[3,2-*b*]pyrrole-2,5-diyl)bis(2-chloro-7-(methylthio)quinoline) (4i)

Off-white solid (394 mg, 39%); m.p.: 133–134 °C; ^1^H NMR (600 MHz, CDCl_3_) δ 7.96 (s, 2H), 7.68 (s, 2H), 7.54 (d, *J* = 8.6 Hz, 2H), 7.36 (d, *J* = 8.6 Hz, 2H), 7.16 (d, *J* = 7.9 Hz, 4H), 7.07 (d, *J* = 8.0 Hz, 4H), 6.58 (s, 2H), 2.59 (s, 6H), 2.54 (t, *J* = 7.9 Hz, 4H), 1.59–1.52 (m, 4H), 1.27–1.24 (m, 36H), 0.87 (t, *J* = 6.8 Hz, 6H) ^13^C{^1^H} NMR (126 MHz, CDCl_3_) δ 151.0, 147.2, 143.1, 140.6, 139.8, 137.1, 130.8, 130.7, 129.2, 127.2, 126.4, 126.3, 124.2, 124.1, 121.6, 97.3, 35.4, 31.9, 31.2, 29.7, 29.6 (3 signals), 29.5, 29.3 (2 signals), 22.7, 14.9, 14.1; HRMS (APCI): *m*/*z* calculated for C_62_H_75_Cl_2_N_4_S_2_: 1009.4810 [M + H]^+^; found: 1009.4819.

#### 3,3′-(1,4-Bis(3,5-di-*tert*-butylphenyl)-1,4-dihydropyrrolo[3,2-*b*]pyrrole-2,5-diyl)bis(2-chloro-6-hexylquinoline) (4j)

Pale yellow solid (400 mg, 41%); m.p.: 294–295 °C; ^1^H NMR (500 MHz, CDCl_3_) δ 8.04 (s, 2H), 7.88 (d, *J* = 8.6 Hz, 2H), 7.53 (d, *J* = 8.7 Hz, 2H), 7.45 (s, 2H), 7.17 (s, 2H), 7.09 (s, 4H), 6.58 (s, 2H), 2.76 (t, *J* = 7.6 Hz, 4H), 1.70–1.64 (m, 4H), 1.35–1.25 (m, 12H), 1.11 (s, 36H), 0.88 (t, *J* = 6.6 Hz, 6H); ^13^C{^1^H} NMR (151 MHz, THF-D_8_) δ 151.5, 149.8, 145.7, 141.9, 139.8, 139.1, 131.6, 131.1, 130.3, 127.8 (2 signals), 126.9, 125.7, 118.8 (2 signals), 96.3, 35.6, 34.5, 31.7, 31.1, 30.6, 28.8, 22.5, 13.5; HRMS (APCI): *m*/*z* calculated for C_64_H_79_Cl_2_N_4_: 973.5682 [M + H]^+^; found: 973.5690.

#### 3,3′-(1,4-Bis(3,5-di-*tert*-butylphenyl)-1,4-dihydropyrrolo[3,2-*b*]pyrrole-2,5-diyl)bis(2-chloro-6-methylquinoline) (4k)

Pale yellow solid (392 mg, 47%); m.p.: 381–382 °C; ^1^H NMR (500 MHz, CDCl_3_) δ 8.05 (s, 2H), 7.88 (d, *J* = 8.6 Hz, 2H), 7.53 (dd, *J* = 8.6, 1.9 Hz, 2H), 7.17 (t, *J* = 1.8 Hz, 2H), 7.48 (s, 2H), 7.10 (d, *J* = 1.8 Hz, 4H), 6.58 (s, 2H), 2.52 (s, 6H), 1.12 (s, 36H); ^13^C{^1^H} NMR (126 MHz, CDCl_3_) δ 151.8, 149.9, 145.2, 139.7, 138.7, 137.2, 132.7, 131.0, 130.3, 127.8, 127.6, 126.8, 126.2, 119.2, 119.1, 96.6, 34.8, 31.2, 21.6; HRMS (APCI): *m*/*z* calculated for C_54_H_59_Cl_2_N_4_: 833.4117 [M + H]^+^; found: 833.4122.

#### 3,3′-(1,4-Di(naphthalen-1-yl)-1,4-dihydropyrrolo[3,2-*b*]pyrrole-2,5-diyl)bis(2-chloro-6-octylquinoline) (4l)

Pale yellow solid (272 mg, 30%); m.p.: 208–209 °C; ^1^H NMR (600 MHz, CDCl_3_, mixture of atropisomers) δ 8.28 (d, *J* = 8.4 Hz, 1H), 8.11 (d, *J* = 8.4 Hz, 1H), 7.90 (d, *J* = 8.0 Hz, 1H), 7.87 (d, *J* = 8.0 Hz, 1H), 7.83 (s, 1H), 7.81 (s, 1H), 7.79–7.75 (m, 3H), 7.59 (t, *J* = 7.9 Hz, 1H), 7.57–7.47 (m, 3H), 7.45–7.40 (m, 2H), 7.38 (t, *J* = 7.8 Hz, 1H), 7.30 (t, *J* = 7.9 Hz, 1H), 7.25 (s, 3H), 7.21 (s, 1H), 7.19 (s, 1H), 6.38 (s, 1H), 6.36 (s, 1H), 2.64 (t, *J* = 7.9 Hz, 4H), 1.62–1.57 (m, 4H), 1.34–1.17 (m, 20H), 0.86 (t, *J* = 7.1 Hz, 6H); ^13^C{^1^H} NMR (126 MHz, CDCl_3_, mixture of atropisomers) δ 149.3, 145.0, 142.0, 139.1, 135.8, 134.5, 132.9, 132.7, 131.8, 129.9, 129.7, 128.3, 128.2, 127.8, 127.6, 126.7, 126.5, 126.5, 125.6, 125.4, 124.2, 124.1, 98.4, 97.9, 35.8, 31.8, 31.1, 29.4, 29.2, 29.2, 22.6, 14.1; HRMS (APCI): *m*/*z* calculated for C_60_H_59_Cl_2_N_4_: 905.4117 [M + H]^+^; found: 905.4118.

### Typical Procedure for the Synthesis of 4,4′-(1,4-Bis(4-(*tert*-butyl)phenyl)-1,4-dihydropyrrolo[3,2-*b*]pyrrole-2,5-diyl)ditetrazolo[1,5-*a*]quinoline (6a)

Glacial acetic acid (10 mL), toluene (10 mL), tetrazolo[1,5-*a*]quinoline-4-carbaldehyde (396 mg, 2 mmol, 2 equiv), and 4-(*tert*-butyl)aniline (299 mg, 2 mmol, 2 equiv) were placed in a 50 mL round-bottom flask equipped with a magnetic stir bar. The mixture reacted at 50 °C for 1 h. After that time, Fe(ClO_4_)_3_·*x*H_2_O (22 mg, 6 mol %) was added, followed by butane-2,3-dione (87 μL, 1 mmol, 1 equiv). The resulting mixture was stirred at 80 °C (oil bath) in an open flask under air for 2 h. Next, the heater was removed, and 5 mL of methanol was added to the reaction mixture, and the resulting mixture was stirred for 10 min. The precipitate was filtered, washed with methanol (5 mL) and diethyl ether (5 mL), recrystallized from dichloromethane/hexane mixture, and dried under vacuum affording 431 mg (61%) of pure product **6a** as a brown solid.

The procedure for the synthesis of compounds **6b**–**6r** is similar to that of compound **6a**.

#### 4,4′-(1,4-Bis(4-(*tert*-butyl)phenyl)-1,4-dihydropyrrolo[3,2-*b*]pyrrole-2,5-diyl)ditetrazolo[1,5-*a*]quinoline (6a)

Brown solid (431 mg, 61%); m.p.: 310–311 °C (dec.); ^1^H NMR (600 MHz, CDCl_3_) δ 8.61 (d, *J* = 7.8 Hz, 2H), 7.71 (td, *J* = 7.7, 1.1 Hz, 2H), 7.63 (s, 2H), 7.55 (td, *J* = 7.7, 1.1 Hz, 2H), 7.50 (d, *J* = 8.6 Hz, 4H), 7.44 (d, *J* = 7.9 Hz, 2H), 7.40 (d, *J* = 8.6 Hz, 4H), 7.18 (s, 2H), 1.40 (s, 18H); ^13^C{^1^H} NMR (126 MHz, tetrachloroethane-[D_2_]) δ 150.5, 146.9, 136.6, 135.0, 130.0, 129.8, 128.6, 128.6, 128.3, 128.0, 126.9, 125.4, 124.2, 118.0, 116.5, 99.7, 34.7, 31.4; HRMS (APCI): *m*/*z* calculated for C_44_H_39_N_10_: 707.3359 [M + H]^+^; found: 707.3362.

#### 4,4′-(1,4-Bis(4-(*tert*-butyl)phenyl)-1,4-dihydropyrrolo[3,2-*b*]pyrrole-2,5-diyl)bis(7-methyltetrazolo[1,5-*a*]quinoline) (6b)

Brown solid (500 mg, 68%); m.p.: 318–319 °C (dec.); ^1^H NMR (500 MHz, CDCl_3_) δ 8.49 (d, *J* = 8.5 Hz, 2H), 7.61 (s, 2H), 7.53 (dd, *J* = 8.5, 1.7 Hz, 2H), 7.49 (d, *J* = 8.5 Hz, 4H), 7.39 (d, *J* = 8.5 Hz, 4H), 7.21 (s, 2H), 7.11 (s, 2H), 2.47 (s, 6H), 1.40 (s, 18H); ^13^C{^1^H} NMR (126 MHz, CDCl_3_) δ 150.3, 146.8, 138.2, 137.1, 135.1, 131.2, 129.9, 127.9, 127.4, 126.9, 126.8, 125.6, 124.4, 118.2, 116.4, 100.0, 34.7, 31.4, 21.4; HRMS (APCI): *m*/*z* calculated for C_46_H_43_N_10_: 735.3672 [M + H]^+^; found: 735.3674.

#### 4,4′-(1,4-Bis(3,4,5-trimethoxyphenyl)-1,4-dihydropyrrolo[3,2-*b*]pyrrole-2,5-diyl)ditetrazolo[1,5-*a*]quinoline (6c)

Cream solid (504 mg, 65%); m.p.: 322–323 °C (dec.); ^1^H NMR (500 MHz, CDCl_3_) δ 8.63 (d, *J* = 8.3 Hz, 2H), 7.75 (dt, *J* = 8.5, 4.2 Hz, 2H), 7.68 (s, 2H), 7.59 (d, *J* = 4.2 Hz, 4H), 7.33 (s, 2H), 6.72 (s, 4H), 3.97 (s, 6H), 3.77 (s, 12H); ^13^C{^1^H} NMR (126 MHz, tetrachloroethane-[D_2_]) δ 153.6, 146.2, 136.8, 134.4, 134.2, 129.8, 129.2, 128.2, 128.0, 127.8, 127.1, 123.4, 117.1, 116.0, 103.2, 99.0, 60.6, 56.0; HRMS (APCI): *m*/*z* calculated for C_42_H_35_N_10_O_6_: 775.2741 [M + H]^+^; found: 775.2742.

#### 4,4′-(1,4-Bis(2,4-dimethoxyphenyl)-1,4-dihydropyrrolo[3,2-*b*]pyrrole-2,5-diyl)ditetrazolo[1,5-*a*]quinoline (6d)

Yellow solid (443 mg, 62%); m.p.: 316–317 °C (dec.); ^1^H NMR (500 MHz, tetrachloroethane-[D_2_], mixture of atropisomers) δ 7.92 (d, *J* = 8.3 Hz, 2H), 7.08 (dt, *J* = 8.4, 4.1 Hz, 2H), 6.89–6.94 (m, 4H), 6.72–6.79 (m, 3H), 6.64 (d, *J* = 6.7 Hz, 2H), 6.58 (d, *J* = 8.6 Hz, 1H), 6.06 (d, *J* = 2.6 Hz, 1H), 5.96–6.00 (m, 2H), 5.89 (dd, *J* = 8.5, 2.6 Hz, 1H), 3.25 (s, 3H), 3.23 (s, 3H), 3.14 (s, 3H), 2.96 (s, 3H); ^13^C{^1^H} NMR (126 MHz, tetrachloroethane-[D_2_], mixture of atropisomers) δ 159.7, 154.8, 146.3, 134.8, 134.6, 130.5, 130.4, 129.2, 128.8, 128.6, 128.2, 127.9, 127.6, 125.6, 125.0, 123.8, 120.9, 120.8, 118.2, 118.0, 115.9, 104.7, 99.8, 99.7, 99.0, 98.3, 55.6, 55.4, 55.3; HRMS (APCI): *m*/*z* calculated for C_40_H_31_N_10_O_4_: 715.2530 [M + H]^+^; found: 715.2524.

#### 4,4′-(1,4-Bis(2-bromo-4-(*tert*-butyl)phenyl)-1,4-dihydropyrrolo[3,2-*b*]pyrrole-2,5-diyl)ditetrazolo[1,5-*a*]quinoline (6e)

Orange solid (363 mg, 42%); m.p.: 328–329 °C (dec.); ^1^H NMR (500 MHz, tetrachloroethane-[D_2_], mixture of atropisomers) δ 8.47 (d, *J* = 8.3 Hz, 2H), 7.72 (s, 1H), 7.68–7.61 (m, 3H), 7.53–7.42 (m, 6H), 7.40–7.30 (m, 4H), 6.97 (s, 2H), 1.31 (s, 18H); ^13^C{^1^H} NMR (126 MHz, tetrachloroethane-[D_2_], mixture of atropisomers) δ 154.3, 154.2, 146.9, 136.3, 136.3, 135.2, 134.9, 131.5, 131.5, 131.4, 130.2, 129.8, 129.7, 128.8, 128.6, 126.7, 126.6, 126.3, 126.1, 124.6, 122.4, 122.2, 118.6, 118.4, 116.8, 100.6, 100.2, 35.2, 31.5; HRMS (APCI): *m*/*z* calculated for C_44_H_37_Br_2_N_10_: 863.1569 [M + H]^+^; found: 863.1560.

#### 4,4′-(1,4-Bis(2-bromo-4-(*tert*-butyl)phenyl)-1,4-dihydropyrrolo[3,2-*b*]pyrrole-2,5-diyl)bis(7-methyltetrazolo[1,5-*a*]quinoline) (6f)

Orange solid (179 mg, 20%); m.p.: 338–339 °C; ^1^H NMR (600 MHz, tetrachloroethane-[D_2_], mixture of atropisomers) δ 8.38 (d, *J* = 1.3 Hz, 1H), 8.37 (d, *J* = 1.6 Hz, 1H), 7.74 (d, *J* = 2.1 Hz, 1H), 7.69 (d, *J* = 2.1 Hz, 1H), 7.53 (d, *J* = 8.2 Hz, 1H), 7.49 (d, *J* = 2.6 Hz, 2H), 7.48 (s, 1H), 7.47–7.45 (m, 2H), 7.40 (d, *J* = 2.0 Hz, 1H), 7.37 (d, *J* = 8.2 Hz, 1H), 7.12 (s, 1H), 7.10 (s, 1H), 6.93 (s, 1H), 6.92 (s, 1H), 2.40 (s, 6H), 1.34 (s, 9H), 1.34 (s, 9H); HRMS (APCI): *m*/*z* calculated for C_46_H_41_Br_2_N_10_: 891.1882 [M + H]^+^; found: 891.1869.

#### Dimethyl 2,2′-(2,5-bis(7-methyltetrazolo[1,5-*a*]quinolin-4-yl)pyrrolo[3,2-*b*]pyrrole-1,4-diyl)dibenzoate (6g)

Yellow solid (377 mg, 51%); m.p.: 249–250 °C (dec.); ^1^H NMR (500 MHz, tetrachloroethane-[D_2_], mixture of atropisomers) δ 7.81 (d, *J* = 4.2 Hz, 2H), 7.79 (d, *J* = 4.3 Hz, 2H), 7.29 (dt, *J* = 7.9, 1.7 Hz, 2H), 7.00 (dd, *J* = 3.2, 1.3 Hz, 2H), 6.95–6.88 (m, 4H), 6.73 (d, *J* = 1.1 Hz, 2H), 6.58 (d, *J* = 5.6 Hz, 2H), 6.40 (s, 1H), 6.39 (s, 1H), 2.90 (s, 3H), 2.87 (s, 3H), 1.82 (s, 6H); ^13^C{^1^H} NMR (126 MHz, tetrachloroethane-[D_2_], mixture of atropisomers) δ 166.0, 165.8, 145.9, 145.9, 138.0, 138.0, 137.9, 134.5, 134.2, 133.0, 131.1, 131.0, 130.5, 128.9, 128.3, 128.3, 128.1, 127.7, 127.5, 127.1, 126.7, 126.2, 123.6, 123.6, 117.0, 116.9, 115.7, 98.8, 98.6, 52.1, 52.0, 20.8; HRMS (APCI): *m*/*z* calculated for C_42_H_31_N_10_O_4_: 739.2530 [M + H]^+^; found: 739.2525.

#### 4,4′-(1,4-Bis(4-dodecylphenyl)-1,4-dihydropyrrolo[3,2-*b*]pyrrole-2,5-diyl)ditetrazolo[1,5-*a*]quinoline (6h)

Off-white solid (559 mg, 60%); m.p.: 183–184 °C; ^1^H NMR (500 MHz, CDCl_3_) δ 8.55 (d, *J* = 8.2 Hz, 2H), 7.68 (t, *J* = 7.8 Hz, 2H), 7.58 (s, 2H), 7.51 (t, *J* = 7.5 Hz, 2H), 7.42 (d, *J* = 7.5 Hz, 2H), 7.37 (d, *J* = 7.8 Hz, 4H), 7.27 (d, *J* = 7.8 Hz, 4H), 7.14 (s, 2H), 2.69 (t, *J* = 7.6 Hz, 4H), 1.73–1.63 (m, 4H), 1.40–1.34 (m, 8H), 1.33–1.19 (m, 28H), 0.87 (t, *J* = 6.8 Hz, 6H); ^13^C{^1^H}NMR (126 MHz, CDCl_3_) δ 146.8, 142.1, 137.2, 135.2, 129.8, 129.6, 128.6, 128.4, 127.9, 127.5, 125.9, 124.2, 118.1, 116.5, 100.0, 35.5, 31.9, 31.4, 29.7 (4 signals), 29.6, 29.4, 29.3, 22.7, 14.1; HRMS (APCI): *m*/*z* calculated for C_60_H_71_N_10_: 931.5863 [M + H]^+^; found: 931.5862.

#### 4,4′-(1,4-Bis(4-dodecylphenyl)-1,4-dihydropyrrolo[3,2-*b*]pyrrole-2,5-diyl)bis(7-methyltetrazolo[1,5-*a*]quinoline) (6i)

Off-white solid (624 mg, 65%); m.p.: 238–239 °C; ^1^H NMR (500 MHz, CDCl_3_) δ 8.39 (d, *J* = 8.4 Hz, 2H), 7.55 (s, 2H), 7.48 (d, *J* = 8.4 Hz, 2H), 7.36 (d, *J* = 7.8 Hz, 4H), 7.26 (d, *J* = 8.1 Hz, 4H), 7.16 (s, 2H), 7.04 (s, 2H), 2.69 (t, *J* = 7.6 Hz, 4H), 2.46 (s, 6H), 1.73–1.63 (m, 4H), 1.41–1.34 (m, 8H), 1.32–1.21 (m, 28H), 0.87 (t, *J* = 6.8 Hz, 6H); ^13^C{^1^H} NMR (126 MHz, CDCl_3_) δ 146.6, 141.9, 138.1, 137.3, 135.1, 131.1, 129.9, 129.8, 127.8, 127.4, 126.7, 125.8, 124.2, 118.0, 116.3, 99.9, 35.5, 31.9, 31.4, 29.7, 29.7, 29.7, 29.5, 29.4, 29.2, 22.7, 21.3, 14.1; HRMS (APCI): *m*/*z* calculated for C_62_H_75_N_10_: 959.6176 [M + H]^+^; found: 959.6185.

#### 4,4′-(1,4-Bis(4-hexylphenyl)-1,4-dihydropyrrolo[3,2-*b*]pyrrole-2,5-diyl)ditetrazolo[1,5-*a*]quinoline (6j)

Yellow solid (473 mg, 62%); m.p.: 262–263 °C; ^1^H NMR (600 MHz, CDCl_3_) δ 8.60 (d, *J* = 8.8 Hz, 2H), 7.71 (ddd, *J* = 8.4, 7.2, 1.3 Hz, 2H), 7.59 (s, 2H), 7.52 (ddd, *J* = 8.4, 7.2, 1.3 Hz, 2H), 7.46 (d, *J* = 7.1 Hz, 2H), 7.37 (d, *J* = 8.3 Hz, 4H), 7.27 (d, *J* = 8.3 Hz, 4H), 7.18 (s, 2H), 2.68 (t, *J* = 7.6 Hz, 4H), 1.69–1.64 (m, 4H), 1.40–1.28 (m, 12H), 0.90 (t, *J* = 6.9 Hz, 6H); ^13^C{^1^H} NMR (151 MHz, CDCl_3_) δ 146.8, 142.0, 137.2, 135.1, 129.8, 129.6, 128.6, 128.4, 127.9, 127.4, 125.9, 124.1, 118.1, 116.5, 100.0, 35.5, 31.7, 31.4, 28.9, 22.6, 14.1; HRMS (APCI): *m*/*z* calculated for C_48_H_47_N_10_: 763.3985 [M + H]^+^; found: 763.3988.

#### 4,4′-(1,4-Bis(4-hexylphenyl)-1,4-dihydropyrrolo[3,2-*b*]pyrrole-2,5-diyl)bis(7-methyltetrazolo[1,5-*a*]quinoline) (6k)

Yellow solid (475 mg, 60%); m.p.: 256–257 °C; ^1^H NMR (600 MHz, CDCl_3_) δ 8.47 (d, *J* = 8.5 Hz, 2H), 7.57 (s, 2H), 7.51 (dd, *J* = 8.5, 1.8 Hz, 2H), 7.36 (d, *J* = 8.3 Hz, 4H), 7.26 (d, *J* = 8.3 Hz, 4H), 7.22 (s, 2H), 7.11 (s, 2H), 2.69 (t, *J* = 7.6 Hz, 4H), 2.46 (s, 6H), 1.68 (m, 4H), 1.40–1.26 (m, 12H), 0.89 (t, *J* = 6.9 Hz, 6H); ^13^C{^1^H} NMR (151 MHz, CDCl_3_) δ 146.6, 141.9, 138.1, 137.3, 135.1, 131.1, 129.8, 129.8, 127.8, 127.4, 126.7, 125.8, 124.1, 118.0, 116.2, 99.9, 35.5, 31.7, 31.4, 28.9, 22.6, 21.3, 14.1; HRMS (APCI): *m*/*z* calculated for C_50_H_51_N_10_: 791.4298 [M + H]^+^; found: 791.4305.

#### 4,4′-(1,4-Bis(3,5-di-*tert*-butylphenyl)-1,4-dihydropyrrolo[3,2-*b*]pyrrole-2,5-diyl)ditetrazolo[1,5-*a*]quinoline (6l)

Yellow solid (524 mg, 64%); m.p.: 323–324 °C (dec.); ^1^H NMR (600 MHz, CDCl_3_) δ 8.61 (d, *J* = 8.3 Hz, 2H), 7.53 (td, *J* = 8.4, 1.3 Hz, 2H), 7.66 (s, 2H), 7.53 (td, *J* = 8.4, 1.3 Hz, 2H), 7.47 (t, *J* = 1.8 Hz, 2H), 7.38 (d, *J* = 6.6 Hz, 2H), 7.32 (d, *J* = 1.8 Hz, 4H), 7.12 (s, 2H), 1.27 (s, 36H); ^13^C{^1^H} NMR (151 MHz, CDCl_3_) δ 152.8, 147.0, 138.8, 134.8, 129.7, 129.6, 128.6, 128.3, 127.9, 127.3, 124.1, 120.9, 120.7, 118.3, 116.6, 99.5, 35.0, 31.4; HRMS (APCI): *m*/*z* calculated for C_52_H_55_N_10_: 819.4611 [M + H]^+^; found: 819.4608.

#### 4,4′-(1,4-Bis(3,5-di-*tert*-butylphenyl)-1,4-dihydropyrrolo[3,2-*b*]pyrrole-2,5-diyl)bis(7-methyltetrazolo[1,5-*a*]quinoline) (6m)

Yellow solid (443 mg, 61%); m.p.: 307–308 °C (dec.); ^1^H NMR (600 MHz, CDCl_3_, mixture of atropisomers) δ 8.49 (d, *J* = 8.4 Hz, 2H), 7.61 (s, 2H), 7.52 (d, *J* = 8.4 Hz, 2H), 7.45 (t, *J* = 1.9 Hz, 2H), 7.31 (s, 4H), 7.16 (s, 2H), 7.09 (s, 2H), 2.48 (s, 6H), 1.27 (s, 36H); ^13^C{^1^H} NMR (151 MHz, CDCl_3_, mixture of atropisomers) δ 152.7, 146.9, 138.8, 138.0, 134.6, 131.2, 129.6, 127.7, 127.4, 126.8, 124.1, 120.8, 120.5, 118.2, 116.4, 99.5, 35.0, 31.3, 21.4; HRMS (APCI): *m*/*z* calculated for C_54_H_59_N_10_: 847.4924 [M + H]^+^; found: 847.4923.

#### 4,4′-(1,4-Bis(2-bromo-4-(*tert*-butyl)phenyl)-1,4-dihydropyrrolo[3,2-*b*]pyrrole-2,5-diyl)bis(7-hexyltetrazolo[1,5-*a*]quinoline) (6n)

Yellow solid (496 mg, 48%); m.p.: 291–292 °C (dec.); ^1^H NMR (500 MHz, CDCl_3_) δ 8.49 (s, 1H), 8.47 (s, 1H), 7.86 (d, *J* = 2.1 Hz, 1H), 7.76 (d, *J* = 2.1 Hz, 1H), 7.62 (d, *J* = 2.8 Hz, 2H), 7.57 (d, *J* = 8.2 Hz, 1H), 7.52 (s, 1H), 7.51 (s, 1H), 7.39 (dd, *J* = 8.3, 2.1 Hz, 1H), 7.28 (s, 1H), 7.17 (s, 1H), 7.13 (s, 1H), 6.98 (s, 1H), 6.97 (s, 2H), 2.71 (t, *J* = 6.9 Hz, 4H), 1.67–1.59 (m, 4H), 1.42 (s, 9H), 1.41 (s, 9H), 1.34–1.26 (m, 12H), 0.88 (t, *J* = 6.4 Hz, 6H); ^13^C{^1^H} NMR (126 MHz, CDCl_3_) δ 153.7, 153.6, 146.6, 146.6, 143.2, 143.2, 136.5, 136.4, 135.0, 134.6, 131.2, 131.2, 131.1, 130.6, 130.6, 129.8, 129.7, 127.3, 127.2, 127.1, 127.0, 126.2, 126.1, 126.0, 125.6, 124.5, 122.2, 122.1, 118.4, 118.1, 116.4, 116.4, 100.8, 100.0, 35.7, 35.0, 35.0, 31.6, 31.2, 28.8, 28.8, 22.5, 14.0; HRMS (APCI): *m*/*z* calculated for C_56_H_61_Br_2_N_10_: 1031.3442 [M + H]^+^; found: 1031.3451.

#### 4,4′-(1,4-Bis(2-bromo-4-dodecylphenyl)-1,4-dihydropyrrolo[3,2-*b*]pyrrole-2,5-diyl)bis(7-methyltetrazolo[1,5-*a*]quinoline) (6o)

Yellow solid (581 mg, 52%); m.p.: 271–272 °C; ^1^H NMR (500 MHz, CDCl_3_) δ 8.49 (d, *J* = 8.3 Hz, 2H), 7.69 (s, 1H), 7.60 (s, 3H), 7.56 (d, *J* = 7.6 Hz, 1H), 7.54 (s, 1H), 7.52 (s, 1H), 7.32 (d, *J* = 7.9 Hz, 1H), 7.24 (s, 2H), 7.23–7.16 (m, 2H), 7.03 (s, 2H), 2.74 (t, *J* = 7.4 Hz, 2H), 2.72 (t, *J* = 7.4 Hz, 2H), 2.49 (s, 6H), 1.77–1.66 (m, 4H), 1.46–1.36 (m, 8H), 1.35–1.21 (m, 28H), 0.90 (t, *J* = 6.7 Hz, 6H); ^13^C{^1^H} NMR (126 MHz, CDCl_3_) δ 145.3, 138.1, 136.6, 135.1, 134.7, 133.9, 131.2, 130.0, 129.0, 128.0, 126.1, 125.4, 124.5, 118.3, 116.4, 100.8, 100.0, 35.3, 31.9, 31.2, 29.7, 29.7, 29.7, 29.6, 29.5, 29.4, 29.2, 22.7, 21.3, 14.1; HRMS (APCI): *m*/*z* calculated for C_62_H_73_Br_2_N_10_: 1115.4386 [M + H]^+^; found: 1115.4421.

#### 4,4′-(1,4-Bis(2-bromo-4-dodecylphenyl)-1,4-dihydropyrrolo[3,2-*b*]pyrrole-2,5-diyl)bis(7-octyltetrazolo[1,5-*a*]quinoline) (6p)

Pale yellow solid (618 mg, 47%); m.p.: 245–246 °C (dec.); ^1^H NMR (500 MHz, CDCl_3_, mixture of atropisomers) δ 8.57–8.46 (m, 2H), 7.70–7.52 (m, 6H), 7.34–7.18 (m, 6H), 7.08–7.02 (m, 2H), 2.83–2.65 (m, 8H), 1.77–1.63 (m, 8H), 1.46–1.16 (m, 56H), 0.95–0.81 (m, 12H); ^13^C{^1^H} NMR (126 MHz, CDCl_3_, mixture of atropisomers) δ 146.6 (2 signals), 145.4, 145.3, 143.2, 143.1, 139.1, 136.6, 135.1, 134.6, 133.9 (2 signals), 131.3, 131.2, 130.5 (2 signals), 130.0, 129.9, 129.1, 128.9, 127.4, 127.3, 127.0, 126.3, 125.6, 124.5, 124.4, 122.1, 122.0, 118.4, 118.2, 116.5, 116.4, 100.8, 100.0, 35.7, 35.3, 31.9, 31.8, 31.4, 31.3 (2 signals), 29.7 (3 signals), 29.6 (2 signals), 29.4 (2 signals), 29.3, 29.2, 22.7, 22.6, 14.1 (2 signals); HRMS (APCI): *m*/*z* calculated for C_76_H_101_Br_2_N_10_: 1311.6577 [M + H]^+^; found: 1311.6583.

#### 4,4′-(1,4-Bis(2-bromonaphthalen-1-yl)-1,4-dihydropyrrolo[3,2-*b*]pyrrole-2,5-diyl)bis(7-hexyltetrazolo[1,5-*a*]quinoline) (6q)

Pale yellow solid (439 mg, 43%); m.p.: 227–228 °C (dec.); ^1^H NMR (500 MHz, CDCl_3_, mixture of atropisomers) δ 8.38 (dd, *J* = 8.5, 2.8 Hz, 2H), 7.98 (d, *J* = 8.8 Hz, 2H), 7.95 (d, *J* = 8.8 Hz, 2H), 7.90 (d, *J* = 8.8 Hz, 1H), 7.87 (d, *J* = 8.8 Hz, 1H), 7.64 (d, *J* = 8.4 Hz, 1H), 7.61 (d, *J* = 4.7 Hz, 2H), 7.59–7.51 (m, 3H), 7.48 (d, *J* = 7.7 Hz, 2H), 7.44 (d, *J* = 8.2 Hz, 2H), 6.96 (s, 1H), 7.00 (s, 1H), 6.93 (s, 1H), 6.90 (s, 1H), 2.66–2.58 (m, 4H), 1.62–1.51 (m, 4H), 1.33–1.22 (m, 12H), 0.83–0.87 (m, 6H); ^13^C{^1^H} NMR (126 MHz, CDCl_3_, mixture of atropisomers) δ 146.3, 143.1, 135.3, 135.0, 134.9, 133.4 (2 signals), 132.8, 132.7, 132.5, 132.4, 130.5 (2 signals), 130.1, 128.8 (2 signals), 128.3, 127.3 (2 signals), 127.0, 124.8, 124.7, 124.3, 123.3, 123.1, 122.4, 122.3, 118.1, 116.3, 100.2, 100.1, 35.6, 31.6, 31.3, 28.8, 22.6, 14.1; HRMS (APCI): *m*/*z* calculated for C_56_H_49_Br_2_N_10_: 1019.2508 [M + H]^+^; found: 1019.2520.

#### 4,4′-(1,4-Bis(10-bromophenanthren-9-yl)-1,4-dihydropyrrolo[3,2-*b*]pyrrole-2,5-diyl)bis(7-octyltetrazolo[1,5-*a*]quinoline) (6r)

Brown solid (424 mg, 36%); m.p.: 231–232 °C (dec.); ^1^H NMR (500 MHz, CDCl_3_) δ 9.23 (s, 2H), 8.91 (s, 2H), 8.63 (d, *J* = 8.2 Hz, 4H), 8.50 (d, *J* = 8.6 Hz, 2H), 8.39 (d, *J* = 8.0 Hz, 2H), 7.87 (d, *J* = 8.2 Hz, 2H), 7.83 (s, 2H), 7.89–7.63 (m, 8H), 7.49 (t, *J* = 7.5 Hz, 2H), 2.75 (t, *J* = 7.7 Hz, 4H), 1.74–1.62 (m, 4H), 1.40–1.22 (m, 20H), 0.88 (t, *J* = 6.7 Hz, 6H); ^13^C{^1^H} NMR (126 MHz, CDCl_3_) δ 160.2, 146.7, 146.3, 143.7, 133.5, 132.0, 130.6, 129.8, 129.6, 129.3 (2 signals), 128.4, 127.9, 127.5, 127.2, 127.1, 126.4, 124.6, 123.6, 122.8, 122.6, 120.5, 116.6, 108.6, 35.6, 31.8, 31.1, 29.4, 29.2 (2 signals), 22.7, 14.1; HRMS (APCI): *m*/*z* calculated for C_68_H_61_Br_2_N_10_: 1175.3442 [M + H]^+^; found: 1175.3443.

### Typical Procedure for the Synthesis of 7,19-Didodecyl-2,14-dihexyldibenzo[*b*,*h*]benzo[5′,6’]quino[2″,3″:7′,8’]indolizino[3′,2′:4,5]pyrrolo[2,1-*f*]-1,6-naphthyridine (10a)

A Schenk flask was charged with chloroquinoline-containing pyrrolopyrrole **4h** (44.0 mg, 0.04 mmol, 1 equiv), Ph_3_P (10.5 mg, 0.04 mmol, 1 equiv), Cs_2_CO_3_ (58.5 mg, 0.18 mmol, 4.5 equiv), and Pd(OAc)_2_ (3.6 mg, 40 mol %). Then, dry *m*-xylene (3 mL) was added, and the resulting mixture was degassed 3 times (by evacuation and refilling with argon) and stirred at 160 °C overnight. After cooling to around 80 °C, toluene (10 mL) was added, and the resulting suspension was passed through a pad of Celite. Next, the product was washed from the Celite pad with boiling toluene, all filtrates were concentrated to *ca.* 2 mL and 36.5 mg (90%) of **10a** as orange solid was filtered off. The dried product **10a** obtained was pure enough for all analytical purposes.

The procedure for the synthesis of compounds **10b** and **10c** is similar to that of compound **10a**.

#### 7,19-Didodecyl-2,14-dihexyldibenzo[*b*,*h*]benzo[5′,6’]quino[2″,3″:7′,8’]indolizino[3′,2′:4,5]pyrrolo[2,1-*f*]-1,6-naphthyridine (10a)

Orange solid (36.5 mg, 90%); m.p.: 305–306 °C (dec.); ^1^H NMR (500 MHz, Benzene-*d*_6_) δ 9.35 (d, *J* = 7.2 Hz, 2H), 8.30 (dd, *J* = 8.5, 3.9 Hz, 2H), 8.05 (d, *J* = 8.4 Hz, 2H), 7.81–7.78 (m, 2H), 7.47–7.43 (m, 4H), 7.31–7.29 (m, 2H), 6.97 (s, 1H), 6.96 (d, *J* = 11.0 Hz, 1H), 2.79 (t, *J* = 7.9 Hz, 4H), 2.71 (t, *J* = 7.8 Hz, 4H), 1.84–1.79 (m, 4H), 1.74–1.71 (m, 4H), 1.53–1.24 (m, 48H), 0.97 (t, *J* = 6.8 Hz, 6H), 0.91 (t, *J* = 6.8 Hz, 6H); ^13^C{^1^H} NMR (126 MHz, tetrachloroethane-[D_2_]) δ 145.7, 143.9, 141.0, 137.7, 134.6, 130.6, 130.3, 129.2, 127.8, 126.8, 126.4, 125.7, 125.1, 122.2, 120.4, 114.8, 99.7, 89.3, 36.0, 35.7, 31.9, 31.8, 31.4, 30.8, 29.7, 29.6 (2 signals), 29.3, 29.1, 22.6, 14.0, 13.9; HRMS (APCI): *m*/*z* calculated for C_72_H_93_N_4_: 1013.7400 [M + H]^+^; found: 1013.7426.

#### 7,19-Didodecyl-3,15-bis(methylthio)dibenzo[*b*,*h*]benzo[5′,6’]quino[2″,3″:7′,8’]indolizino[3′,2′:4,5]pyrrolo[2,1-*f*]-1,6-naphthyridine (10b)

Red solid (14.7 mg, 39%); m.p.: 323–324 °C (dec.); ^1^H NMR (500 MHz, tetrachloroethane-[D_2_]) δ 8.76 (s, 2H), 8.06–8.20 (br. s, 2H), 7.71–7.69 (m, 4H), 7.55–7.48 (br. s, 2H), 7.44 (d, *J* = 8.2 Hz, 2H), 7.29 (d, *J* = 8.2 Hz, 2H), 6.85–6.99 (br. s, 2H), 2.83 (t, *J* = 7.9 Hz, 4H), 2.64 (s, 6H), 1.82–1.85 (m, 4H), 1.50–1.55 (m, 4H), 1.43–1.49 (m, 4H), 1.26–1.40 (m, 28H), 0.87 (t, *J* = 6.8 Hz, 6H); HRMS (APCI): *m*/*z* calculated for C_62_H_73_N_4_S_2_: 937.5277 [M + H]^+^; found: 937.5278.

#### 2,16-Dioctylbenzo[*b*]naphtho[1,2-*h*]naphtho[1″,2″:5′,6’]quino[2″,3″:7′,8’]indolizino[3′,2′:4,5]pyrrolo[2,1-*f*]-1,6-naphthyridine (10c)

Orange solid (11.7 mg, 35%); m.p.: 295–296 °C (dec.); ^1^H NMR (500 MHz, CDCl_3_) δ 9.25 (d, *J* = 7.1 Hz, 2H), 9.20 (d, *J* = 8.6 Hz, 2H), 8.52 (s, 2H), 8.07 (d, *J* = 8.6 Hz, 2H), 8.05 (dd, *J* = 8.6, 2.4 Hz, 2H), 7.90 (d, *J* = 8.6 Hz, 2H), 7.75–7.72 (m, 4H), 7.62 (s, 2H), 7.52 (dd, *J* = 8.6, 1.9 Hz, 2H), 7.21 (s, 2H), 2.80 (t, *J* = 7.8 Hz, 4H), 1.76–1.73 (m, 4H), 1.44–1.24 (m, 20H), 0.88 (t, *J* = 6.7 Hz, 6H); ^13^C{^1^H} NMR (126 MHz, CDCl_3_) δ 146.2, 144.4, 141.3, 135.6, 133.1, 131.9, 131.8, 130.8, 129.3, 128.5, 127.8, 127.4, 126.2, 125.4, 124.8, 124.6, 124.5, 123.4, 122.8, 121.1, 120.8, 95.5, 36.0, 31.9, 31.1, 29.5, 29.4, 29.3, 22.7, 14.1; HRMS (APCI): *m*/*z* calculated for C_60_H_57_N_4_: 833.4583 [M + H]^+^; found: 833.4585.

### Typical Procedure for the Synthesis of 10,24-Didodecyl-7,21-dimethyldibenzo[*c*,*f*]benzo[5′,6’]tetrazolo[1‴,5‴:1″,2″]quino[4″,3″:7′,8’]indolizino[3′,2′:4,5]pyrrolo[2,1-*a*]tetrazolo[1,5-*h*]-2,7-naphthyridine (11a)

A Schenk flask was charged with dibrominated tetrazoloquinoline-containing pyrrolopyrrole **6o** (90.0 mg, 0.08 mmol, 1 equiv), Ph_3_P (21.0 mg, 0.08 mmol, 1 equiv), Cs_2_CO_3_ (117.0 mg, 0.36 mmol, 4.5 equiv), and Pd(OAc)_2_ (7.2 mg, 40 mol %). Then, dry *m*-xylene (6 mL) was added, the resulting mixture degassed 3 times (by evacuation and refilling with Argon) and stirred at 160 °C for 20 h. After cooling to around 80 °C, toluene (10 mL) was added and resulting suspension was passed through a pad of Celite. Next, the product was washed from the Celite pad with boiling toluene, and all filtrates were concentrated to *ca.* 1 mL. Next, 1 mL of chloroform was added, and the flask with reaction mixture was moved to the fridge. Upon 8 h, 35.0 mg (45%) of compound **11a** was filtered as dark purple fine crystals. The dried product **11a** obtained was pure enough for all analytical purposes.

The procedure for the synthesis of compounds **11b**–**11d** is similar to that of compound **11a**, except that compounds **11c** and **11d** were purified by column chromatography (SiO_2_, EtOAc:hexanes, 20:80, than 25:75).

#### 10,24-Didodecyl-7,21-dimethyldibenzo[*c*,*f*]benzo[5′,6’]tetrazolo[1‴,5‴:1″,2″]quino[4″,3″:7′,8’]indolizino[3′,2′:4,5]pyrrolo[2,1-*a*]tetrazolo[1,5-*h*]-2,7-naphthyridine (11a)

Dark purple solid (35.0 mg, 45%); m.p.: 296–297 °C (dec.); ^1^H NMR (600 MHz, CDCl_3_) δ 8.40 (s, 2H), 8.33 (d, *J* = 8.1 Hz, 2H), 8.08 (s, 2H), 7.89 (s, 2H), 7.76 (d, *J* = 8.1 Hz, 2H), 7.43 (d, *J* = 8.2 Hz, 2H), 7.23 (d, *J* = 8.2 Hz, 2H), 2.68 (t, *J* = 7.9 Hz, 4H), 2.58 (s, 6H), 1.75–1.70 (m, 4H), 1.44–1.40 (m, 4H), 1.39–1.24 (m, 32H), 0.88 (t, *J* = 6.9 Hz, 6H); ^13^C{^1^H} NMR (126 MHz, CDCl_3_) δ 144.5, 137.5, 137.1, 133.2, 130.7, 129.9, 128.4, 127.7, 127.2, 127.0, 124.0, 122.6, 121.8, 117.0, 115.7, 111.7, 92.7, 35.6, 31.9, 31.4, 31.4, 30.2, 29.8, 29.7, 29.7, 29.4, 29.4, 22.7, 21.9, 14.1; HRMS (APCI): *m*/*z* calculated for C_62_H_71_N_10_: 955.5863 [M + H]^+^; found: 955.5852.

#### 10,24-Didodecyl-7,21-dioctyldibenzo[*c*,*f*]benzo[5′,6’]tetrazolo[1‴,5‴:1″,2″]quino[4″,3″:7′,8’]indolizino[3′,2′:4,5]pyrrolo[2,1-*a*]tetrazolo[1,5-*h*]-2,7-naphthyridine (11b)

Dark purple solid (43.3 mg, 47%); m.p.: 312–313 °C (dec.); ^1^H NMR (500 MHz, CDCl_3_) δ 8.42 (s, 2H), 8.37 (d, *J* = 8.2 Hz, 2H), 8.11 (s, 2H), 7.98 (s, 2H), 7.87 (d, *J* = 8.2 Hz, 2H), 7.45 (dd, *J* = 8.3, 1.5 Hz, 2H), 7.31 (d, *J* = 8.2 Hz, 2H), 2.80 (t, *J* = 7.9 Hz, 4H), 2.71 (t, *J* = 7.9 Hz, 4H), 1.83–1.78 (m, 4H), 1.76–1.70 (m, 4H), 1.52–1.25 (m, 56H), 0.91 (t, *J* = 7.2 Hz, 6H), 0.86 (t, *J* = 7.2 Hz, 6H); ^13^C{^1^H} NMR (126 MHz, CDCl_3_) δ 144.3, 142.3, 136.9, 133.0, 130.6, 129.1, 128.0, 127.8, 127.5, 127.2, 126.8, 122.4, 121.6, 118.0, 116.8, 115.5, 111.4, 92.6, 36.2, 35.7, 32.0, 31.9, 31.7, 31.5, 29.8, 29.8, 29.8, 29.7, 29.7, 29.6, 29.5, 29.4, 29.4, 22.7, 22.7, 14.1, 14.2; HRMS (APCI): *m*/*z* calculated for C_76_H_98_N_10_: 1150.7976 [M]^+^; found: 1150.7977.

#### 7,23-Dihexylbenzo[*c*]naphtho[2,1-*f*]naphtho[1″,2″:5′,6’]tetrazolo[1‴,5‴:1″,2″]quino[4″,3″:7′,8’]indolizino[3′,2′:4,5]pyrrolo[1,2-*h*]tetrazolo[5,1-*a*]-2,7-naphthyridine (11c)

Purple solid (26.8 mg, 39%); m.p.: 301–302 °C (dec.); ^1^H NMR (500 MHz, CDCl_3_) δ 8.97 (d, *J* = 8.7 Hz, 2H), 8.95 (s, 2H), 8.58 (d, *J* = 8.4 Hz, 2H), 8.40 (d, *J* = 8.9 Hz, 2H), 7.71 (dd, *J* = 8.2, 1.6 Hz, 2H), 7.63 (d, *J* = 8.0 Hz, 2H), 7.59 (d, *J* = 8.8 Hz, 2H), 7.44–7.39 (br. s, 2H), 7.35 (t, *J* = 7.7 Hz, 2H), 7.29 (t, *J* = 7.7 Hz, 2H), 3.14 (t, *J* = 7.8 Hz, 4H), 2.09–2.04 (m, 4H), 1.70–1.64 (m, 4H), 1.58–1.55 (m, 2H), 1.53–1.51 (m, 2H), 1.49–1.42 (m, 4H), 0.98 (t, *J* = 7.2 Hz, 6H); ^13^C{^1^H} NMR (151 MHz, CD_2_Cl_2_) δ 149.1, 144.6, 142.9, 133.1, 132.1, 130.1, 128.9, 128.6, 128.5, 127.9, 127.7, 124.8, 124.4, 124.0, 123.8, 123.5, 122.7, 120.3, 117.6, 113.4, 111.7, 95.5, 36.6, 32.2, 31.9, 29.6, 23.2, 14.3; HRMS (APCI): *m*/*z* calculated for C_56_H_47_N_10_: 859.3985 [M + H]^+^; found: 859.3992.

#### 7-Octyl-19-(7-octyltetrazolo[1,5-*a*]quinolin-4-yl)-20-(9-phenanthryl)-20*H*-benzo[*c*]phenanthro[9,10-*f*]pyrrolo[2′,3′:4,5]pyrrolo[1,2-*h*]tetrazolo[5,1-*a*]-2,7-naphthyridine (11d)

Purple solid (21.2 mg, 26%); m.p.: 286–287 °C (dec.); ^1^H NMR (600 MHz, CDCl_3_) δ 9.40–9.38 (m, 1H); 8.87–7.29 (complex multiplets, 24H), 6.83 (d, *J* = 8.4 Hz, 1H), 2.53–2.48 (m, 4H), 1.50–1.43 (m, 4H), 1.31–1.17 (m, 20H), 0.88–0.83 (m, 6H); ^13^C{^1^H}NMR (126 MHz, CD_2_Cl_2_) δ 145.3, 143.2, 141.6, 140.0, 139.8, 135.6, 134.6, 131.9, 131.6, 131.3, 130.8, 130.5, 130.4, 130.4, 130.2, 129.8, 129.5, 129.4, 129.1, 129.0, 127.9, 127.8, 127.7, 127.6, 127.5, 127.4, 127.4, 127.3, 127.2, 127.1, 126.8, 126.7, 126.3, 126.2, 126.2, 125.6, 124.7, 124.5, 124.0, 124.0, 123.8, 123.5, 123.3, 122.9, 122.1, 117.9, 116.7, 116.0, 112.5, 105.3, 105.0, 91.4, 91.2, 35.8, 35.4, 31.8, 31.8, 31.2, 29.7, 29.3, 29.3, 29.1, 29.1, 29.1, 22.6, 22.6, 13.9, 13.8; HRMS (APCI): *m*/*z* calculated for C_68_H_61_N_10_: 1017.5081 [M + H]^+^; found: 1017.5085.

## Data Availability

The data underlying this study are available in the published article and its Supporting Information.

## Supporting Information Available

Theoretical data; typical procedure for the synthesis of **4a**, **6a**, **10a**, **11a**; spectral data, ^1^H and ^13^C NMR spectra, optical properties, and photophysical data of **4a**–**4l**, **6a**–**6r**, **10a**–**10c**, **11a**–**11d**; crystallographic data of **4k**, **6j**, **10a**, **11d** (PDF)
