# Supplementary material for: Strongly Polarized π-Extended 1,4-Dihydropyrrolo[3,2-b]pyrroles Fused with Tetrazolo[1,5-a]quinolines
Source: J Org Chem. 2024 Mar 26;89(7):4657–72. doi: 10.1021/acs.joc.3c02916 (PMC11002929; doi:10.1021/acs.joc.3c02916)
Supplement: Supplementary file 1 — jo3c02916_si_001.pdf [file jo3c02916_si_001.pdf]

Strongly Polarized π-Extended 1,4-Dihydropyrrolo[3,2-*b*]pyrroles Fused with Tetrazolo[1,5-*a*]quinolines

Mohammad B. Teimouri,[1,2]* Irena Deperasińska,[3] Matt Rammo,[4] Marzena Banasiewicz,[3] Charles W. Stark,[4] Łukasz Dobrzycki,[5] Michał K. Cyrański,[5]* Aleksander Rebane,[4,6]* Daniel T. Gryko[1]*

[1] *Institute of Organic Chemistry, Polish Academy of Sciences, Kasprzaka 44/52 01-224 Warsaw, Poland.*

[2] *Faculty of Chemistry, Kharazmi University, Mofateh Ave, 15719-14911 Tehran, Iran.*

[3] *Institute of Physics of Polish Academy of Sciences, Al. Lotników 32/46, 02-668 Warsaw, Poland.*

[4] *National Institute for Chemical Physics and Biophysics, Akadeemia tee 23, Tallinn,12618, Estonia.*

[5] *University of Warsaw, Faculty of Chemistry, Pasteura 1, 02-093 Warsaw, Poland.*

[6] *Department of Physics, Montana State University, Bozeman, MT 59717, USA.*

teimouri@khu.ac.ir

mkc@chem.uw.edu.pl

arebane@montana.edu

dtgryko@icho.edu.pl

Table of Contents

1. General remarks S2
2. Typical procedure for the synthesis of **4a** S3
3. Spectral data for **4a-4l** S4
4. Typical procedure for the synthesis of **6a** S10
5. Spectral data for **6a-4r** S10
6. Typical procedure for the synthesis of **10a** S19
7. Spectral data for **10a-10c** S19
8. Typical procedure for the synthesis of **11a** S21
9. Spectral data for **11a-11d** S21
10. ^{1}H and ^{13}C NMR spectra for **4a-4l** S24
11. ^{1}H and ^{13}C NMR spectra for **6a-rl** S42
12. ^{1}H and ^{13}C NMR spectra for **10a-10c** S69
13. ^{1}H and ^{13}C NMR spectra for **11a-11d**....S72
14. Optical properties **4a-4l**....S79
15. Photophysical data for dyes **4a-4l**....S88
16. Optical properties **6a-6r**.... S89
17. Photophysical data for dyes **6a-6r**....S103
18. Optical properties **10a-10c** and **11a-11d** S104
19. Photophysical data for dyes **10a-10c** and **11a-11d**....S109
20. Crystallographic data of **4k**, **6j** and **10a** S110
21. Crystallographic data of **11d** S114
22. Theoretical calculations S118
23. Optical properties of compounds **4d**, **4h**, **4i**, **4k**, **6a**, **6e**, **6i**, and **6o** (in solid state) S122
24. References S124

General remarks

All chemicals were bought from Sigma Aldrich, TCI, Ambeed and AlfaAesar, and were used as received unless otherwise noted. All used for reaction solvents were pure for analysis grade and were taken without further purification. 2-Chloroquinoline-3-carbaldehyde derivatives **1** [1], tetrazolo[1,5-*a*]quinoline-4-carbaldehyde **5** [2], 2-bromo-4-dodecylaniline [3], and 9-amino-10-bromophenanthrene [4] were synthesized according to literature procedures. All reactions requiring heating were carried out using an oil bath. The reaction progress was monitored by means of thin layer chromatography (TLC), which was performed on aluminium foil plates, covered with Silica gel 60 F254 (Merck). The identity and purity of prepared compounds were proved by ^{1}H NMR and ^{13}C NMR spectroscopy as well as by MS spectrometry (*via* APCI-MS or EI-MS). NMR spectra were measured on Varian 500 MHz and Varian 600 MHz instruments. Chemical shifts for ^{1}H NMR are expressed in parts per million (ppm) relative to tetramethylsilane (δ 0.00 ppm), $CDCl_3$ (δ 7.26 ppm), CD_2Cl_2 (δ 5.32 ppm), tetrachloroethane-[D_2] (δ 5.91 ppm), C_6D_6 (δ 7.16 ppm), THF-[D_8] (δ 1.73 & 3.58 ppm). Chemical shifts for ^{13}C NMR are expressed in ppm relative to $CDCl_3$ (δ 77.2 ppm), CD_2Cl_2 (δ 54.0 ppm), tetrachloroethane-[D_2] (δ 73.7 ppm), C_6D_6 (δ 128.4 ppm), THF-[D_8] (δ 25.4 & 67.6 ppm). Data are reported as follows: chemical shift, multiplicity (s = singlet, d = doublet, dd = doublet of doublets, t = triplet, td = triplet of doublets, q = quartet, quint = quintet, sex = sextet, br. s = broad singlet, m = multiplet), coupling constant (in Hz), and integration. Because of the very low solubility of compounds **6a**, **6c**-**6e**, **6g** and **10a**, their ^{13}C NMR spectra have been measured at high temperature in tetrachloroethane-[D_2]. Compounds **6f** and **10b** are not sufficiently soluble in common NMR solvents such as $CDCl_3$, acetone-[D_6], DMSO-[D_6], CD_3CN, methanol-[D_4] and tetrachloroethane-[D_2] (even after heating) to allow for measurement of ^{13}C NMR spectra.

All melting points for crystalline products were measured with automated melting point apparatus EZ-MELT and were given without correction.

Two-photon measurements

Spectroscopic samples were prepared in 2 mL 1 cm path length quartz cuvettes for 2PA measurements. Toluene (OmniSolv, UN 1593, DX 0831-6, 99.96%) were used. The sample concentrations used in the 2PEF measurements were ~1 μM. For samples with high quantum yields the relative 2PA spectra were obtained using the 2PEF method. A Ti:Sapphire femtosecond laser system (Coherent, Libra) operated at 1 kHz repetition rate and producing pulses with duration ~100 fs pumped an optical parametric amplifier (1PA) (Light Conversion, OPerA Solo). The 1PA output wavelength was tuned in the wavelength region 570–900 nm with 2 nm steps. The approximate 1PA pulse spectral width was ~ 15–35 nm. For detection of the fluorescent signal, a grating spectrometer (Jobin-Yvon, Triax 550) combined with a CCD detector (Spectrum One) was used. Bis-diphenylaminostilbene (bDPAS) diluted in dichloromethane was used as the reference standard for the 2PEF measurements. Briefly, for the NLT measurements, the same laser setup was employed, but the pulse repetition rate was reduced to 100 Hz. The 1PA beam was additionally collimated using a series of apertures and lenses.

Typical procedure for the synthesis of 3,3'-(1,4-bis(4-(*tert*-butyl)phenyl)-1,4-dihydropyrrolo[3,2-*b*]pyrrole-2,5-diyl)bis(2-chloroquinoline) (4a):

Glacial acetic acid (2 mL), toluene (2 mL), 2-chloroquinoline-3-carbaldehyde (383 mg, 2 mmol, 2 *eq.*) and 4-(*tert*-butyl)aniline (299 mg, 2 mmol, 2 *eq.*) were placed in a 50 mL round-bottom flask equipped with a magnetic stir bar. The mixture reacted at 50 °C for 1 h. After that time, $Fe(ClO_4)_3 \cdot xH_2O$ (22 mg, 6 mol%) was added, followed by butane-2,3-dione (87 μL, 1 mmol, 1 *eq.*). The resulting mixture was stirred at 50 °C (oil bath) in an open flask under air for 2 h. Next,

the heater was removed, and 5 mL of methanol was added to the reaction mixture, and the resulting mixture was stirred for 10 min. The precipitate was filtered off, washed with methanol (3 mL) and diethyl ether (5 mL), and recrystallized from dichloromethane/hexanes mixture and dried under vacuum affording 278 mg (40%) of pure product **4a** as a yellow solid.

The procedure for the synthesis of compounds **4b-4l** is similar to compound **4a**.

3,3'-(1,4-Bis(4-(*tert*-butyl)phenyl)-1,4-dihydropyrrolo[3,2-*b*]pyrrole-2,5-diyl)bis(2-chloroquinoline) (4a):

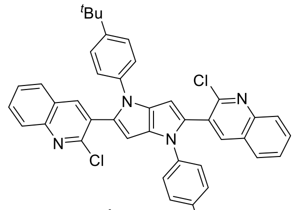

4a

Yellow solid (278 mg, 40%); m.p.: 333-334 °C; ^{1}H NMR (600 MHz, $CDCl_3$) δ 8.11 (d, *J* = 0.9 Hz, 2H), 8.03 (td, *J* = 8.8, 0.9 Hz, 2H), 7.74-7.70 (m, 4H), 7.55-7.52 (m, 2H), 7.28 (d, *J* = 9.0 Hz, 4H), 7.18 (d, *J* = 9.0 Hz, 4H), 6.59 (s, 2H), 1.26 (s, 18H); ^{13}C{^{1}H} NMR (151 MHz, $CDCl_3$) δ 150.5, 148.7, 146.4, 140.2, 136.9, 130.9, 130.6, 130.5, 128.2, 127.5, 127.5, 127.3, 126.8, 126.2, 123.7, 97.6, 34.5, 31.3; HRMS (APCI): m/z calculated for $C_{44}H_{39}Cl_2N_4$: 693.2552 $[M+H]^+$; found: 693.2549.

3,3'-(1,4-Bis(4-(*tert*-butyl)phenyl)-1,4-dihydropyrrolo[3,2-*b*]pyrrole-2,5-diyl)bis(2-chloro-6-methylquinoline) (4b):

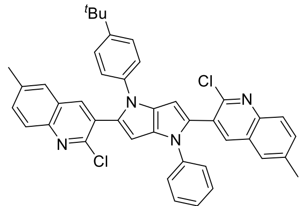

4b

Yellow solid (267 mg, 37%); m.p.: 324-325 °C; ^{1}H NMR (600 MHz, $CDCl_3$) δ 8.02 (s, 2H), 7.90 (d, *J* = 8.6 Hz, 2H), 7.54 (dd, *J* = 8.6, 1.9 Hz, 2H), 7.47 (s, 2H), 7.27 (d, *J* = 8.7 Hz, 4H), 7.18 (d, *J* = 8.6 Hz, 4H), 6.57 (s, 2H), 2.50 (s, 6H), 1.26 (s, 18H); ^{13}C{^{1}H} NMR (151 MHz, $CDCl_3$) δ 149.6, 148.6, 145.2, 139.6, 137.3, 137.0, 132.7, 130.8, 130.8, 127.9, 127.4, 126.9, 126.3, 126.2, 123.6, 97.5, 34.4, 31.3, 21.6; HRMS (APCI): m/z calculated for $C_{46}H_{43}Cl_2N_4$: 721.2865 $[M+H]^+$; found: 721.2862.

3,3'-(1,4-Bis(3,4,5-trimethoxyphenyl)-1,4-dihydropyrrolo[3,2-*b*]pyrrole-2,5-diyl)bis(2-chloroquinoline) (4c):

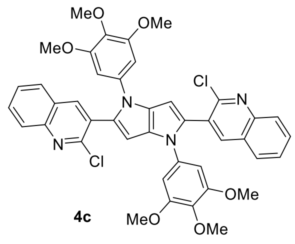

4c

Yellow solid (343 mg, 45%); m.p.: 330-331 °C; ^{1}H NMR (600 MHz, $CDCl_3$) δ 8.14 (s, 2H), 8.04 (d, *J* = 8.6 Hz, 2H), 7.76-7.73 (m, 4H), 7.57 (t, *J* = 7.5 Hz, 2H), 6.60 (s, 2H), 6.50 (s, 4H), 3.80 (s, 6H), 3.58 (s, 12H); ^{13}C{^{1}H} NMR (126 MHz, $CDCl_3$) δ 153.5, 150.6, 146.6, 140.3, 136.1, 135.1, 130.9, 130.9, 130.4, 128.3, 127.5, 127.5, 127.4, 126.6, 101.9, 97.4, 61.0, 56.1; HRMS (APCI): m/z calculated for $C_{42}H_{35}Cl_2N_4O_6$: 761.1934 [M+H]$^+$; found: 761.1932.

3,3'-(1,4-Bis(4-heptylphenyl)-1,4-dihydropyrrolo[3,2-*b*]pyrrole-2,5-diyl)bis(2-chloroquinoline) (4d):

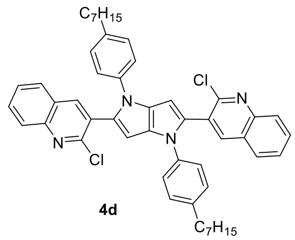

4d

Off-white solid (241 mg, 31%); m.p.: 206-207 °C; ^{1}H NMR (500 MHz, $CDCl_3$) δ 8.09 (s, 2H), 8.04 (d, *J* = 8.5 Hz, 2H), 7.73-7.70 (m, 4H), 7.54 (t, *J* = 7.5 Hz, 2H), 7.17 (d, *J* = 8.0 Hz, 4H), 7.08 (d, *J* = 8.0 Hz, 4H), 6.61 (s, 2H), 2.54 (t, *J* = 7.8 Hz, 4H), 1.59-1.53 (m, 4H), 1.30-1.22 (m, 16H), 0.87 (t, *J* = 6.9 Hz, 6H); ^{13}C{^{1}H} NMR (126 MHz, $CDCl_3$) δ 150.4, 146.4, 140.7, 140.2, 137.1, 130.9, 130.7, 130.5, 129.2, 128.2, 127.5, 127.4, 127.3, 126.8, 124.1, 97.5, 35.4, 31.8, 31.2, 29.2, 29.1, 22.6, 14.1; HRMS (APCI): m/z calculated for $C_{50}H_{51}Cl_2N_4$: 777.3491 [M+H]$^+$; found: 777.3497.

3,3'-(1,4-Bis(4-heptylphenyl)-1,4-dihydropyrrolo[3,2-b]pyrrole-2,5-diyl)bis(2-chloro-6-methylquinoline) (4e):

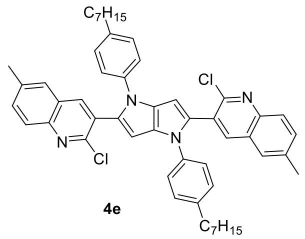

4e

Off-white solid (282 mg, 35%); m.p.: 253-254 °C; ^{1}H NMR (600 MHz, $CDCl_3$) δ 7.99 (s, 2H), 7.93 (d, *J* = 8.6 Hz, 2H), 7.55 (dd, *J* = 8.6, 1.9 Hz, 2H), 7.46 (s, 2H), 7.17 (d, *J* = 8.1 Hz, 4H), 7.08 (d, *J* = 8.1 Hz, 4H), 6.59 (s, 2H), 2.54 (t, *J* = 7.8 Hz, 4H), 2.51 (s, 6H), 1.58-1.55 (m, 4H), 1.30-1.23 (m, 16H), 0.86 (t, *J* = 6.8 Hz, 6H); $^{13}C\{^{1}H\}$ NMR (126 MHz, $CDCl_3$) δ 149.5, 145.0, 140.6, 139.7, 137.4, 137.1, 132.8, 130.8, 130.8, 129.2, 127.8, 127.3, 126.8, 126.3, 124.1, 97.4, 35.4, 31.8, 31.2, 29.2, 29.1, 22.6, 21.6, 14.1; HRMS (APCI): m/z calculated for $C_{52}H_{55}Cl_2N_4$: 805.3804 $[M+H]^+$; found: 805.3807.

3,3'-(1,4-Bis(4-decylphenyl)-1,4-dihydropyrrolo[3,2-*b*]pyrrole-2,5-diyl)bis(2-chloroquinoline) (4f):

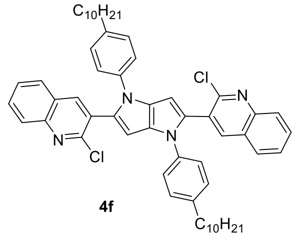

4f

Off-white solid (311 mg, 36%); m.p.: 203-204 °C; ^{1}H NMR (500 MHz, $CDCl_3$) δ 8.08 (s, 2H), 8.02 (d, *J* = 8.2 Hz, 2H), 7.73-7.70 (m, 4H), 7.54 (t, *J* = 7.4 Hz, 2H), 7.17 (d, *J* = 8.0 Hz, 4H), 7.08 (d, *J* = 8.0 Hz, 4H), 6.61 (s, 2H), 2.54 (t, *J* = 7.8 Hz, 4H), 1.59-1.53 (m, 4H), 1.30-1.24 (m, 28H), 0.87 (t, *J* = 6.8 Hz, 6H); $^{13}C\{^{1}H\}$ NMR (126 MHz, $CDCl_3$) δ 150.5, 146.5, 140.6, 140.2, 137.1, 130.9, 130.7, 130.5, 129.2, 128.3, 127.5, 127.4, 127.2, 126.8, 124.1, 97.4, 35.4, 31.9, 31.2, 29.6, 29.6, 29.5, 29.3, 29.3, 22.7, 14.1; HRMS (APCI): m/z calculated for $C_{56}H_{63}Cl_2N_4$: 861.4430 $[M+H]^+$; found: 861.4433.

3,3'-(1,4-Bis(4-octylphenyl)-1,4-dihydropyrrolo[3,2-*b*]pyrrole-2,5-diyl)bis(2-chloro-6-hexylquinoline) (4g):

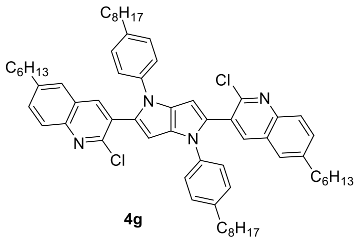

4g

Yellow solid (390 mg, 40%); m.p.: 121-122 °C; ^{1}H NMR (500 MHz, $CDCl_3$) δ 8.02 (s, 2H), 7.91 (d, *J* = 8.6 Hz, 2H), 7.56 (dd, *J* = 8.7, 2.0 Hz, 2H), 7.46 (d, *J* = 1.9 Hz, 2H), 7.17 (d, *J* = 8.2 Hz, 4H), 7.08 (d, *J* = 8.1 Hz, 4H), 6.58 (s, 2H), 2.76 (t, *J* = 7.8 Hz, 4H), 2.54 (t, *J* = 7.8 Hz, 4H), 1.60-1.53 (m, 4H), 1.71-1.66 (m, 4H), 1.37-1.24 (m, 32H), 0.91-0.85 (m, 12H); $^{13}C\{^1H\}$ NMR (126 MHz, $CDCl_3$) δ 149.6, 145.4, 142.2, 140.5, 139.7, 137.2, 132.0, 130.9, 130.8, 129.2, 128.0, 127.3, 126.8, 125.7, 124.0, 97.3, 35.9, 35.4, 31.9, 31.7, 31.2, 31.1, 29.4, 29.3, 29.2, 29.0, 22.6 (2 signals), 14.1 (2 signals); HRMS (APCI): m/z calculated for $C_{64}H_{79}Cl_2N_4$: 973.5682 $[M+H]^+$; found: 973.5696.

3,3'-(1,4-Bis(4-octylphenyl)-1,4-dihydropyrrolo[3,2-b]pyrrole-2,5-diyl)bis(2-chloro-6-hexylquinoline) (4h):

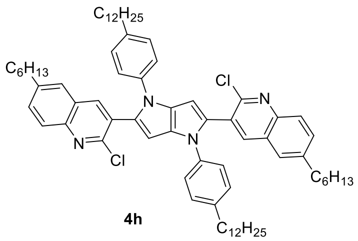

4h

Pale yellow solid (348 mg, 32%); m.p.: 108-109 °C; ^{1}H NMR (600 MHz, $CDCl_3$) δ 8.01 (s, 2H), 7.91 (d, *J* = 8.6 Hz, 2H), 7.55 (d, *J* = 8.6 Hz, 2H), 7.45 (s, 2H), 7.16 (d, *J* = 7.9 Hz, 4H), 7.06 (d, *J* = 7.9 Hz, 4H), 6.57 (s, 2H), 2.75 (t, *J* = 7.7 Hz, 4H), 2.53 (t, *J* = 7.9 Hz, 4H), 1.70-1.65 (m, 4H), 1.56-1.52 (m, 4H), 1.23-1.36 (m, 48H), 0.89-0.84 (m, 12H); $^{13}C\{^1H\}$ NMR (151 MHz, $CDCl_3$) δ 149.6, 145.3, 142.3, 140.5, 139.7, 137.2, 132.0, 130.8, 130.8, 129.2, 127.9, 127.3, 126.8, 125.7, 124.0, 97.3, 35.9, 35.4, 31.9, 31.7, 31.2, 31.1, 29.6, 29.6, 29.6, 29.4, 29.3, 29.3, 29.0, 22.7, 22.6, 14.1 (2 signals); HRMS (APCI): m/z calculated for $C_{72}H_{95}Cl_2N_4$: 1085.6934 $[M+H]^+$; found: 1085.6943.

3,3'-(1,4-Bis(4-dodecylphenyl)-1,4-dihydropyrrolo[3,2-*b*]pyrrole-2,5-diyl)bis(2-chloro-7-(methylthio)quinoline) (4i):

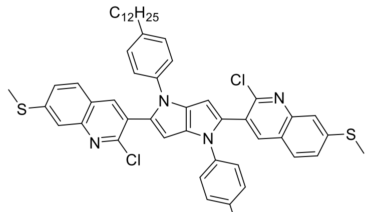

Off-white solid (394 mg, 39%); m.p.: 133-134 °C; ^{1}H NMR (600 MHz, $CDCl_3$) δ 7.96 (s, 2H), 7.68 (s, 2H), 7.54 (d, *J* = 8.6 Hz, 2H), 7.36 (d, *J* = 8.6 Hz, 2H), 7.16 (d, *J* = 7.9 Hz, 4H), 7.07 (d, *J* = 8.0 Hz, 4H), 6.58 (s, 2H), 2.59 (s, 6H), 2.54 (t, *J* = 7.9 Hz, 4H), 1.59-1.52 (m, 4H), 1.27-1.24 (m, 36H), 0.87 (t, *J* = 6.8 Hz, 6H); $^{13}C\{^{1}H\}$ NMR (126 MHz, $CDCl_3$) δ 151.0, 147.2, 143.1, 140.6, 139.8, 137.1, 130.8, 130.7, 129.2, 127.2, 126.4, 126.3, 124.2, 124.1, 121.6, 97.3, 35.4, 31.9, 31.2, 29.7, 29.6 (3 signals), 29.5, 29.3 (2 signals), 22.7, 14.9, 14.1; HRMS (APCI): m/z calculated for $C_{62}H_{75}Cl_2N_4S_2$: 1009.4810 $[M+H]^+$; found: 1009.4819.

3,3'-(1,4-Bis(3,5-di-*tert*-butylphenyl)-1,4-dihydropyrrolo[3,2-*b*]pyrrole-2,5-diyl)bis(2-chloro-6-hexylquinoline) (4j):

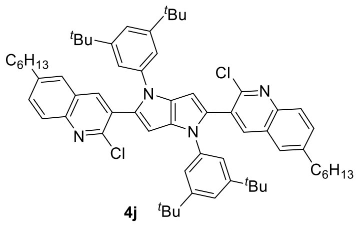

Pale yellow solid (400 mg, 41%); m.p.: 294-295 °C; ^{1}H NMR (500 MHz, $CDCl_3$) δ 8.04 (s, 2H), 7.88 (d, *J* = 8.6 Hz, 2H), 7.53 (d, *J* = 8.7 Hz, 2H), 7.45 (s, 2H), 7.17 (s, 2H), 7.09 (s, 4H), 6.58 (s, 2H), 2.76 (t, *J* = 7.6 Hz, 4H), 1.70-1.64 (m, 4H), 1.35-1.25 (m, 12H), 1.11 (s, 36H), 0.88 (t, *J* = 6.6 Hz, 6H); $^{13}C\{^{1}H\}$ NMR (151 MHz, THF-D_8) δ 151.5, 149.8, 145.7, 141.9, 139.8, 139.1, 131.6, 131.1, 130.3, 127.8 (2 signals), 126.9, 125.7, 118.8 (2 signals), 96.3, 35.6, 34.5, 31.7, 31.1, 30.6, 28.8, 22.5, 13.5; HRMS (APCI): m/z calculated for $C_{64}H_{79}Cl_2N_4$: 973.5682 $[M+H]^+$; found: 973.5690.

3,3'-(1,4-Bis(3,5-di-*tert*-butylphenyl)-1,4-dihydropyrrolo[3,2-*b*]pyrrole-2,5-diyl)bis(2-chloro-6-methylquinoline) 4k:

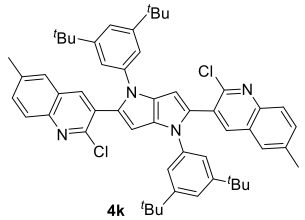

4k

Pale yellow solid (392 mg, 47%); m.p.: 381-382 ˚C; ^{1}H NMR (500 MHz, $CDCl_3$) δ 8.05 (s, 2H), 7.88 (d, *J* = 8.6 Hz, 2H), 7.53 (dd, *J* = 8.6, 1.9 Hz, 2H), 7.17 (t, *J* = 1.8 Hz, 2H), 7.48 (s, 2H), 7.10 (d, *J* = 1.8 Hz, 4H), 6.58 (s, 2H), 2.52 (s, 6H), 1.12 (s, 36H); $^{13}C\{^1H\}$ NMR (126 MHz, $CDCl_3$) δ 151.8, 149.9, 145.2, 139.7, 138.7, 137.2, 132.7, 131.0, 130.3, 127.8, 127.6, 126.8, 126.2, 119.2, 119.1, 96.6, 34.8, 31.2, 21.6; HRMS (APCI): m/z calculated for $C_{54}H_{59}Cl_2N_4$: 833.4117 [M+H]$^+$; found: 833.4122.

3,3'-(1,4-Di(naphthalen-1-yl)-1,4-dihydropyrrolo[3,2-*b*]pyrrole-2,5-diyl)bis(2-chloro-6-octylquinoline) (4l):

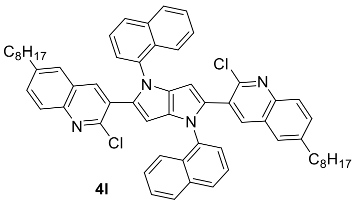

4l

Pale yellow solid (272 mg, 30%); m.p.: 208-209 ˚C; ^{1}H NMR (600 MHz, $CDCl_3$, mixture of atropisomers) δ 8.28 (d, *J* = 8.4 Hz, 1H), 8.11 (d, *J* = 8.4 Hz, 1H), 7.90 (d, *J* = 8.0 Hz, 1H), 7.87 (d, *J* = 8.0 Hz, 1H), 7.83 (s, 1H), 7.81 (s, 1H), 7.79-7.75 (m, 3H), 7.59 (t, *J* = 7.9 Hz, 1H), 7.57–7.47 (m, 3H), 7.45-7.40 (m, 2H), 7.38 (t, *J* = 7.8 Hz, 1H), 7.30 (t, *J* = 7.9 Hz, 1H), 7.25 (s, 3H), 7.21 (s, 1H), 7.19 (s, 1H), 6.38 (s, 1H), 6.36 (s, 1H), 2.64 (t, *J* = 7.9 Hz, 4H), 1.62-1.57 (m, 4H), 1.34–1.17 (m, 20H), 0.86 (t, *J* = 7.1 Hz, 6H); $^{13}C\{^1H\}$ NMR (126 MHz, $CDCl_3$, mixture of atropisomers) δ 149.3, 145.0, 142.0, 139.1, 135.8, 134.5, 132.9, 132.7, 131.8, 129.9, 129.7, 128.3, 128.2, 127.8, 127.6, 126.7, 126.5, 126.5, 125.6, 125.4, 124.2, 124.1, 98.4, 97.9, 35.8, 31.8, 31.1, 29.4, 29.2, 29.2, 22.6, 14.1; HRMS (APCI): m/z calculated for $C_{60}H_{59}Cl_2N_4$: 905.4117 [M+H]$^+$; found: 905.4118.

Typical procedure for the synthesis of 4,4'-(1,4-bis(4-(*tert*-butyl)phenyl)-1,4-dihydropyrrolo[3,2-*b*]pyrrole-2,5-diyl)ditetrazolo[1,5-*a*]quinoline (6a):

Glacial acetic acid (10 mL), toluene (10 mL), tetrazolo[1,5-*a*]quinoline-4-carbaldehyde (396 mg, 2 mmol, 2 *eq*.) and 4-(*tert*-butyl)aniline (299 mg, 2 mmol, 2 *eq.*) were placed in a 50 mL round-bottom flask equipped with a magnetic stir bar. The mixture reacted at 50 °C for 1 h. After that time, $Fe(ClO_4)_3 \cdot xH_2O$ (22 mg, 6 mol%) was added, followed by butane-2,3-dione (87 μl, 1 mmol, 1 *eq.*). The resulting mixture was stirred at 80 °C (oil bath) in an open flask under air for 2 h. Next, the heater was removed, and 5 mL of methanol was added to the reaction mixture, and the resulting mixture was stirred for 10 min. The precipitate was filtered off, washed with methanol (5 mL) and diethyl ether (5 mL), and recrystallized from dichloromethane/hexanes mixture and dried under vacuum affording 431 mg (61%) of pure product **6a** as a brown solid.

The procedure for the synthesis of compounds **6b**-**6r** is similar to compound **6a**.

4,4'-(1,4-Bis(4-(*tert*-butyl)phenyl)-1,4-dihydropyrrolo[3,2-*b*]pyrrole-2,5-diyl)ditetrazolo[1,5-*a*]quinoline (6a):

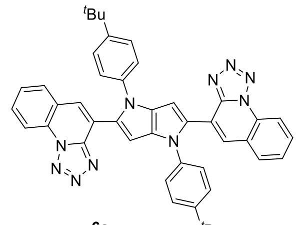

6a

Brown solid (431 mg, 61%); m.p.: 310-311 °C (dec.); ^{1}H NMR (600 MHz, $CDCl_3$) δ 8.61 (d, *J* = 7.8 Hz, 2H), 7.71 (td, *J* = 7.7, 1.1 Hz, 2H), 7.63 (s, 2H), 7.55 (td, *J* = 7.7, 1.1 Hz, 2H), 7.50 (d, *J* = 8.6 Hz, 4H), 7.44 (d, *J* = 7.9 Hz, 2H), 7.40 (d, *J* = 8.6 Hz, 4H), 7.18 (s, 2H), 1.40 (s, 18H); ^{13}C{^{1}H} NMR (126 MHz, tetrachloroethane-[D_2]) δ 150.5, 146.9, 136.6, 135.0, 130.0, 129.8, 128.6, 128.6, 128.3, 128.0, 126.9, 125.4, 124.2, 118.0, 116.5, 99.7, 34.7, 31.4; HRMS (APCI): m/z calculated for $C_{44}H_{39}N_{10}$: 707.3359 [M+H]$^+$; found: 707.3362.

4,4'-(1,4-Bis(4-(*tert*-butyl)phenyl)-1,4-dihydropyrrolo[3,2-*b*]pyrrole-2,5-diyl)bis(7-methyltetrazolo[1,5-*a*]quinoline) (6b):

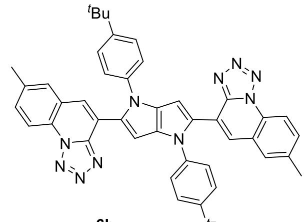

Brown solid (500 mg, 68%); m.p.: 318-319 °C (dec.); ^{1}H NMR (500 MHz, $CDCl_3$) δ 8.49 (d, *J* = 8.5 Hz, 2H), 7.61 (s, 2H), 7.53 (dd, *J* = 8.5, 1.7 Hz, 2H), 7.49 (d, *J* = 8.5 Hz, 4H), 7.39 (d, *J* = 8.5 Hz, 4H), 7.21 (s, 2H), 7.11 (s, 2H), 2.47 (s, 6H), 1.40 (s, 18H); $^{13}C\{^{1}H\}$ NMR (126 MHz, $CDCl_3$) δ 150.3, 146.8, 138.2, 137.1, 135.1, 131.2, 129.9, 127.9, 127.4, 126.9, 126.8, 125.6, 124.4, 118.2, 116.4, 100.0, 34.7, 31.4, 21.4; HRMS (APCI): m/z calculated for $C_{46}H_{43}N_{10}$: 735.3672 $[M+H]^+$; found: 735.3674.

4,4'-(1,4-Bis(3,4,5-trimethoxyphenyl)-1,4-dihydropyrrolo[3,2-*b*]pyrrole-2,5-diyl)ditetrazolo[1,5-*a*]quinoline (6c):

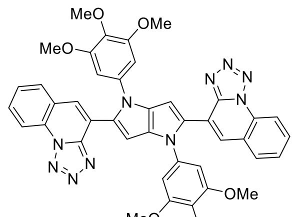

Cream solid (504 mg, 65%); m.p.: 322-323 °C (dec.); ^{1}H NMR (500 MHz, $CDCl_3$) δ 8.63 (d, *J* = 8.3 Hz, 2H), 7.75 (dt, *J* = 8.5, 4.2 Hz, 2H), 7.68 (s, 2H), 7.59 (d, *J* = 4.2 Hz, 4H), 7.33 (s, 2H), 6.72 (s, 4H), 3.97 (s, 6H), 3.77 (s, 12H); $^{13}C\{^{1}H\}$ NMR (126 MHz, tetrachloroethane-[D_2]) δ 153.6, 146.2, 136.8, 134.4, 134.2, 129.8, 129.2, 128.2, 128.0, 127.8, 127.1, 123.4, 117.1, 116.0, 103.2, 99.0, 60.6, 56.0; HRMS (APCI): m/z calculated for $C_{42}H_{35}N_{10}O_6$: 775.2741 $[M+H]^+$; found: 775.2742.

4,4'-(1,4-Bis(2,4-dimethoxyphenyl)-1,4-dihydropyrrolo[3,2-*b*]pyrrole-2,5-diyl)ditetrazolo[1,5-*a*]quinoline (6d):

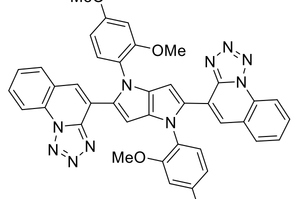

Yellow solid (443 mg, 62%); m.p.: 316-317 °C (dec.); ^{1}H NMR (500 MHz, tetrachloroethane-[D_2], mixture of atropisomers) δ 7.92 (d, *J* = 8.3 Hz, 2H), 7.08 (dt, *J* = 8.4, 4.1 Hz, 2H), 6.94-6.89 (m, 4H), 6.79-6.72 (m, 3H), 6.64 (d, *J* = 6.7 Hz, 2H), 6.58 (d, *J* = 8.6 Hz, 1H), 6.06 (d, *J* = 2.6 Hz, 1H), 6.00-5.96 (m, 2H), 5.89 (dd, *J* = 8.5, 2.6 Hz, 1H), 3.25 (s, 3H), 3.23 (s, 3H),

3.14 (s, 3H), 2.96 (s, 3H); $^{13}C\{^1H\}$ NMR (126 MHz, tetrachloroethane-[D_2], mixture of atropisomers) δ 159.7, 154.8, 146.3, 134.8, 134.6, 130.5, 130.4, 129.2, 128.8, 128.6, 128.2, 127.9, 127.6, 125.6, 125.0, 123.8, 120.9, 120.8, 118.2, 118.0, 115.9, 104.7, 99.8, 99.7, 99.0, 98.3, 55.6, 55.4, 55.3; HRMS (APCI): m/z calculated for $C_{40}H_{31}N_{10}O_4$: 715.2530 $[M+H]^+$; found: 715.2524.

4,4'-(1,4-Bis(2-bromo-4-(*tert*-butyl)phenyl)-1,4-dihydropyrrolo[3,2-*b*]pyrrole-2,5-diyl)ditetrazolo[1,5-*a*]quinoline (6e):

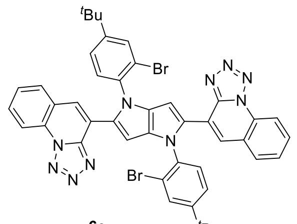

6e

Orange solid (363 mg, 42%); m.p.: 328-329 °C (dec.); ^1H NMR (500 MHz, tetrachloroethane-[D_2], mixture of atropisomers) δ δ 8.47 (d, *J* = 8.3 Hz, 2H), 7.72 (s, 2H), 7.68–7.61 (m, 3H), 7.53-7.42 (m, 6H), 7.40–7.30 (m, 4H), 6.97 (s, 2H), 1.31 (s, 18H); $^{13}C\{^1H\}$ NMR (126 MHz, tetrachloroethane-[D_2], mixture of atropisomers) δ 154.3, 154.2, 146.9, 136.3, 136.3, 135.2, 134.9, 131.5, 131.5, 131.4, 130.2, 129.8, 129.7, 128.8, 128.6, 126.7, 126.6, 126.3, 126.1, 124.6, 122.4, 122.2, 118.6, 118.4, 116.8, 100.6, 100.2, 35.2, 31.5; HRMS (APCI): m/z calculated for $C_{44}H_{37}Br_2N_{10}$: 863.1569 $[M+H]^+$; found: 863.1560.

4,4'-(1,4-Bis(2-bromo-4-(*tert*-butyl)phenyl)-1,4-dihydropyrrolo[3,2-*b*]pyrrole-2,5-diyl)bis(7-methyltetrazolo[1,5-*a*]quinoline) (6f):

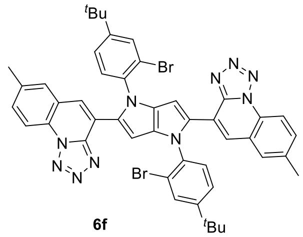

6f

Orange solid (179 mg, 20%); m.p.: 338-339 °C; ^1H NMR (600 MHz, tetrachloroethane-[D_2], mixture of atropisomers) δ 8.38 (d, *J* = 1.3 Hz, 1H), 8.37 (d, *J* = 1.6 Hz, 1H), 7.74 (d, *J* = 2.1 Hz, 1H), 7.69 (d, *J* = 2.1 Hz, 1H), 7.53 (d, *J* = 8.2 Hz, 1H), 7.49 (d, *J* = 2.6 Hz, 2H), 7.48 (s, 1H), 7.47-7.45 (m, 2H), 7.40 (d, *J* = 2.0 Hz, 1H), 7.37 (d, *J* = 8.2 Hz, 1H), 7.12 (s, 1H), 7.10 (s, 1H), 6.93 (s, 1H), 6.92 (s, 1H), 2.40 (s, 6H), 1.34 (s, 9H), 1.34 (s, 9H); HRMS (APCI): m/z calculated for $C_{46}H_{41}Br_2N_{10}$: 891.1882 $[M+H]^+$; found: 891.1869.

Dimethyl 2,2'-(2,5-bis(7-methyltetrazolo[1,5-*a*]quinolin-4-yl)pyrrolo[3,2-*b*]pyrrole-1,4-diyl)dibenzoate (6g):

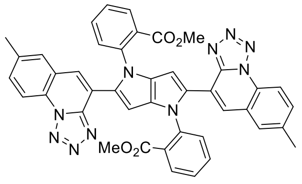

6g

Yellow solid (377 mg, 51%); m.p.: 249-250 °C (dec.); ^{1}H NMR (500 MHz, tetrachloroethane-[D$_2$], mixture of atropisomers) δ 7.81 (d, *J* = 4.2 Hz, 2H), 7.79 (d, *J* = 4.3 Hz, 2H), 7.29 (dt, *J* = 7.9, 1.7 Hz, 2H), 7.00 (dd, *J* = 3.2, 1.3 Hz, 2H), 6.95-6.88 (m, 4H), 6.73 (d, *J* = 1.1 Hz, 2H), 6.58 (d, *J* = 5.6 Hz, 2H), 6.40 (s, 1H), 6.39 (s, 1H), 2.90 (s, 3H), 2.87 (s, 3H), 1.82 (s, 6H); ^{13}C{^{1}H} NMR (126 MHz, tetrachloroethane-[D$_2$], mixture of atropisomers) δ 166.0, 165.8, 145.9, 145.9, 138.0, 138.0, 137.9, 134.5, 134.2, 133.0, 131.1, 131.0, 130.5, 128.9, 128.3, 128.3, 128.1, 127.7, 127.5, 127.1, 126.7, 126.2, 123.6, 123.6, 117.0, 116.9, 115.7, 98.8, 98.6, 52.1, 52.0, 20.8; HRMS (APCI): m/z calculated for $C_{42}H_{31}N_{10}O_4$: 739.2530 [M+H]$^+$; found: 739.2525.

4,4'-(1,4-Bis(4-dodecylphenyl)-1,4-dihydropyrrolo[3,2-*b*]pyrrole-2,5-diyl)ditetrazolo[1,5-*a*]quinoline (6h):

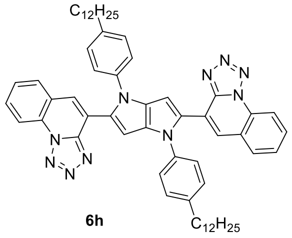

6h

Off-white solid (559 mg, 60%); m.p.: 183-184 °C; ^{1}H NMR (500 MHz, CDCl$_3$) δ 8.55 (d, *J* = 8.2 Hz, 2H), 7.68 (t, *J* = 7.8 Hz, 2H), 7.58 (s, 2H), 7.51 (t, *J* = 7.5 Hz, 2H), 7.42 (d, *J* = 7.5 Hz, 2H), 7.37 (d, *J* = 7.8 Hz, 4H), 7.27 (d, *J* = 7.8 Hz, 4H), 7.14 (s, 2H), 2.69 (t, *J* = 7.6 Hz, 4H), 1.73-1.63 (m, 4H), 1.40-1.34 (m, 8H), 1.33-1.19 (m, 28H), 0.87 (t, *J* = 6.8 Hz, 6H); ^{13}C{^{1}H} NMR (126 MHz, CDCl$_3$) δ 146.8, 142.1, 137.2, 135.2, 129.8, 129.6, 128.6, 128.4, 127.9, 127.5, 125.9, 124.2, 118.1, 116.5, 100.0, 35.5, 31.9, 31.4, 29.7 (4 signals), 29.6, 29.4, 29.3, 22.7, 14.1; HRMS (APCI): m/z calculated for $C_{60}H_{71}N_{10}$: 931.5863 [M+H]$^+$; found: 931.5862.

4,4'-(1,4-Bis(4-dodecylphenyl)-1,4-dihydropyrrolo[3,2-*b*]pyrrole-2,5-diyl)bis(7-methyltetrazolo[1,5-*a*]quinoline) (6i):

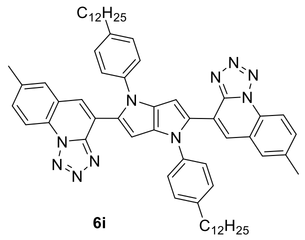

6i

Off-white solid (624 mg, 65%); m.p.: 238-239 °C; ^{1}H NMR (500 MHz, $CDCl_3$) δ 8.39 (d, *J* = 8.4 Hz, 2H), 7.55 (s, 2H), 7.48 (d, *J* = 8.4 Hz, 2H), 7.36 (d, *J* = 7.8 Hz, 4H), 7.26 (d, *J* = 8.1 Hz, 4H), 7.16 (s, 2H), 7.04 (s, 2H), 2.69 (t, *J* = 7.6 Hz, 4H), 2.46 (s, 6H), 1.73-1.63 (m, 4H), 1.41-1.34 (m, 8H), 1.32-1.21 (m, 28H), 0.87 (t, *J* = 6.8 Hz, 6H); $^{13}C\{^1H\}$ NMR (126 MHz, $CDCl_3$) δ 146.6, 141.9, 138.1, 137.3, 135.1, 131.1, 129.9, 129.8, 127.8, 127.4, 126.7, 125.8, 124.2, 118.0, 116.3, 99.9, 35.5, 31.9, 31.4, 29.7, 29.7, 29.7, 29.5, 29.4, 29.2, 22.7, 21.3, 14.1; HRMS (APCI): m/z calculated for $C_{62}H_{75}N_{10}$: 959.6176 $[M+H]^+$; found: 959.6185.

4,4'-(1,4-Bis(4-hexylphenyl)-1,4-dihydropyrrolo[3,2-*b*]pyrrole-2,5-diyl)ditetrazolo[1,5-*a*]quinoline (6j):

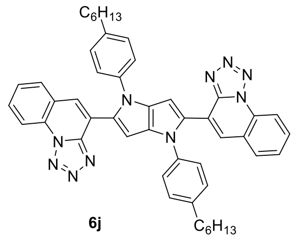

6j

Yellow solid (473 mg, 62%); m.p.: 262-263 °C; ^{1}H NMR (600 MHz, $CDCl_3$) δ δ 8.60 (d, *J* = 8.8 Hz, 2H), 7.71 (ddd, *J* = 8.4, 7.2, 1.3 Hz, 2H), 7.59 (s, 2H), 7.52 (ddd, *J* = 8.4, 7.2, 1.3 Hz, 2H), 7.46 (d, *J* = 7.1 Hz, 2H), 7.37 (d, *J* = 8.3 Hz, 4H), 7.27 (d, *J* = 8.3 Hz, 4H), 7.18 (s, 2H), 2.68 (t, *J* = 7.6 Hz, 4H), 1.69-1.64 (m, 4H), 1.40-1.28 (m, 12H), 0.90 (t, *J* = 6.9 Hz, 6H); $^{13}C\{^1H\}$ NMR (151 MHz, $CDCl_3$) δ 146.8, 142.0, 137.2, 135.1, 129.8, 129.6, 128.6, 128.4, 127.9, 127.4, 125.9, 124.1, 118.1, 116.5, 100.0, 35.5, 31.7, 31.4, 28.9, 22.6, 14.1; HRMS (APCI): m/z calculated for $C_{48}H_{47}N_{10}$: 763.3985 $[M+H]^+$; found: 763.3988.

4,4'-(1,4-Bis(4-hexylphenyl)-1,4-dihydropyrrolo[3,2-*b*]pyrrole-2,5-diyl)bis(7-methyltetrazolo[1,5-*a*]quinoline) (6k):

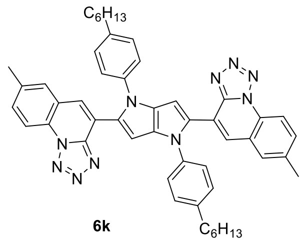

6k

Yellow solid (475 mg, 60%); m.p.: 256-257 °C; ^{1}H NMR (600 MHz, $CDCl_3$) δ 8.47 (d, *J* = 8.5 Hz, 2H), 7.57 (s, 2H), 7.51 (dd, *J* = 8.5, 1.8 Hz, 2H), 7.36 (d, *J* = 8.3 Hz, 4H), 7.26 (d, *J* = 8.3 Hz, 4H), 7.22 (s, 2H), 7.11 (s, 2H), 2.69 (t, *J* = 7.6 Hz, 4H), 2.46 (s, 6H), 1.68 (m, 4H), 1.40-1.26 (m, 12H), 0.89 (t, *J* = 6.9 Hz, 6H); $^{13}C\{^{1}H\}$ NMR (151 MHz, $CDCl_3$) δ 146.6, 141.9, 138.1, 137.3, 135.1, 131.1, 129.8, 129.8, 127.8, 127.4, 126.7, 125.8, 124.1, 118.0, 116.2, 99.9, 35.5, 31.7, 31.4, 28.9, 22.6, 21.3, 14.1; HRMS (APCI): m/z calculated for $C_{50}H_{51}N_{10}$: 791.4298 $[M+H]^+$; found: 791.4305.

4,4'-(1,4-Bis(3,5-di-*tert*-butylphenyl)-1,4-dihydropyrrolo[3,2-*b*]pyrrole-2,5-diyl)ditetrazolo[1,5-*a*]quinoline (6l):

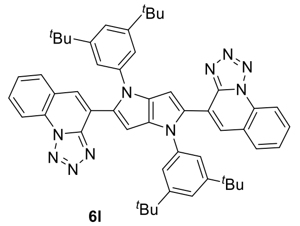

6l

Yellow solid (524 mg, 64%); m.p.: 323-324 °C (dec.); ^{1}H NMR (600 MHz, $CDCl_3$) δ 8.61 (d, *J* = 8.3 Hz, 2H), 7.53 (td, *J* = 8.4, 1.3 Hz, 2H), 7.66 (s, 2H), 7.53 (td, *J* = 8.4, 1.3 Hz, 2H), 7.47 (t, *J* = 1.8 Hz, 2H), 7.38 (d, *J* = 6.6 Hz, 2H), 7.32 (d, *J* = 1.8 Hz, 4H), 7.12 (s, 2H), 1.27 (s, 36H); $^{13}C\{^{1}H\}$ NMR (151 MHz, $CDCl_3$) δ 152.8, 147.0, 138.8, 134.8, 129.7, 129.6, 128.6, 128.3, 127.9, 127.3, 124.1, 120.9, 120.7, 118.3, 116.6, 99.5, 35.0, 31.4; HRMS (APCI): m/z calculated for $C_{52}H_{55}N_{10}$: 819.4611 $[M+H]^+$; found: 819.4608.

4,4'-(1,4-Bis(3,5-di-*tert*-butylphenyl)-1,4-dihydropyrrolo[3,2-*b*]pyrrole-2,5-diyl)bis(7-methyltetrazolo[1,5-*a*]quinoline) (6m):

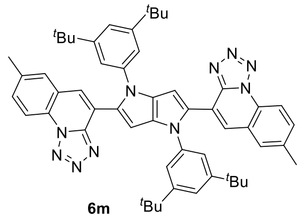

6m

Yellow solid (443 mg, 61%); m.p.: 307-308 °C (dec.); ^{1}H NMR (600 MHz, $CDCl_3$) δ 8.49 (d, *J* = 8.4 Hz, 2H), 7.61 (s, 2H), 7.52 (d, *J* = 8.4 Hz, 2H), 7.45 (t, *J* = 1.9 Hz, 2H), 7.31 (s, 4H), 7.16 (s, 2H), 7.09 (s, 2H), 2.48 (s, 6H), 1.27 (s, 36H); $^{13}C\{^{1}H\}$ NMR (151 MHz, $CDCl_3$) δ 152.7, 146.9, 138.8, 138.0, 134.6, 131.2, 129.6, 127.7, 127.4, 126.8, 124.1, 120.8, 120.5, 118.2,

116.4, 99.5, 35.0, 31.3, 21.4; HRMS (APCI): m/z calculated for $C_{54}H_{59}N_{10}$: 847.4924 $[M+H]^+$; found: 847.4923.

4,4'-(1,4-Bis(2-bromo-4-(*tert*-butyl)phenyl)-1,4-dihydropyrrolo[3,2-*b*]pyrrole-2,5-diyl)bis(7-hexyltetrazolo[1,5-*a*]quinoline) (6n):

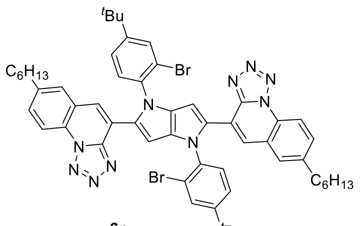

6n

Yellow solid (496 mg, 48%); m.p.: 291-292 °C (dec.); ^{1}H NMR (500 MHz, $CDCl_3$, mixture of atropisomers) δ 8.49 (s, 1H), 8.47 (s, 1H), 7.86 (d, *J* = 2.1 Hz, 1H), 7.76 (d, *J* = 2.1 Hz, 1H), 7.62 (d, *J* = 2.8 Hz, 2H), 7.57 (d, *J* = 8.2 Hz, 1H), 7.52 (s, 1H), 7.51 (s, 1H), 7.39 (dd, *J* = 8.3, 2.1 Hz, 1H), 7.28 (s, 1H), 7.17 (s, 1H), 7.13 (s, 1H), 6.98 (s, 1H), 6.97 (s, 2H), 2.71 (t, *J* = 6.9 Hz, 4H), 1.67-1.59 (m, 4H), 1.42 (s, 9H), 1.41 (s, 9H), 1.34-1.26 (m, 12H), 0.88 (t, *J* = 6.4 Hz, 6H); $^{13}C\{^1H\}$ NMR (126 MHz, $CDCl_3$, mixture of atropisomers) δ 153.7, 153.6, 146.6, 146.6, 143.2, 143.2, 136.5, 136.4, 135.0, 134.6, 131.2, 131.2, 131.1, 130.6, 130.6, 129.8, 129.7, 127.3, 127.2, 127.1, 127.0, 126.2, 126.1, 126.0, 125.6, 124.5, 122.2, 122.1, 118.4, 118.1, 116.4, 116.4, 100.8, 100.0, 35.7, 35.0, 35.0, 31.6, 31.2, 28.8, 28.8, 22.5, 14.0; HRMS (APCI): m/z calculated for $C_{56}H_{61}Br_2N_{10}$: 1031.3442 $[M+H]^+$; found: 1031.3451.

4,4'-(1,4-Bis(2-bromo-4-dodecylphenyl)-1,4-dihydropyrrolo[3,2-*b*]pyrrole-2,5-diyl)bis(7-methyltetrazolo[1,5-*a*]quinoline) (6o):

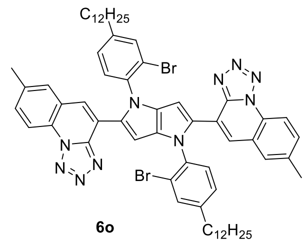

6o

Yellow solid (581 mg, 52%); m.p.: 271-272 °C; ^{1}H NMR (500 MHz, $CDCl_3$) δ 8.49 (d, *J* = 8.3 Hz, 2H), 7.69 (s, 1H), 7.60 (s, 3H), 7.56 (d, *J* = 7.6 Hz, 1H), 7.54 (s, 1H), 7.52 (s, 1H), 7.32 (d, *J* = 7.9 Hz, 1H), 7.24 (s, 2H), 7.23–7.16 (m, 2H), 7.03 (s, 2H), 2.74 (t, *J* = 7.4 Hz, 2H), 2.72 (t, *J* = 7.4 Hz, 2H), 2.49 (s, 6H), 1.77–1.66 (m, 4H), 1.46-1.36 (m, 8H), 1.35-1.21 (m, 28H), 0.90 (t, *J* = 6.7 Hz, 6H); $^{13}C\{^1H\}$ NMR (126 MHz, $CDCl_3$) δ 145.3, 138.1, 136.6, 135.1, 134.7,

133.9, 131.2, 130.0, 129.0, 128.0, 126.1, 125.4, 124.5, 118.3, 116.4, 100.8, 100.0, 35.3, 31.9, 31.2, 29.7, 29.7, 29.7, 29.6, 29.5, 29.4, 29.2, 22.7, 21.3, 14.1; HRMS (APCI): m/z calculated for $C_{62}H_{73}Br_2N_{10}$: 1115.4386 $[M+H]^+$; found: 1115.4421.

4,4'-(1,4-Bis(2-bromo-4-dodecylphenyl)-1,4-dihydropyrrolo[3,2-*b*]pyrrole-2,5-diyl)bis(7-octyltetrazolo[1,5-*a*]quinoline) (6p):

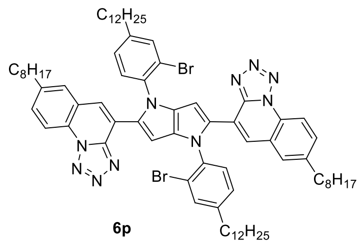

6p

Pale yellow solid (618 mg, 47%); m.p.: 245-246 °C (dec.); ^{1}H NMR (500 MHz, $CDCl_3$) δ 8.57-8.46 (m, 2H), 7.70-7.52 (m, 6H), 7.34-7.18 (m, 6H), 7.08-7.02 (m, 2H), 2.83-2.65 (m, 8H), 1.77-1.63 (m, 8H), 1.46-1.16 (m, 56H), 0.95-0.81 (m, 12H); $^{13}C\{^1H\}$ NMR (126 MHz, $CDCl_3$, mixture of atropisomers) δ 146.6 (2 signals), 145.4, 145.3, 143.2, 143.1, 139.1, 136.6, 135.1, 134.6, 133.9 (2 signals), 131.3, 131.2, 130.5 (2 signals), 130.0, 129.9, 129.1, 128.9, 127.4, 127.3, 127.0, 126.3, 125.6, 124.5, 124.4, 122.1, 122.0, 118.4, 118.2, 116.5, 116.4, 100.8, 100.0, 35.7, 35.3, 31.9, 31.8, 31.4, 31.3 (2 signals), 29.7 (3 signals), 29.6 (2 signals), 29.4 (2 signals), 29.3, 29.2, 22.7, 22.6, 14.1 (2 signals); HRMS (APCI): m/z calculated for $C_{76}H_{101}Br_2N_{10}$: 1311.6577 $[M+H]^+$; found: 1311.6583.

4,4'-(1,4-Bis(2-bromonaphthalen-1-yl)-1,4-dihydropyrrolo[3,2-*b*]pyrrole-2,5-diyl)bis(7-hexyltetrazolo[1,5-*a*]quinoline) (6q):

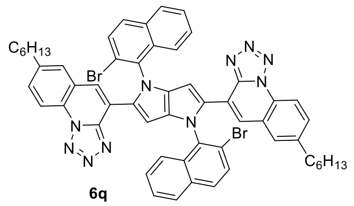

6q

Pale yellow solid (439 mg, 43%); m.p.: 227-228 °C (dec.); ^{1}H NMR (500 MHz, $CDCl_3$) δ 8.38 (dd, *J* = 8.5, 2.8 Hz, 2H), 7.98 (d, *J* = 8.8 Hz, 2H), 7.95 (d, *J* = 8.8 Hz, 2H), 7.90 (d, *J* = 8.8 Hz, 1H), 7.87 (d, *J* = 8.8 Hz, 1H), 7.64 (d, *J* = 8.4 Hz, 1H), 7.61 (d, *J* = 4.7 Hz, 2H), 7.59–7.51 (m, 3H), 7.48 (d, *J* = 7.7 Hz, 2H), 7.44 (d, *J* = 8.2 Hz, 2H), 6.96 (s, 1H), 7.00 (s, 1H), 6.93 (s, 1H), 6.90 (s, 1H), 2.66-2.58 (m, 4H), 1.62-1.51 (m, 4H), 1.33-1.22 (m, 12H), 0.83–

0.87 (m, 6H); $^{13}C\{^1H\}$ NMR (126 MHz, $CDCl_3$) δ 146.3, 143.1, 135.3, 135.0, 134.9, 133.4, 133.4, 132.8, 132.7, 132.5, 132.4, 130.5, 130.5, 130.1, 128.8, 128.8, 128.3, 127.3, 127.3, 127.0, 124.8, 124.7, 124.3, 123.3, 123.1, 122.4, 122.3, 118.1, 116.3, 100.2, 100.1, 35.6, 31.6, 31.3, 28.8, 22.6, 14.1; HRMS (APCI): m/z calculated for $C_{56}H_{49}Br_2N_{10}$: 1019.2508 $[M+H]^+$; found: 1019.2520.

4,4'-(1,4-Bis(10-bromophenanthren-9-yl)-1,4-dihydropyrrolo[3,2-*b*]pyrrole-2,5-diyl)bis(7-octyltetrazolo[1,5-*a*]quinoline) (6r):

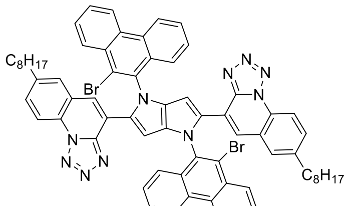

6r

Brown solid (424 mg, 36%); m.p.: 231-232 ˚C (dec.); ^1H NMR (500 MHz, $CDCl_3$) δ 9.23 (s, 2H), 8.91 (s, 2H), 8.63 (d, *J* = 8.2 Hz, 4H), 8.50 (d, *J* = 8.6 Hz, 2H), 8.39 (d, *J* = 8.0 Hz, 2H), 7.87 (d, *J* = 8.2 Hz, 2H), 7.83 (s, 2H), 7.89-7.63 (m, 8H), 7.49 (t, *J* = 7.5 Hz, 2H), 2.75 (t, *J* = 7.7 Hz, 4H), 1.74-1.62 (m, 4H), 1.40-1.22 (m, 20H), 0.88 (t, *J* = 6.7 Hz, 6H); $^{13}C\{^1H\}$ NMR (126 MHz, $CDCl_3$) δ 160.2, 146.7, 146.3, 143.7, 133.5, 132.0, 130.6, 129.8, 129.6, 129.3, 129.3, 128.4, 127.9, 127.5, 127.2, 127.1, 126.4, 124.6, 123.6, 122.8, 122.6, 120.5, 116.6, 108.6, 35.6, 31.8, 31.1, 29.4, 29.2, 29.2, 22.7, 14.1; HRMS (APCI): m/z calculated for $C_{68}H_{61}Br_2N_{10}$: 1175.3442 $[M+H]^+$; found: 1175.3443.

Typical procedure for the synthesis of 7,19-didodecyl-2,14-dihexyldibenzo[*b*,*h*]benzo[5',6']quino[2'',3'':7',8']indolizino[3',2':4,5]pyrrolo[2,1-*f*]-1,6-naphthyridine (10a):

The Schenk flask was charged with chloroquinoline-containing pyrrolopyrrole **4h** (44.0 mg, 0.04 mmol, 1 *eq.*), Ph_3P (10.5 mg, 0.04 mmol, 1 *eq.*), Cs_2CO_3 (58.5 mg, 0.18 mmol, 4.5 *eq.*) and $Pd(OAc)_2$ (3.6 mg, 40 mol%). Then, dry *m*-xylene (3 mL) was added, resulting mixture was degassed 3 times (by evacuation and refilling with Argon) and stirred at 160 °C overnight. After cooling to around 80 °C, toluene (10 mL) was added and resulting suspension was passed through a pad of Celite. Next, the product was washed from the Celite pad with boiling toluene, all filtrates were concentrated to *ca*. 2 mL and 36.5 mg (90%) of **10a** as orange solid was filtered off. The dried product **10a** obtained was pure enough for all analytical purposes.

The procedure for the synthesis of compounds **10b** and **10c** is similar to compound **10a**.

7,19-Didodecyl-2,14-dihexyldibenzo[*b*,*h*]benzo[5',6']quino[2'',3'':7',8']indolizino[3',2':4,5]pyrrolo[2,1-*f*]-1,6-naphthyridine (10a):

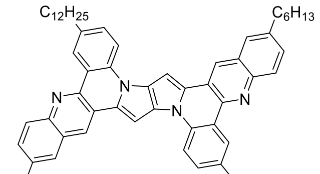

10a

Orange solid (36.5 mg, 90%); m.p.: 305-306 °C (dec.); ^{1}H NMR (500 MHz, Benzene-d_6) δ 9.35 (d, *J* = 7.2 Hz, 2H), 8.30 (dd, *J* = 8.5, 3.9 Hz, 2H), 8.05 (d, *J* = 8.4 Hz, 2H), 7.81-7.78 (m, 2H), 7.47-7.43 (m, 4H), 7.31-7.29 (m, 2H), 6.97 (s, 1H), 6.96 (d, *J* = 11.0 Hz, 1H), 2.79 (t, *J* = 7.9 Hz, 4H), 2.71 (t, *J* = 7.8 Hz, 4H), 1.84-1.79 (m, 4H), 1.74-1.71 (m, 4H), 1.53-1.24 (m, 48H), 0.97 (t, *J* = 6.8 Hz, 6H), 0.91 (t, *J* = 6.8 Hz, 6H); $^{13}C\{^{1}H\}$ NMR (126 MHz, tetrachloroethane-[D_2]) δ 145.7, 143.9, 141.0, 137.7, 134.6, 130.6, 130.3, 129.2, 127.8, 126.8, 126.4, 125.7, 125.1, 122.2, 120.4, 114.8, 99.7, 89.3, 36.0, 35.7, 31.9, 31.8, 31.4, 30.8, 29.7, 29.6,

29.6, 29.3, 29.1, 22.6, 14.0, 13.9; HRMS (APCI): m/z calculated for $C_{72}H_{93}N_4$: 1013.7400 $[M+H]^+$; found: 1013.7426.

7,19-Didodecyl-3,15-bis(methylthio)dibenzo[*b*,*h*]benzo[5',6']quino[2'',3'':7',8']indolizino[3',2':4,5]pyrrolo[2,1-*f*]-1,6-naphthyridine (10b):

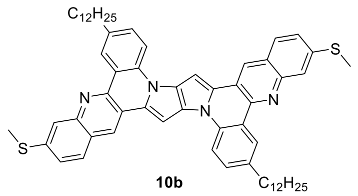

Red solid (14.7 mg, 39%); m.p.: 323-324 °C (dec.); ^{1}H NMR (500 MHz, tetrachloroethane-[D_2]) δ 8.76 (s, 2H), 8.06-8.20 (br. s, 2H), 7.71-7.69 (m, 4H), 7.55-7.48 (br. s, 2H), 7.44 (d, *J* = 8.2 Hz, 2H), 7.29 (d, *J* = 8.2 Hz, 2H), 6.85-6.99 (br. s, 2H), 2.83 (t, *J* = 7.9 Hz, 4H), 2.64 (s, 6H), 1.82-1.85 (m, 4H), 1.50-1.55 (m, 4H), 1.43-1.49 (m, 4H), 1.26-1.40 (m, 28H), 0.87 (t, *J* = 6.8 Hz, 6H); HRMS (APCI): m/z calculated for $C_{62}H_{73}N_4S_2$: 937.5277 $[M+H]^+$; found: 937.5278.

2,16-Dioctylbenzo[*b*]naphtho[1,2-*h*]naphtho[1'',2'':5',6']quino[2'',3'':7',8']indolizino[3',2':4,5]pyrrolo[2,1-*f*]-1,6-naphthyridine (10c):

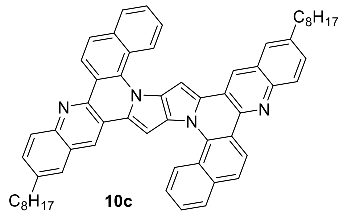

Orange solid (11.7 mg, 35%); m.p.: 295-296 °C (dec.); ^{1}H NMR (500 MHz, $CDCl_3$) δ 9.25 (d, *J* = 7.1 Hz, 2H), 9.20 (d, *J* = 8.6 Hz, 2H), 8.52 (s, 2H), 8.07 (d, *J* = 8.6 Hz, 2H), 8.05 (dd, *J* = 8.6, 2.4 Hz, 2H), 7.90 (d, *J* = 8.6 Hz, 2H), 7.75–7.72 (m, 4H), 7.62 (s, 2H), 7.52 (dd, *J* = 8.6, 1.9 Hz, 2H), 7.21 (s, 2H), 2.80 (t, *J* = 7.8 Hz, 4H), 1.76–1.73 (m, 4H), 1.44–1.24 (m, 20H), 0.88 (t, *J* = 6.7 Hz, 6H); $^{13}C\{^1H\}$ NMR (126 MHz, $CDCl_3$) δ 146.2, 144.4, 141.3, 135.6, 133.1, 131.9, 131.8, 130.8, 129.3, 128.5, 127.8, 127.4, 126.2, 125.4, 124.8, 124.6, 124.5, 123.4, 122.8, 121.1, 120.8, 95.5, 36.0, 31.9, 31.1, 29.5, 29.4, 29.3, 22.7, 14.1; HRMS (APCI): m/z calculated for $C_{60}H_{57}N_4$: 833.4583 $[M+H]^+$; found: 833.4585.

Typical procedure for the synthesis of 10,24-didodecyl-7,21-dimethyldibenzo[*c*,*f*]benzo[5',6']tetrazolo[1''',5''':1'',2'']quino[4'',3'':7',8']indolizino[3',2':4,5]pyrrolo[2,1-*a*]tetrazolo[1,5-*h*]-2,7-naphthyridine (11a):

The Schenk flask was charged with di-brominated tetrazoloquinoline-containing pyrrolopyrrole **6o** (90.0 mg, 0.08 mmol, 1 *eq.*), Ph_3P (21.0 mg, 0.08 mmol, 1 *eq.*), Cs_2CO_3 (117.0 mg, 0.36 mmol, 4.5 *eq.*) and $Pd(OAc)_2$ (7.2 mg, 40 mol%). Then, dry *m*-xylene (6 mL) was added, resulting mixture was degassed 3 times (by evacuation and refilling with Argon) and stirred at 160 °C for 20 h. After cooling to around 80 °C, toluene (10 mL) was added and resulting suspension was passed through a pad of Celite. Next, the product was washed from the Celite pad with boiling toluene, all filtrates were concentrated to *ca*. 1 mL. Next, 1 mL of chloroform was added and the flask with reaction mixture was moved to the fridge. Upon 8 h 35.0 mg (45%) of compound **11a** was filtered off as dark purple fine crystals. The dried product **11a** obtained was pure enough for all analytical purposes.

The procedure for the synthesis of compounds **11b-11d** is similar to compound **11a**, except that compounds **11c** and **11d** were purified by column chromatography (SiO_2, EtOAc:hexanes, 20:80, then 25:75).

10,24-Didodecyl-7,21-dimethyldibenzo[*c*,*f*]benzo[5',6']tetrazolo[1''',5''':1'',2'']quino[4'',3'':7',8']indolizino[3',2':4,5]pyrrolo[2,1-*a*]tetrazolo[1,5-*h*]-2,7-naphthyridine (11a):

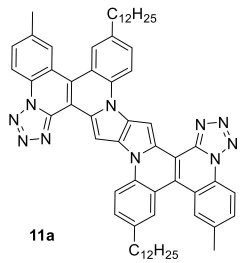

11a

Dark purple solid (35.0 mg, 45%); m.p.: 296-297 °C (dec.); ^{1}H NMR (600 MHz, $CDCl_3$) δ 8.40 (s, 2H), 8.33 (d, *J* = 8.1 Hz, 2H), 8.08 (s, 2H), 7.89 (s, 2H), 7.76 (d, *J* = 8.1 Hz, 2H), 7.43 (d, *J* = 8.2 Hz, 2H), 7.23 (d, *J* = 8.2 Hz, 2H), 2.68 (t, *J* = 7.9 Hz, 4H), 2.58 (s, 6H), 1.75-1.70 (m, 4H), 1.44-1.40 (m, 4H), 1.39-1.24 (m, 32H), 0.88 (t, *J* = 6.9 Hz, 6H); ^{13}C{^{1}H} NMR

(126 MHz, $CDCl_3$) δ 144.5, 137.5, 137.1, 133.2, 130.7, 129.9, 128.4, 127.7, 127.2, 127.0, 124.0, 122.6, 121.8, 117.0, 115.7, 111.7, 92.7, 35.6, 31.9, 31.4, 31.4, 30.2, 29.8, 29.7, 29.7, 29.4, 29.4, 22.7, 21.9, 14.1; HRMS (APCI): m/z calculated for $C_{62}H_{71}N_{10}$: 955.5863 $[M+H]^+$; found: 955.5852.

10,24-Didodecyl-7,21-dioctyldibenzo[*c*,*f*]benzo[5',6']tetrazolo[1''',5''':1'',2'']quino[4'',3'':7',8']indolizino[3',2':4,5]pyrrolo[2,1-*a*]tetrazolo[1,5-*h*]-2,7-naphthyridine (11b):

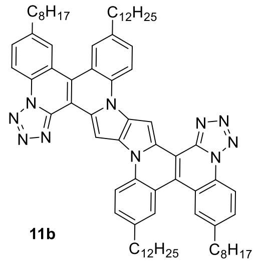

11b

Dark purple solid (43.3 mg, 47%); m.p.: 312-313 °C (dec.); ^1H NMR (500 MHz, $CDCl_3$) δ 8.42 (s, 2H), 8.37 (d, *J* = 8.2 Hz, 2H), 8.11 (s, 2H), 7.98 (s, 2H), 7.87 (d, *J* = 8.2 Hz, 2H), 7.45 (dd, *J* = 8.3, 1.5 Hz, 2H), 7.31 (d, *J* = 8.2 Hz, 2H), 2.80 (t, *J* = 7.9 Hz, 4H), 2.71 (t, *J* = 7.9 Hz, 4H), 1.83-1.78 (m, 4H), 1.76–1.70 (m, 4H), 1.52-1.25 (m, 56H), 0.91 (t, *J* = 7.2 Hz, 6H), 0.86 (t, *J* = 7.2 Hz, 6H); $^{13}C\{^1H\}$ NMR (126 MHz, $CDCl_3$) δ 144.3, 142.3, 136.9, 133.0, 130.6, 129.1, 128.0, 127.8, 127.5, 127.2, 126.8, 122.4, 121.6, 118.0, 116.8, 115.5, 111.4, 92.6, 36.2, 35.7, 32.0, 31.9, 31.7, 31.5, 29.8, 29.8, 29.8, 29.7, 29.7, 29.6, 29.5, 29.4, 29.4, 22.7, 22.7, 14.1, 14.2; HRMS (APCI): m/z calculated for $C_{76}H_{98}N_{10}$: 1150.7976 $[M]^+$; found: 1150.7977.

7,23-Dihexylbenzo[*c*]naphtho[2,1-*f*]naphtho[1'',2'':5',6']tetrazolo[1''',5''':1'',2'']quino[4'',3'':7',8']indolizino[3',2':4,5]pyrrolo[1,2-*h*]tetrazolo[5,1-*a*]-2,7-naphthyridine (11c):

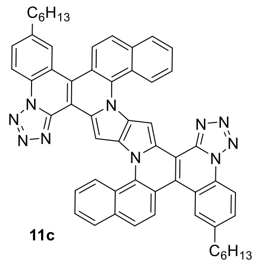

11c

Purple solid (26.8 mg, 39%); m.p.: 301-302 ˚C (dec.); ^{1}H NMR (500 MHz, $CDCl_3$) δ 8.97 (d, *J* = 8.7 Hz, 2H), 8.95 (s, 2H), 8.58 (d, *J* = 8.4 Hz, 2H), 8.40 (d, *J* = 8.9 Hz, 2H), 7.71 (dd, *J* = 8.2, 1.6 Hz, 2H), 7.63 (d, *J* = 8.0 Hz, 2H), 7.59 (d, *J* = 8.8 Hz, 2H), 7.44-7.39 (br. s, 2H), 7.35 (t, *J* = 7.7 Hz, 2H), 7.29 (t, *J* = 7.7 Hz, 2H), 3.14 (t, *J* = 7.8 Hz, 4H), 2.09-2.04 (m, 4H), 1.70-1.64 (m, 4H), 1.58-1.55 (m, 2H), 1.53-1.51 (m, 2H), 1.49-1.42 (m, 4H), 0.98 (t, *J* = 7.2 Hz, 6H); ^{13}C{^{1}H} NMR (151 MHz, CD_2Cl_2) δ 149.1, 144.6, 142.9, 133.1, 132.1, 130.1, 128.9, 128.6, 128.5, 127.9, 127.7, 124.8, 124.4, 124.0, 123.8, 123.5, 122.7, 120.3, 117.6, 113.4, 111.7, 95.5, 36.6, 32.2, 31.9, 29.6, 23.2, 14.3; HRMS (APCI): m/z calculated for $C_{56}H_{47}N_{10}$: 859.3985 $[M+H]^+$; found: 859.3992.

7-Octyl-19-(7-octyltetrazolo[1,5-*a*]quinolin-4-yl)-20-(9-phenanthryl)-20*H*-benzo[*c*]phenanthro[9,10-*f*]pyrrolo[2',3':4,5]pyrrolo[1,2-*h*]tetrazolo[5,1-*a*]-2,7-naphthyridine (11d):

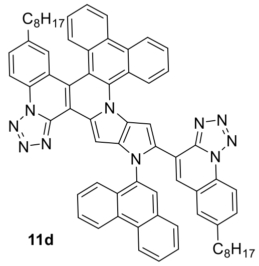

11d

Purple solid (21.2 mg, 26%); m.p.: 286-287 ˚C (dec.); ^{1}H NMR (600 MHz, $CDCl_3$) δ 9.40-9.38 (m, 1H); 8.87-7.29 (complex multiplets, 24H), 6.83 (d, *J* = 8.4 Hz, 1H), 2.53-2.48 (m, 4H), 1.50-1.43 (m, 4H), 1.31-1.17 (m, 20H), 0.88-0.83 (m, 6H); ^{13}C{^{1}H} NMR (126 MHz, CD_2Cl_2) δ 145.3, 143.2, 141.6, 140.0, 139.8, 135.6, 134.6, 131.9, 131.6, 131.3, 130.8, 130.5, 130.4, 130.4, 130.2, 129.8, 129.5, 129.4, 129.1, 129.0, 127.9, 127.8, 127.7, 127.6, 127.5, 127.4, 127.4, 127.3, 127.2, 127.1, 126.8, 126.7, 126.3, 126.2, 126.2, 125.6, 124.7, 124.5, 124.0, 124.0, 123.8, 123.5, 123.3, 122.9, 122.1, 117.9, 116.7, 116.0, 112.5, 105.3, 105.0, 91.4, 91.2, 35.8, 35.4, 31.8, 31.8, 31.2, 29.7, 29.3, 29.3, 29.1, 29.1, 29.1, 22.6, 22.6, 13.9, 13.8; HRMS (APCI): m/z calculated for $C_{68}H_{61}N_{10}$: 1017.5081 $[M+H]^+$; found: 1017.5085.

^{1}H NMR (600 MHz, 300K, $CDCl_3$) of **4a**

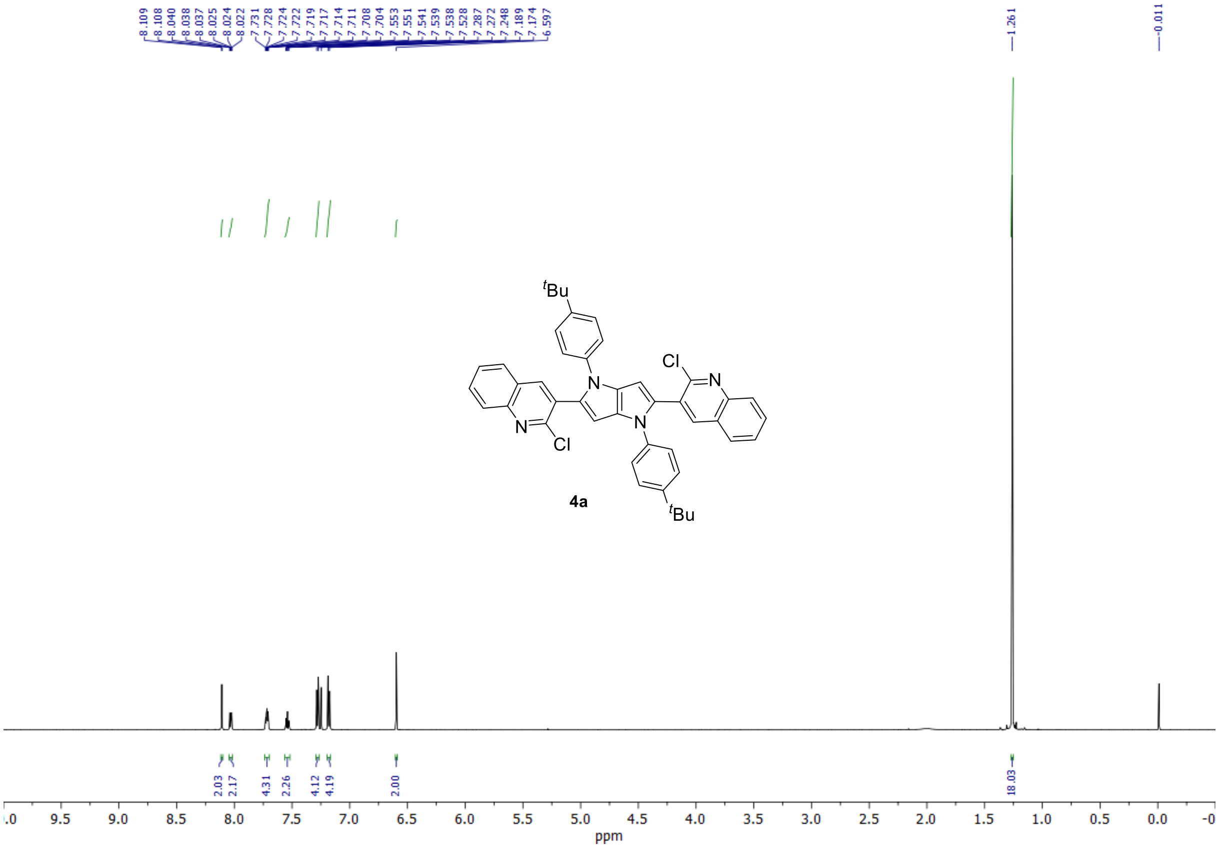

$^{13}C\{^{1}H\}$ NMR (151 MHz, 300K, $CDCl_3$) of **4a**

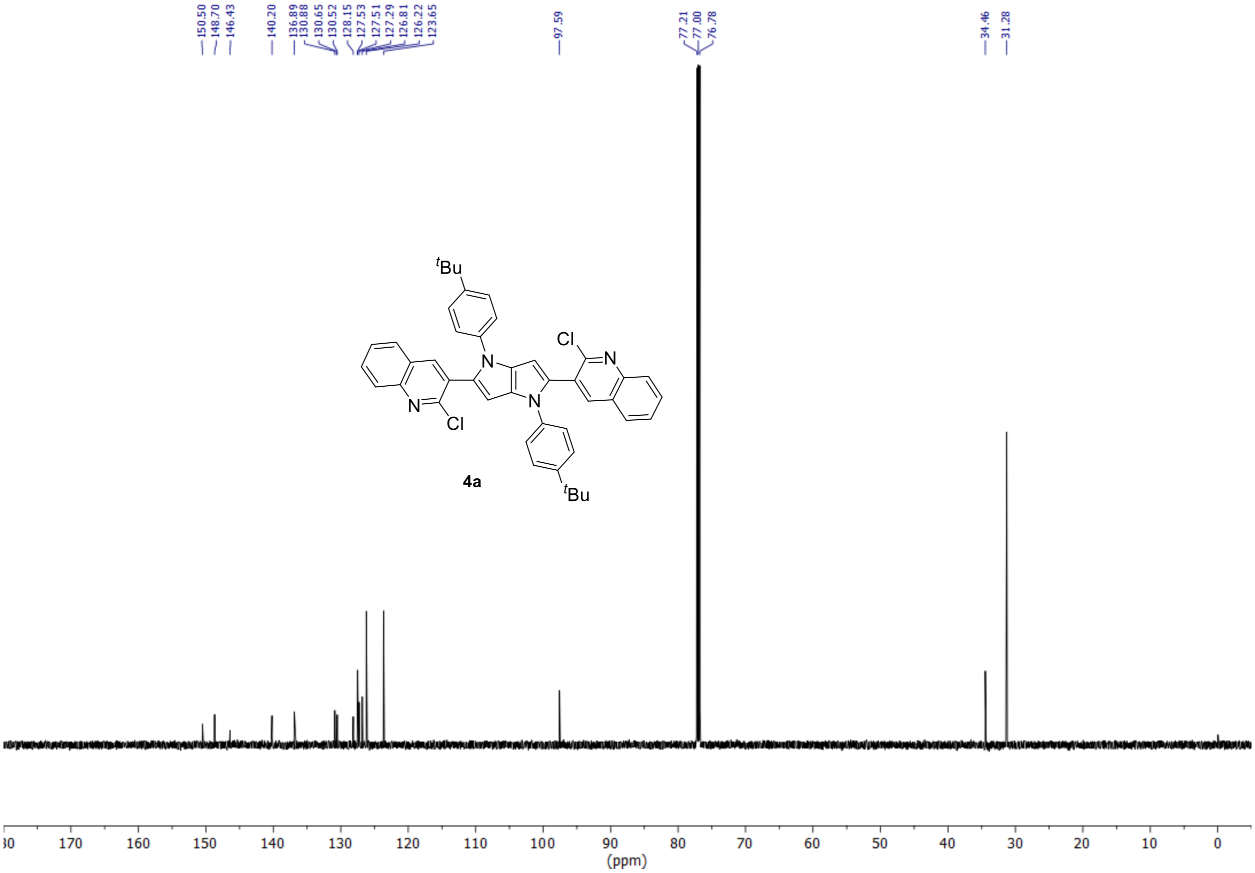

Report of HRMS of **4a**:

Single Mass Analysis
Tolerance = 5.0 PPM / DBE: min = -1.5, max = 500.0
Element prediction: Off
Number of isotope peaks used for i-FIT = 3
Monoisotopic Mass, Even Electron Ions
77 formula(e) evaluated with 1 results within limits (up to 50 closest results for each mass)

Elements Used:
C: 0-200 H: 0-200 N: 2-4 Cl: 0-2

Mass	Calc. Mass	mDa	PPM	DBE	Formula	i-FIT	i-FIT Norm	Fit Conf %	C	H	N	Cl
693.2549	693.2552	-0.3	-0.4	26.5	C44 H39 N4 Cl2	709.6	n/a	n/a	44	39	4	2

^{1}H NMR (600 MHz, 300K, $CDCl_3$) of **4b**

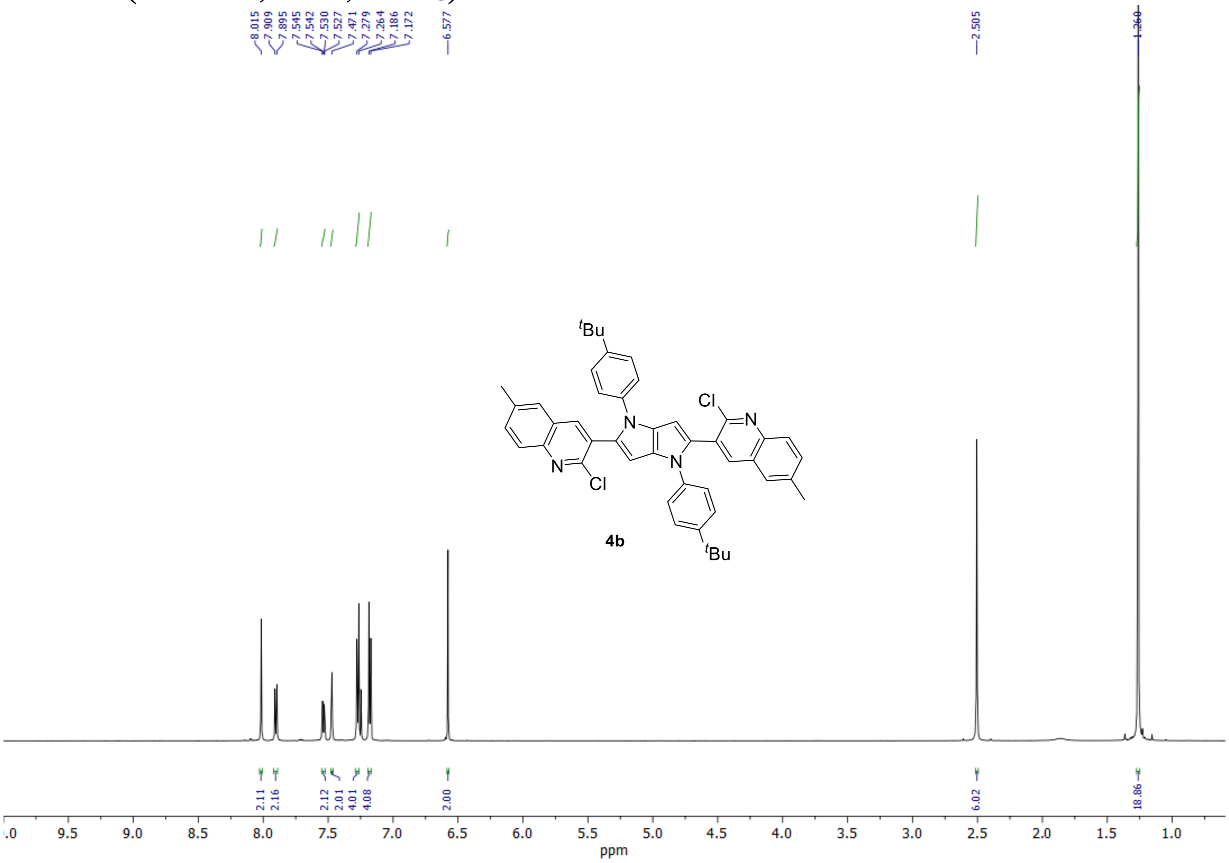

$^{13}C\{^{1}H\}$ NMR (151 MHz, 300K, $CDCl_3$) of **4b**

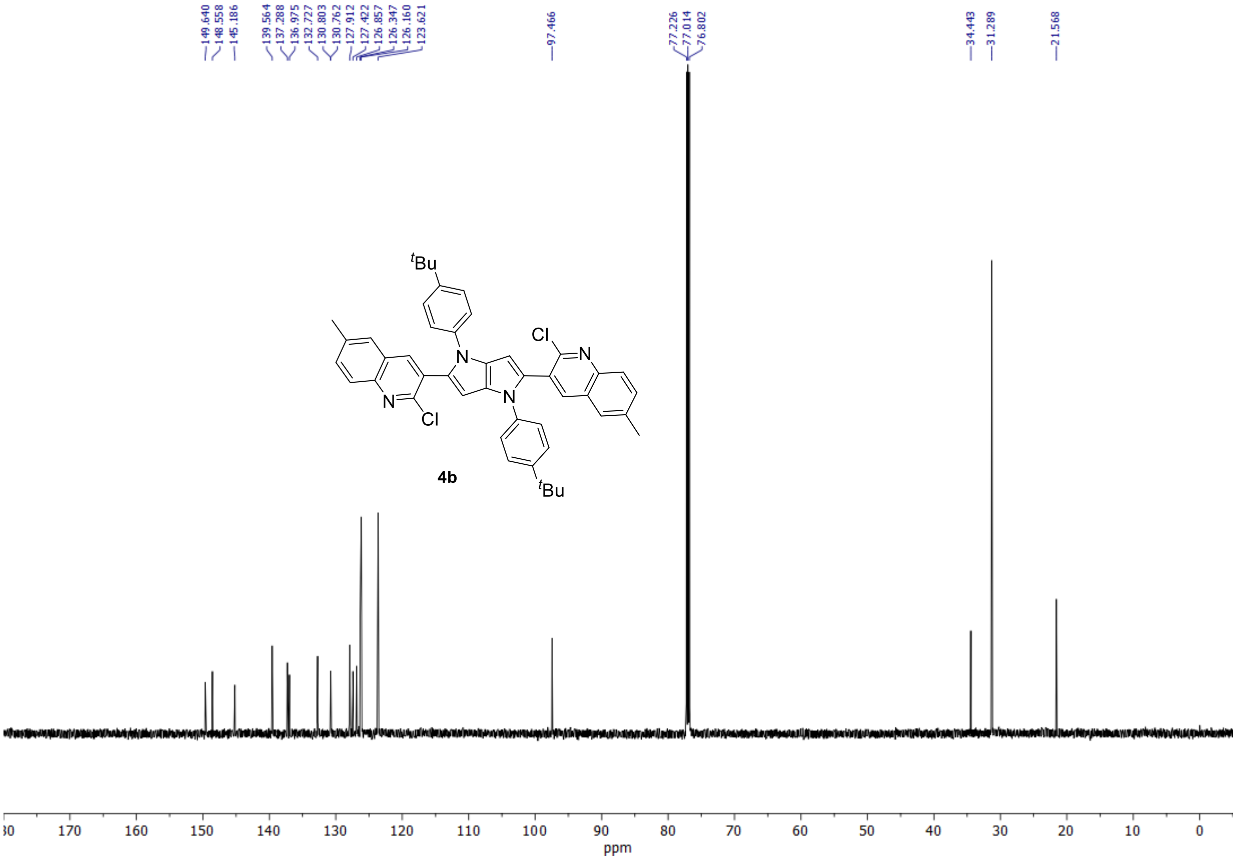

Report of HRMS of **4b**:

Single Mass Analysis
Tolerance = 5.0 PPM / DBE: min = -1.5, max = 500.0
Element prediction: Off
Number of isotope peaks used for i-FIT = 3
Monoisotopic Mass, Even Electron Ions
78 formula(e) evaluated with 1 results within limits (up to 50 closest results for each mass)
Elements Used:
C: 0-200 H: 0-200 N: 2-4 Cl: 0-2

Mass	Calc. Mass	mDa	PPM	DBE	Formula	i-FIT	i-FIT Norm	Fit Conf %	C	H	N	Cl
721.2862	721.2865	-0.3	-0.4	26.5	C46 H43 N4 Cl2	741.8	n/a	n/a	46	43	4	2

^{1}H NMR (600 MHz, 300K, $CDCl_3$) of **4c**

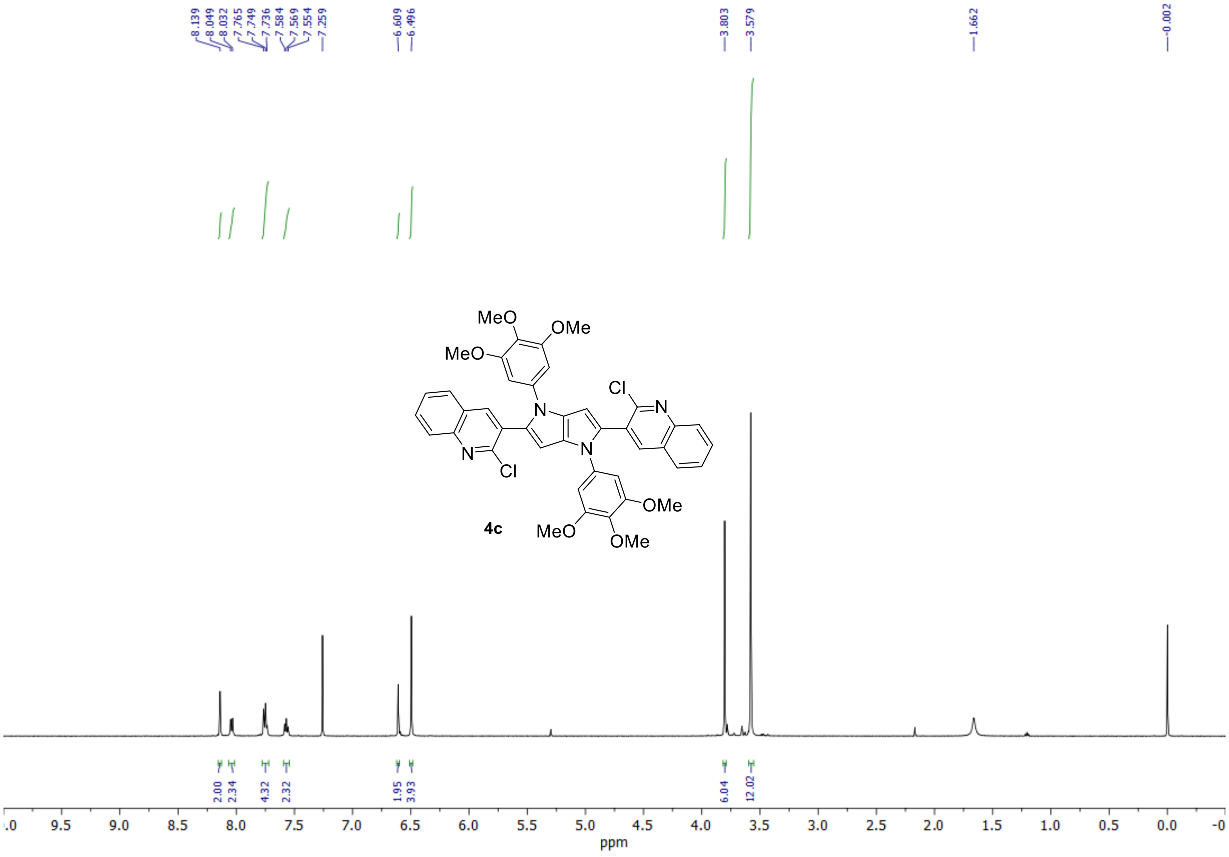

^{13}C{^{1}H} NMR (126 MHz, 300K, $CDCl_3$) of **4c**

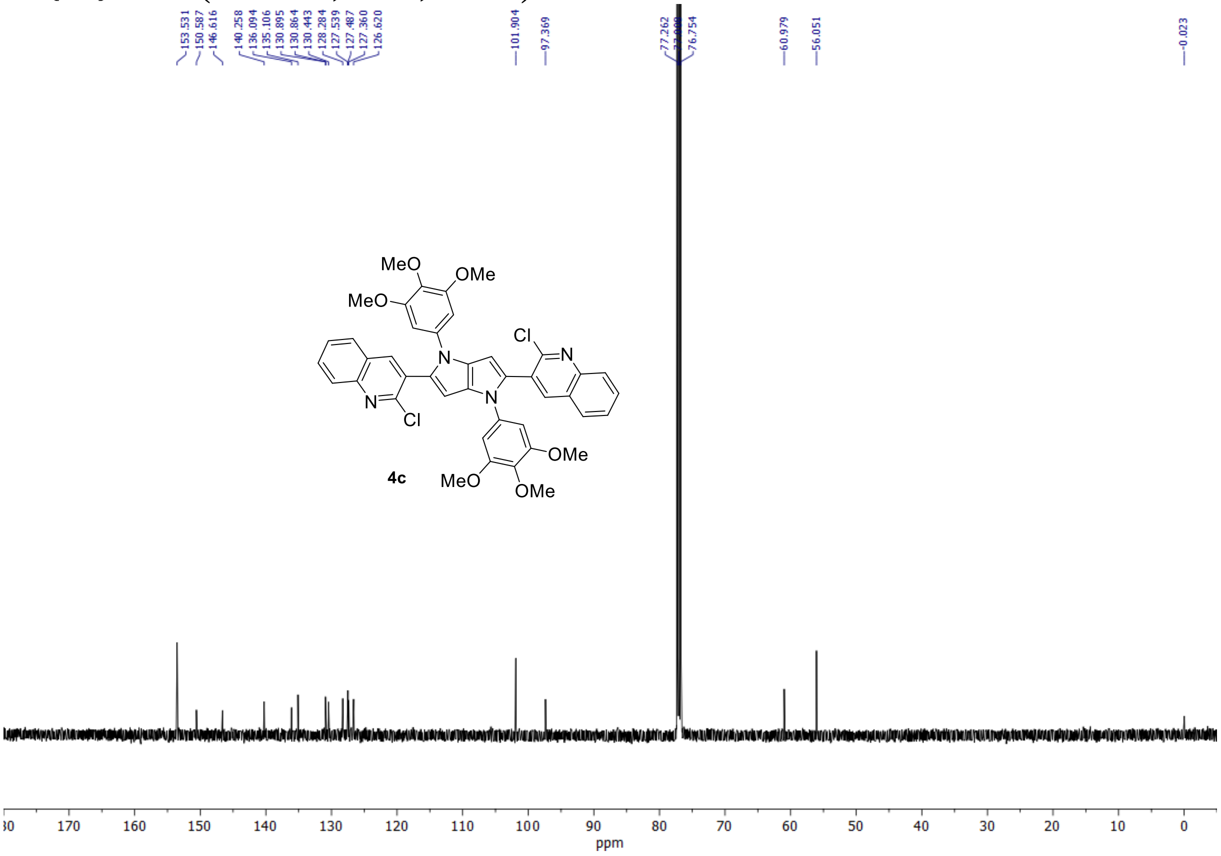

Report of HRMS of **4c**:

Single Mass Analysis
Tolerance = 10.0 PPM / DBE: min = -1.5, max = 500.0
Element prediction: Off
Number of isotope peaks used for i-FIT = 3
Monoisotopic Mass, Even Electron Ions
48 formula(e) evaluated with 1 results within limits (up to 50 closest results for each mass)
Elements Used:
C: 0-200 H: 0-200 N: 3-4 O: 4-6 Cl: 2-2

Mass	Calc. Mass	mDa	PPM	DBE	Formula	i-FIT	i-FIT Norm	Fit Conf %	C	H	N	O	Cl
761.1932	761.1934	-0.2	-0.3	26.5	C42 H35 N4 O6 Cl2	481.5	n/a	n/a	42	35	4	6	2

^{1}H NMR (500 MHz, 300K, $CDCl_3$) of **4d**

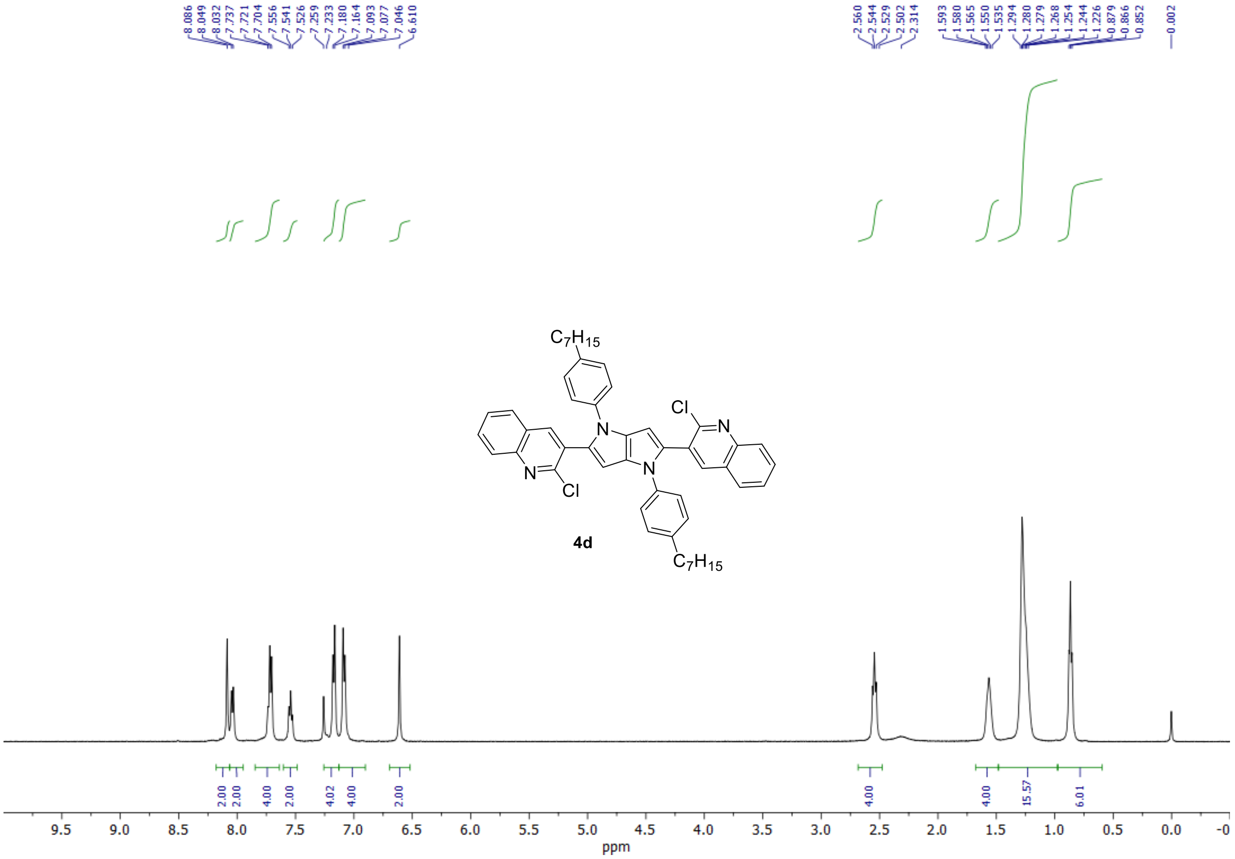

$^{13}C\{^{1}H\}$ NMR (126 MHz, 300K, $CDCl_3$) of **4d**

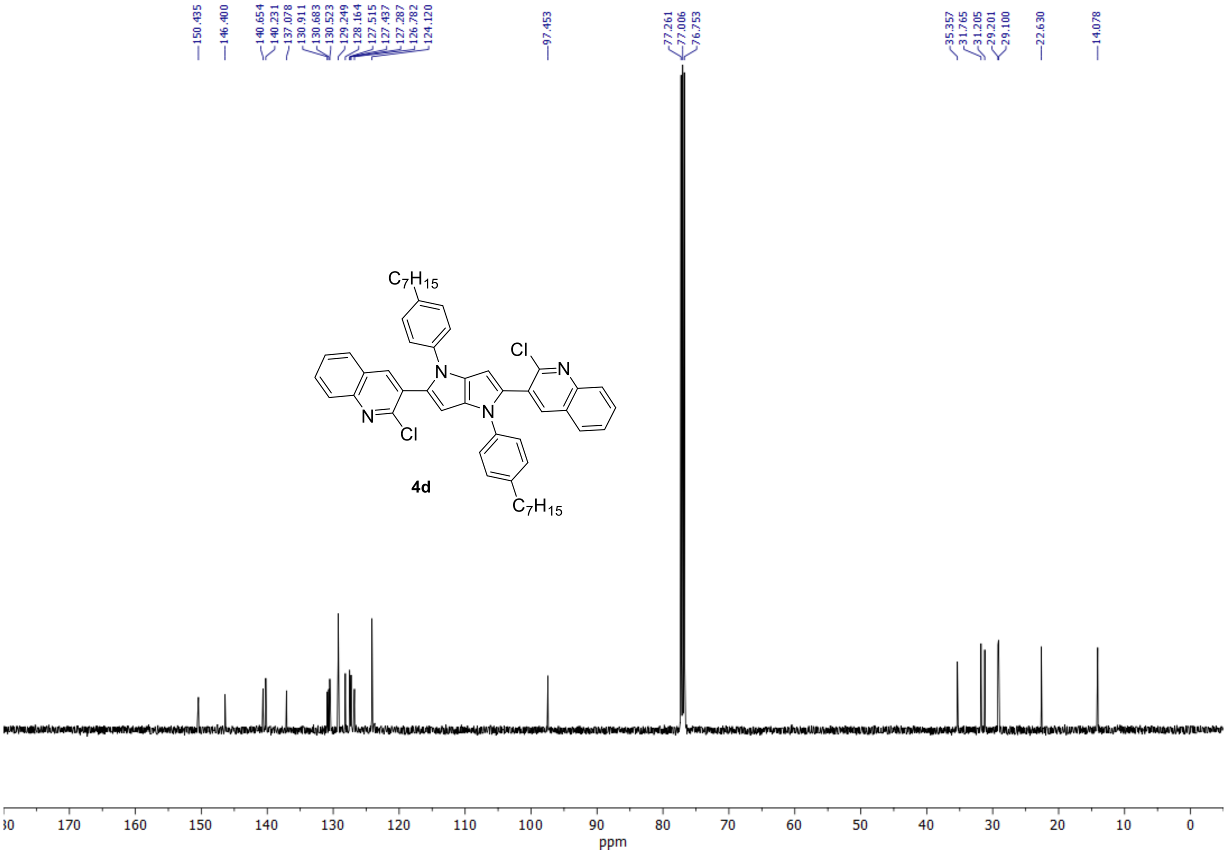

Report of HRMS of **4d**:

Single Mass Analysis
Tolerance = 5.0 mDa / DBE: min = -1.5, max = 500.0
Element prediction: Off
Number of isotope peaks used for i-FIT = 3
Monoisotopic Mass, Even Electron Ions
126 formula(e) evaluated with 2 results within limits (all results (up to 1000) for each mass)
Elements Used:
C: 0-100 H: 0-100 N: 0-4 Cl: 0-2

Mass	Calc. Mass	mDa	PPM	DBE	Formula	i-FIT	i-FIT Norm	Fit Conf %	C	H	N	Cl
777.3497	777.3491	0.6	0.8	26.5	C50 H51 N4 Cl2	533.5	0.000	100.00	50	51	4	2
	777.3521	-2.4	-3.1	39.5	C61 H45	550.3	16.747	0.00	61	45		

^{1}H NMR (500 MHz, 300K, $CDCl_3$) of **4e**

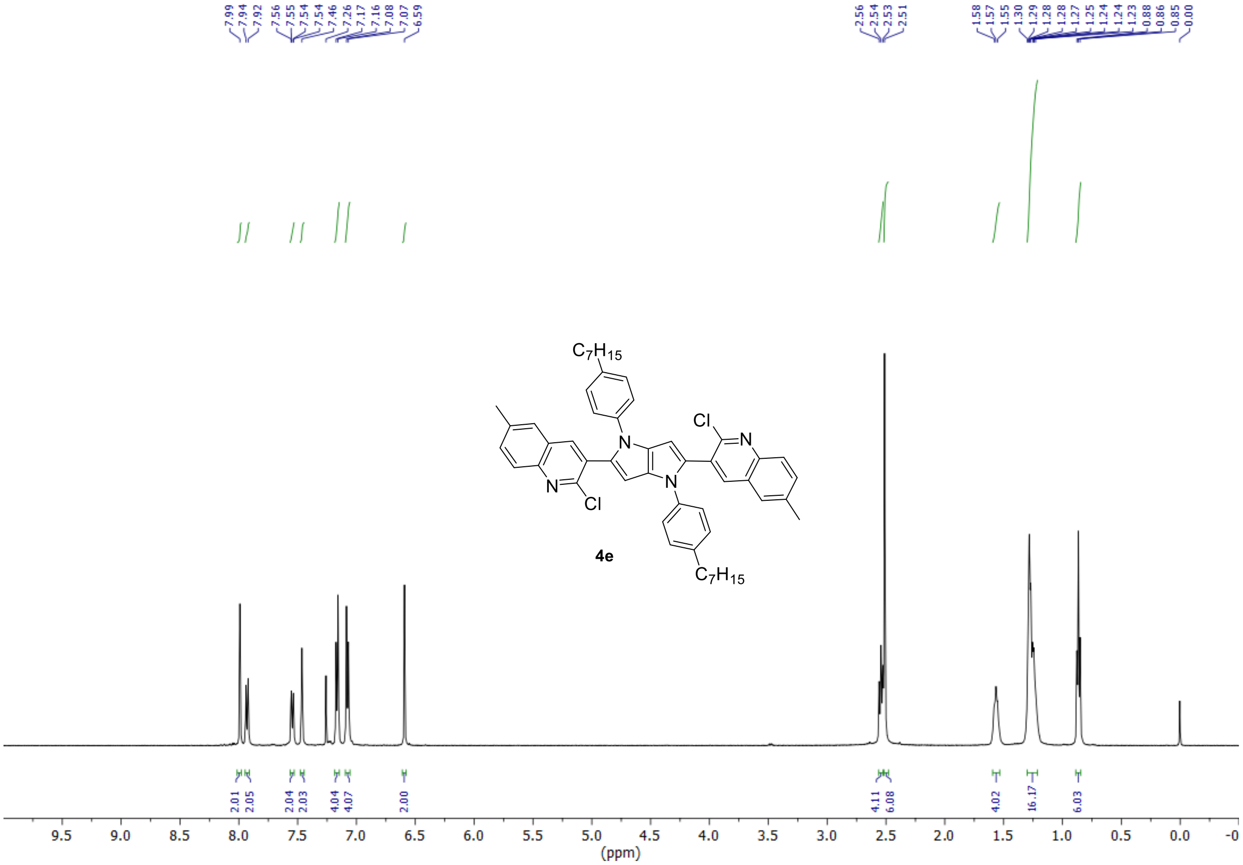

$^{13}C\{^{1}H\}$ NMR (126 MHz, 300K, $CDCl_3$) of **4e**

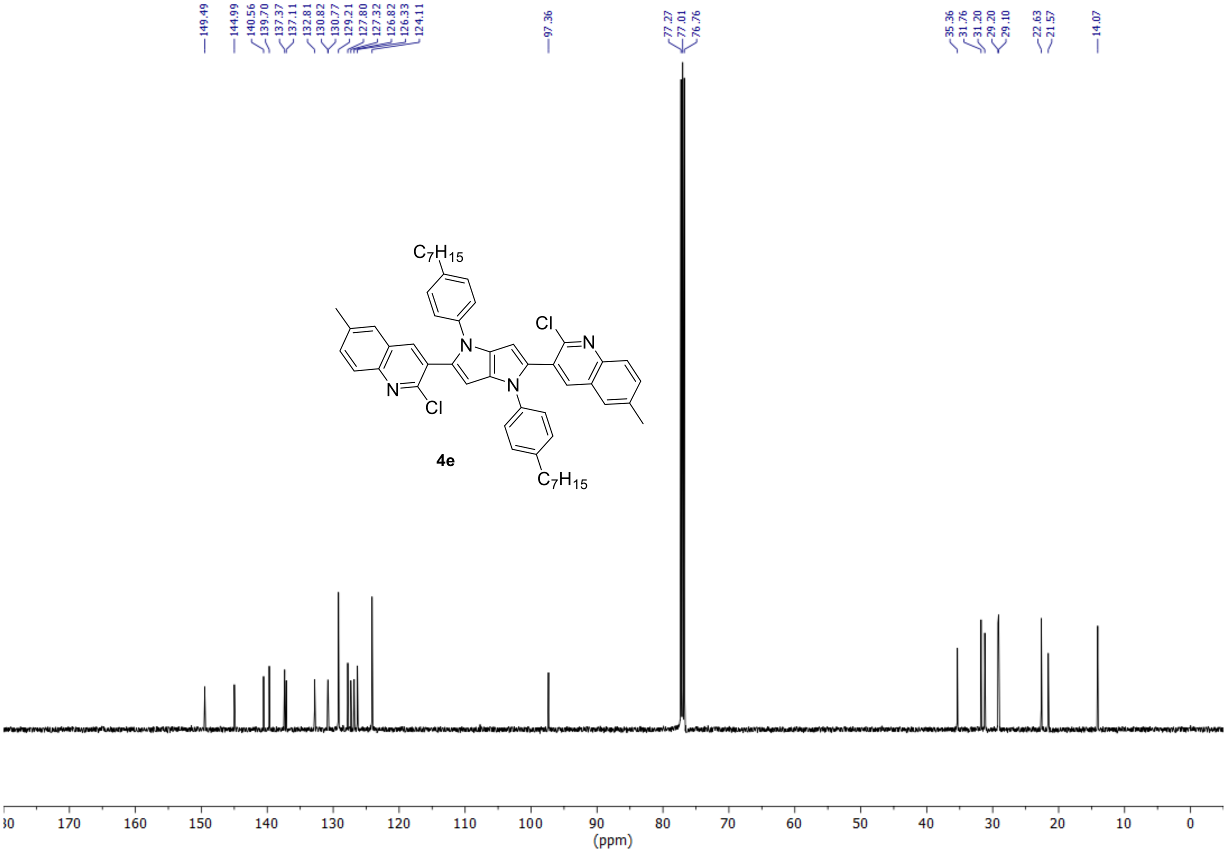

Report of HRMS of **4e**:

Single Mass Analysis
Tolerance = 5.0 mDa / DBE: min = -1.5, max = 500.0
Element prediction: Off
Number of isotope peaks used for i-FIT = 3
Monoisotopic Mass, Even Electron Ions
127 formula(e) evaluated with 2 results within limits (all results (up to 1000) for each mass)
Elements Used:
C: 0-100 H: 0-100 N: 0-4 Cl: 0-2

Mass	Calc. Mass	mDa	PPM	DBE	Formula	i-FIT	i-FIT Norm	Fit Conf %	C	H	N	Cl
805.3807	805.3804	0.3	0.4	26.5	C52 H55 N4 Cl2	586.6	0.000	100.00	52	55	4	2
	805.3834	-2.7	-3.4	39.5	C63 H49	603.2	16.600	0.00	63	49		

^{1}H NMR (500 MHz, 300K, $CDCl_3$) of **4f**

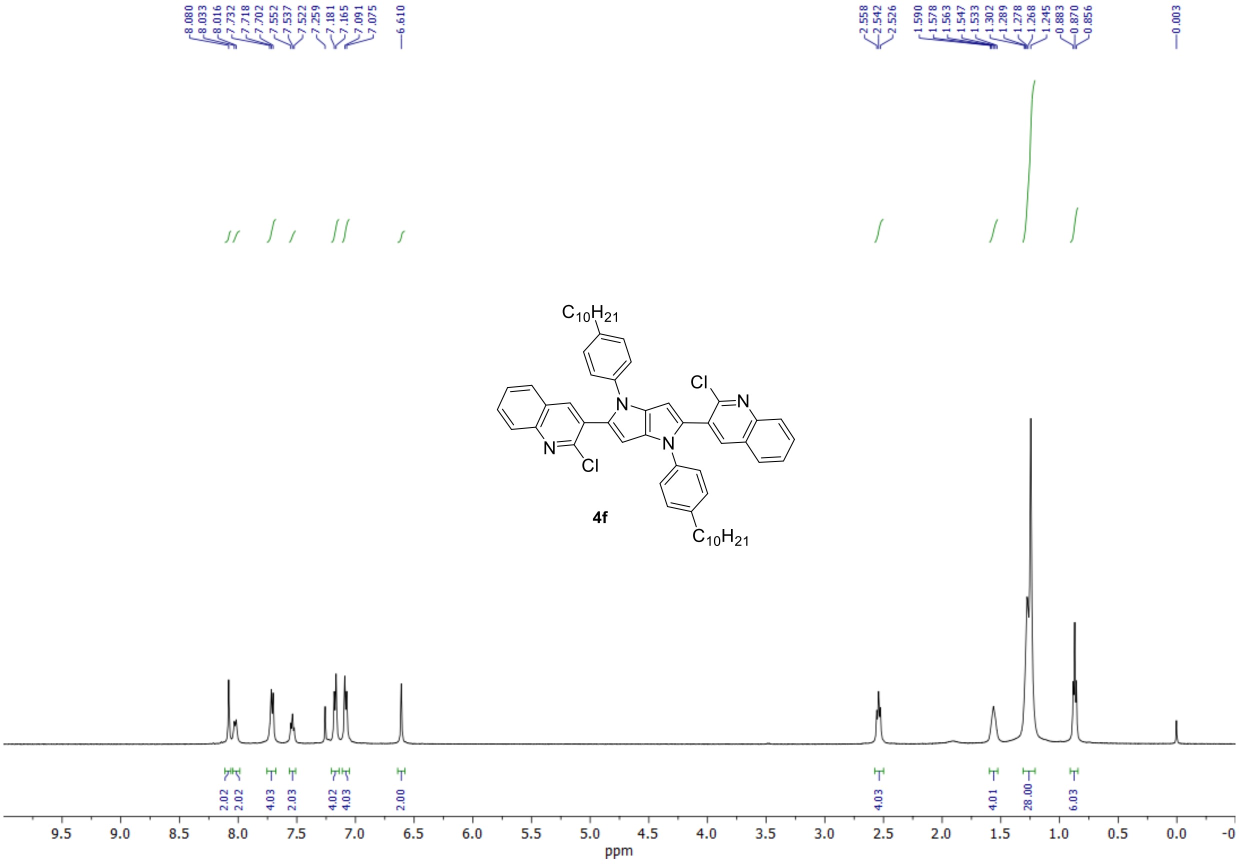

$^{13}C\{^1H\}$ NMR (126 MHz, 300K, $CDCl_3$) of **4f**

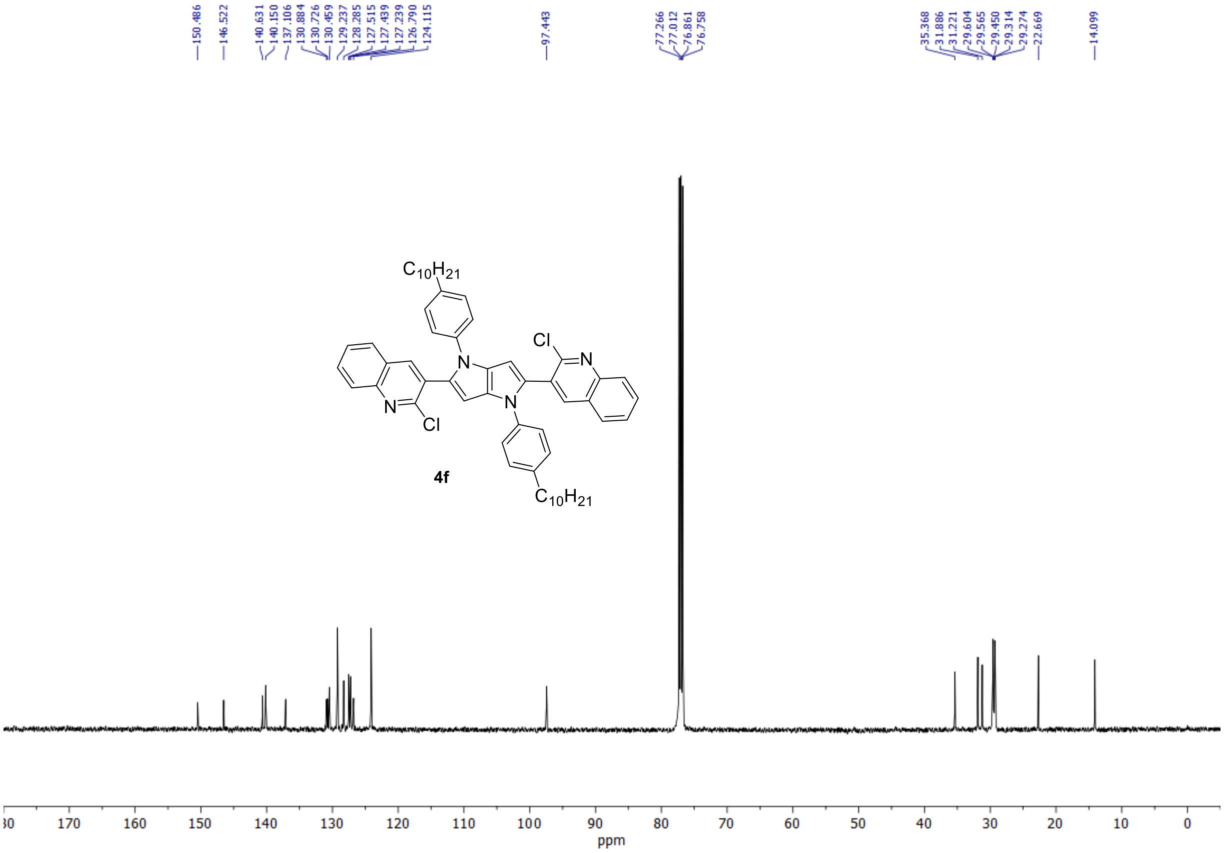

Report of HRMS of **4f**:

Single Mass Analysis

Tolerance = 5.0 mDa / DBE: min = -1.5, max = 500.0

Element prediction: Off

Number of isotope peaks used for i-FIT = 3

Monoisotopic Mass, Even Electron Ions

126 formula(e) evaluated with 2 results within limits (all results (up to 1000) for each mass)

Elements Used:

C: 0-100 H: 0-100 N: 0-4 Cl: 0-2

Mass	Calc. Mass	mDa	PPM	DBE	Formula	i-FIT	i-FIT Norm	Fit Conf %	C	H	N	Cl
861.4433	861.4430	0.3	0.3	26.5	C56 H63 N4 Cl2	587.9	0.000	100.00	56	63	4	2
	861.4460	-2.7	-3.1	39.5	C67 H57	605.2	17.316	0.00	67	57		

^{1}H NMR (500 MHz, 300K, $CDCl_3$) of **4g**

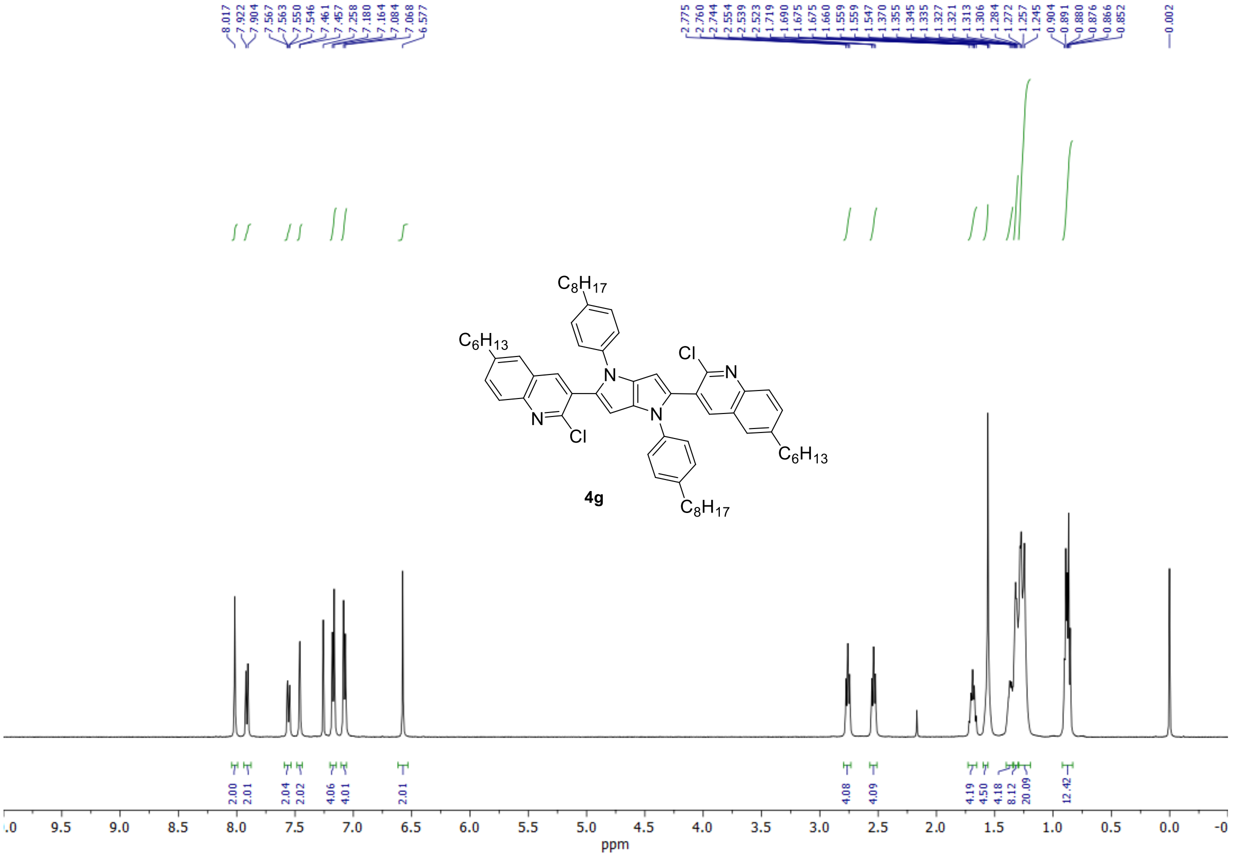

$^{13}C\{^{1}H\}$ NMR (126 MHz, 300K, $CDCl_3$) of **4g**

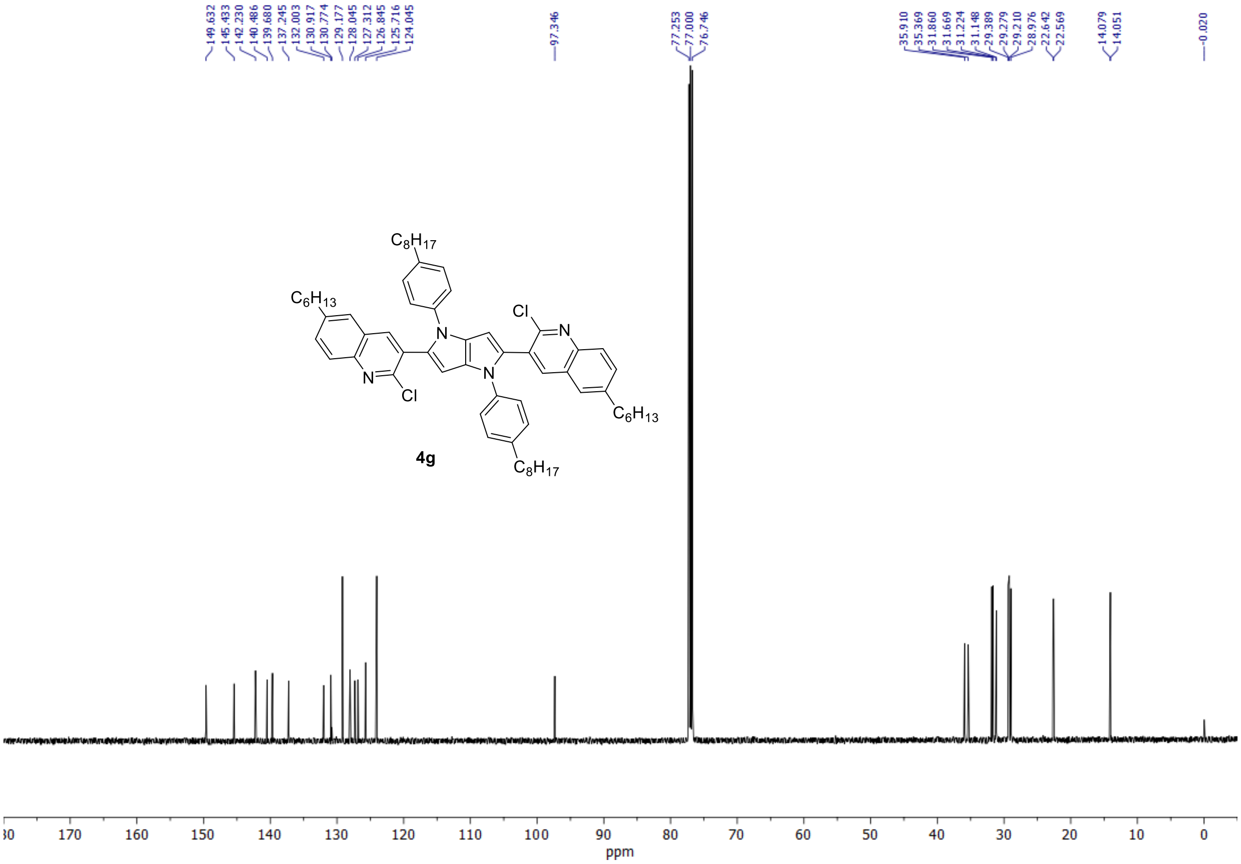

Report of HRMS of **4g**:

Single Mass Analysis
Tolerance = 3.0 mDa / DEE: min = -1.5, max = 200.0
Element prediction: Off
Number of isotope peaks used for i-FIT = 3
Monoisotopic Mass, Even Electron Ions
206 formula(e) evaluated with 2 results within limits (all results (up to 1000) for each mass)
Elements Used:
C: 0-100 H: 0-200 N: 0-5 Cl: 1-3

Mass	Calc. Mass	mDa	PPM	DBE	Formula	i-FIT	i-FIT Norm	Fit Conf %	C	H	N	Cl
973.5696	973.5682	1.4	1.4	26.5	C64 H79 N4 Cl2	136.7	0.872	41.80	64	79	4	2
	973.5700	-0.4	-0.4	21.5	C63 H84 N2 Cl3	136.4	0.541	58.20	63	84	2	3

^{1}H NMR (600 MHz, 300K, $CDCl_3$) of **4h**

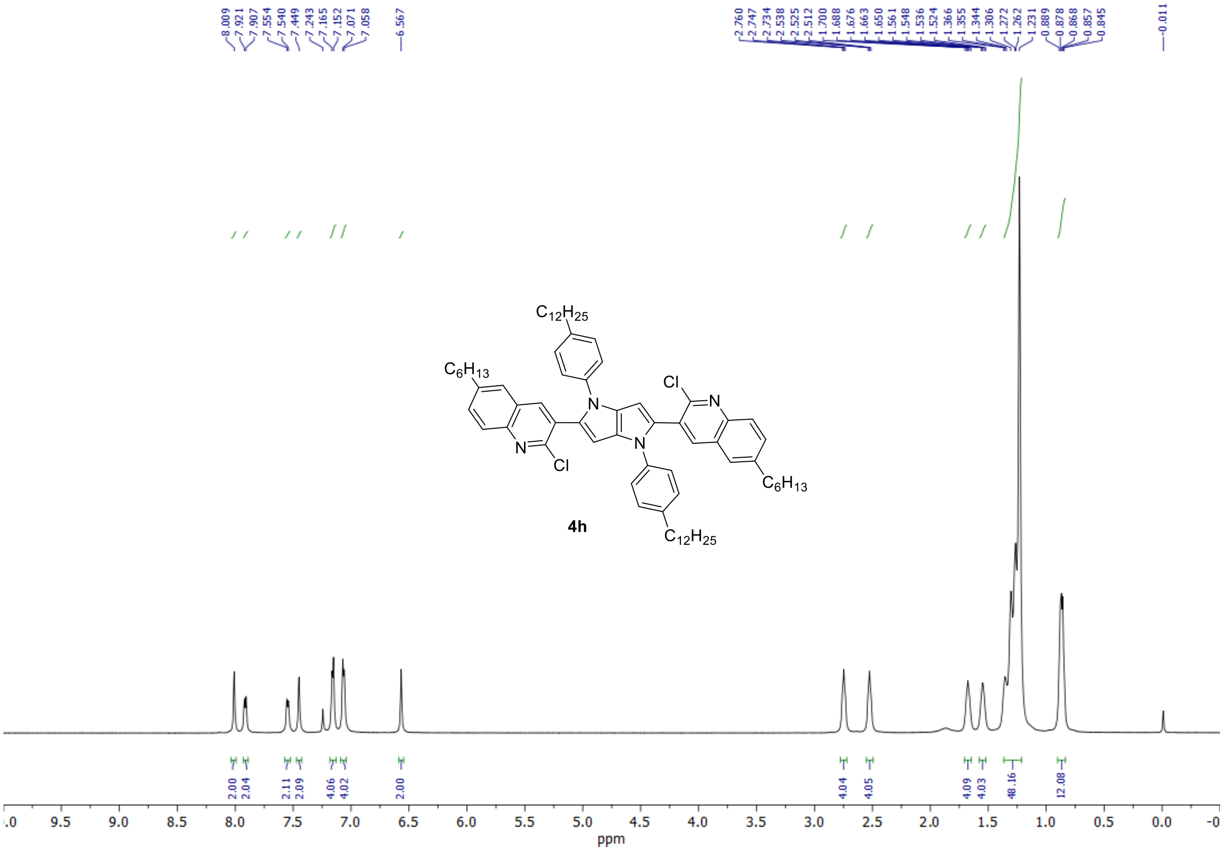

$^{13}C\{^1H\}$ NMR (151 MHz, 300K, $CDCl_3$) of **4h**

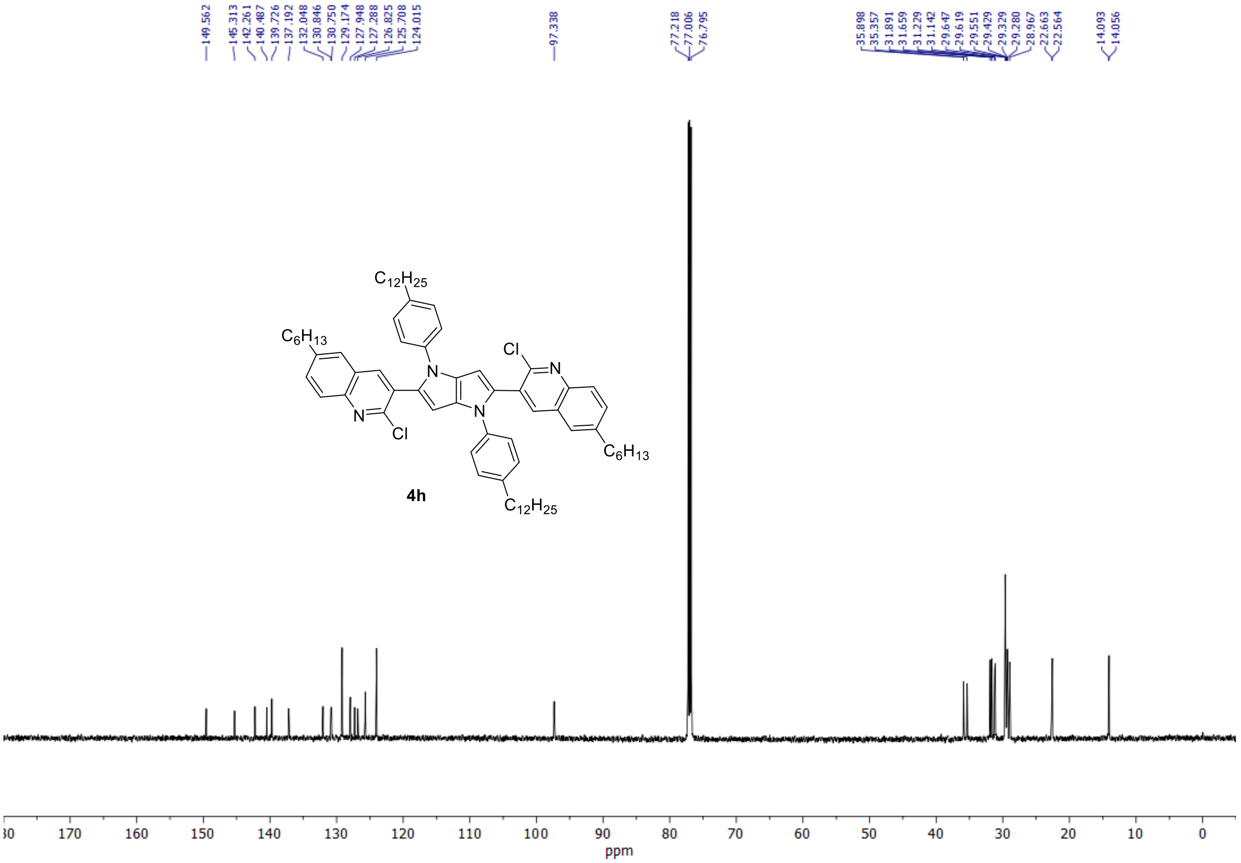

Report of HRMS of **4h**:

Single Mass Analysis
Tolerance = 5.0 PPM / DBE: min = -1.5, max = 500.0
Element prediction: Off
Number of isotope peaks used for i-FIT = 3
Monoisotopic Mass, Even Electron Ions
121 formula(e) evaluated with 1 results within limits (up to 50 closest results for each mass)
Elements Used:
C: 0-200 H: 0-200 N: 2-4 Cl: 0-2

Mass	Calc. Mass	mDa	PPM	DBE	Formula	i-FIT	i-FIT Norm	Fit Conf %	C	H	N	Cl
1085.6943	1085.6934	0.9	0.8	26.5	C72 H95 N4 Cl2	474.4	n/a	n/a	72	95	4	2

^{1}H NMR (600 MHz, 300K, $CDCl_3$) of **4i**

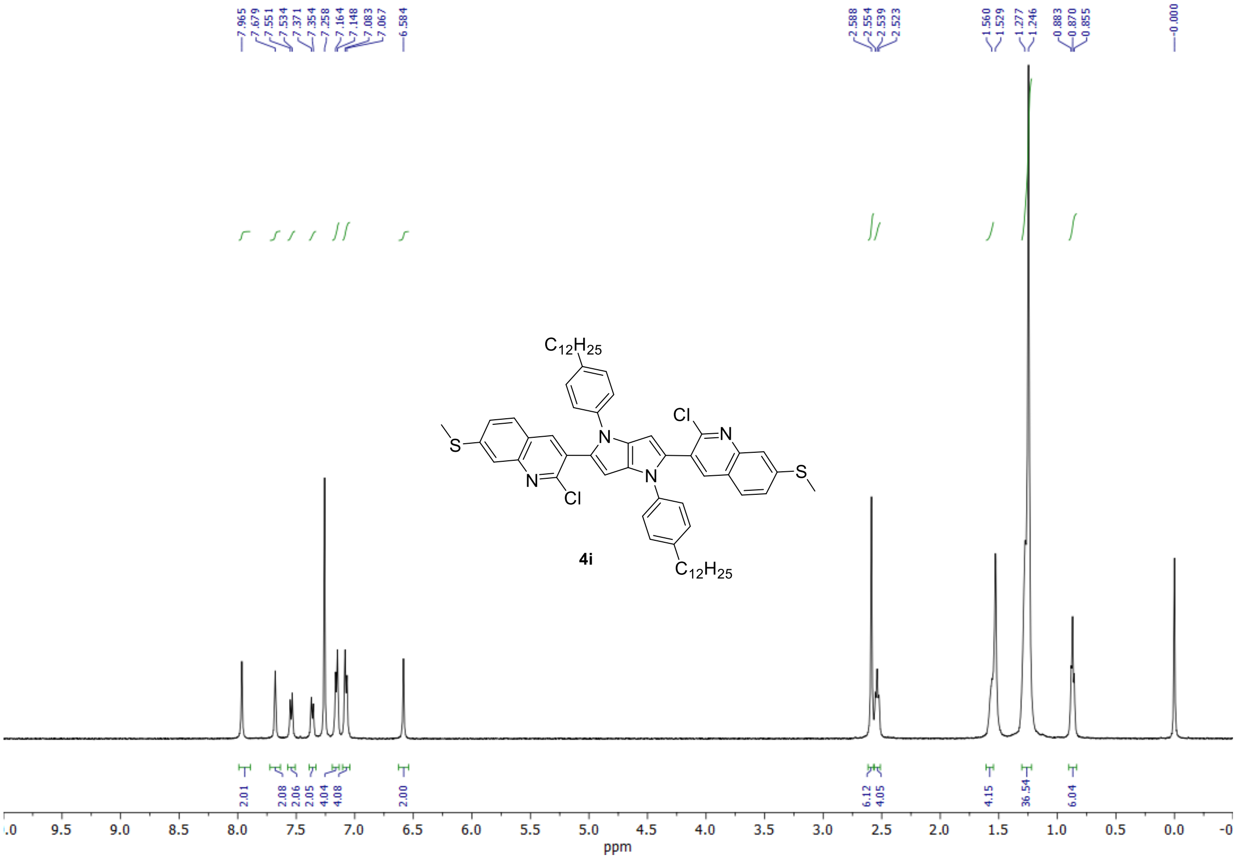

$^{13}C\{^{1}H\}$ NMR (126 MHz, 300K, $CDCl_3$) of **4i**

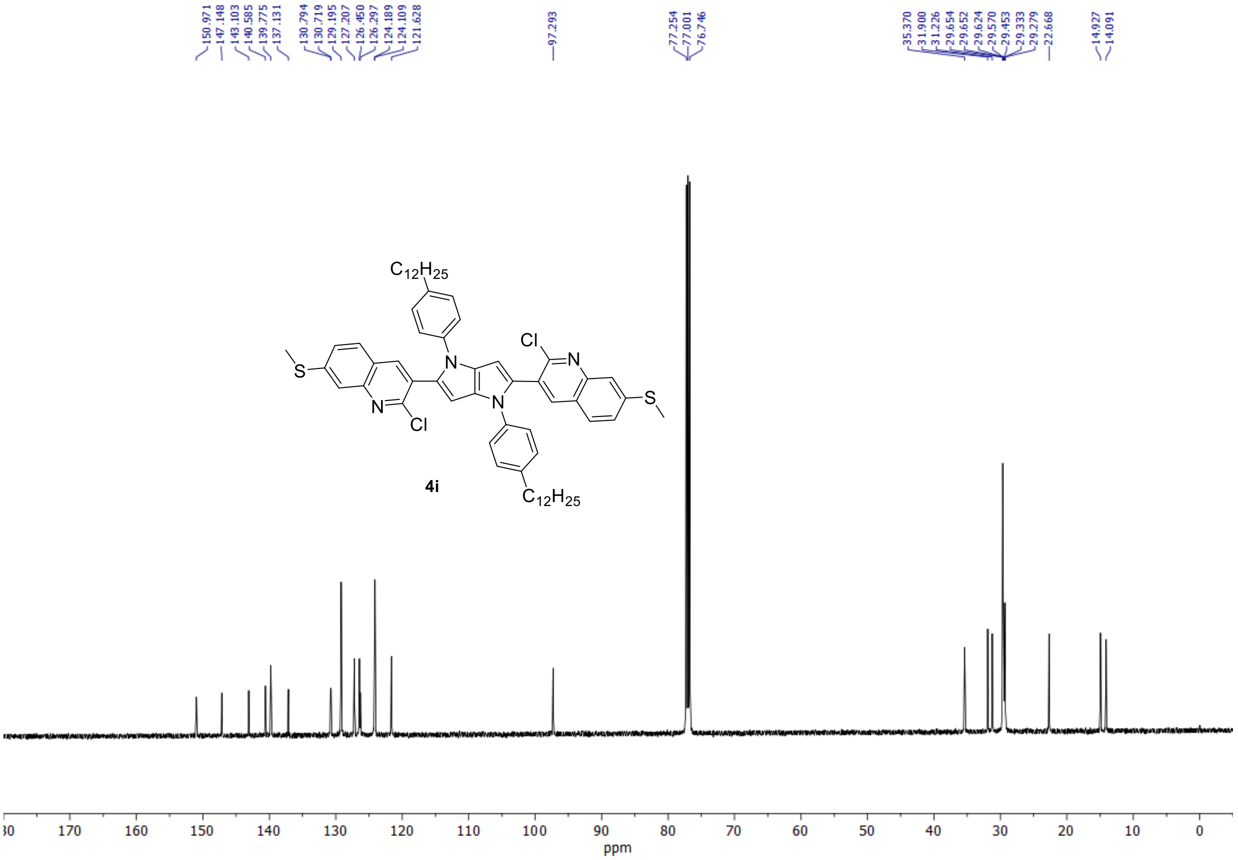

Report of HRMS of **4i**:

Single Mass Analysis
Tolerance = 3.0 mDa / DBE: min = -1.5, max = 150.0
Element prediction: Off
Number of isotope peaks used for i-FIT = 3
Monoisotopic Mass, Even Electron Ions
398 formula(e) evaluated with 4 results within limits (up to 50 closest results for each mass)
Elements Used:
C: 0-100 H: 0-200 N: 2-5 S: 1-3 Cl: 1-3

Mass	Calc. Mass	mDa	PPM	DBE	Formula	i-FIT	i-FIT Norm	Fit Conf %	C	H	N	S	Cl
1009.4819	1009.4810	0.9	0.9	26.5	C62 H75 N4 S2 Cl2	420.2	0.281	75.54	62	75	4	2	2
	1009.4829	-1.0	-1.0	21.5	C61 H80 N2 S2 Cl3	426.6	6.710	0.12	61	80	2	2	3
	1009.4795	2.4	2.4	26.5	C64 H76 N2 S Cl3	427.8	7.849	0.04	64	76	2	1	3
	1009.4844	-2.5	-2.5	21.5	C59 H79 N4 S3 Cl2	421.3	1.415	24.30	59	79	4	3	2

^{1}H NMR (500 MHz, 300K, $CDCl_3$) of **4j**

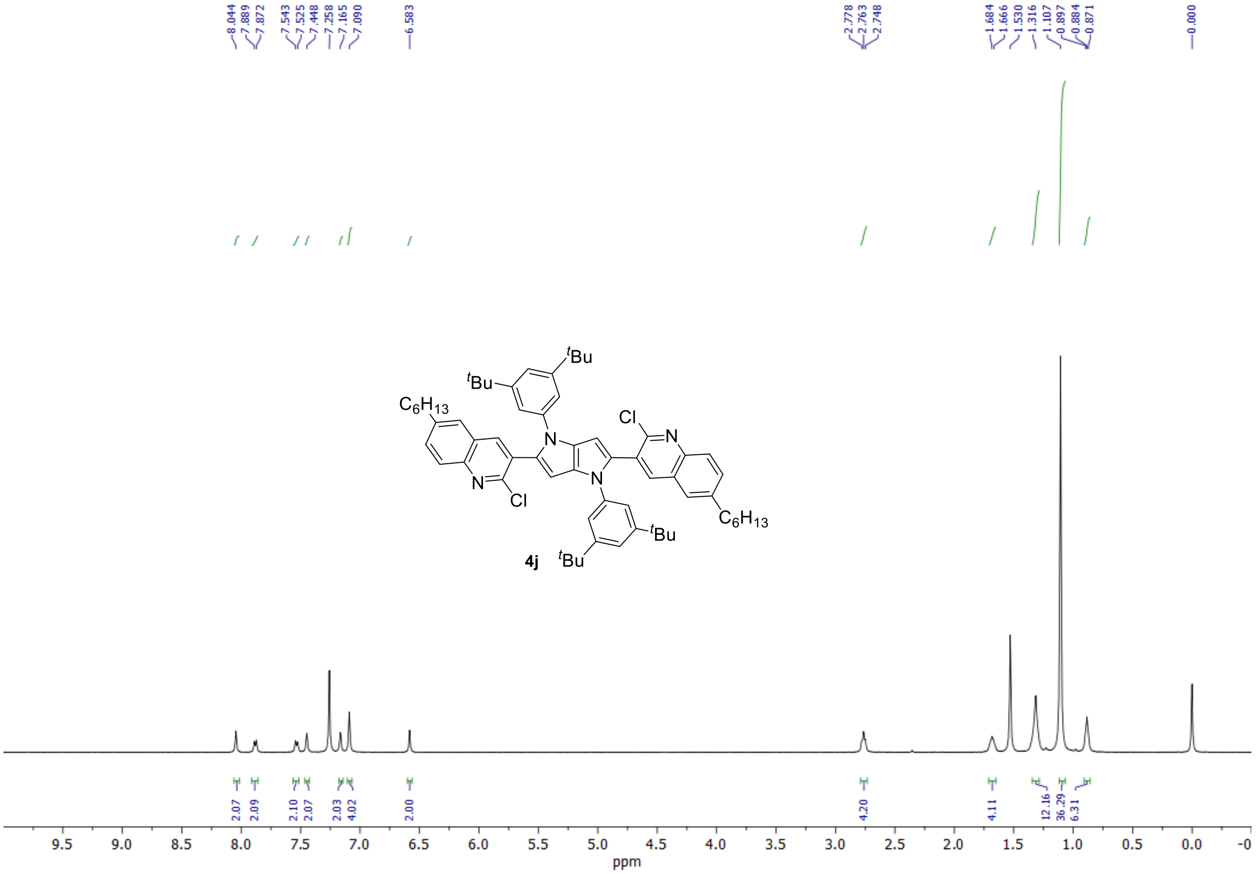

$^{13}C\{^1H\}$ NMR (151 MHz, 300K, THF-d_8) of **4j**

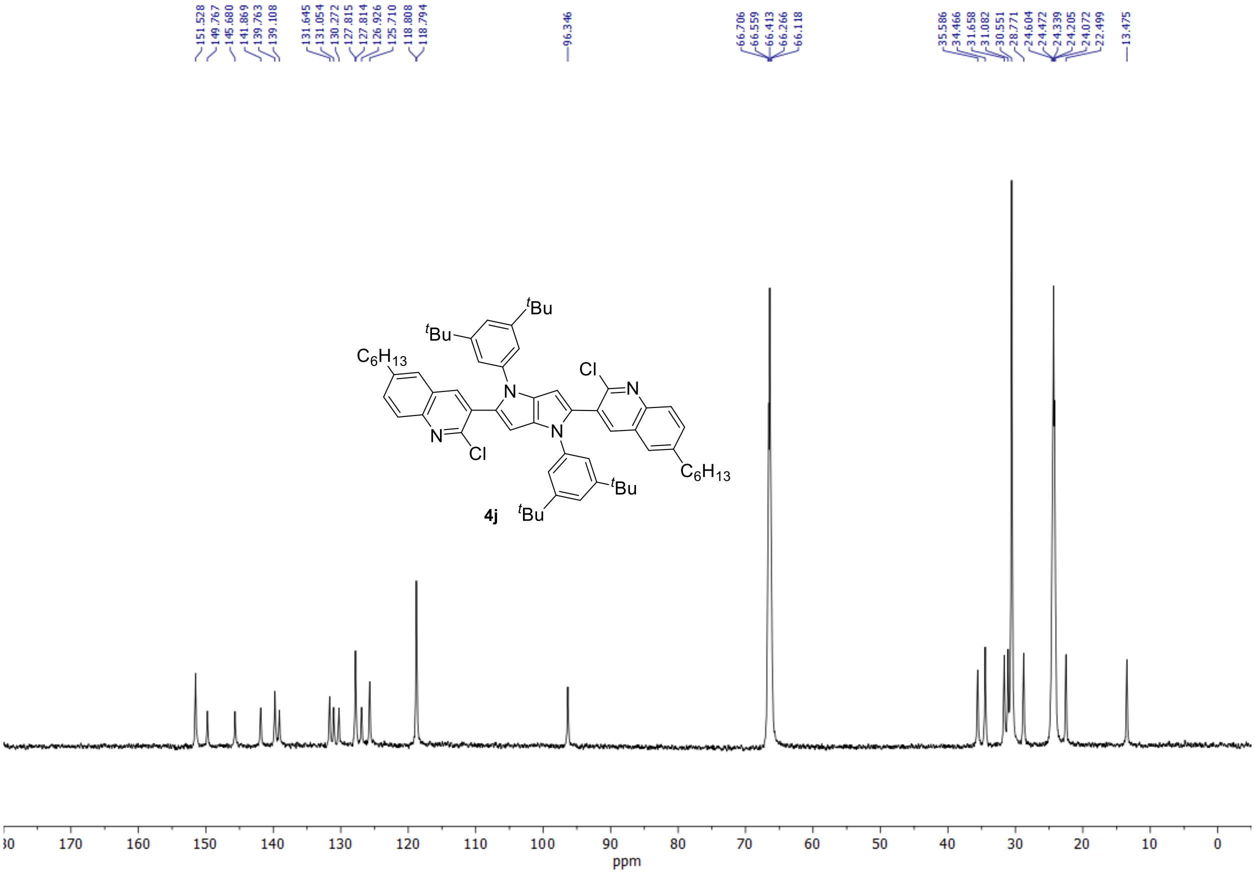

Report of HRMS of **4j**:

Single Mass Analysis
Tolerance = 5.0 PPM / DBE: min = -1.5, max = 500.0
Element prediction: Off
Number of isotope peaks used for i-FIT = 3
Monoisotopic Mass, Even Electron Ions
106 formula(e) evaluated with 1 results within limits (up to 50 closest results for each mass)
Elements Used:
C: 0-200 H: 0-200 N: 2-4 Cl: 0-2

Mass	Calc. Mass	mDa	PPM	DBE	Formula	i-FIT	i-FIT Norm	Fit Conf %	C	H	N	Cl
973.5690	973.5682	0.8	0.8	26.5	C64 H79 N4 Cl2	544.0	n/a	n/a	64	79	4	2

^{1}H NMR (500 MHz, 300K, $CDCl_3$) of **4k**

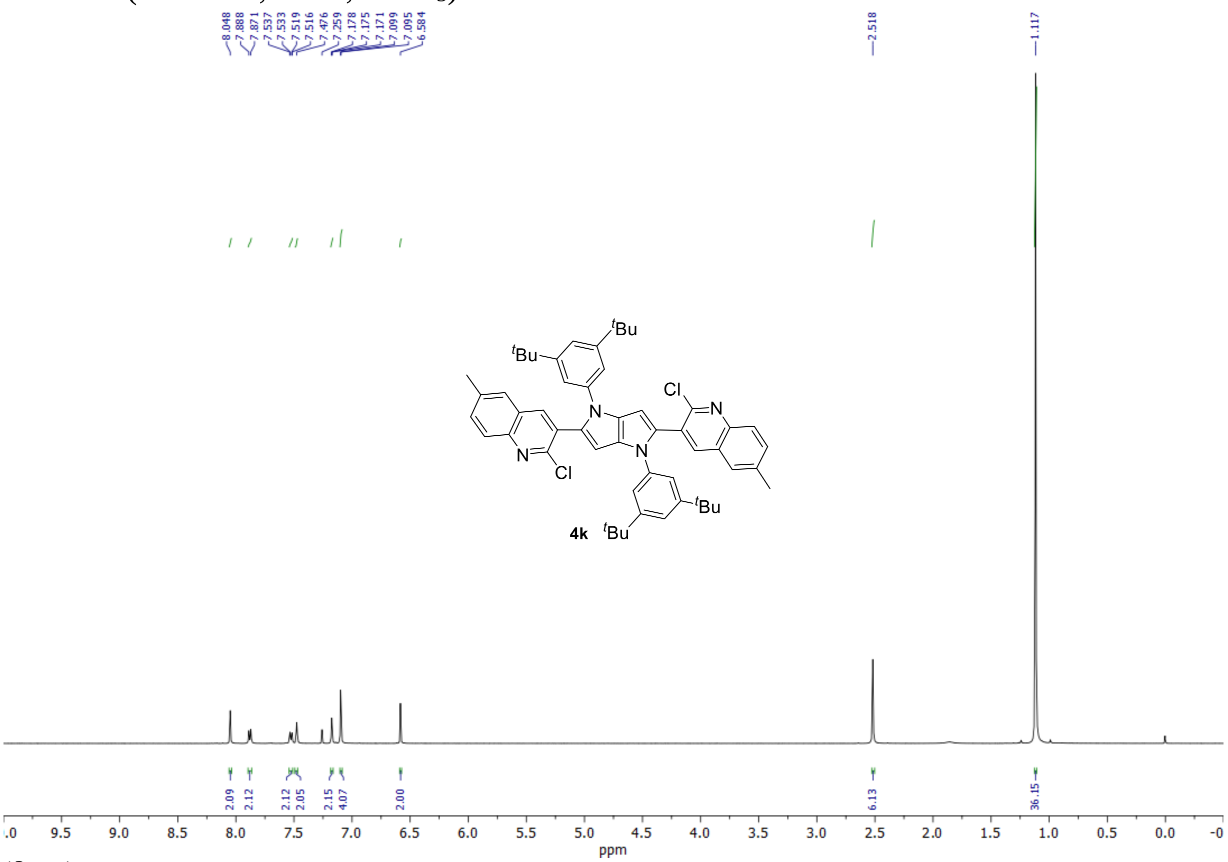

^{13}C{^{1}H} NMR (126 MHz, 300K, $CDCl_3$) of **4k**

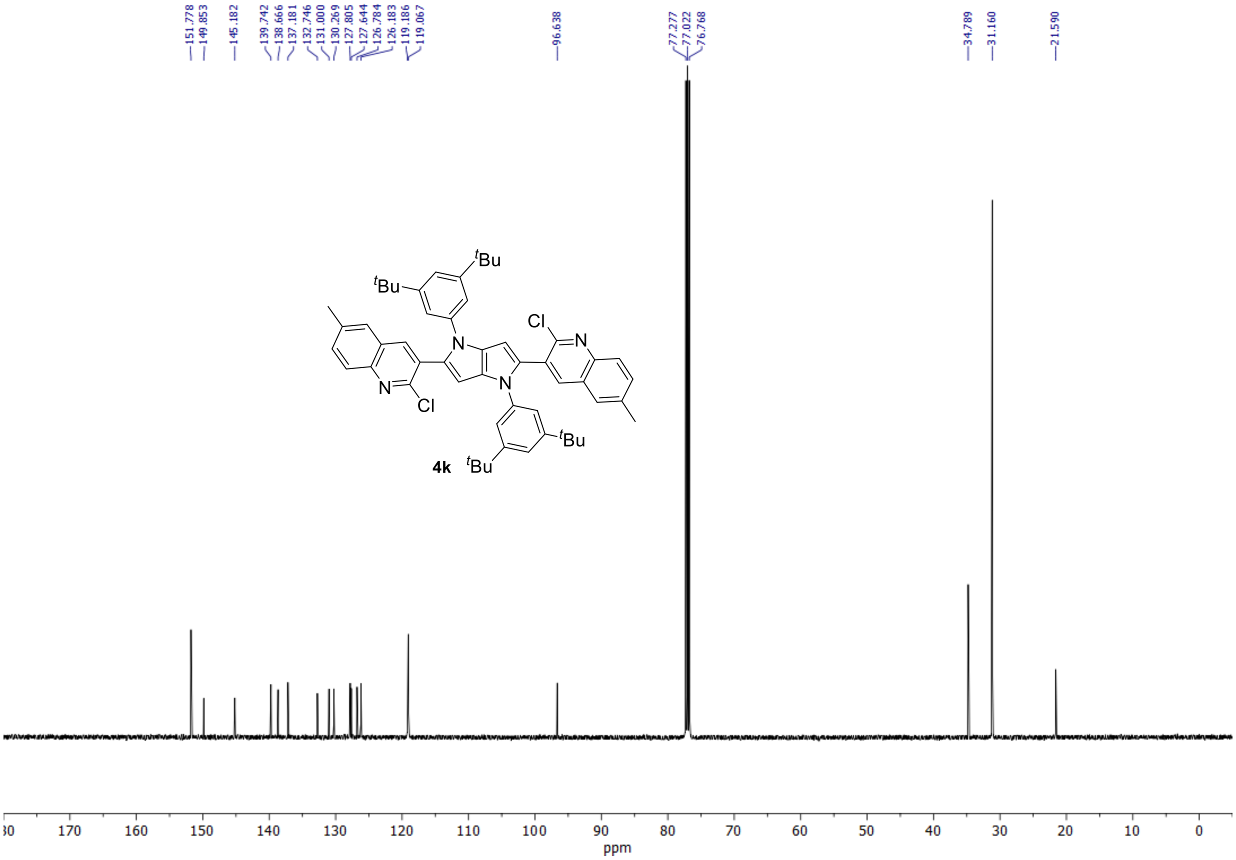

Report of HRMS of **4k**:

Single Mass Analysis
Tolerance = 5.0 PPM / DBE: min = -1.5, max = 500.0
Element prediction: Off
Number of isotope peaks used for i-FIT = 3
Monoisotopic Mass, Even Electron Ions
93 formula(e) evaluated with 1 results within limits (up to 50 closest results for each mass)
Elements Used:
C: 0-200 H: 0-200 N: 2-4 Cl: 0-2

Mass	Calc. Mass	mDa	PPM	DBE	Formula	i-FIT	i-FIT Norm	Fit Conf %	C	H	N	Cl
833.4122	833.4117	0.5	0.6	26.5	C54 H59 N4 Cl2	552.0	n/a	n/a	54	59	4	2

^{1}H NMR (600 MHz, 300K, $CDCl_3$) of **4l**

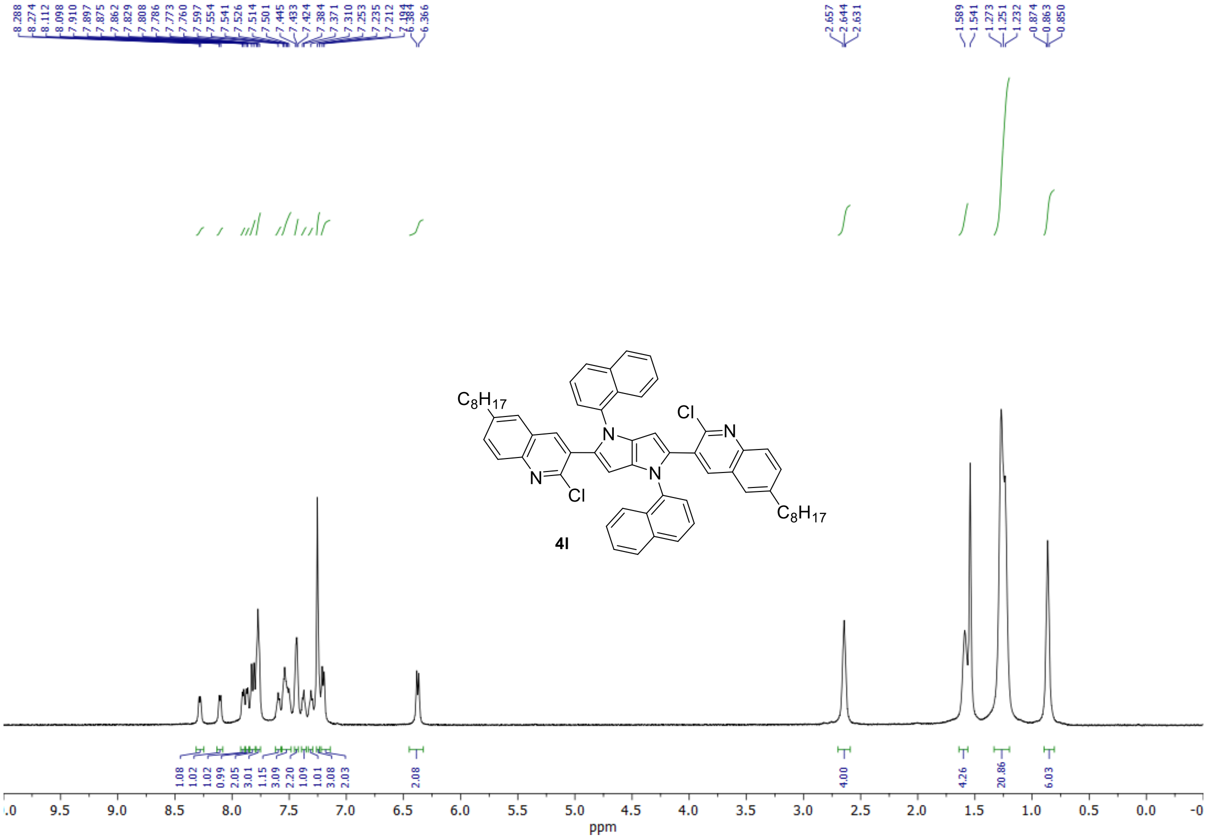

$^{13}C\{^{1}H\}$ NMR (126 MHz, 300K, $CDCl_3$) of **4l**

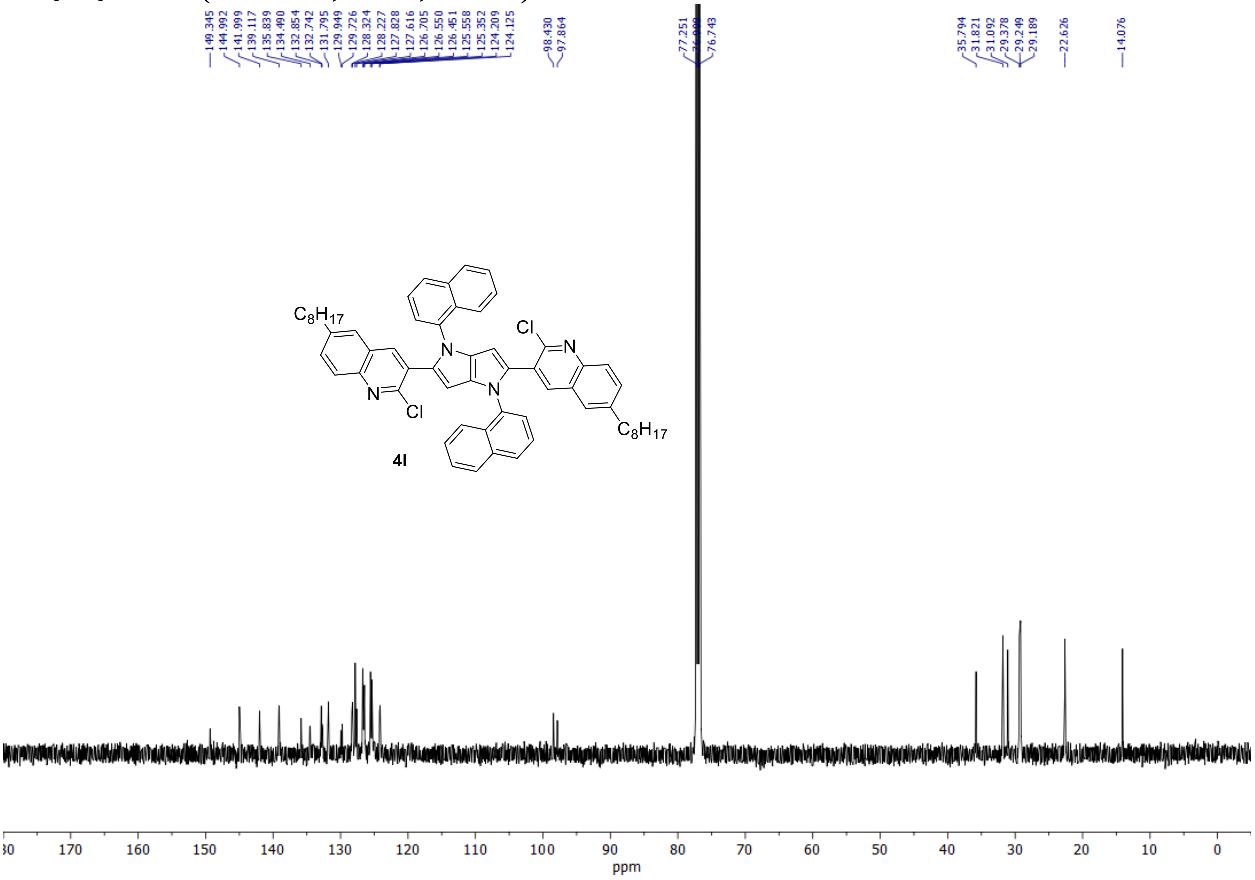

Report of HRMS of **4l**:

Single Mass Analysis

Tolerance = 3.0 mDa / DBE: min = -1.5, max = 150.0

Element prediction: Off

Number of isotope peaks used for i-FIT = 3

Monoisotopic Mass, Even Electron Ions

130 formula(e) evaluated with 1 results within limits (up to 200 closest results for each mass)

Elements Used:

C: 0-200 H: 0-200 N: 0-5 Cl: 1-2

Mass	Calc. Mass	mDa	PPM	DBE	Formula	i-FIT	i-FIT Norm	Fit Conf %	C	H	N	Cl
905.4118	905.4117	0.1	0.1	32.5	C60 H59 N4 Cl2	328.3	n/a	n/a	60	59	4	2

^{1}H NMR (600 MHz, 300K, $CDCl_3$) of **6a**

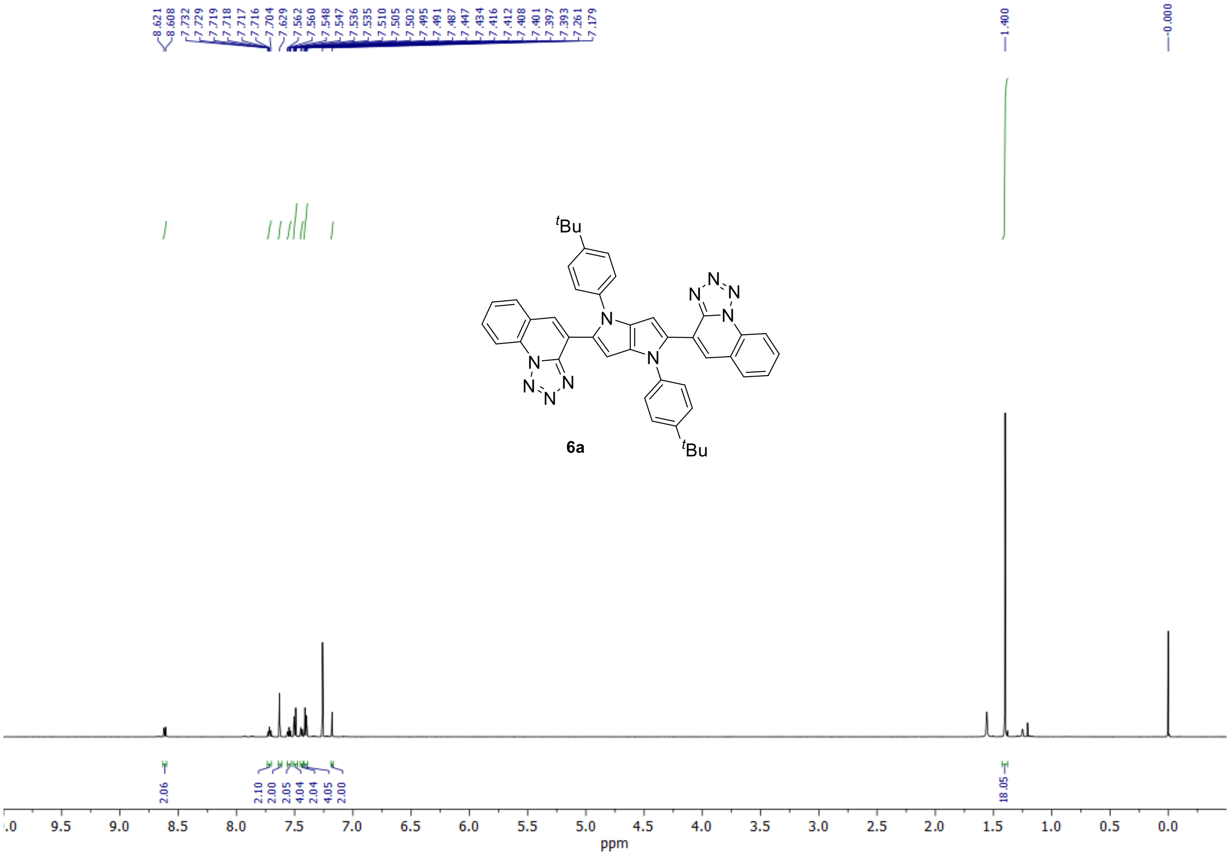

$^{13}C\{^{1}H\}$ NMR (126 MHz, 350K, tetrachloroethane-[D_2]) of **6a**

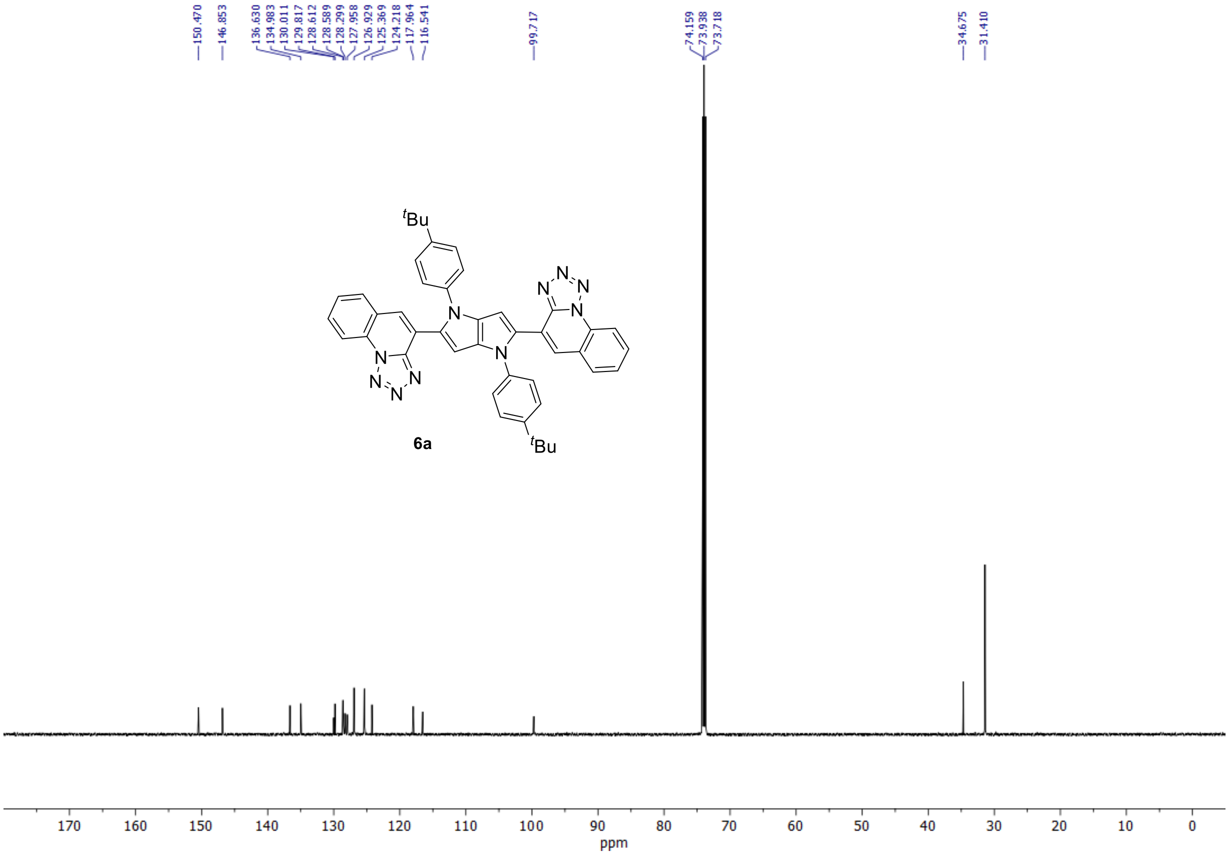

Report of HRMS of **6a**:

Single Mass Analysis
Tolerance = 3.0 mDa / DBE: min = -1.5, max = 500.0
Element prediction: Off
Number of isotope peaks used for i-FIT = 3
Monoisotopic Mass, Odd and Even Electron Ions
107 formula(e) evaluated with 1 results within limits (up to 50 closest results for each mass)
Elements Used:
C: 0-200 H: 0-200 N: 0-11

Mass	Calc. Mass	mDa	PPM	DBE	Formula	i-FIT	i-FIT Norm	Fit Conf %	C	H	N
707.3362	707.3359	0.3	0.4	30.5	C44 H39 N10	647.6	n/a	n/a	44	39	10

^{1}H NMR (500 MHz, 300K, $CDCl_3$) of **6b**

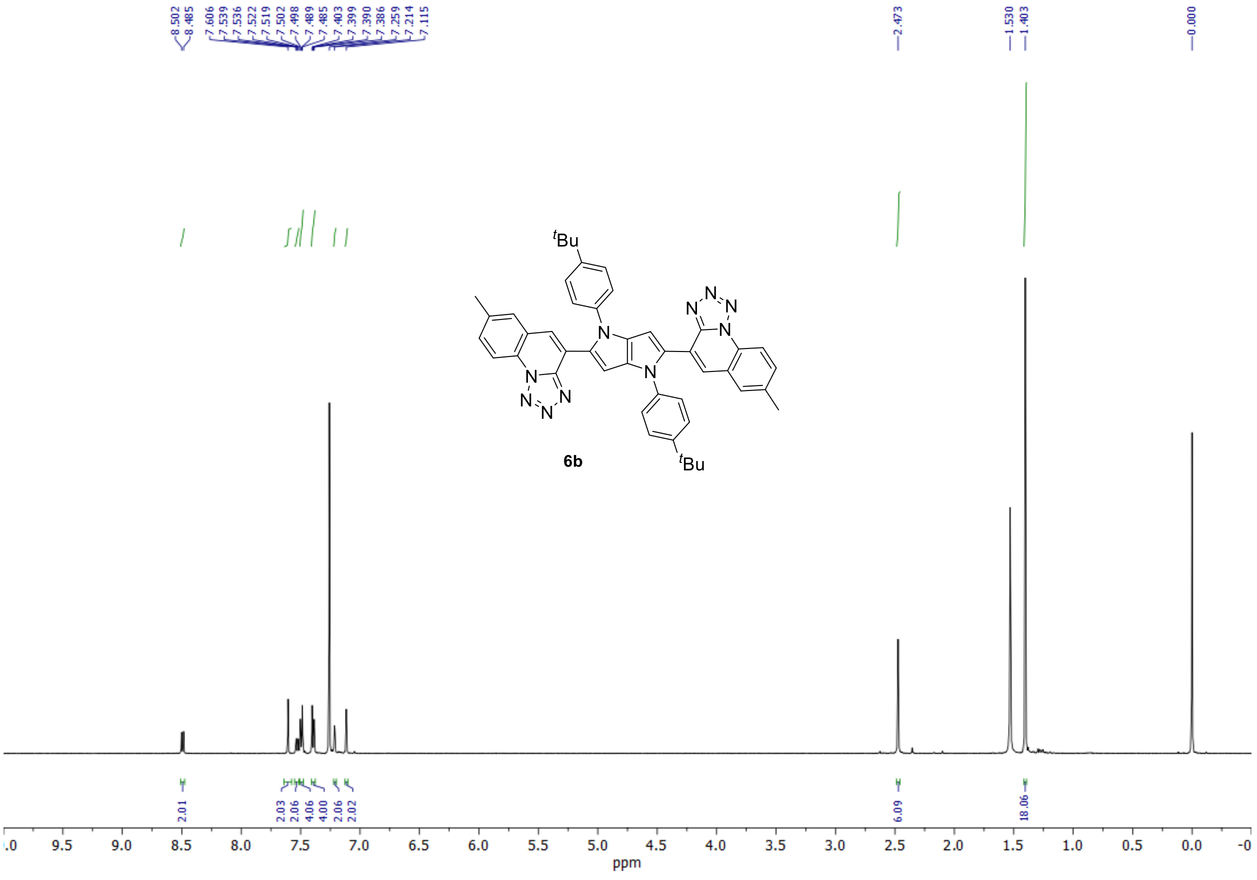

$^{13}C\{^{1}H\}$ NMR (126 MHz, 300K, $CDCl_3$) of **6b**

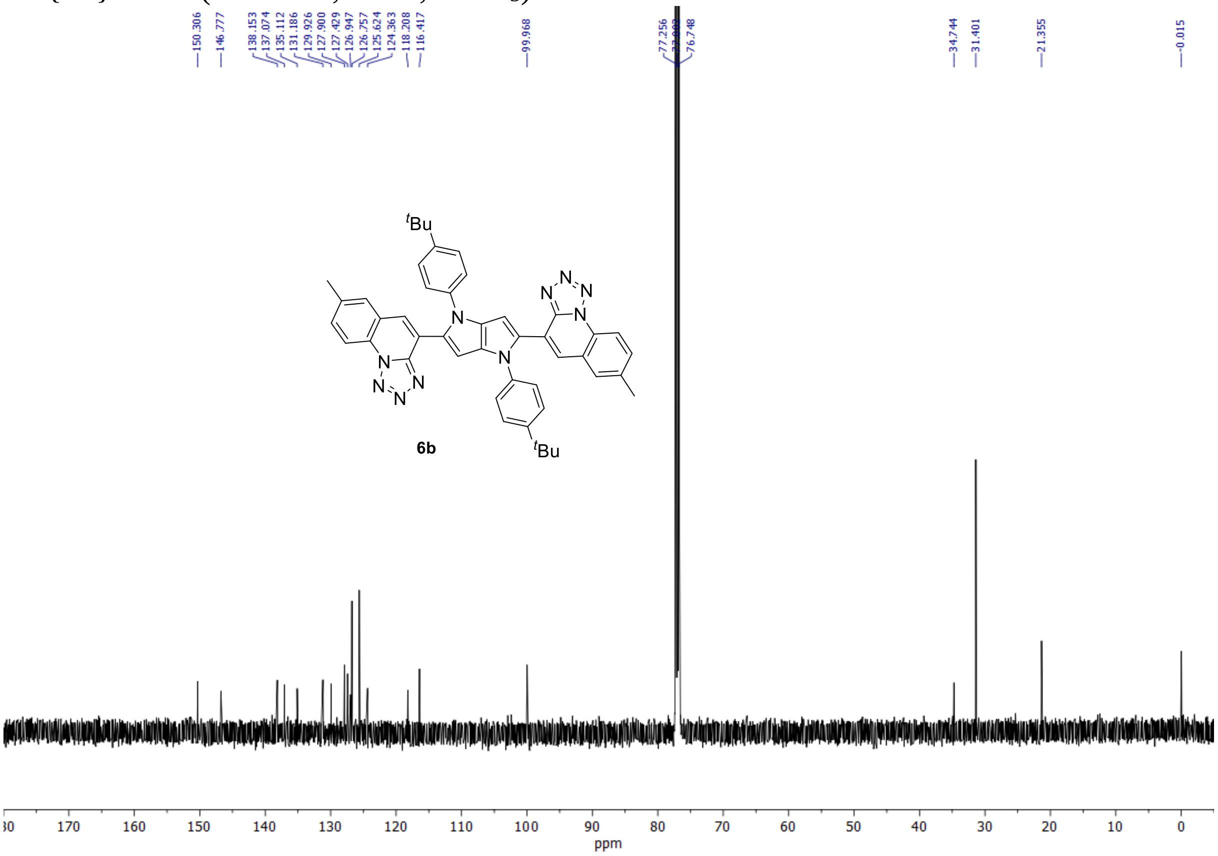

Report of HRMS of **6b**:

Single Mass Analysis
Tolerance = 3.0 mDa / DBE: min = -1.5, max = 500.0
Element prediction: Off
Number of isotope peaks used for i-FIT = 3
Monoisotopic Mass, Even Electron Ions
111 formula(e) evaluated with 1 results within limits (up to 50 closest results for each mass)

Elements Used:
C: 0-200 H: 0-200 N: 0-11

Mass	Calc. Mass	mDa	PPM	DBE	Formula	i-FIT	i-FIT Norm	Fit Conf %	C	H	N
735.3674	735.3672	0.2	0.3	30.5	C46 H43 N10	617.1	n/a	n/a	46	43	10

^{1}H NMR (500 MHz, 300K, $CDCl_3$) of **6c**

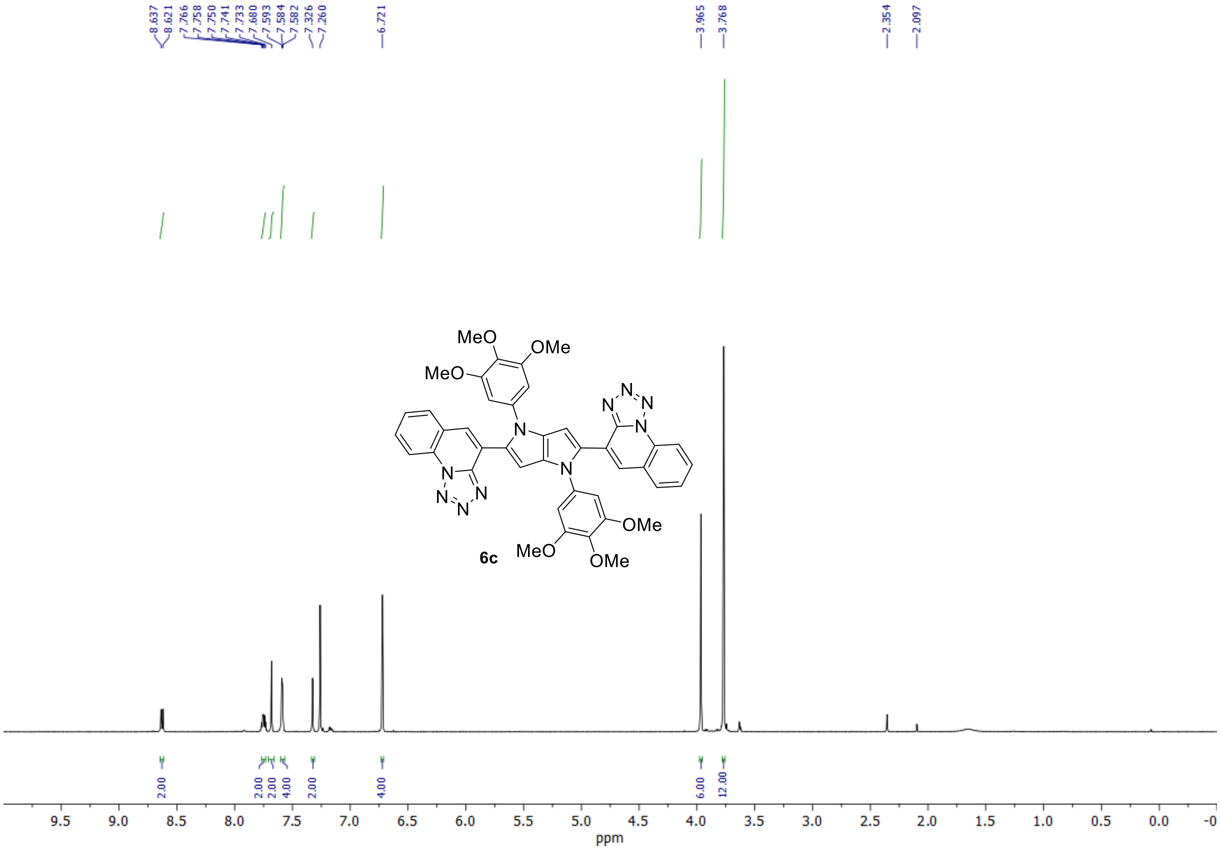

$^{13}C\{^{1}H\}$ NMR (126 MHz, 350K, tetrachloroethane-[D_2]) of **6c**

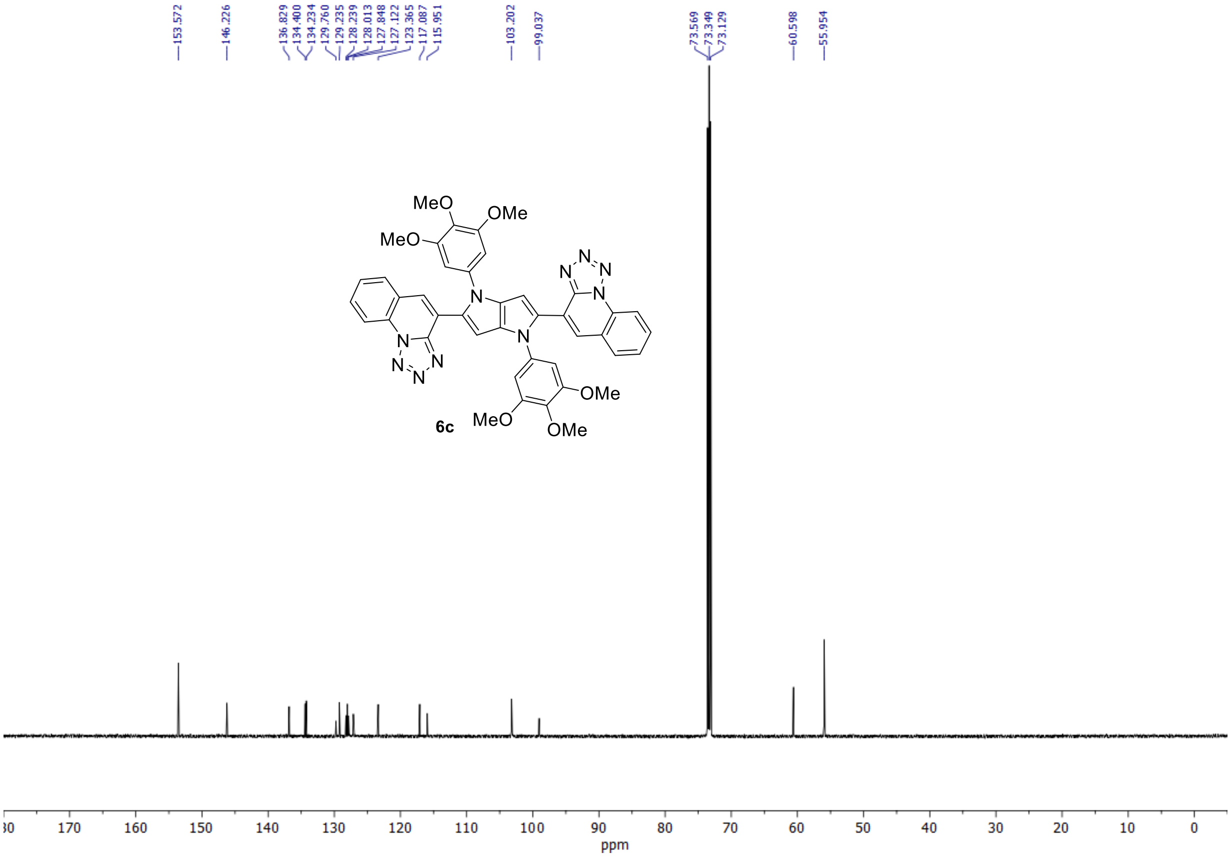

Report of HRMS of **6c**:

Single Mass Analysis
Tolerance = 3.0 mDa / DBE: min = -1.5, max = 500.0
Element prediction: Off
Number of isotope peaks used for i-FIT = 3
Monoisotopic Mass, Even Electron Ions
873 formula(e) evaluated with 2 results within limits (up to 50 closest results for each mass)
Elements Used:

Mass	Calc. Mass	mDa	PPM	DBE	Formula	i-FIT	i-FIT Norm	Fit Conf %	C	H	N	O
775.2742	775.2741	0.1	0.1	30.5	C42 H35 N10 O6	538.9	0.002	99.77	42	35	10	6
	775.2749	-0.7	-0.9	42.5	C58 H35 N2 O	545.0	6.073	0.23	58	35	2	1

^{1}H NMR (500 MHz, 350K, tetrachloroethane-[D_2]) of **6d**

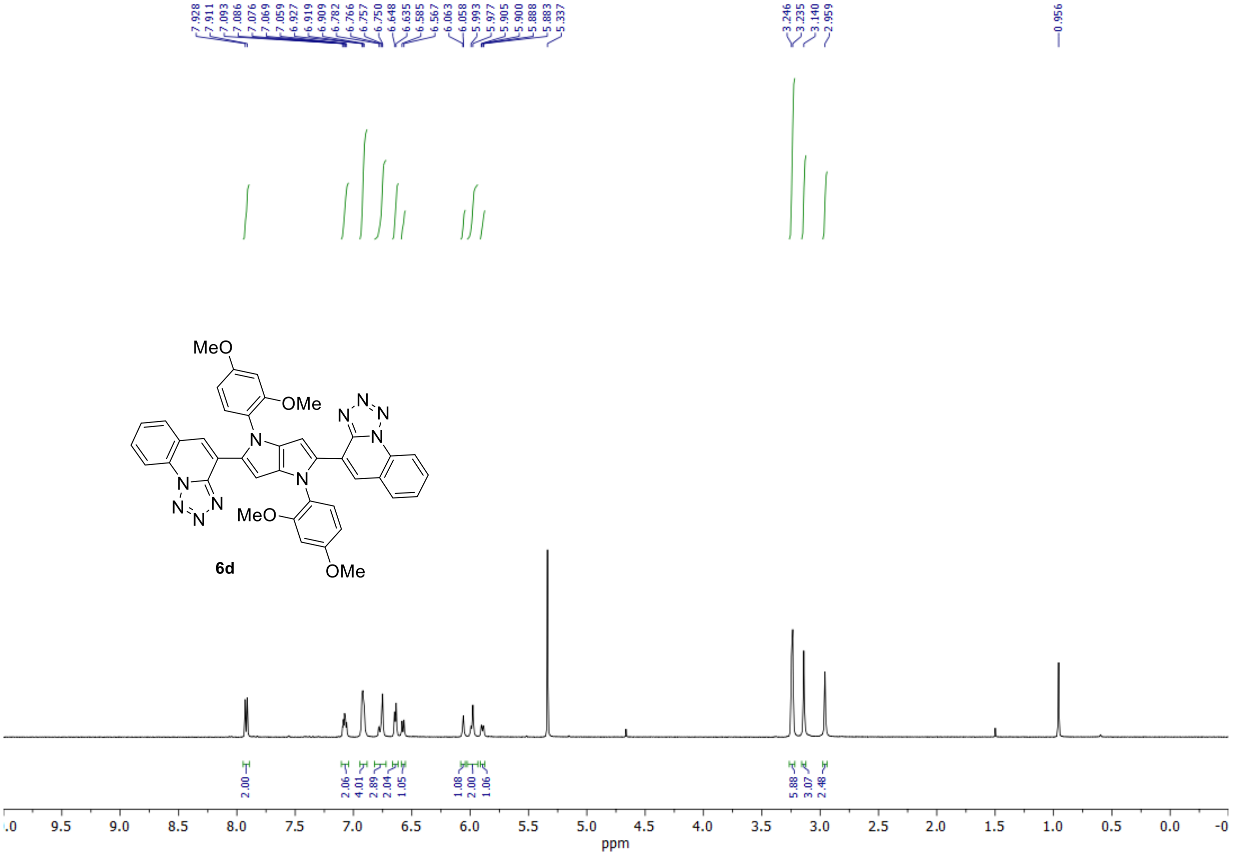

$^{13}C\{^1H\}$ NMR (126 MHz, 350K, tetrachloroethane-[D_2]) of **6d**

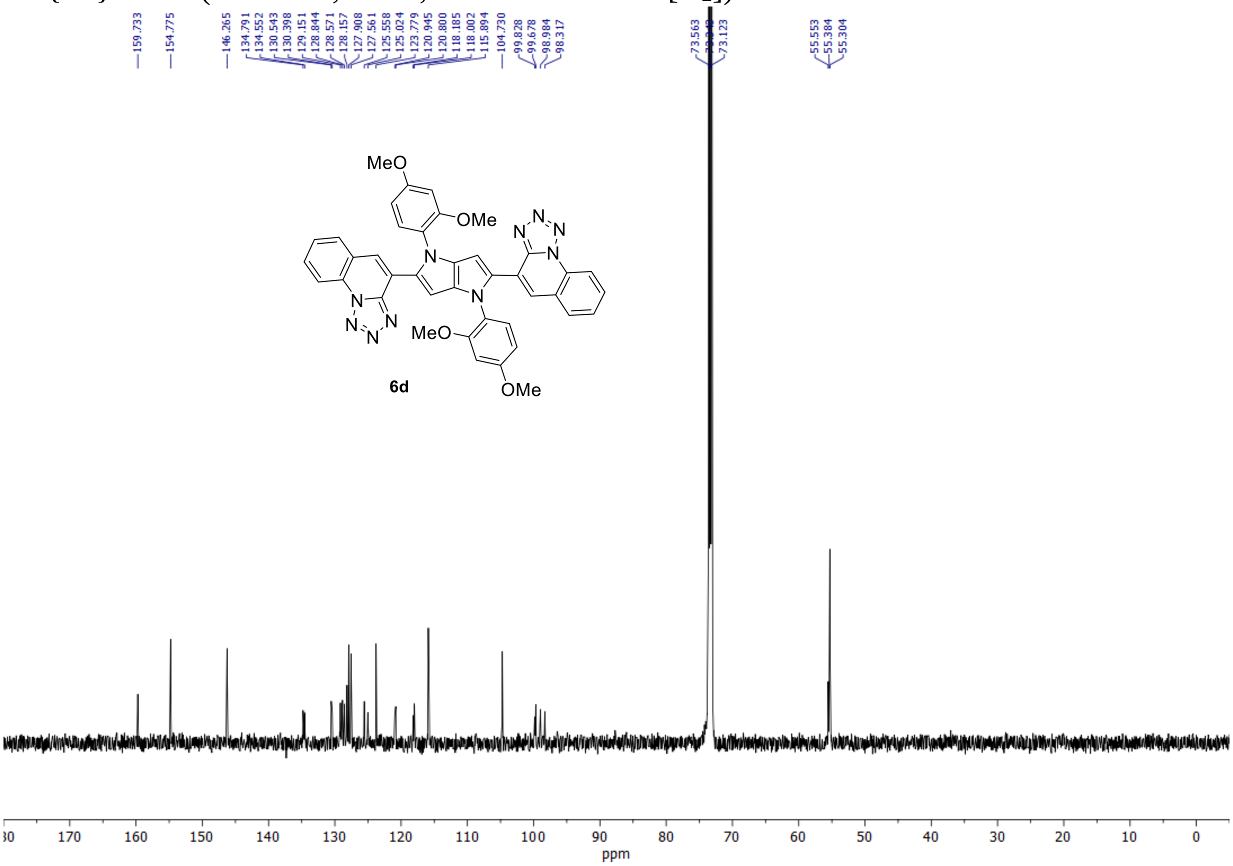

Report of HRMS of **6d**:

Single Mass Analysis
Tolerance = 3.0 mDa / DBE: min = -1.5, max = 500.0
Element prediction: Off
Number of isotope peaks used for i-FIT = 3
Monoisotopic Mass, Even Electron Ions
617 formula(e) evaluated with 2 results within limits (up to 50 closest results for each mass)

Elements Used:
C: 0-200 H: 0-200 N: 0-11 O: 0-5

Mass	Calc. Mass	mDa	PPM	DBE	Formula	i-FIT	i-FIT Norm	Fit Conf %	C	H	N	O
715.2524	715.2530	-0.6	-0.8	30.5	C40 H31 N10 O4	503.2	0.116	89.02	40	31	10	4
	715.2498	2.6	3.6	38.5	C51 H31 N4 O	505.3	2.209	10.98	51	31	4	1

^{1}H NMR (500 MHz, 350K, tetrachloroethane-[D_2]) of **6e**

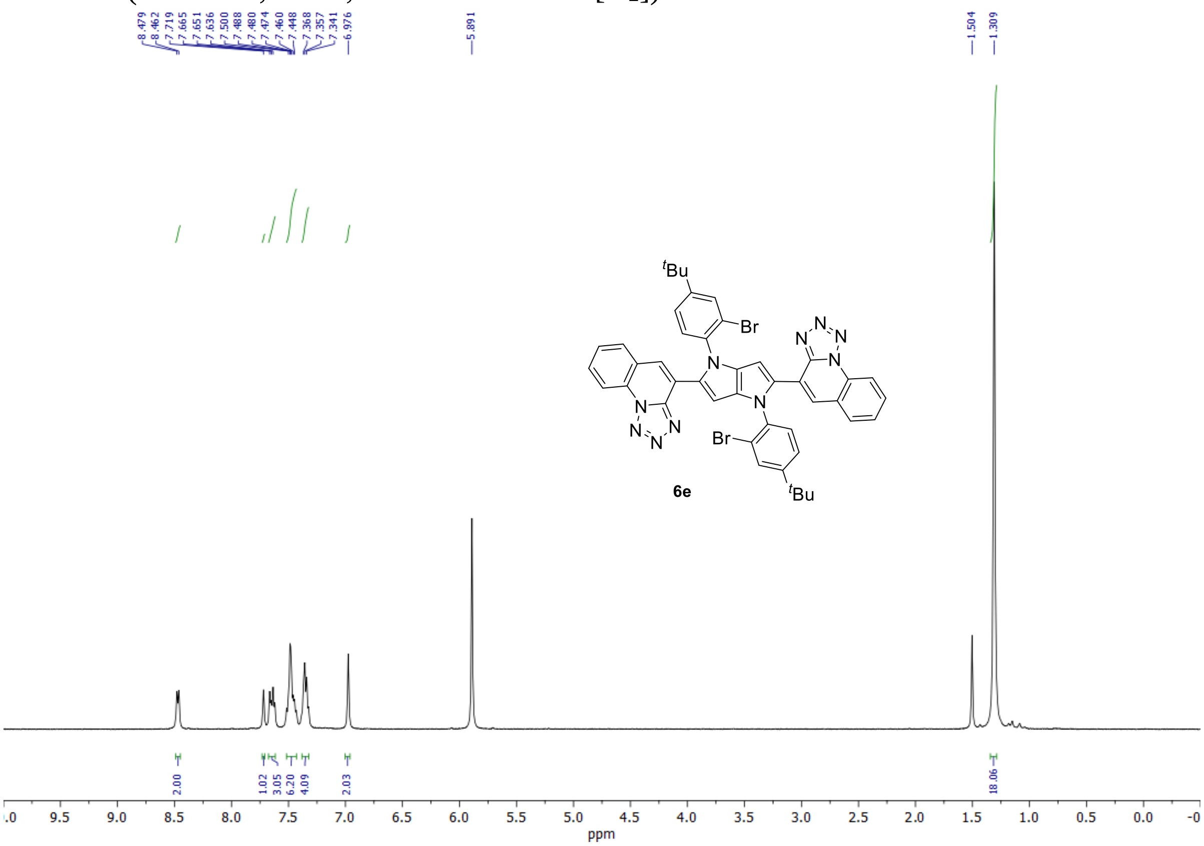

$^{13}C\{^{1}H\}$ NMR (126 MHz, 350K, tetrachloroethane-[D_2]) of **6e**

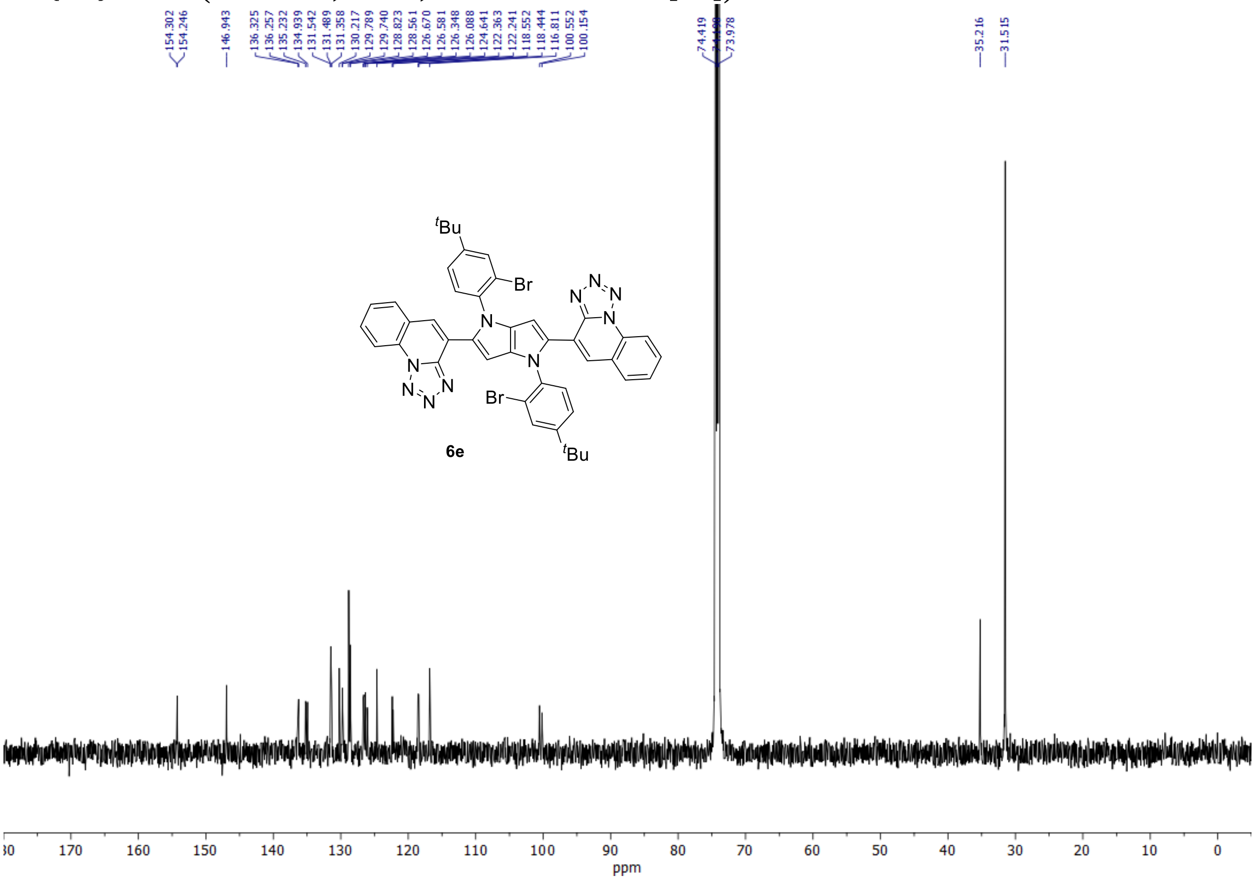

Report of HRMS of **6e**:

Single Mass Analysis
Tolerance = 3.0 mDa / DBE: min = -1.5, max = 500.0
Element prediction: Off
Number of isotope peaks used for i-FIT = 3
Monoisotopic Mass, Even Electron Ions
105 formula(e) evaluated with 1 results within limits (up to 50 closest results for each mass)
Elements Used:
C: 0-200 H: 0-200 N: 0-11 Br: 2-2

Mass	Calc. Mass	mDa	PPM	DBE	Formula	i-FIT	i-FIT Norm	Fit Conf %	C	H	N	Br
863.1560	863.1569	-0.9	-1.0	30.5	C44 H37 N10 Br2	353.1	n/a	n/a	44	37	10	2

^{1}H NMR (600 MHz, 350K, tetrachloroethane-[D_2]) of **6f**

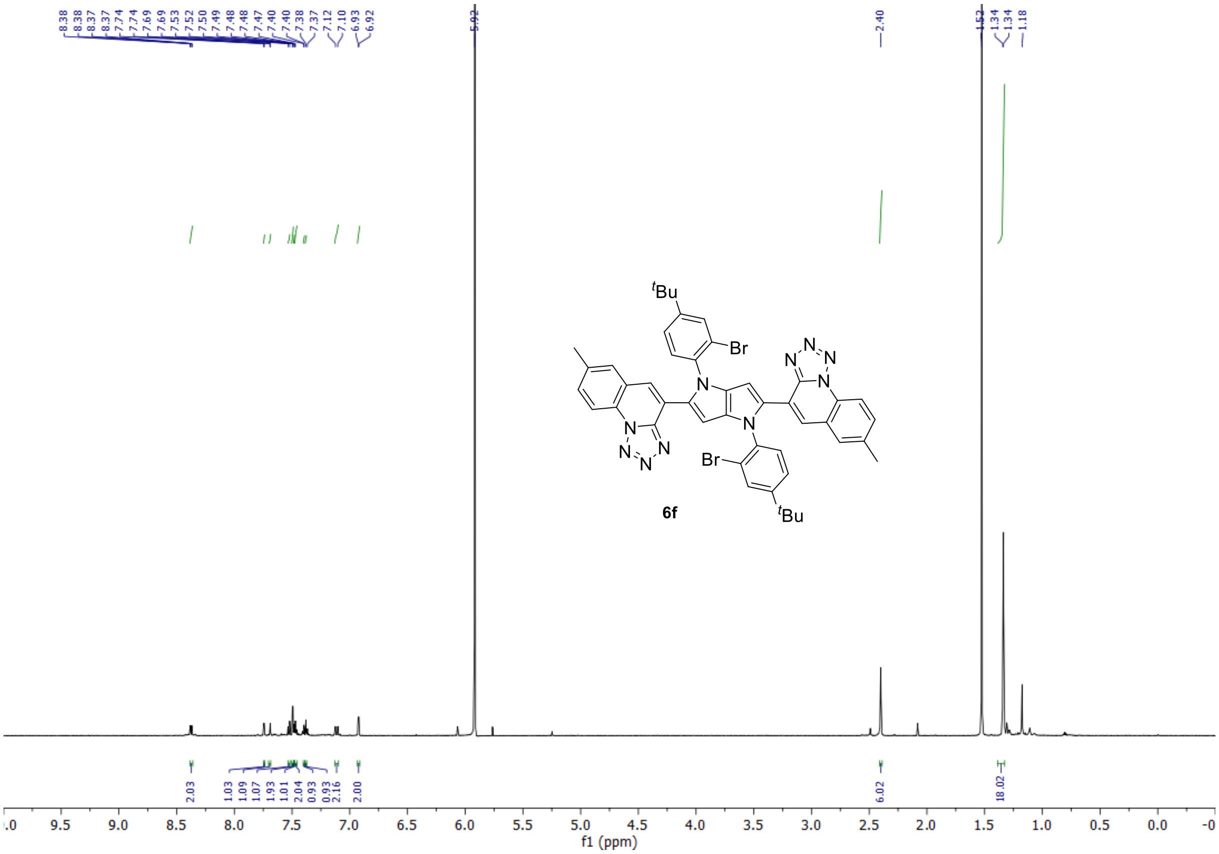

Report of HRMS of **6f**:

Single Mass Analysis
Tolerance = 3.0 mDa / DBE: min = -1.5, max = 100.0
Element prediction: Off
Number of isotope peaks used for i-FIT = 3
Monoisotopic Mass, Odd and Even Electron Ions
263 formula(e) evaluated with 3 results within limits (up to 50 closest results for each mass)
Elements Used:
C: 0-60 H: 0-100 N: 0-12 Na: 0-1 Br: 0-1

Mass	Calc. Mass	mDa	PPM	DBE	Formula	i-FIT	i-FIT Norm	Fit Conf %	C	H	N	Na	Br
444.0793	444.0800	-0.7	-1.6	13.5	C21 H20 N5 Na Br	539.9	0.000	100.00	21	20	5	1	1
	444.0789	0.4	0.9	28.5	C33 H11 N Na	564.8	24.917	0.00	33	11	1	1	
	444.0813	-2.0	-4.5	31.5	C35 H10 N	564.9	25.027	0.00	35	10	1		

Mass	Calc. Mass	mDa	PPM	DBE	Formula	i-FIT	i-FIT Norm	Fit Conf %	C	H	N	Na	Br
915.1823	915.1858	-3.5	-3.8	29.5	C46 H42 N10 Na Br2	412.8	0.091	91.33	46	42	10	1	2
	915.1800	2.3	2.5	33.0	C54 H44 N3 Na Br2	415.9	3.202	4.07	54	44	3	1	2
	915.1824	-0.1	-0.1	36.0	C56 H43 N3 Br2	415.8	3.079	4.60	56	43	3		2

^{1}H NMR (500 MHz, 350K, tetrachloroethane-[D_2]) of **6g**

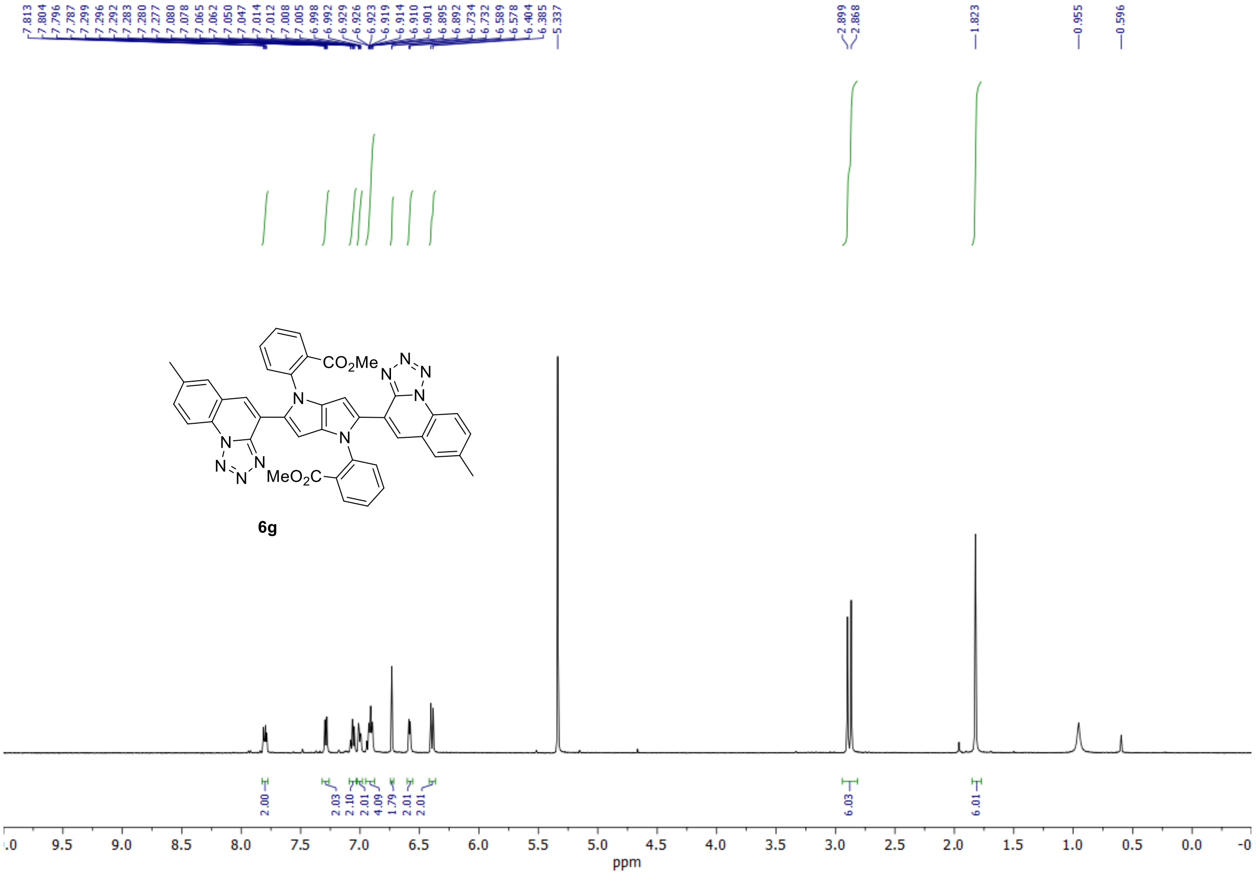

$^{13}C\{^1H\}$ NMR (126 MHz, 350K, tetrachloroethane-[D_2]) of **6g**

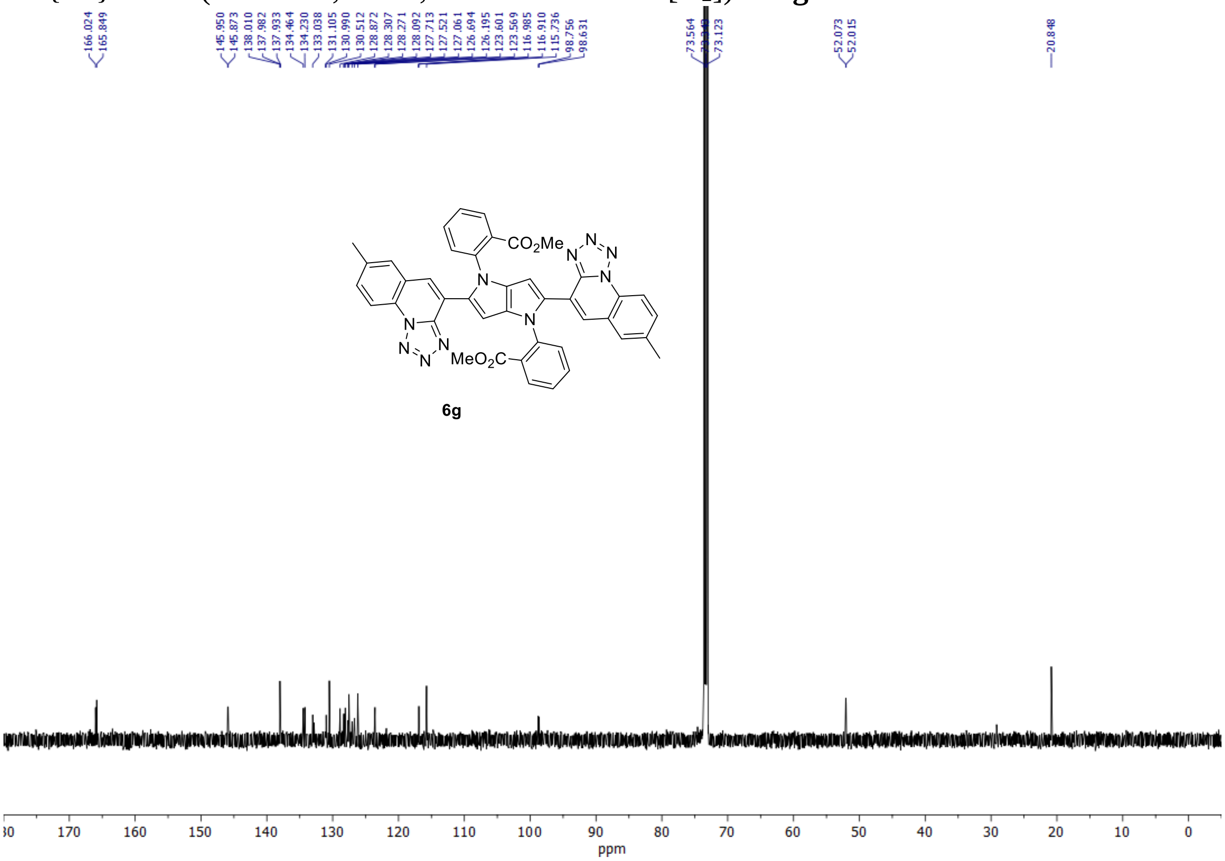

Report of HRMS of **6g**:

Single Mass Analysis

Tolerance = 3.0 mDa / DBE: min = -1.5, max = 100.0

Element prediction: Off

Number of isotope peaks used for i-FIT = 3

Monoisotopic Mass, Odd and Even Electron Ions

560 formula(e) evaluated with 3 results within limits (up to 50 closest results for each mass)

Elements Used:

C: 0-60 H: 0-100 N: 0-12 O: 0-4

Mass	Calc. Mass	mDa	PPM	DBE	Formula	i-FIT	i-FIT Norm	Fit Conf %	C	H	N	O
739.2525	739.2530	-0.5	-0.7	32.5	C42 H31 N10 O4	499.3	0.022	97.79	42	31	10	4
	739.2511	1.4	1.9	40.0	C55 H33 N O2	503.9	4.647	0.96	55	33	1	2
	739.2498	2.7	3.7	40.5	C53 H31 N4 O	503.6	4.378	1.26	53	31	4	1

Mass	Calc. Mass	mDa	PPM	DBE	Formula	i-FIT	i-FIT Norm	Fit Conf %	C	H	N	O	Na
761.2349	761.2349	0.0	0.0	32.5	C42 H30 N10 O4 Na	600.8	0.031	96.99	42	30	10	4	1
	761.2341	0.8	1.1	43.5	C55 H29 N4 O	605.7	4.864	0.77	55	29	4	1	
	761.2373	-2.4	-3.2	35.5	C44 H29 N10 O4	604.6	3.799	2.24	44	29	10	4	

^{1}H NMR (500 MHz, 300K, $CDCl_3$) of **6h**

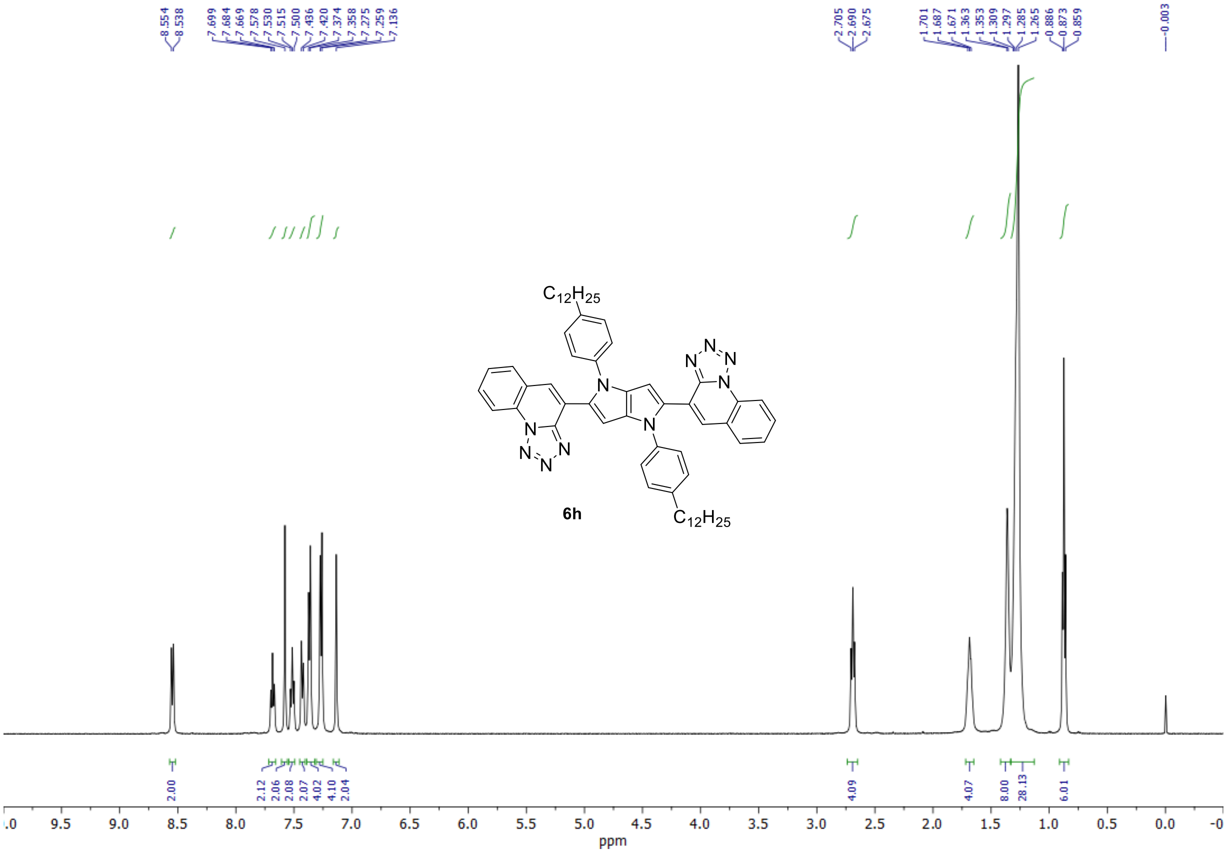

$^{13}C\{^{1}H\}$ NMR (126 MHz, 300K, $CDCl_3$) of **6h**

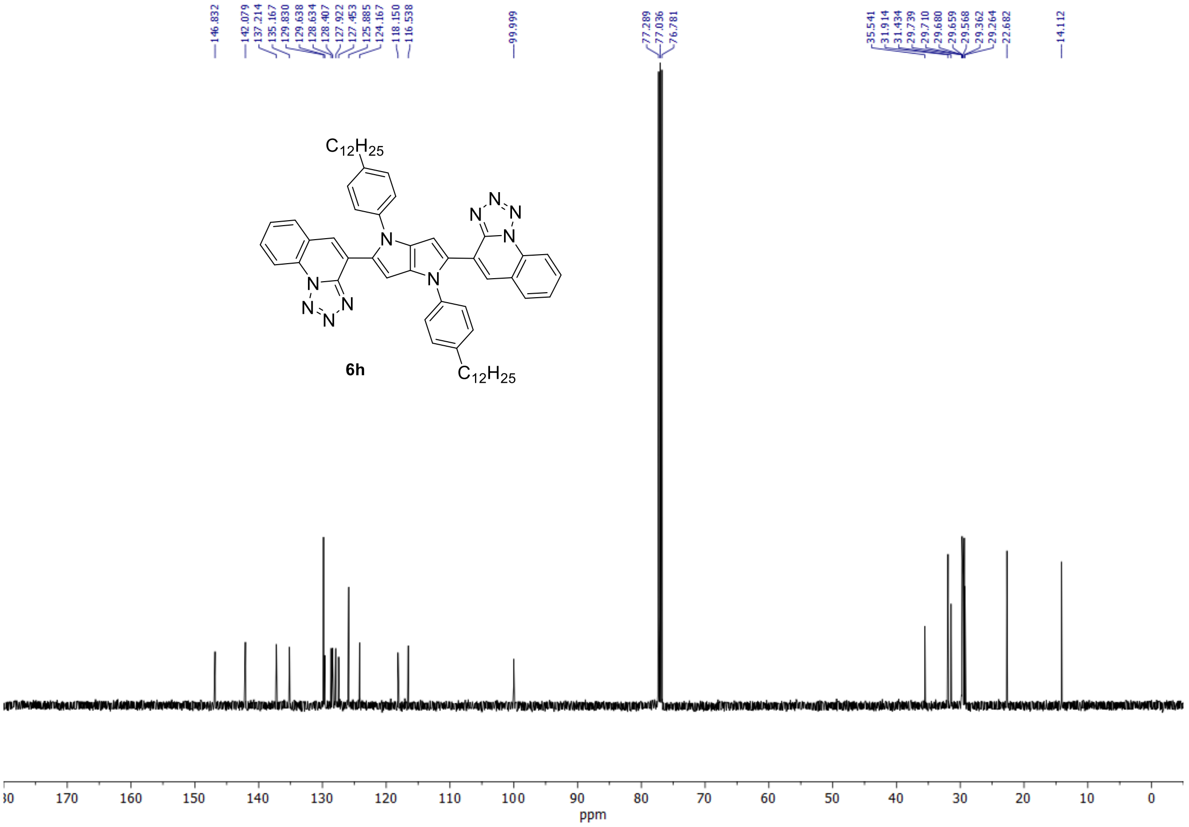

Report of HRMS of **6h**:

Single Mass Analysis
Tolerance = 3.0 mDa / DBE: min = -1.5, max = 500.0
Element prediction: Off
Number of isotope peaks used for i-FIT = 3
Monoisotopic Mass, Even Electron Ions
139 formula(e) evaluated with 1 results within limits (up to 50 closest results for each mass)
Elements Used:
C: 0-200 H: 0-200 N: 0-11

Mass	Calc. Mass	mDa	PPM	DBE	Formula	i-FIT	i-FIT Norm	Fit Conf %	C	H	N
931.5862	931.5863	-0.1	-0.1	30.5	C60 H71 N10	453.5	n/a	n/a	60	71	10

^{1}H NMR (500 MHz, 300K, $CDCl_3$) of **6i**

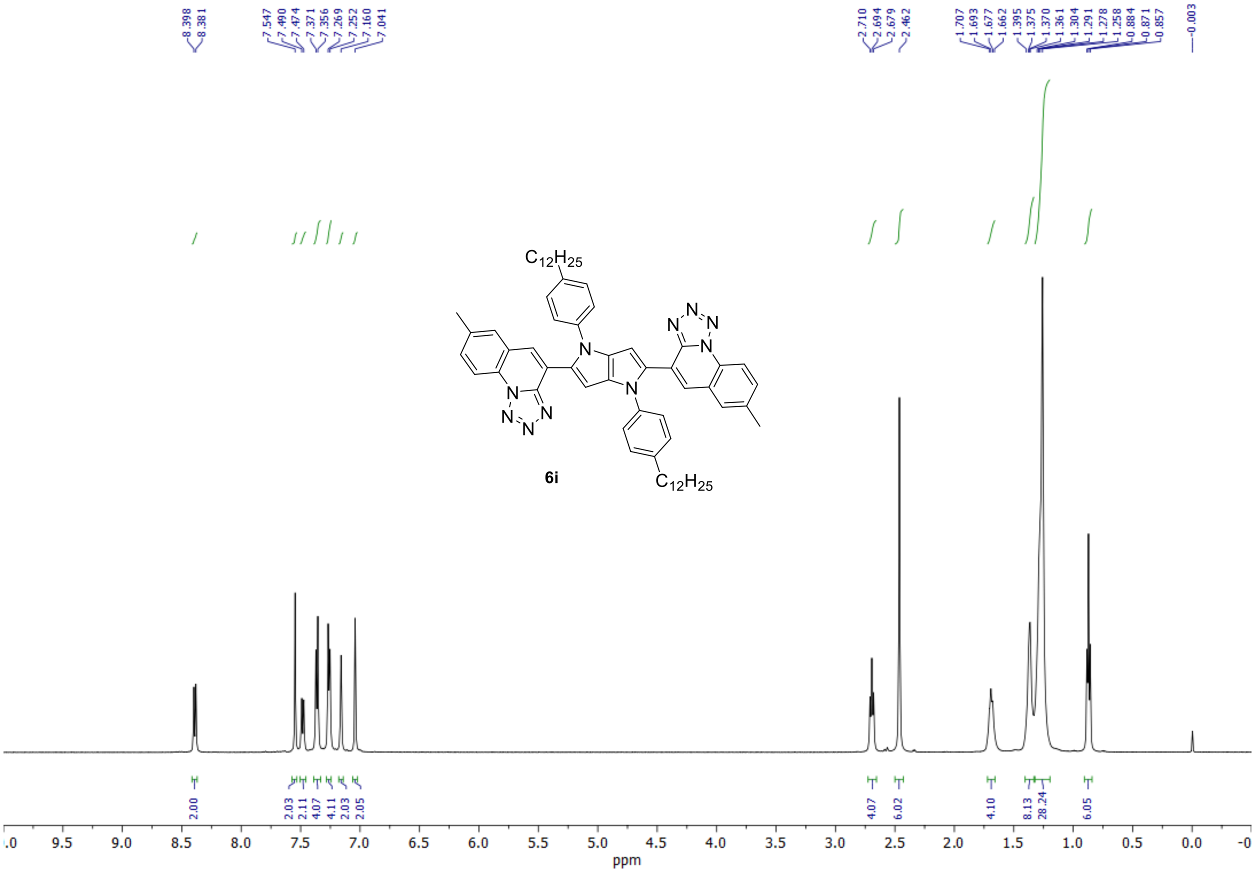

$^{13}C\{^{1}H\}$ NMR (126 MHz, 300K, $CDCl_3$) of **6i**

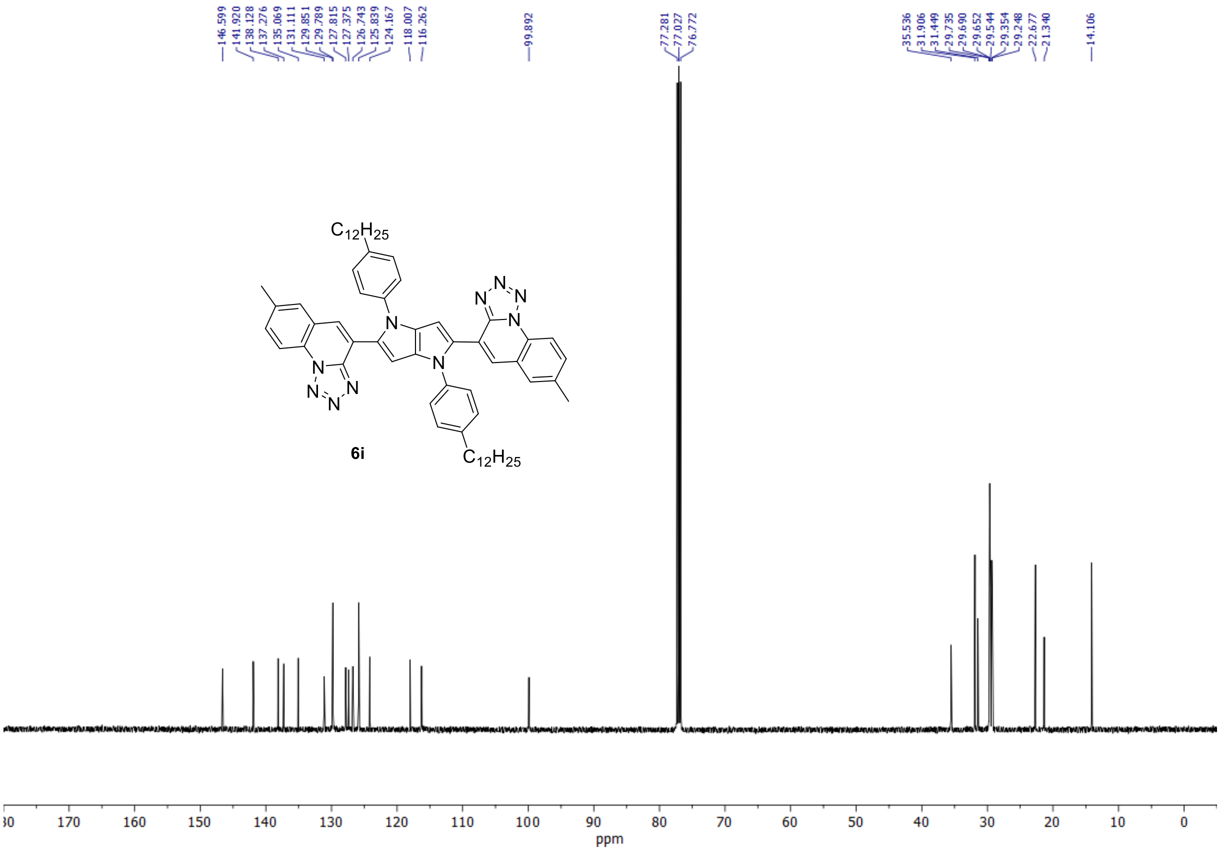

Report of HRMS of **6i**:

Single Mass Analysis
Tolerance = 3.0 mDa / DBE: min = -1.5, max = 500.0
Element prediction: Off
Number of isotope peaks used for i-FIT = 3
Monoisotopic Mass, Even Electron Ions
143 formula(e) evaluated with 1 results within limits (up to 50 closest results for each mass)
Elements Used:
C: 0-200 H: 0-200 N: 0-11

Mass	Calc. Mass	mDa	PPM	DBE	Formula	i-FIT	i-FIT Norm	Fit Conf %	C	H	N
959.6185	959.6176	0.9	0.9	30.5	C62 H75 N10	392.7	n/a	n/a	62	75	10

^{1}H NMR (600 MHz, 300K, $CDCl_3$) of **6j**

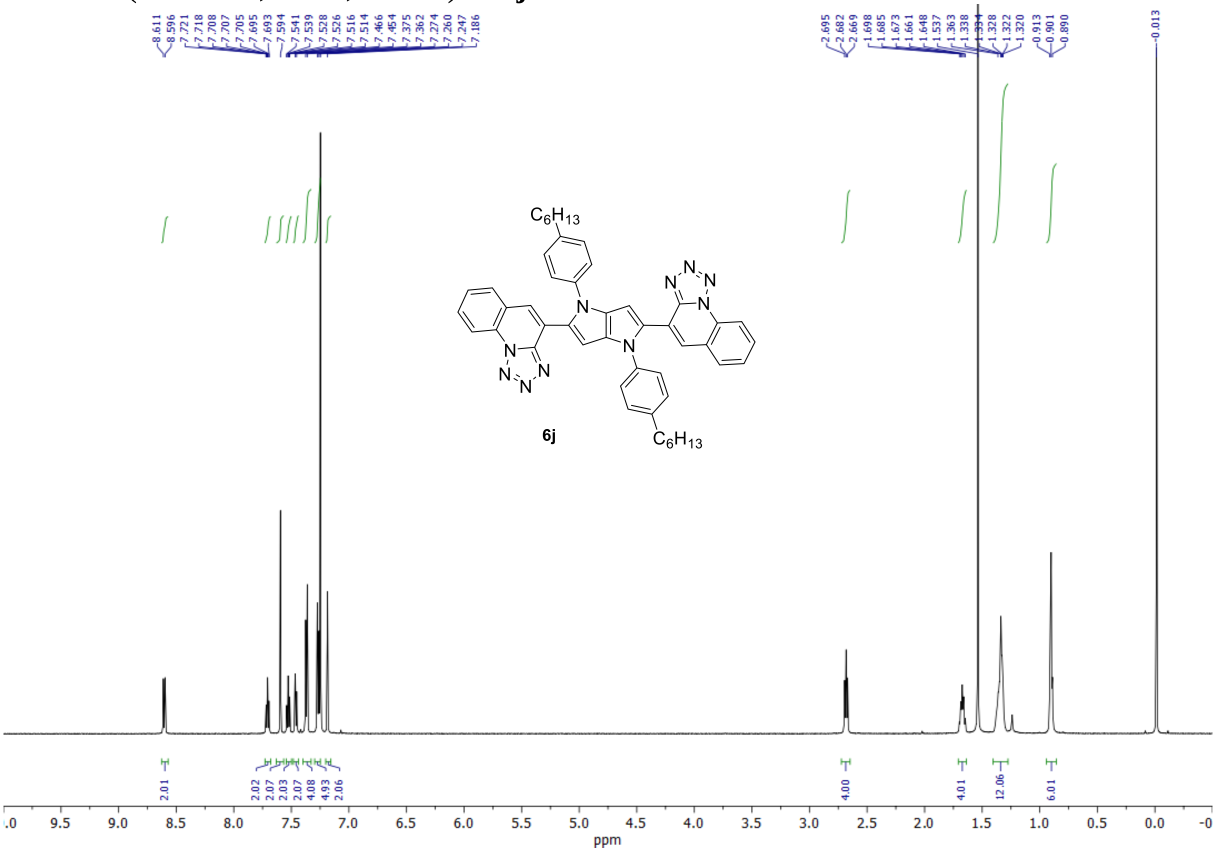

$^{13}C\{^{1}H\}$ NMR (151 MHz, 300K, $CDCl_3$) of **6j**

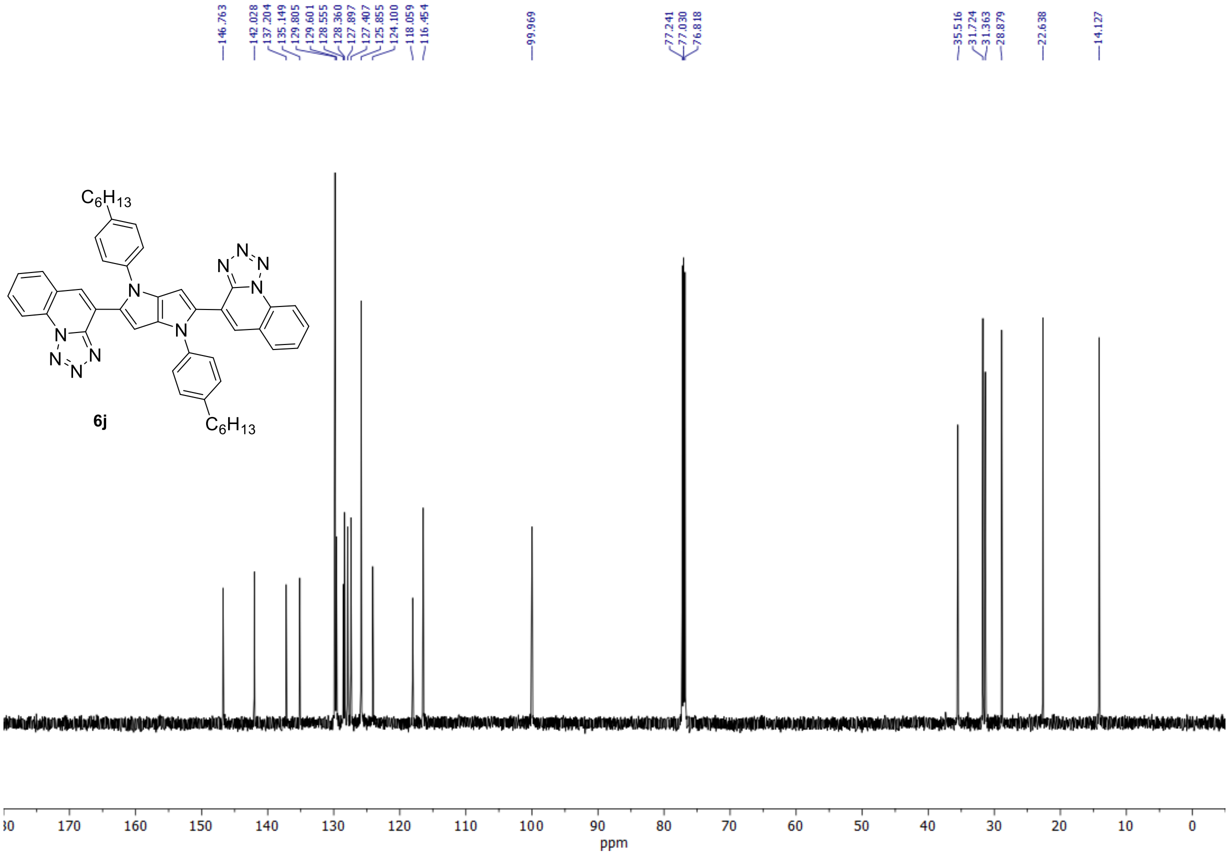

Report of HRMS of **6j**:

Single Mass Analysis
Tolerance = 3.0 mDa / DBE: min = -1.5, max = 300.0
Element prediction: Off
Number of isotope peaks used for i-FIT = 3
Monoisotopic Mass, Even Electron Ions
106 formula(e) evaluated with 1 results within limits (up to 50 closest results for each mass)
Elements Used:
C: 0-100 H: 0-200 N: 0-10

Mass	Calc. Mass	mDa	PPM	DBE	Formula	i-FIT	i-FIT Norm	Fit Conf %	C	H	N
763.3988	763.3985	0.3	0.4	30.5	C48 H47 N10	563.1	n/a	n/a	48	47	10

^{1}H NMR (600 MHz, 300K, $CDCl_3$) of **6k**

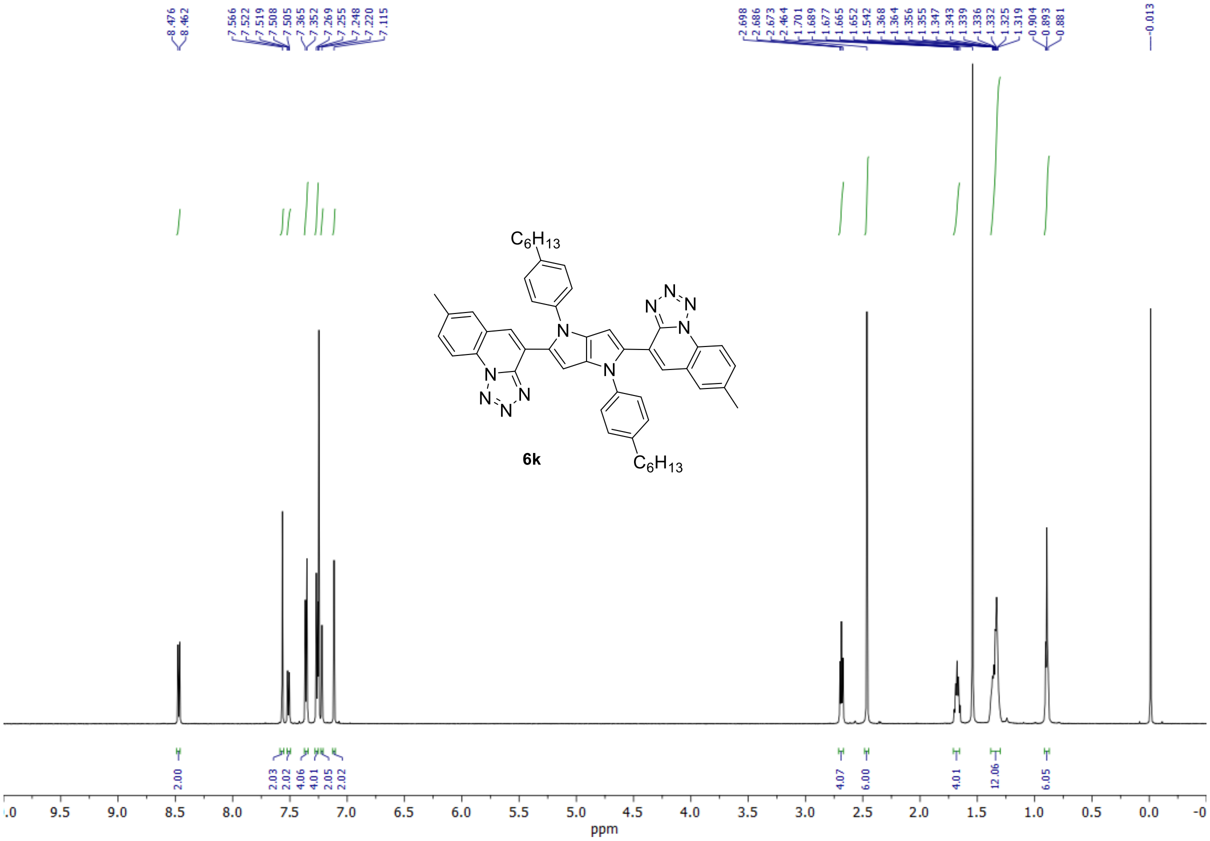

$^{13}C\{^1H\}$ NMR (151 MHz, 300K, $CDCl_3$) of **6k**

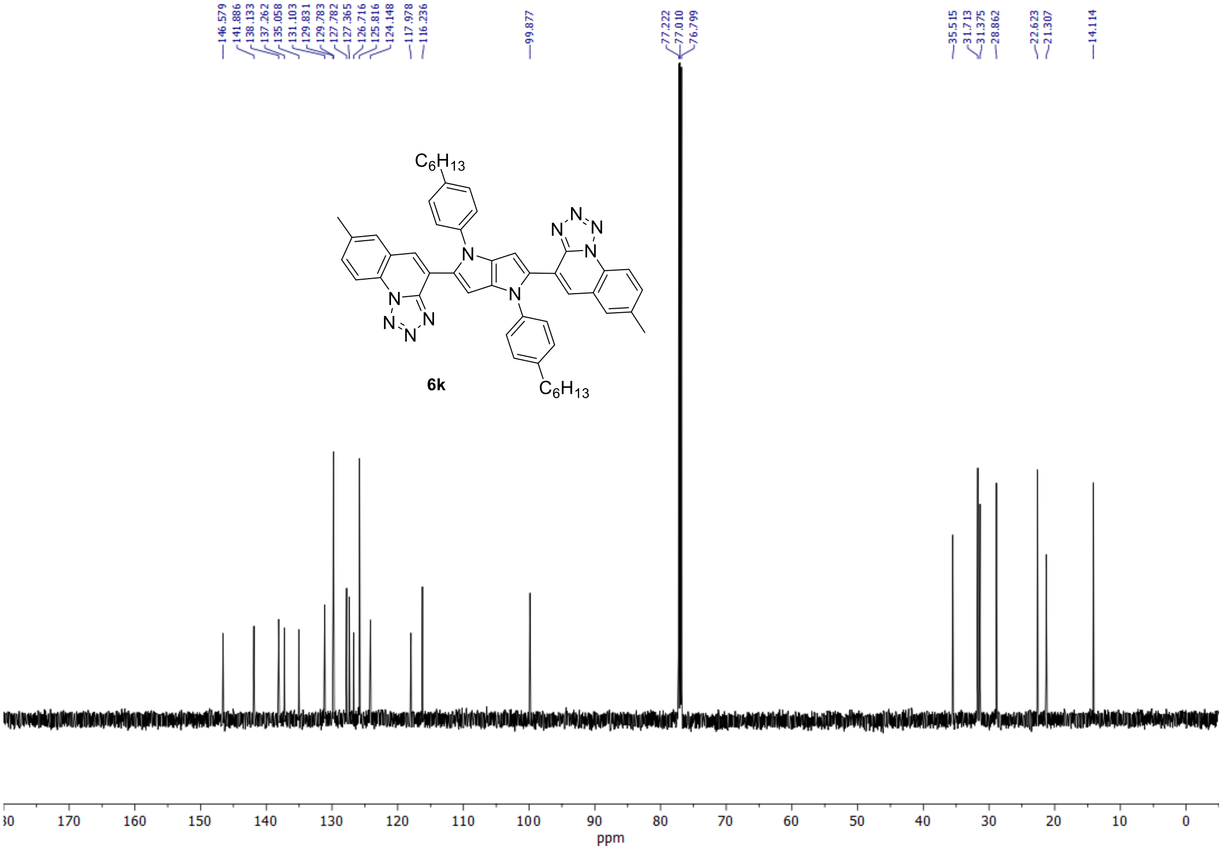

Report of HRMS of **6k**:

Single Mass Analysis

Tolerance = 3.0 mDa / DBE: min = -1.5, max = 300.0

Element prediction: Off

Number of isotope peaks used for i-FIT = 3

Monoisotopic Mass, Even Electron Ions

109 formula(e) evaluated with 1 results within limits (up to 50 closest results for each mass)

Elements Used:

C: 0-100 H: 0-200 N: 0-10

Mass	Calc. Mass	mDa	PPM	DBE	Formula	i-FIT	i-FIT Norm	Fit Conf %	C	H	N
791.4305	791.4298	0.7	0.9	30.5	C50 H51 N10	545.1	n/a	n/a	50	51	10

^{1}H NMR (600 MHz, 300K, $CDCl_3$) of **6l**

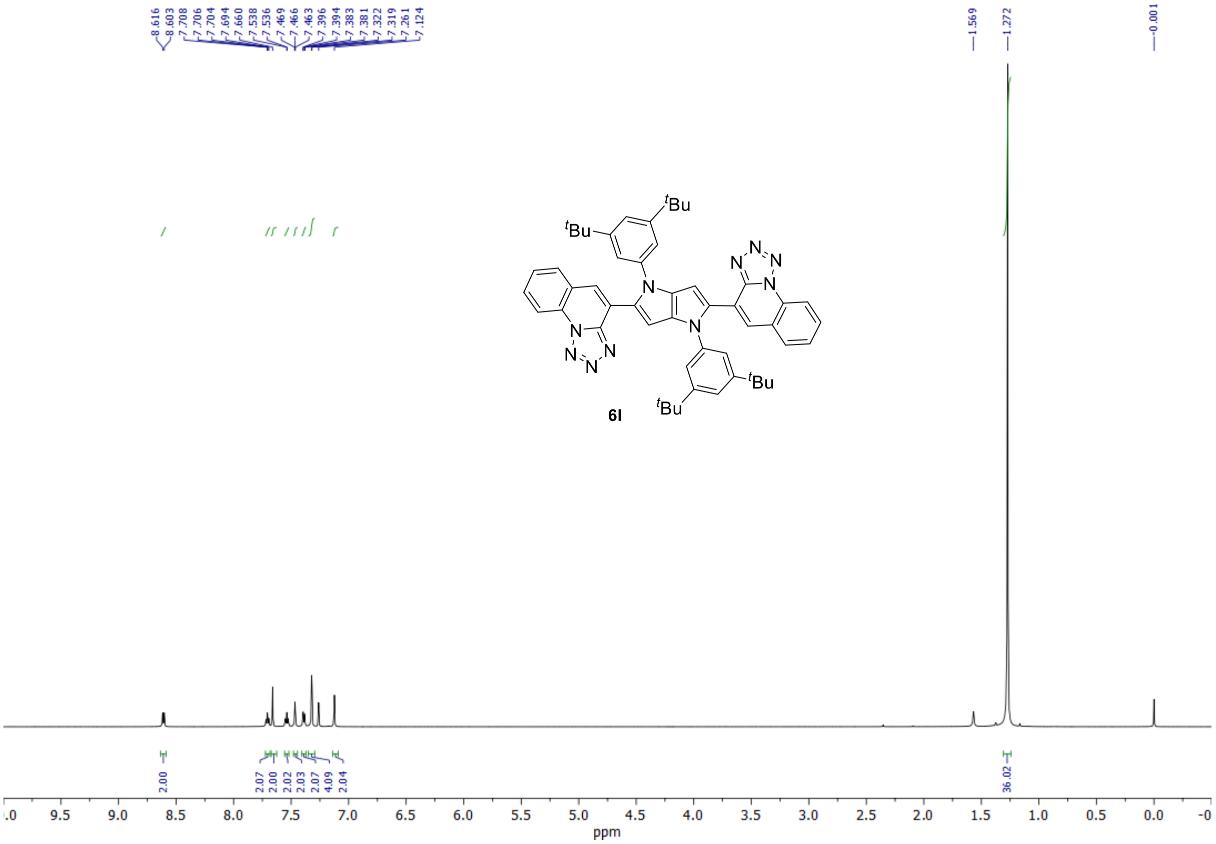

^{13}C{^{1}H} NMR (151 MHz, 300K, $CDCl_3$) of **6l**

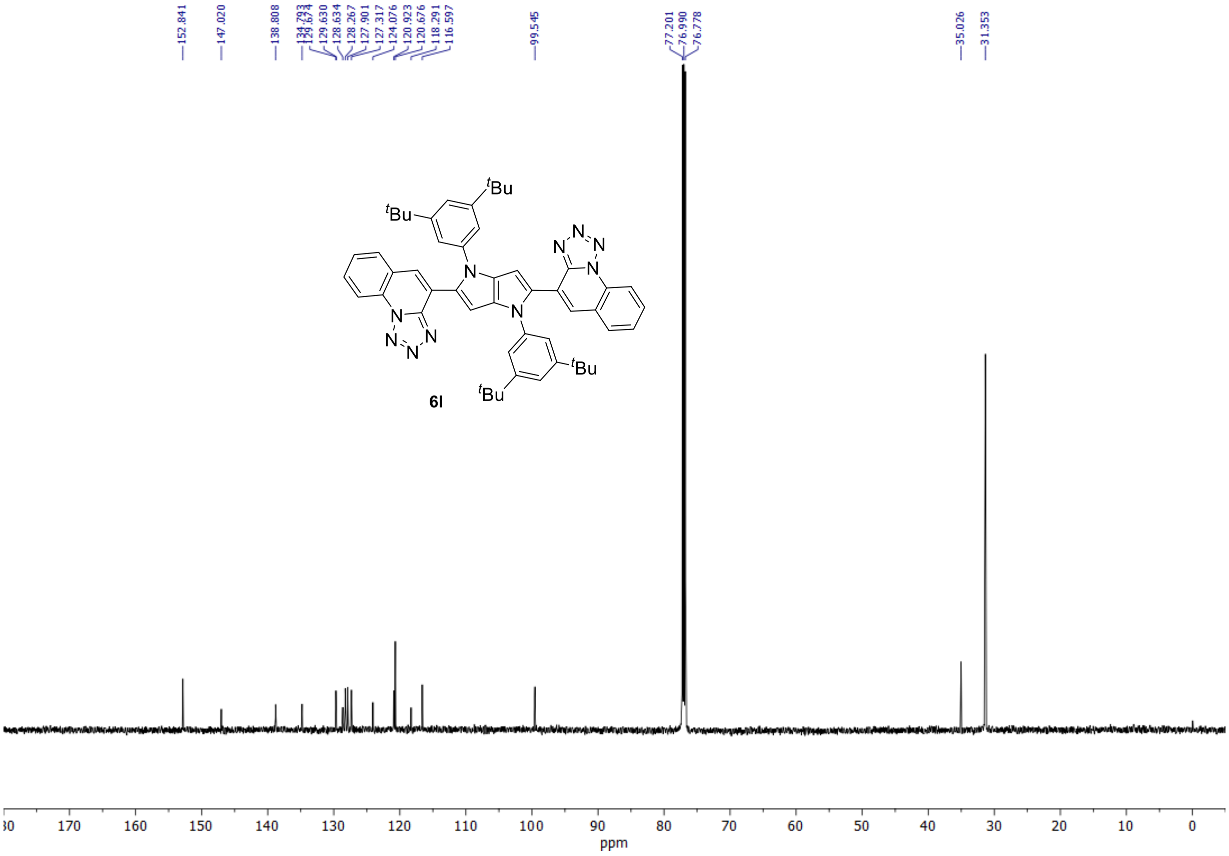

Report of HRMS of **6l**:

Single Mass Analysis
Tolerance = 3.0 mDa / DBE: min = -1.5, max = 300.0
Element prediction: Off
Number of isotope peaks used for i-FIT = 3
Monoisotopic Mass, Even Electron Ions
133 formula(e) evaluated with 1 results within limits (up to 50 closest results for each mass)
Elements Used:
C: 0-100 H: 0-200 N: 0-12

Mass	Calc. Mass	mDa	PPM	DBE	Formula	i-FIT	i-FIT Norm	Fit Conf %	C	H	N
819.4608	819.4611	-0.3	-0.4	30.5	C52 H55 N10	619.9	n/a	n/a	52	55	10

^{1}H NMR (600 MHz, 300K, $CDCl_3$) of **6m**

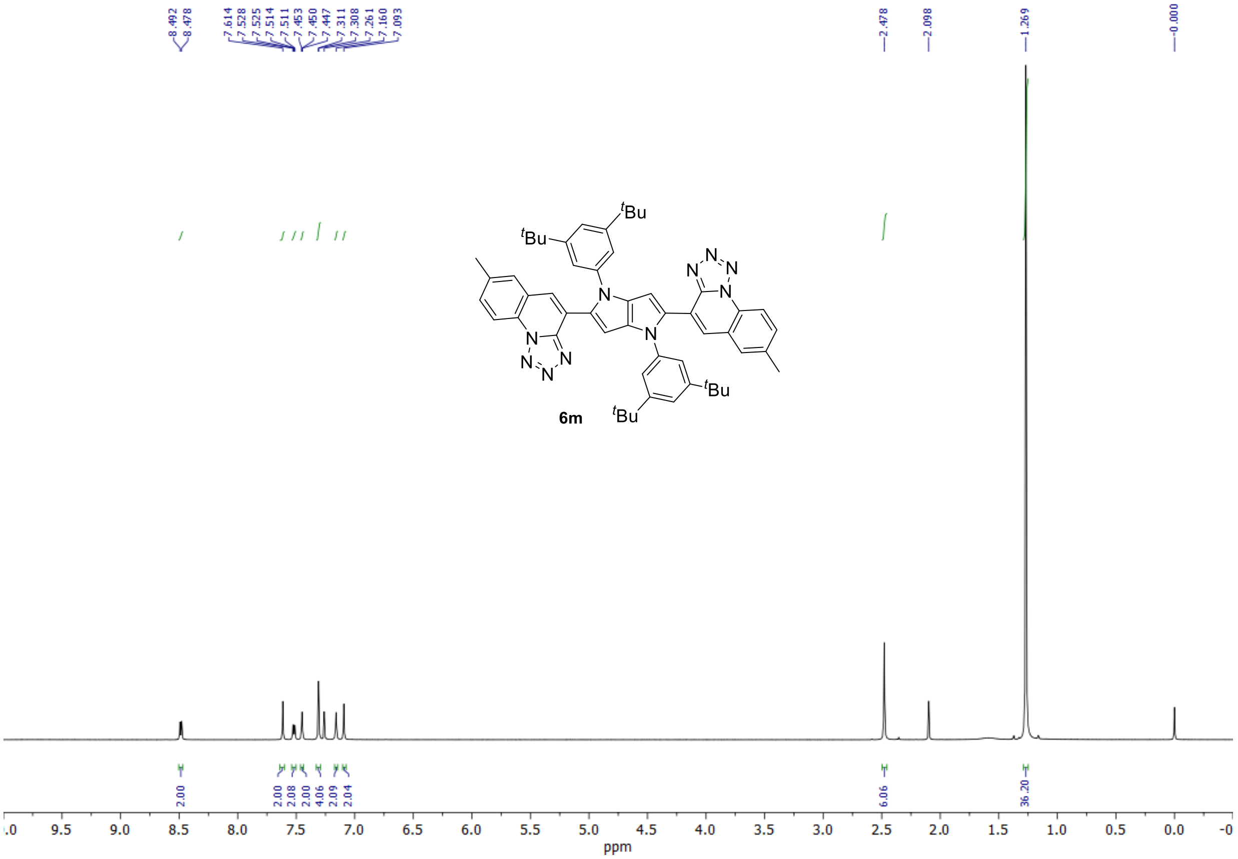

$^{13}C\{^{1}H\}$ NMR (151 MHz, 300K, $CDCl_3$) of **6m**

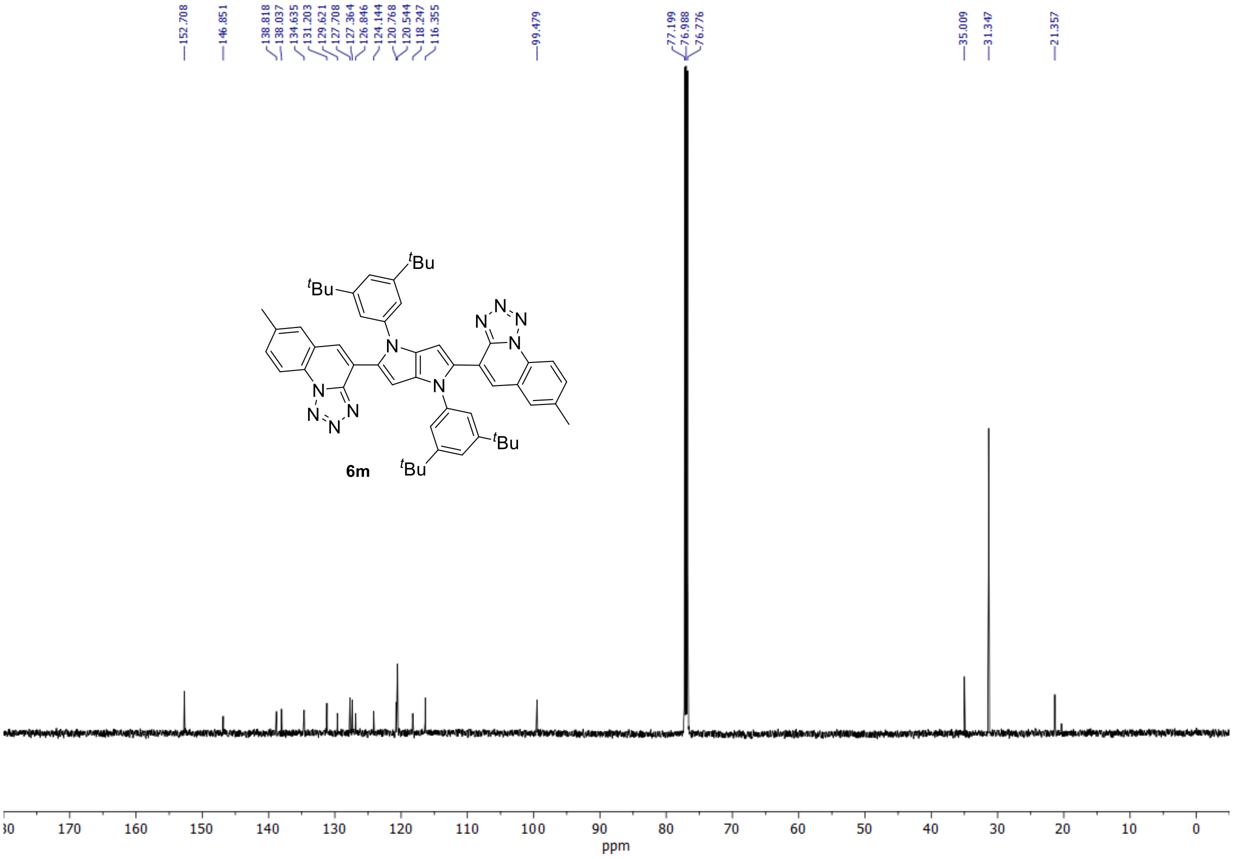

Report of HRMS of **6m**:

Single Mass Analysis

Tolerance = 3.0 mDa / DBE: min = -1.5, max = 300.0

Element prediction: Off

Number of isotope peaks used for i-FIT = 3

Monoisotopic Mass, Even Electron Ions

137 formula(e) evaluated with 1 results within limits (up to 50 closest results for each mass)

Elements Used:

C: 0-100 H: 0-200 N: 0-12

Mass	Calc. Mass	mDa	PPM	DBE	Formula	i-FIT	i-FIT Norm	Fit Conf %	C	H	N
847.4923	847.4924	-0.1	-0.1	30.5	C54 H59 N10	495.8	n/a	n/a	54	59	10

^{1}H NMR (500 MHz, 300K, $CDCl_3$) of **6n**

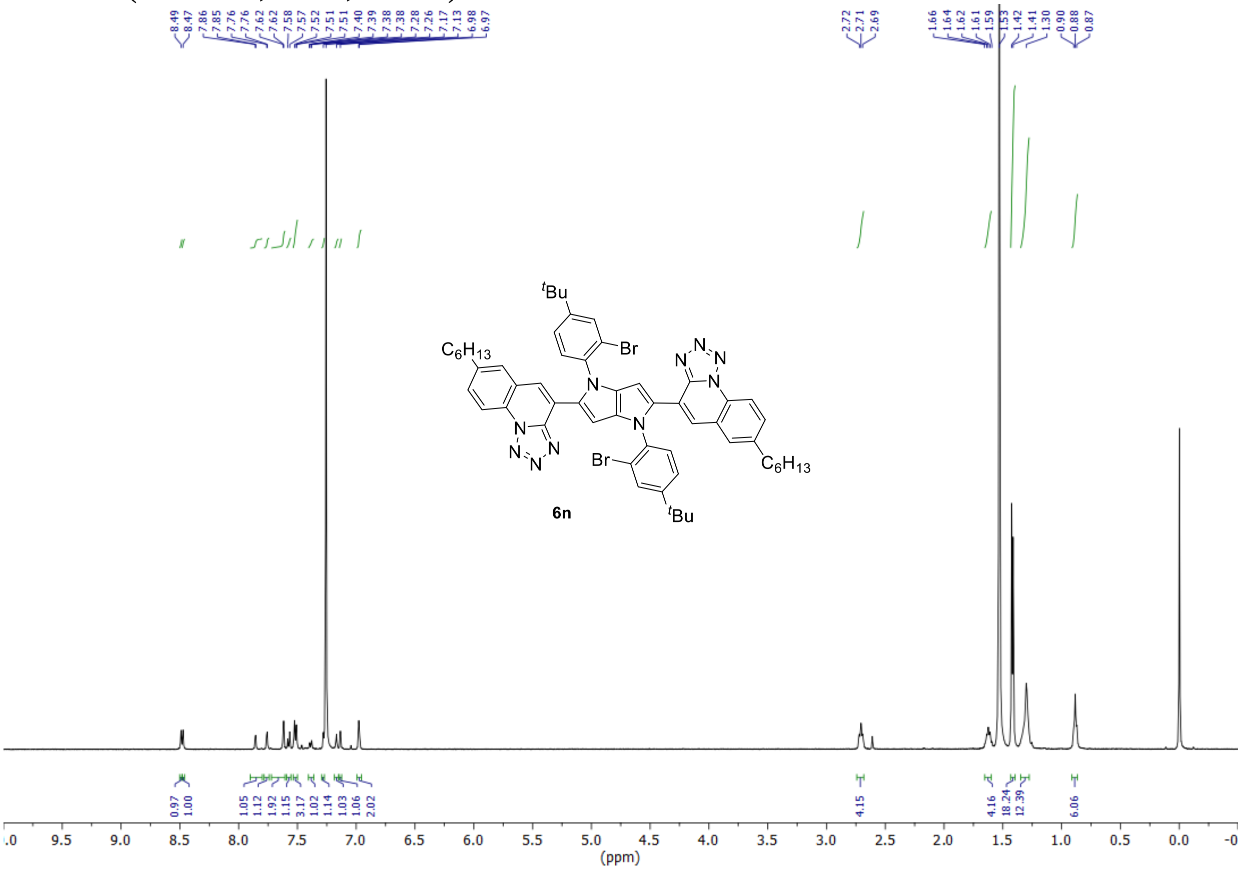

$^{13}C\{^{1}H\}$ NMR (126 MHz, 300K, $CDCl_3$) of **6n**

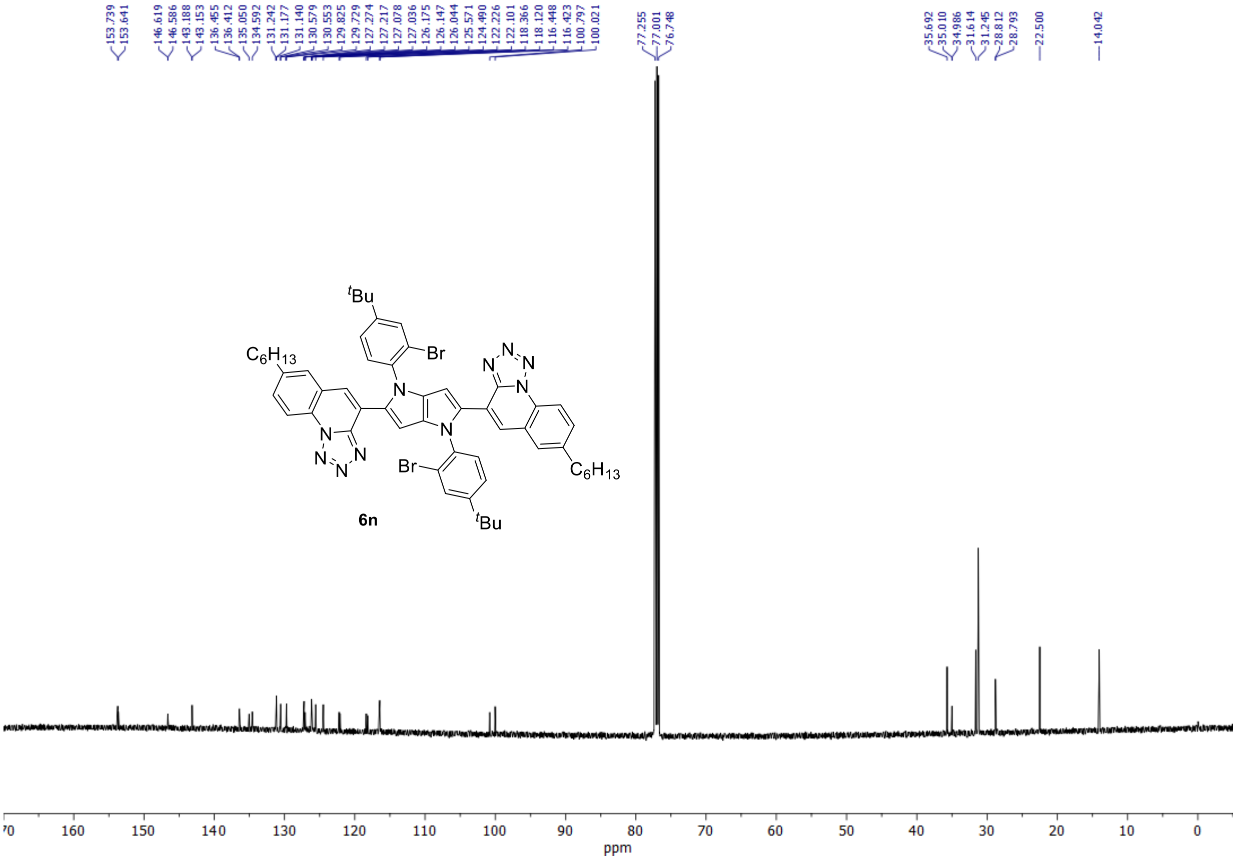

Report of HRMS of **6n**:

Single Mass Analysis
Tolerance = 3.0 mDa / DBE: min = -1.5, max = 150.0
Element prediction: Off
Number of isotope peaks used for i-FIT = 3
Monoisotopic Mass, Even Electron Ions
119 formula(e) evaluated with 1 results within limits (up to 200 closest results for each mass)
Elements Used:
C: 0-200 H: 0-200 N: 2-11 Br: 2-2

Mass	Calc. Mass	mDa	PPM	DBE	Formula	i-FIT	i-FIT Norm	Fit Conf %	C	H	N	Br
1031.3451	1031.3442	0.9	0.9	30.5	C56 H61 N10 Br2	247.7	n/a	n/a	56	61	10	2

^{1}H NMR (500 MHz, 300K, $CDCl_3$) of **6o**

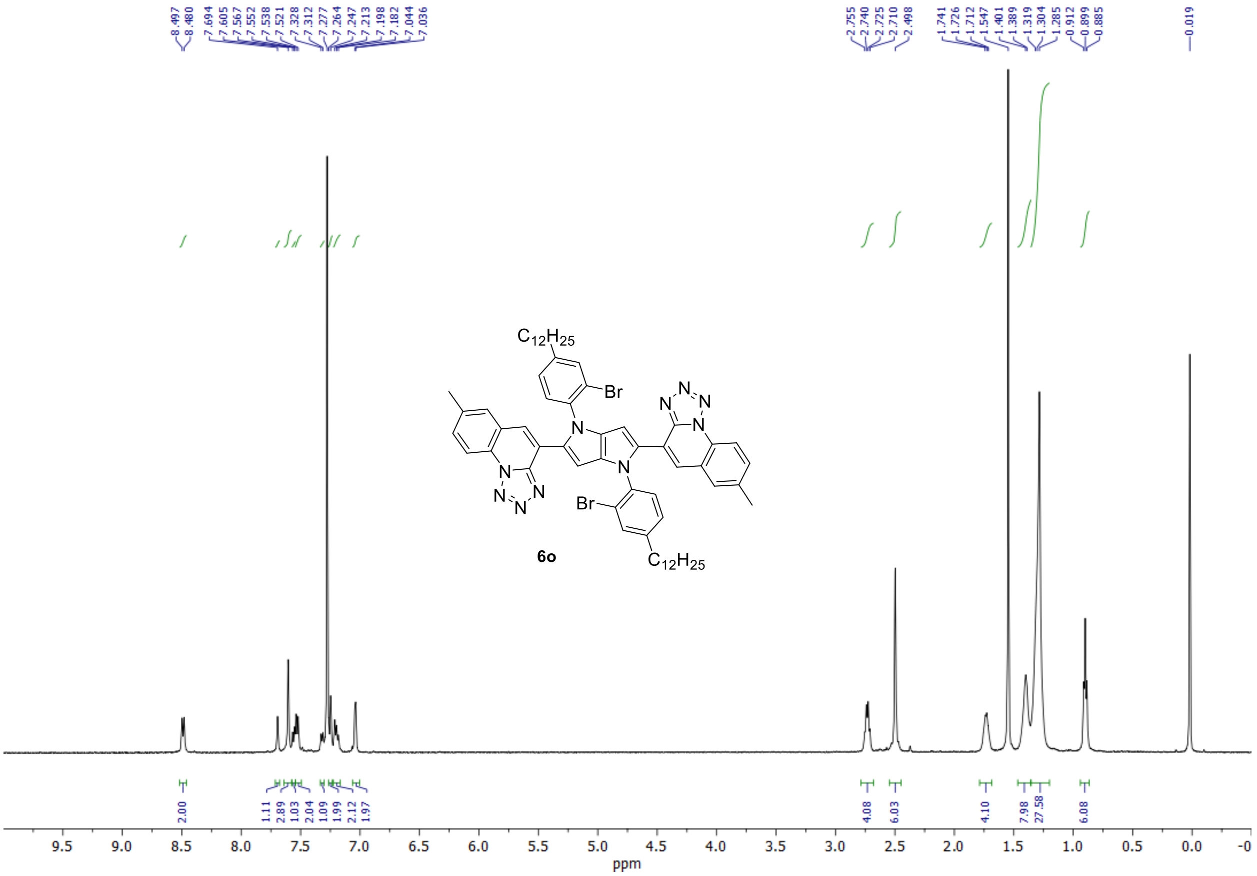

$^{13}C\{^{1}H\}$ NMR (126 MHz, 300K, $CDCl_3$) of **6o**

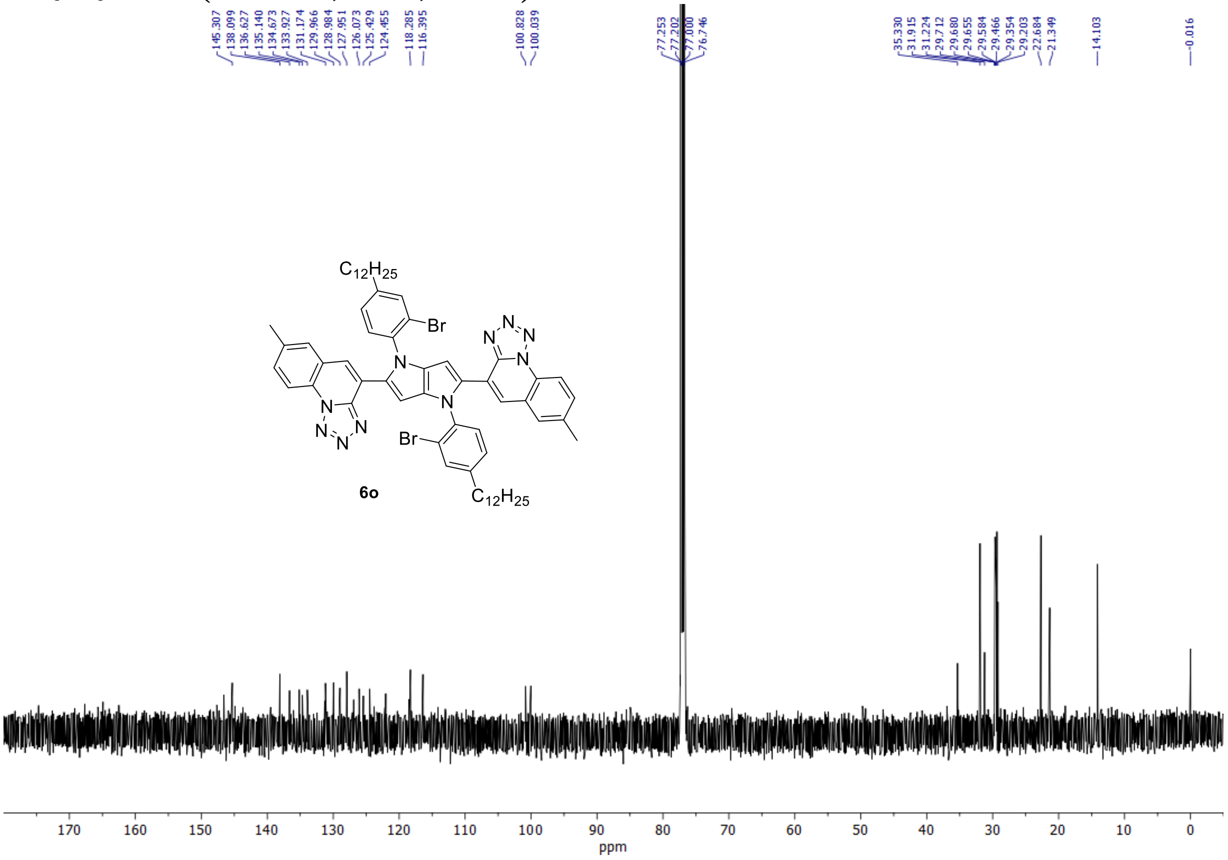

Report of HRMS of **6o**:

Single Mass Analysis
Tolerance = 10.0 mDa / DBE: min = -1.5, max = 150.0
Element prediction: Off
Number of isotope peaks used for i-FIT = 3
Monoisotopic Mass, Even Electron Ions
269 formula(e) evaluated with 3 results within limits (up to 50 closest results for each mass)

Elements Used:
C: 0-100 H: 0-200 N: 2-12 Br: 1-2

Mass	Calc. Mass	mDa	PPM	DBE	Formula	i-FIT	i-FIT Norm	Fit Conf %	C	H	N	Br
1115.4421	1115.4454	-3.3	-3.0	33.5	C71 H77 N2 Br2	278.4	1.538	21.48	71	77	2	2
	1115.4386	3.5	3.1	30.5	C62 H73 N10 Br2	277.1	0.243	78.39	62	73	10	2
	1115.4376	4.5	4.0	45.5	C74 H64 N6 Br	283.6	6.685	0.12	74	64	6	1

^{1}H NMR (500 MHz, 300K, $CDCl_3$) of **6p**

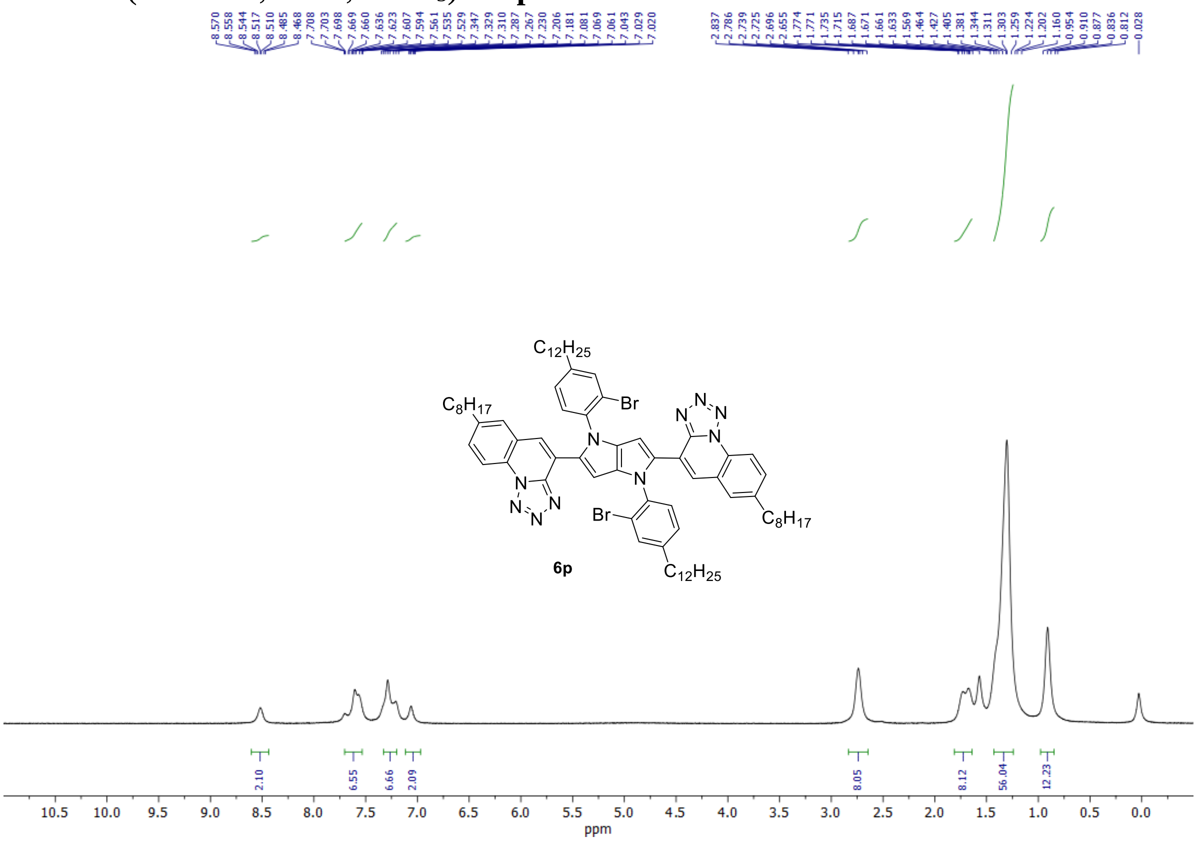

$^{13}C\{^{1}H\}$ NMR (126 MHz, 300K, $CDCl_3$) of **6p**

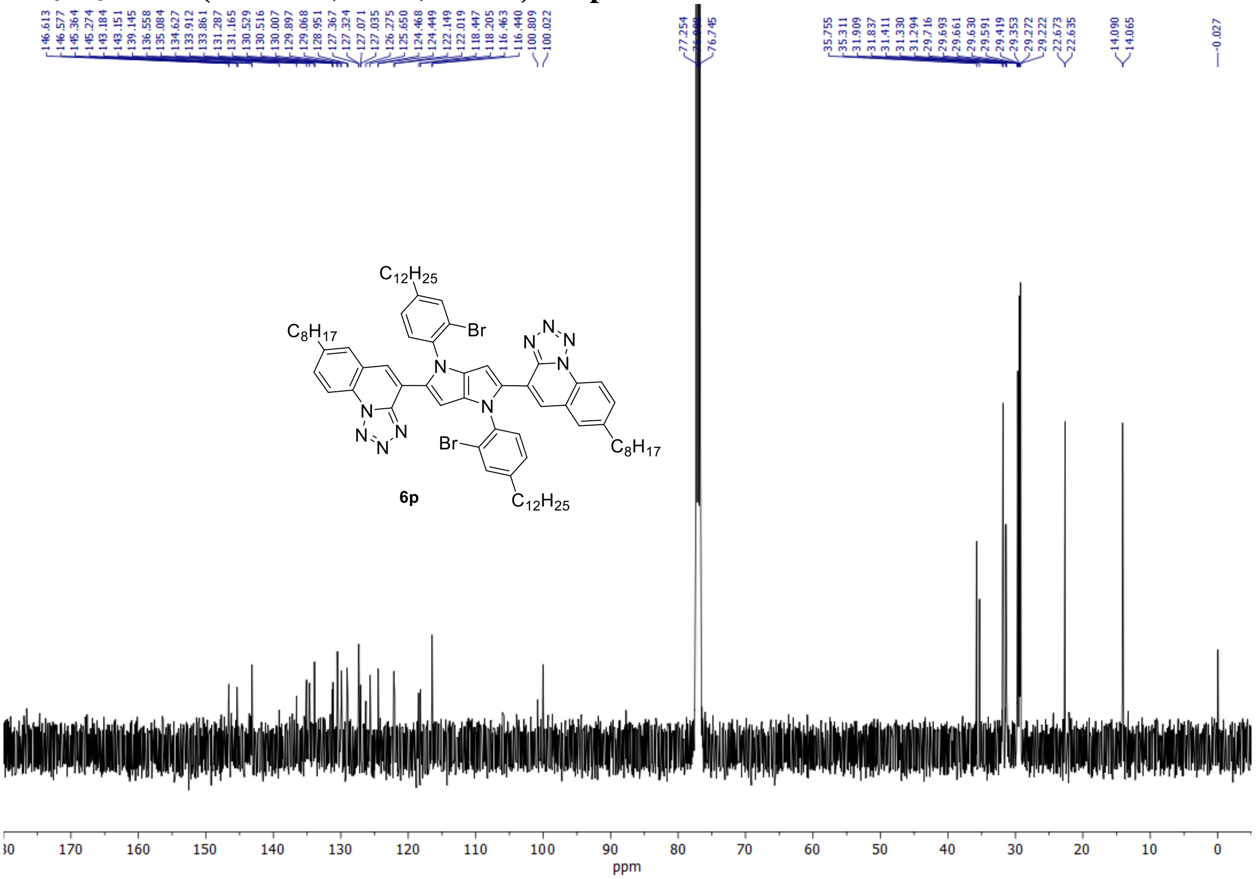

Report of HRMS of **6p**:

Single Mass Analysis
Tolerance = 3.0 mDa / DBE: min = -1.5, max = 150.0
Element prediction: Off
Number of isotope peaks used for i-FIT = 3
Monoisotopic Mass, Even Electron Ions
139 formula(e) evaluated with 1 results within limits (up to 200 closest results for each mass)
Elements Used:
C: 0-200 H: 0-200 N: 2-11 Br: 2-2

Mass	Calc. Mass	mDa	PPM	DBE	Formula	i-FIT	i-FIT Norm	Fit Conf %	C	H	N	Br
1311.6583	1311.6577	0.6	0.5	30.5	C76 H101 N10 Br2	244.9	n/a	n/a	76	101	10	2

^{1}H NMR (500 MHz, 300K, $CDCl_3$) of **6q**

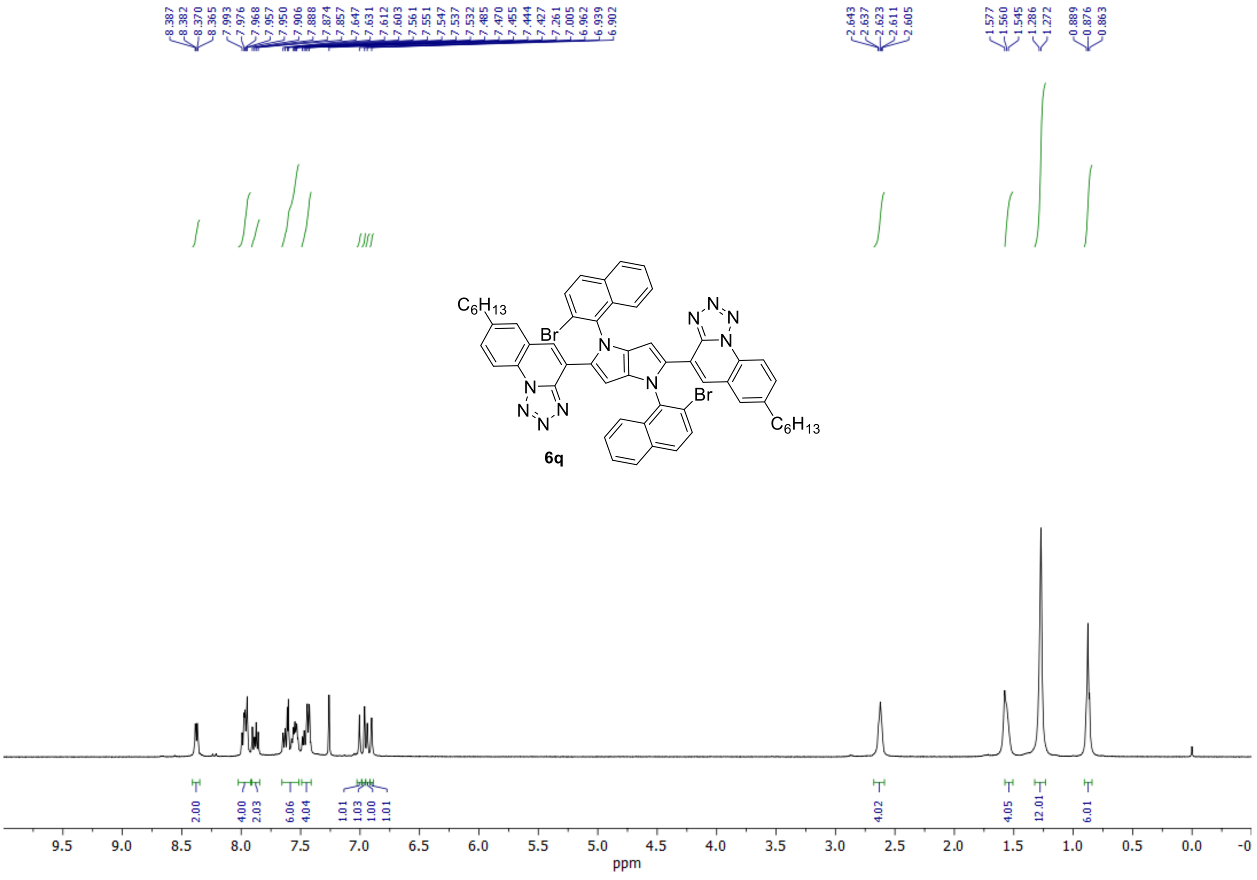

$^{13}C\{^{1}H\}$ NMR (126 MHz, 300K, $CDCl_3$) of **6q**

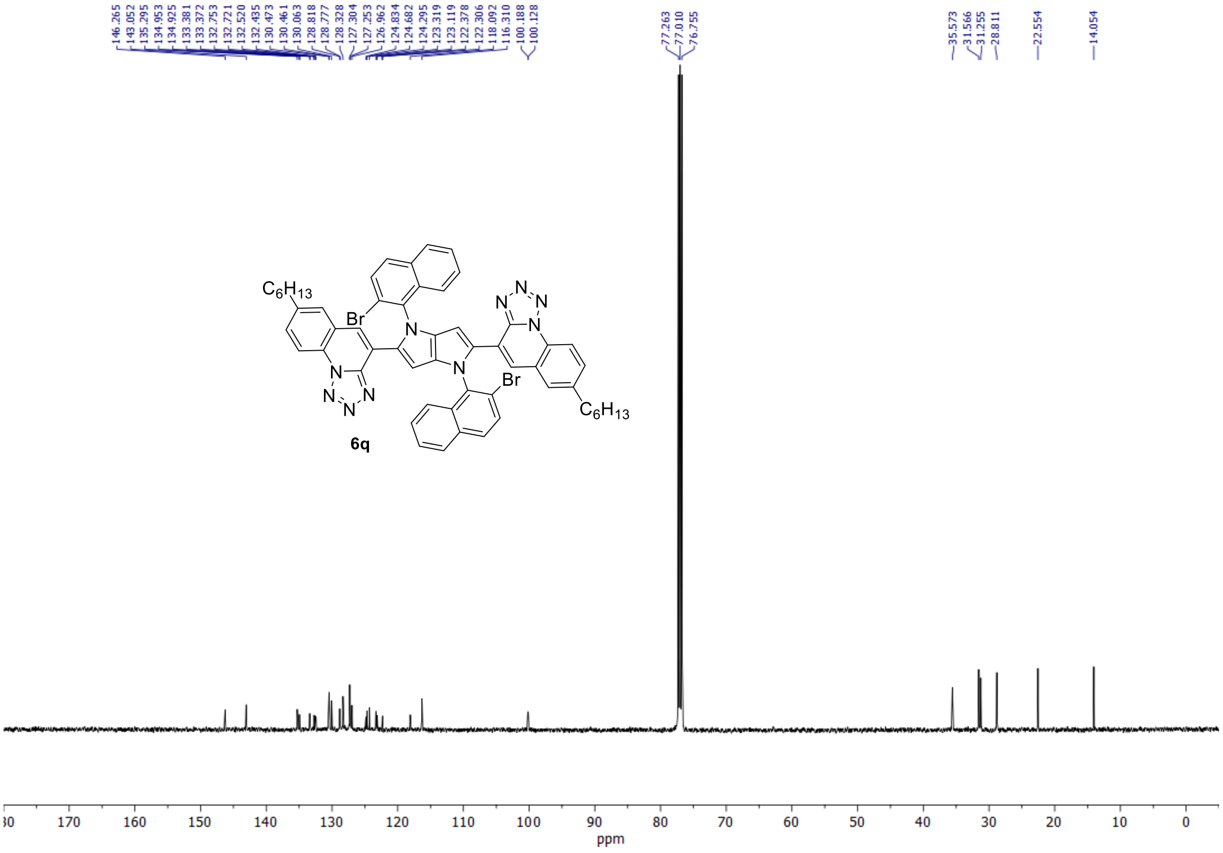

Report of HRMS of **6q**:

Single Mass Analysis

Tolerance = 3.0 mDa / DBE: min = -1.5, max = 150.0

Element prediction: Off

Number of isotope peaks used for i-FIT = 3

Monoisotopic Mass, Even Electron Ions

83 formula(e) evaluated with 1 results within limits (up to 200 closest results for each mass)

Elements Used:

C: 0-200 H: 0-200 N: 4-11 Br: 2-2

Mass	Calc. Mass	mDa	PPM	DBE	Formula	i-FIT	i-FIT Norm	Fit Conf %	C	H	N	Br
1019.2520	1019.2508	1.2	1.2	36.5	C56 H49 N10 Br2	354.8	n/a	n/a	56	49	10	2

^{1}H NMR (500 MHz, 300K, $CDCl_3$) of **6r**

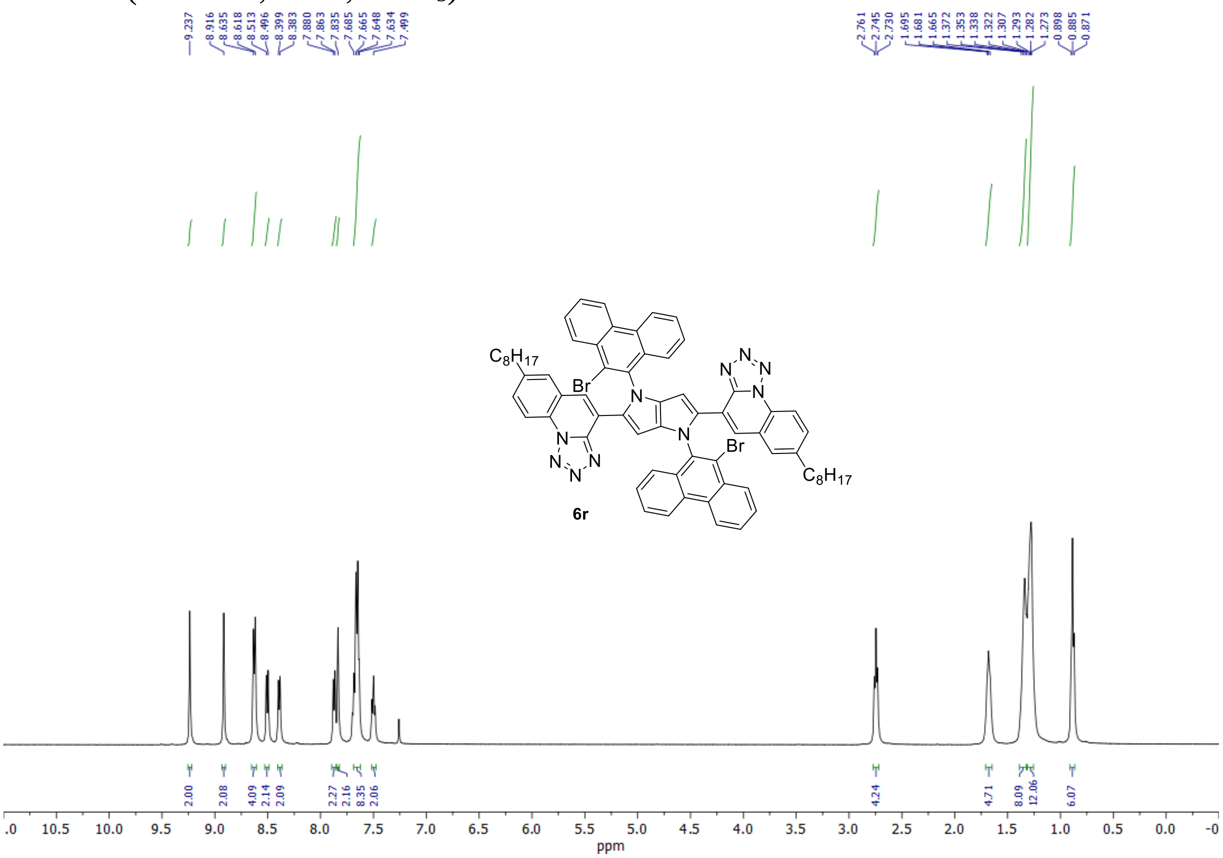

$^{13}C\{^{1}H\}$ NMR (126 MHz, 300K, $CDCl_3$) of **6r**

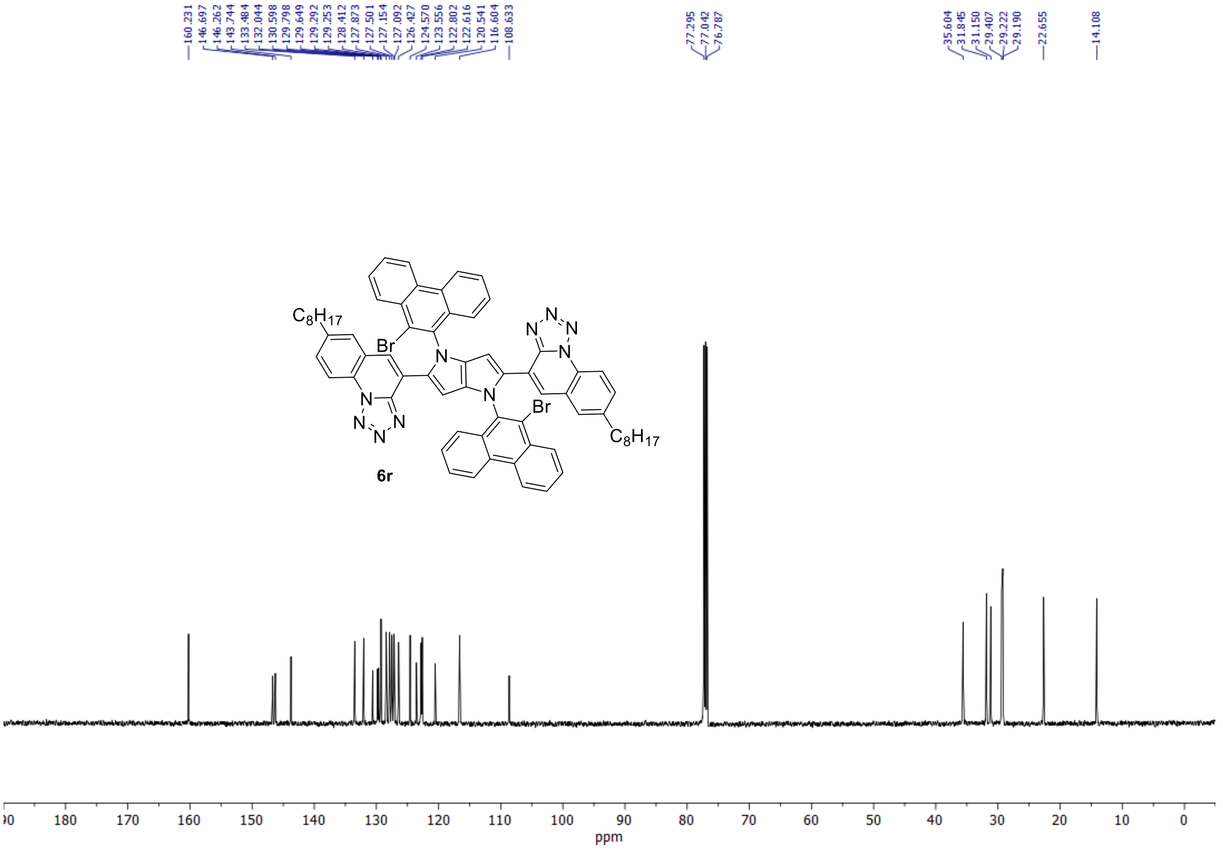

Report of HRMS of **6r**:

Single Mass Analysis
Tolerance = 3.0 mDa / DBE: min = -1.5, max = 150.0
Element prediction: Off
Number of isotope peaks used for i-FIT = 3
Monoisotopic Mass, Even Electron Ions
88 formula(e) evaluated with 1 results within limits (up to 200 closest results for each mass)
Elements Used:
C: 0-200 H: 0-200 N: 4-11 Br: 2-2

Mass	Calc. Mass	mDa	PPM	DBE	Formula	i-FIT	i-FIT Norm	Fit Conf %	C	H	N	Br
1175.3443	1175.3442	0.1	0.1	42.5	C68 H61 N10 Br2	354.8	n/a	n/a	68	61	10	2

^{1}H NMR (500 MHz, 300K, C_6D_6) of **10a**

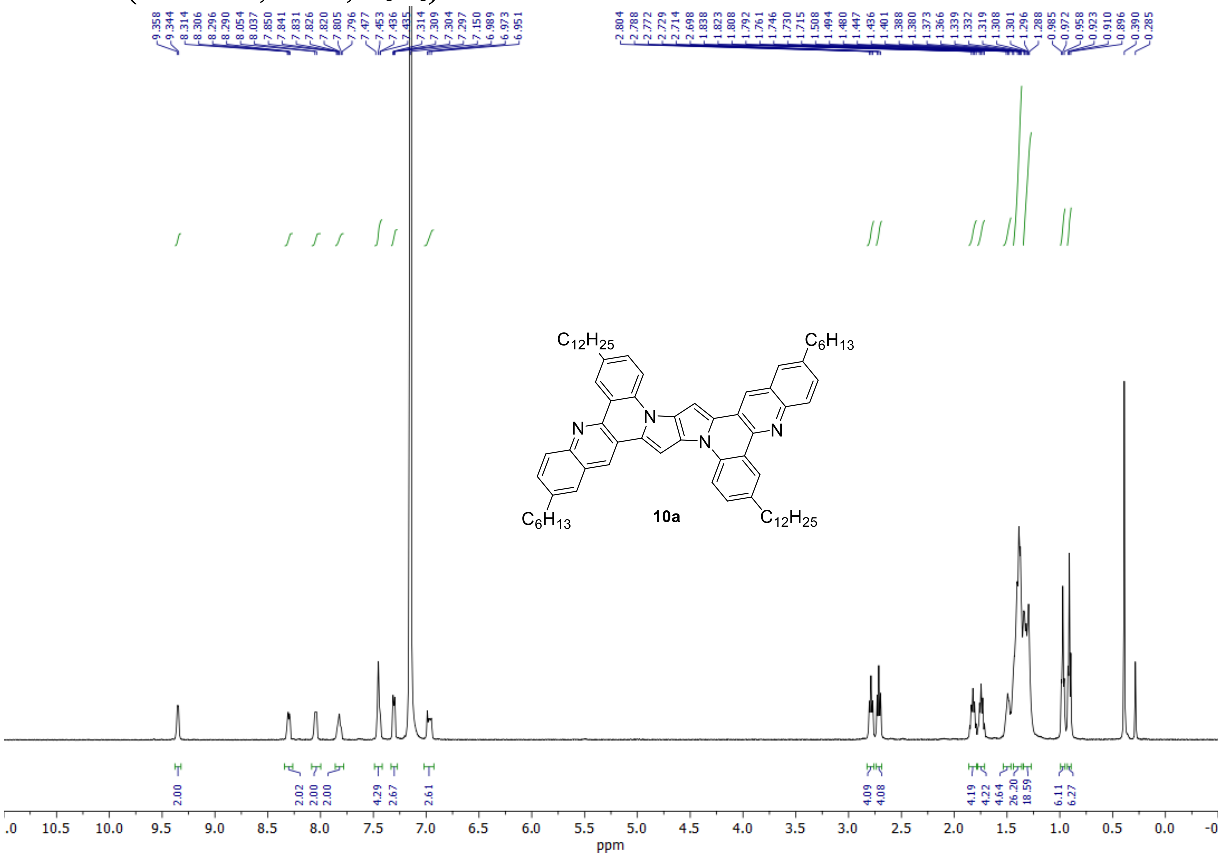

$^{13}C\{^{1}H\}$ NMR (126 MHz, 350K, tetrachloroethane-[D_2]) of **10a**

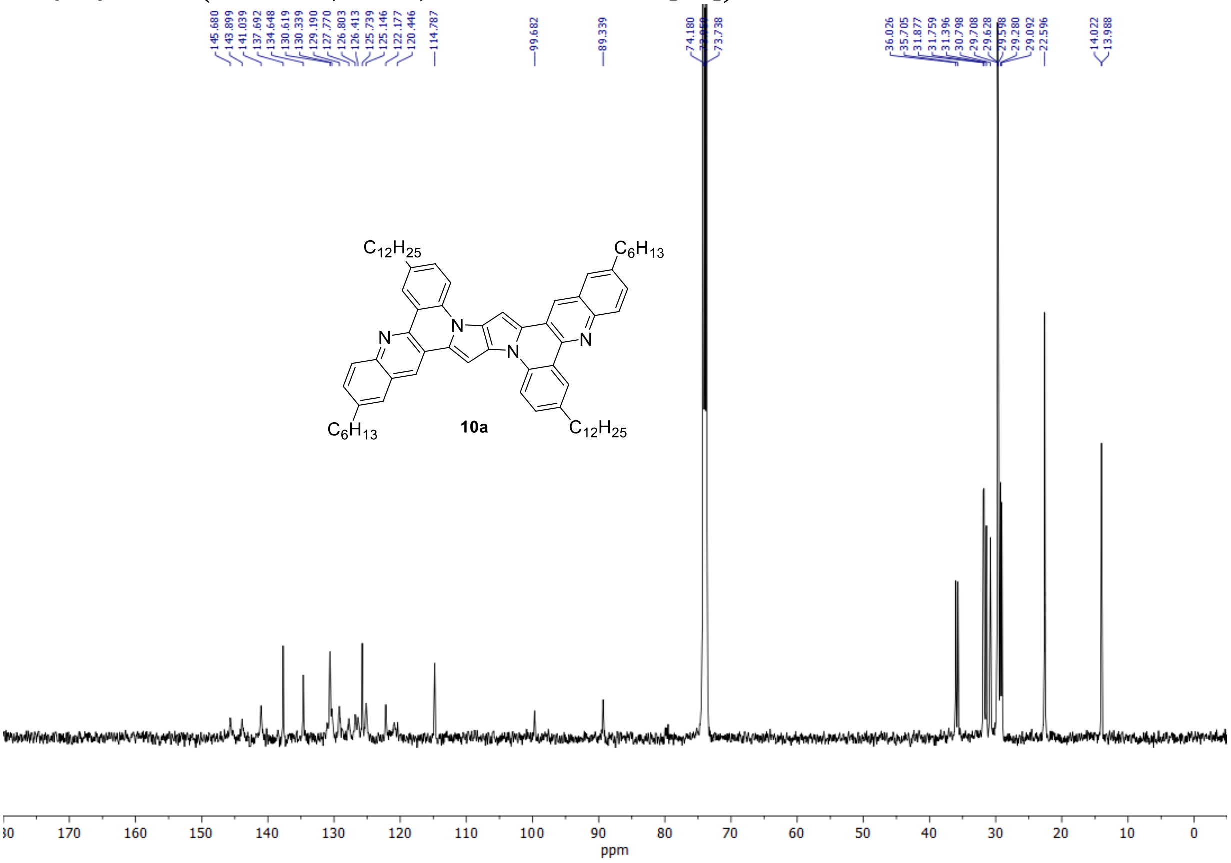

Report of HRMS of **10a**:

Single Mass Analysis

Tolerance = 3.0 mDa / DBE: min = -1.5, max = 50.0

Element prediction: Off

Number of isotope peaks used for i-FIT = 3

Monoisotopic Mass, Even Electron Ions

447 formula(e) evaluated with 1 results within limits (up to 50 best isotopic matches for each mass)

Elements Used:

C: 0-100 H: 0-200 N: 0-5 O: 0-5

Mass	Calc. Mass	mDa	PPM	DBE	Formula	i-FIT	i-FIT Norm	Fit Conf %	C	H	N	O
1013.7426	1013.7400	2.6	2.6	28.5	C72 H93 N4	291.9	n/a	n/a	72	93	4	

^{1}H NMR (500 MHz, 350K, tetrachloroethane-[D_2]) of **10b**

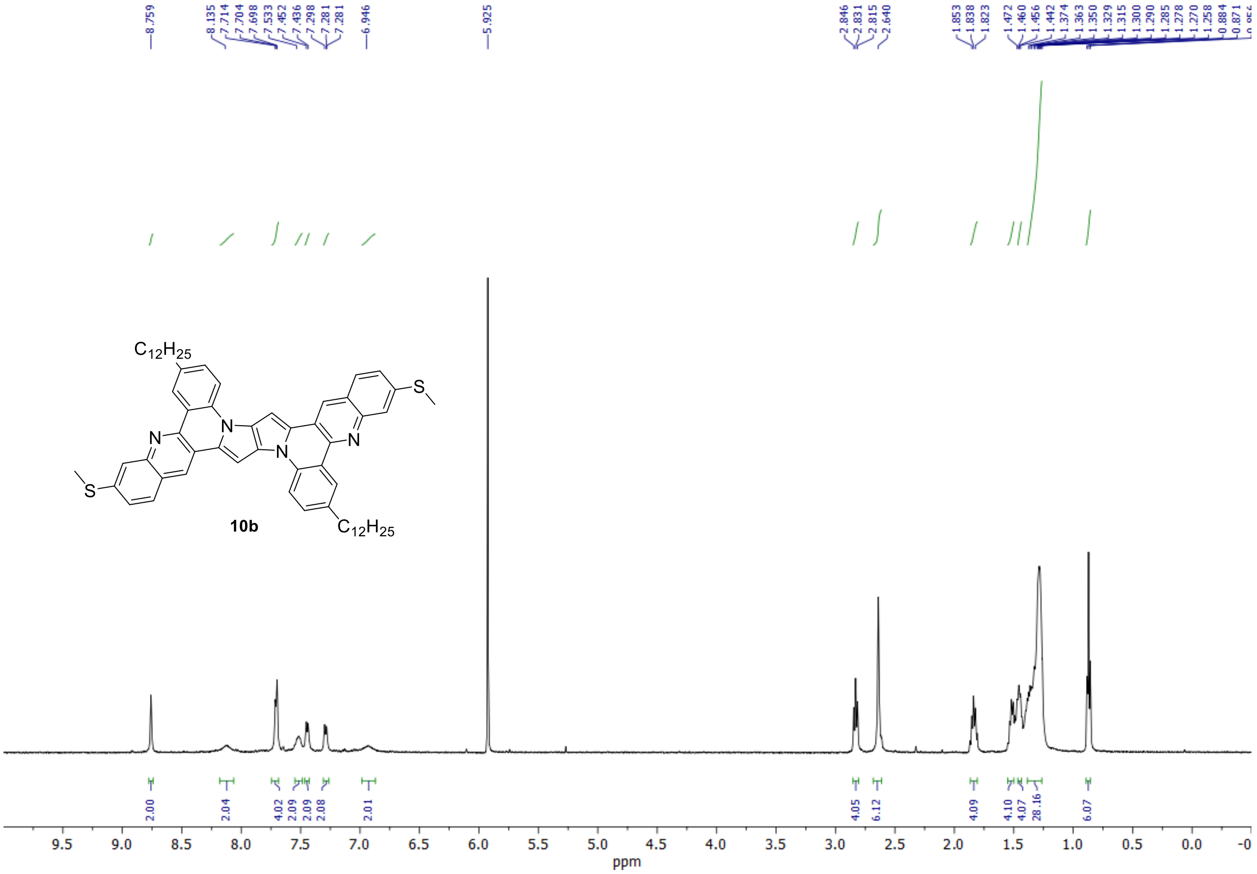

Report of HRMS of **10b**:

Single Mass Analysis

Tolerance = 3.0 mDa / DBE: min = -1.5, max = 150.0

Element prediction: Off

Number of isotope peaks used for i-FIT = 3

Monoisotopic Mass, Even Electron Ions

114 formula(e) evaluated with 1 results within limits (up to 200 closest results for each mass)

Elements Used:

C: 0-200 H: 0-200 N: 0-4 S: 1-2

Mass	Calc. Mass	mDa	PPM	DBE	Formula	i-FIT	i-FIT Norm	Fit Conf %	C	H	N	S
937.5278	937.5277	0.1	0.1	28.5	C62 H73 N4 S2	413.0	n/a	n/a	62	73	4	2

^{1}H NMR (500 MHz, 300K, $CDCl_3$) of **10c**

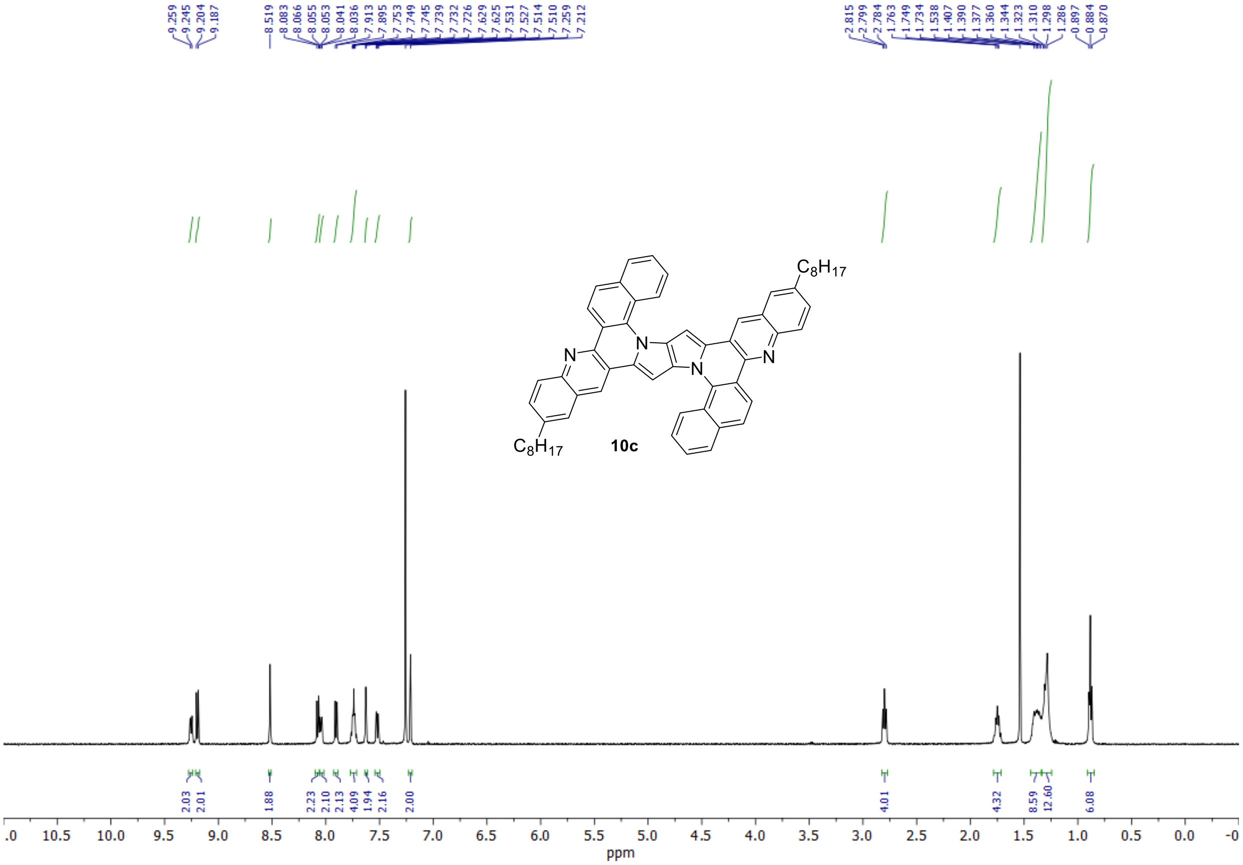

^{13}C{^{1}H} NMR (126 MHz, 300K, $CDCl_3$) of **10c**

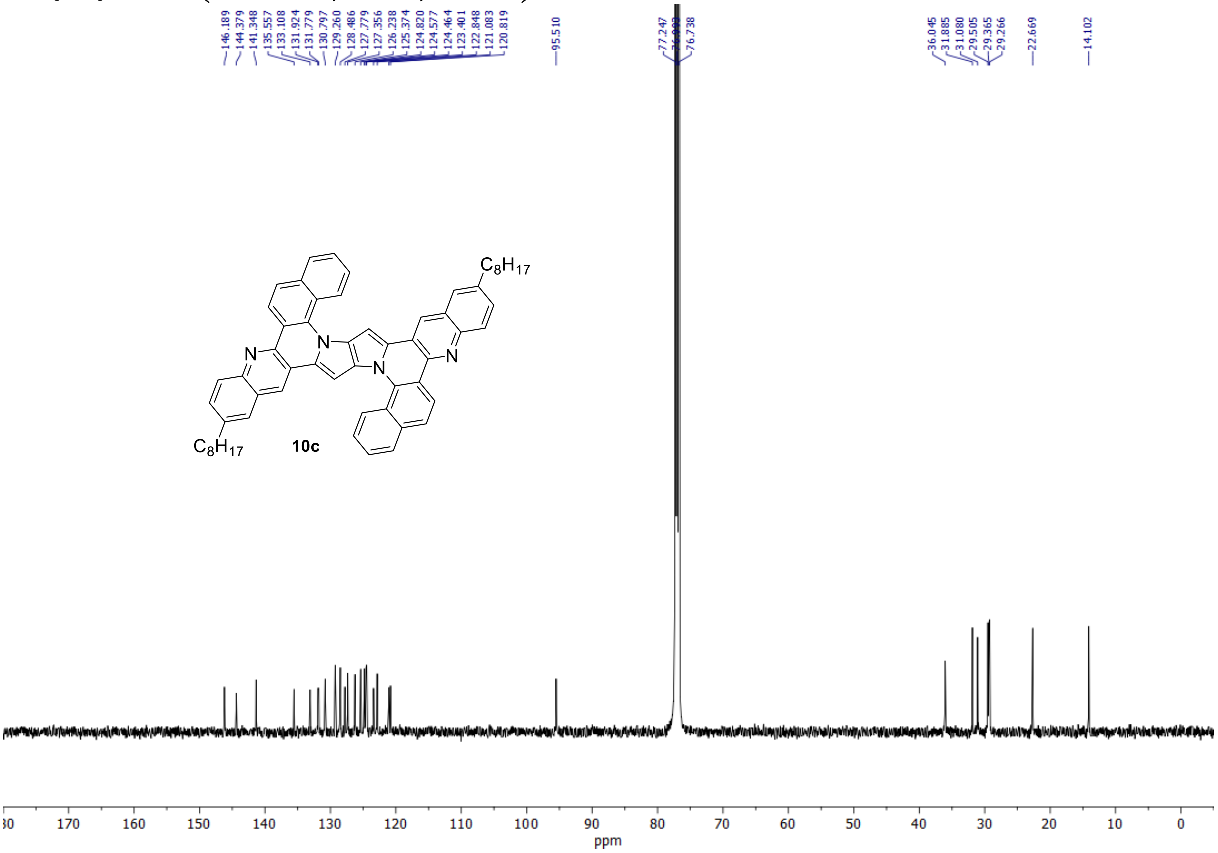

Report of HRMS of **10c**:

Single Mass Analysis
Tolerance = 3.0 mDa / DBE: min = -1.5, max = 150.0
Element prediction: Off
Number of isotope peaks used for i-FIT = 3
Monoisotopic Mass, Even Electron Ions
64 formula(e) evaluated with 1 results within limits (up to 200 closest results for each mass)
Elements Used:

Mass	Calc. Mass	mDa	PPM	DBE	Formula	i-FIT	i-FIT Norm	Fit Conf %	C	H	N
833.4585	833.4583	0.2	0.2	34.5	C60 H57 N4	449.3	n/a	n/a	60	57	4

^{1}H NMR (600 MHz, 300K, $CDCl_3$) of **11a**

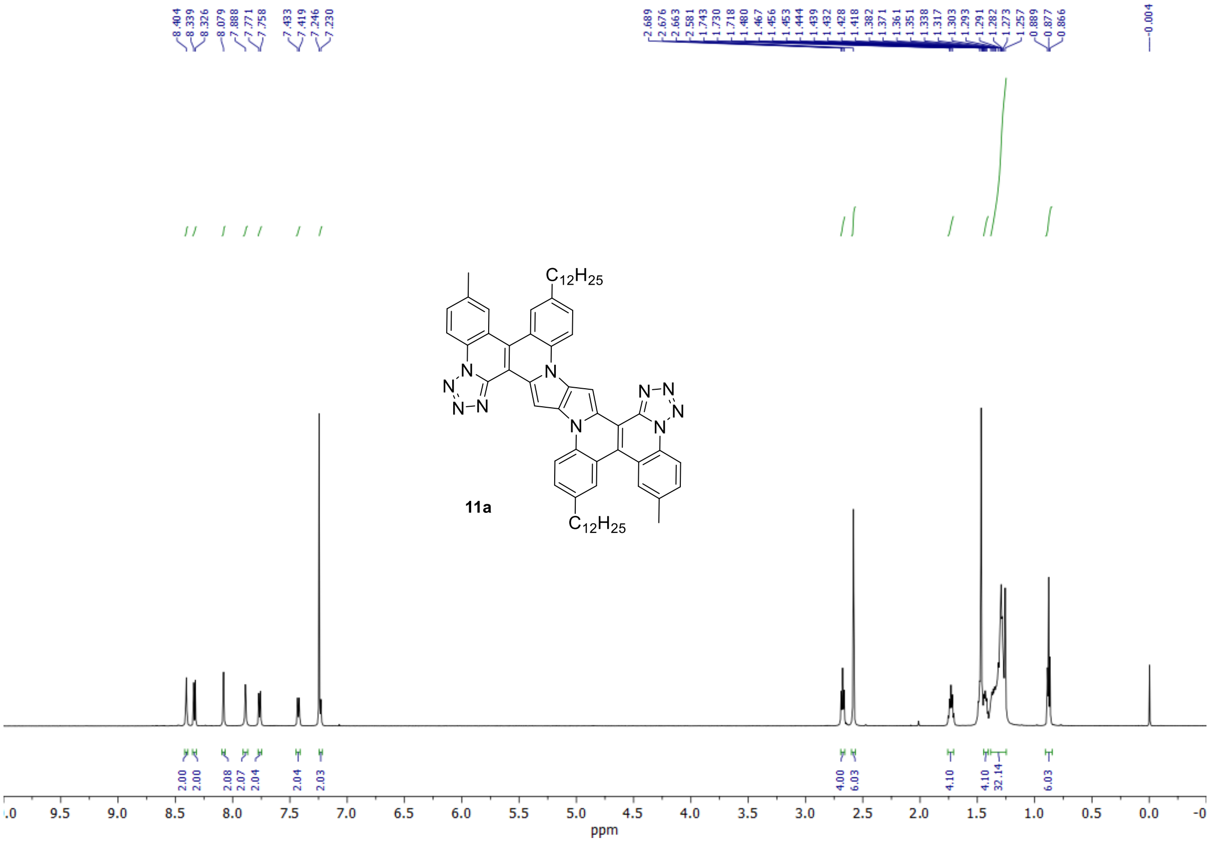

$^{13}C\{^{1}H\}$ NMR (126 MHz, 300K, $CDCl_3$) of **11a**

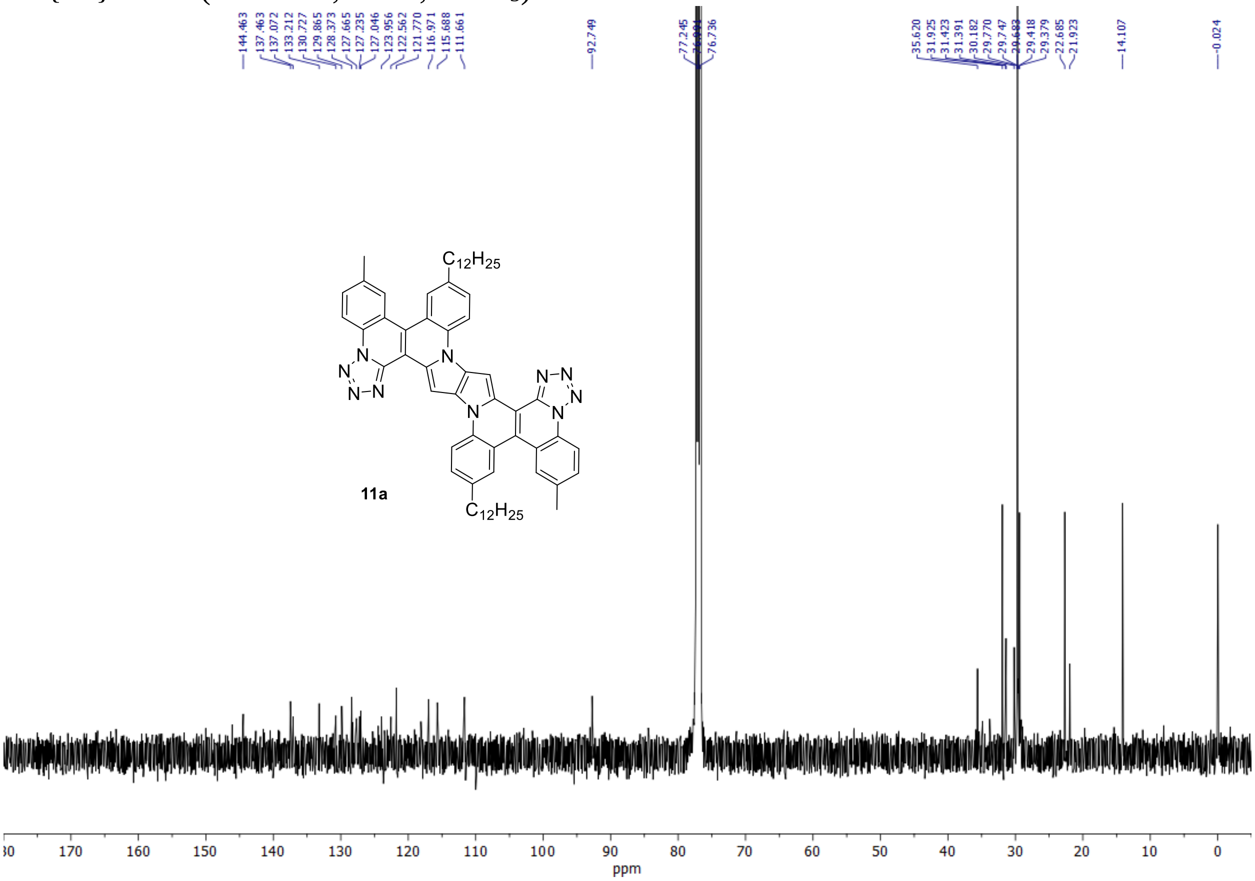

Report of HRMS of **11a**:

Single Mass Analysis

Tolerance = 10.0 PPM / DBE: min = -1.5, max = 150.0

Element prediction: Off

Number of isotope peaks used for i-FIT = 3

Monoisotopic Mass, Even Electron Ions

35 formula(e) evaluated with 1 results within limits (up to 200 closest results for each mass)

Elements Used:

C: 0-200 H: 0-200 N: 9-11

Mass	Calc. Mass	mDa	PPM	DBE	Formula	i-FIT	i-FIT Norm	Fit Conf %	C	H	N
955.5852	955.5863	-1.1	-1.2	32.5	C62 H71 N10	280.5	n/a	n/a	62	71	10

^{1}H NMR (500 MHz, 300K, $CDCl_3$) of **11b**

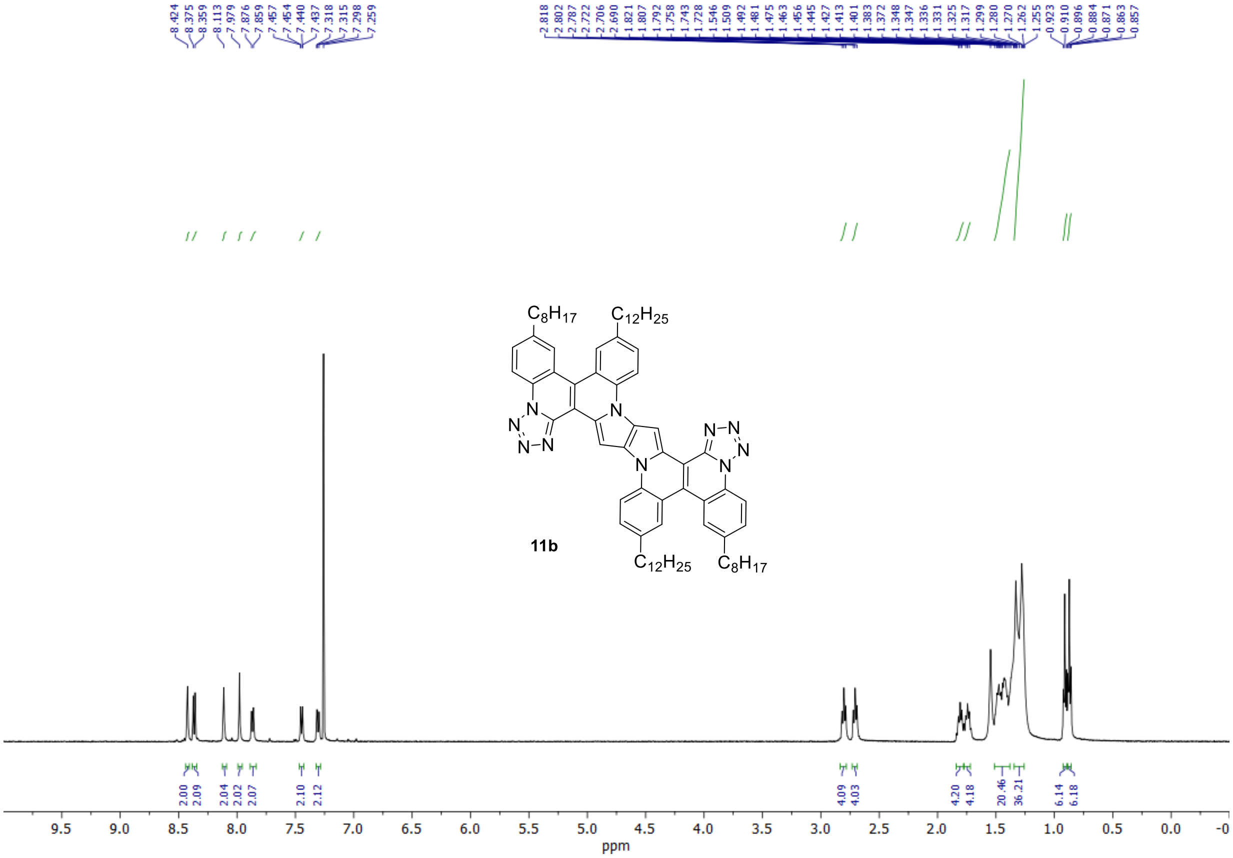

$^{13}C\{^{1}H\}$ NMR (126 MHz, 300K, $CDCl_3$) of **11b**

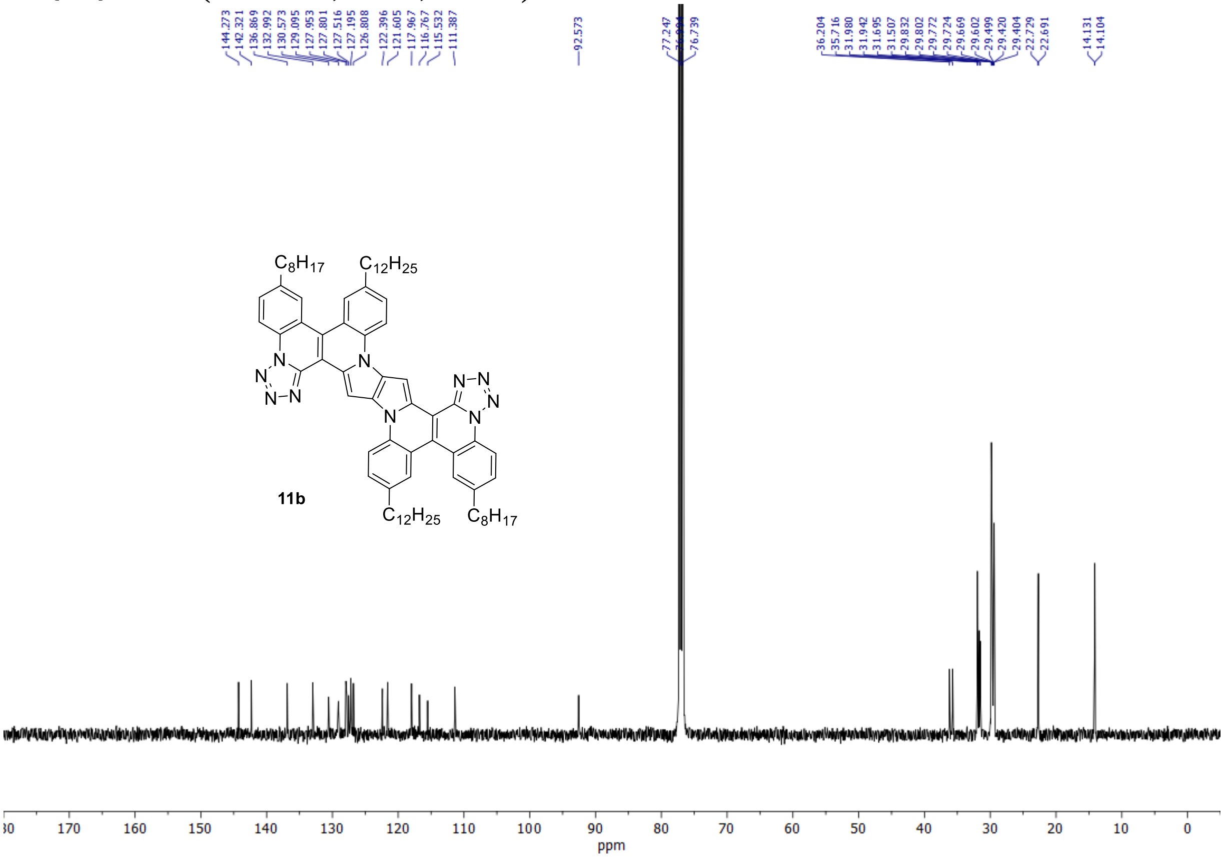

Report of HRMS of **11b**:

Single Mass Analysis
Tolerance = 3.0 mDa / DBE: min = -1.5, max = 150.0
Element prediction: Off
Number of isotope peaks used for i-FIT = 3
Monoisotopic Mass, Odd and Even Electron Ions
172 formula(e) evaluated with 1 results within limits (up to 200 closest results for each mass)
Elements Used:
C: 0-200 H: 0-200 N: 0-11

Mass	Calc. Mass	mDa	PPM	DBE	Formula	i-FIT	i-FIT Norm	Fit Conf %	C	H	N
1150.7977	1150.7976	0.1	0.1	33.0	C76 H98 N10	363.0	n/a	n/a	76	98	10

^{1}H NMR (500 MHz, 300K, $CDCl_3$) of **11c**

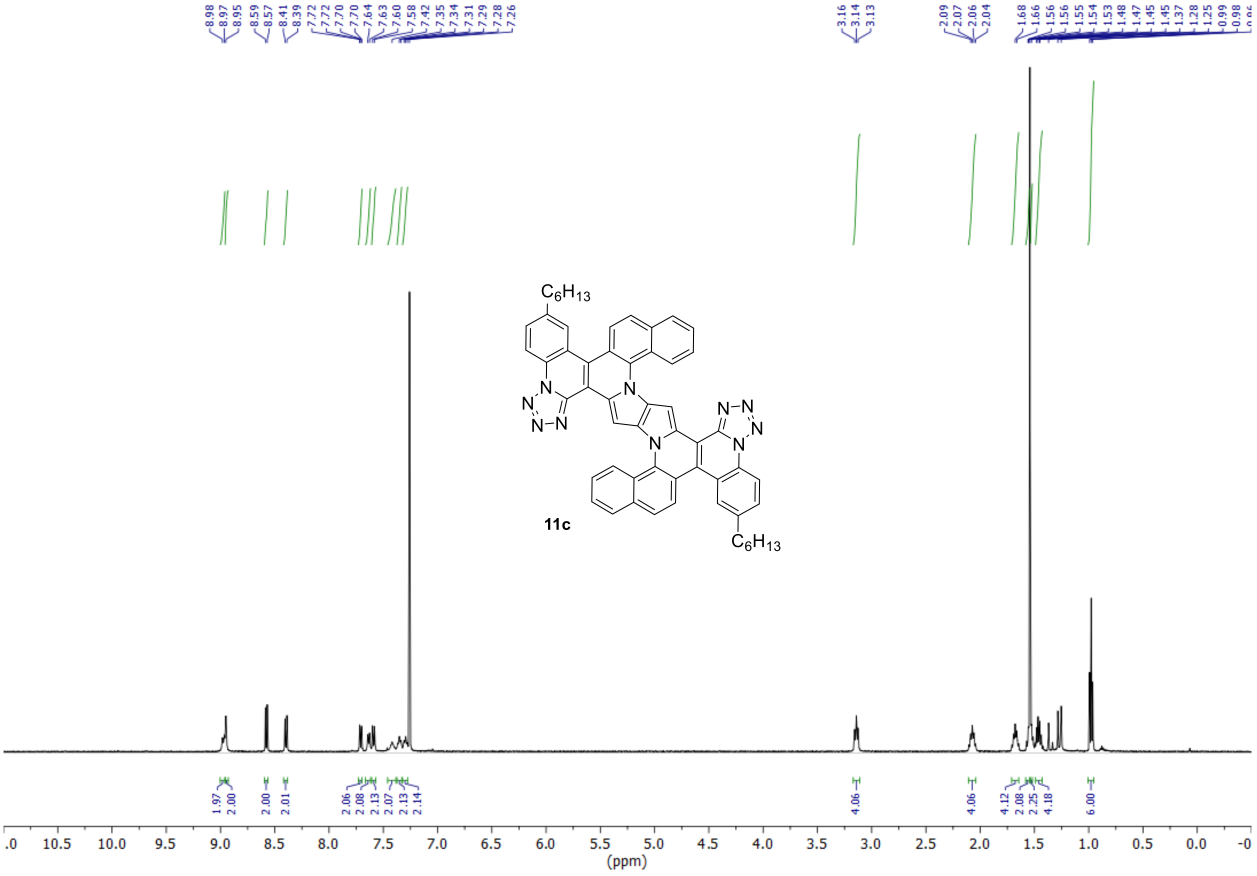

$^{13}C\{^{1}H\}$ NMR (151 MHz, 300K, CD_2Cl_2) of **11c**

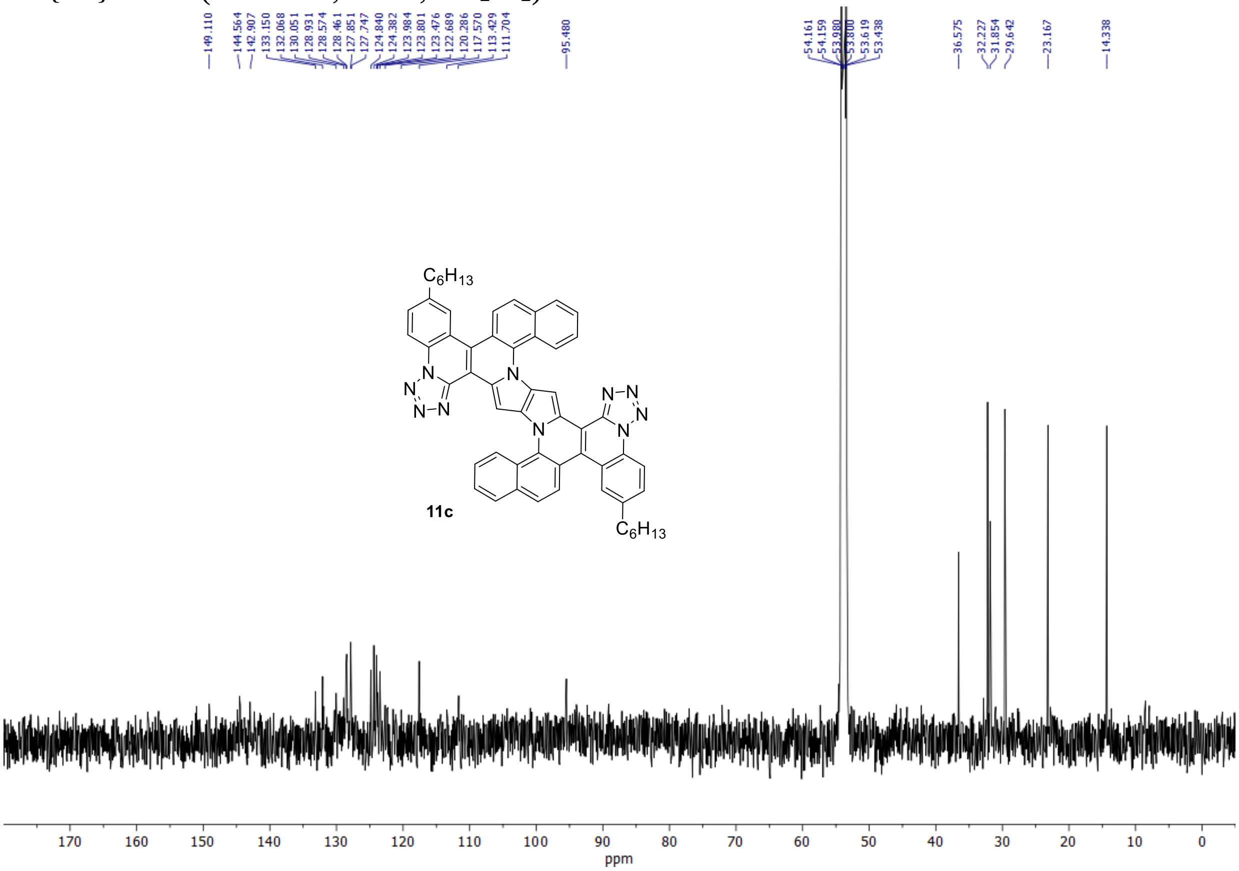

Report of HRMS of **11c**:

Single Mass Analysis
Tolerance = 10.0 mDa / DBE: min = -1.5, max = 150.0
Element prediction: Off
Number of isotope peaks used for i-FIT = 3
Monoisotopic Mass, Even Electron Ions
129 formula(e) evaluated with 2 results within limits (up to 200 closest results for each mass)
Elements Used:
C: 0-200 H: 0-200 N: 0-11

Mass	Calc. Mass	mDa	PPM	DBE	Formula	i-FIT	i-FIT Norm	Fit Conf %	C	H	N
859.3992	859.3985	0.7	0.8	38.5	C56 H47 N10	382.9	0.002	99.85	56	47	10
	859.4052	-6.0	-7.0	41.5	C65 H51 N2	389.4	6.501	0.15	65	51	2

^{1}H NMR (600 MHz, 350K, tetrachloroethane-[D_2]) of **11d**

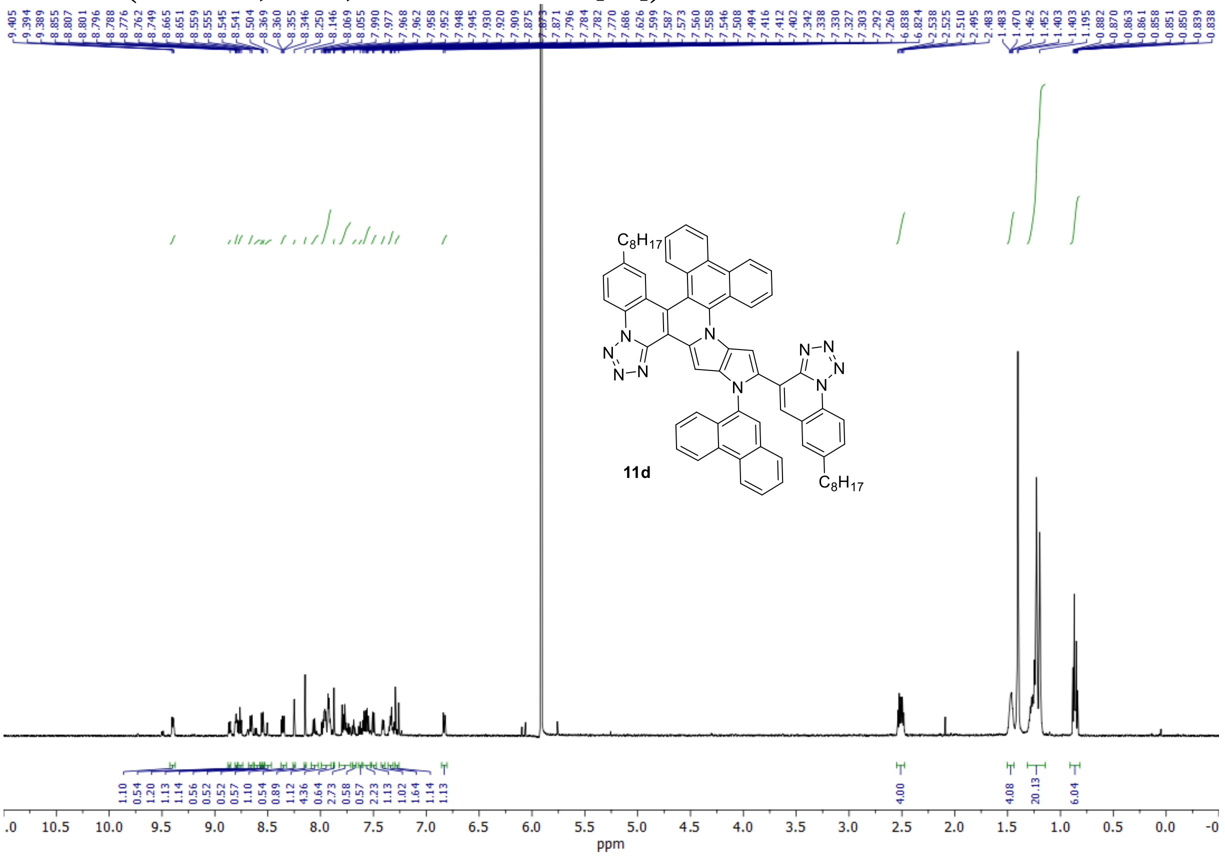

$^{13}C\{^{1}H\}$ NMR (126 MHz, 300K, CD_2Cl_2) of **11d**

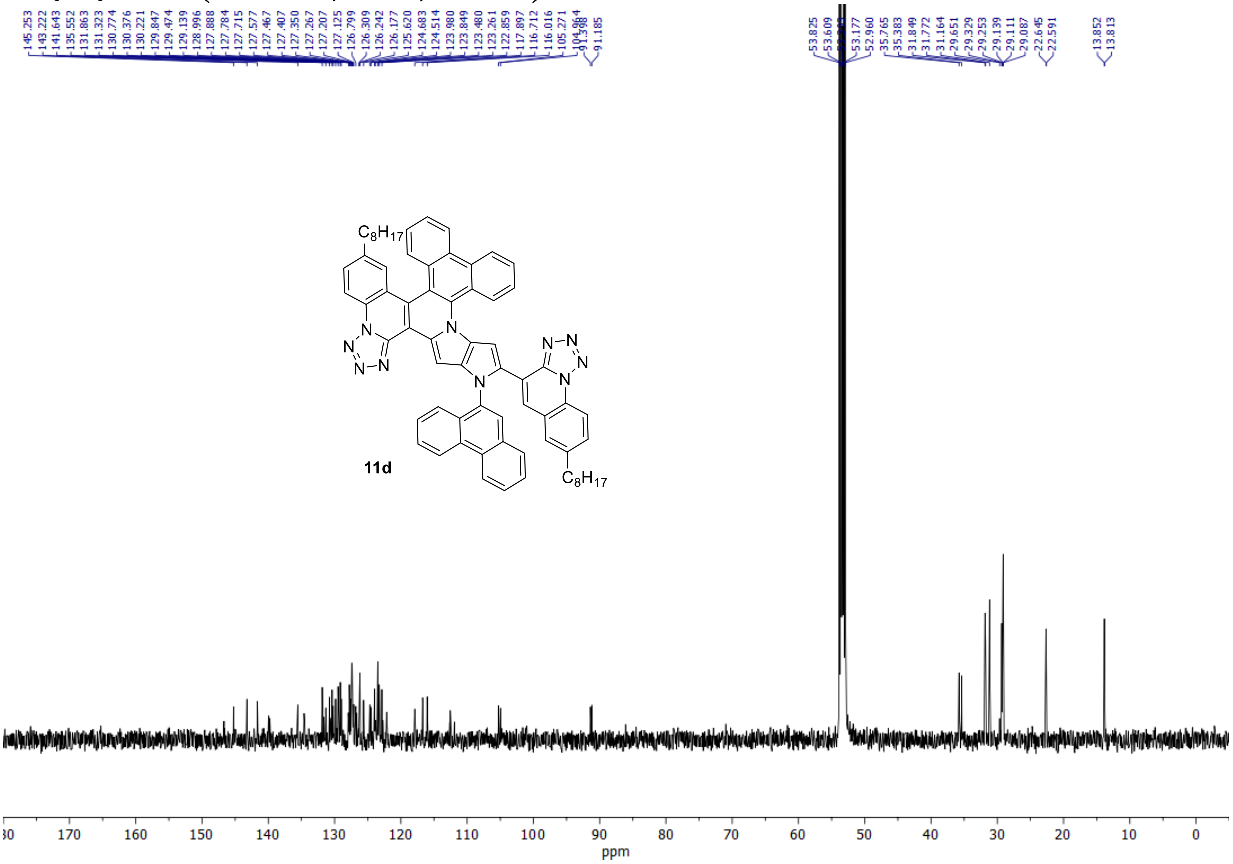

Report of HRMS of **11d**:

Single Mass Analysis
Tolerance = 3.0 mDa / DBE: min = -1.5, max = 150.0
Element prediction: Off
Number of isotope peaks used for i-FIT = 3
Monoisotopic Mass, Even Electron Ions
125 formula(e) evaluated with 1 results within limits (up to 200 closest results for each mass)
Elements Used:
C: 0-200 H: 0-200 N: 2-11

Mass	Calc. Mass	mDa	PPM	DBE	Formula	i-FIT	i-FIT Norm	Fit Conf %	C	H	N
1017.5085	1017.5081	0.4	0.4	43.5	C68 H61 N10	352.1	n/a	n/a	68	61	10

Optical properties of 4a-4l

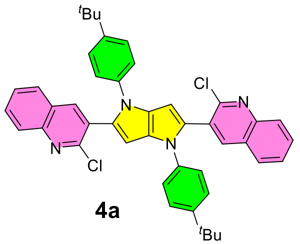

4a

Ab/Em and *ε* ($M^{-1}.cm^{-1}$) *in DCM*	Ab/Em and *ε* ($M^{-1}.cm^{-1}$) *in Toluene*

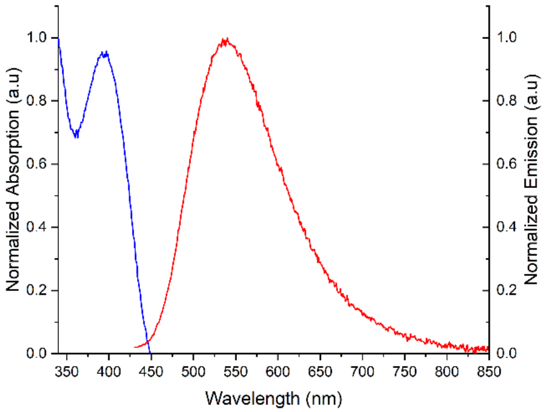

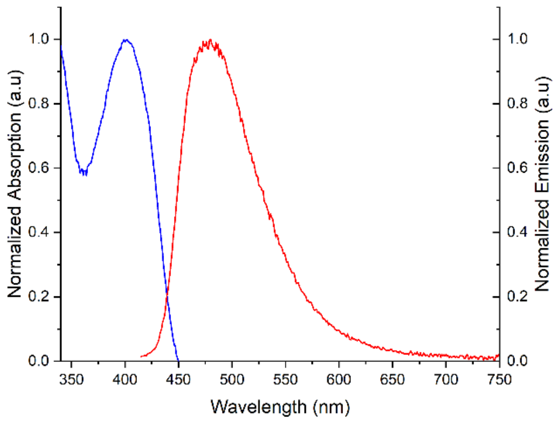

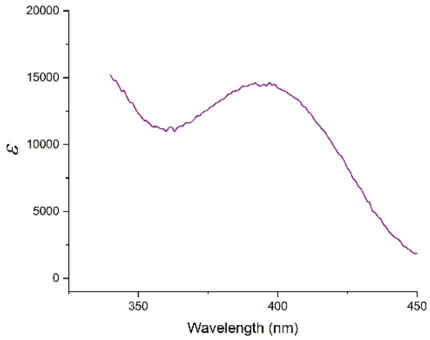

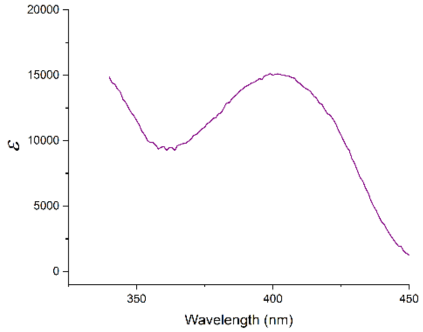

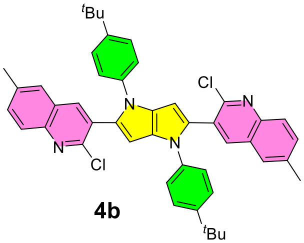

4b

Ab/Em and *ε* ($M^{-1}.cm^{-1}$) *in DCM*	Ab/Em and *ε* ($M^{-1}.cm^{-1}$) *in Toluene*

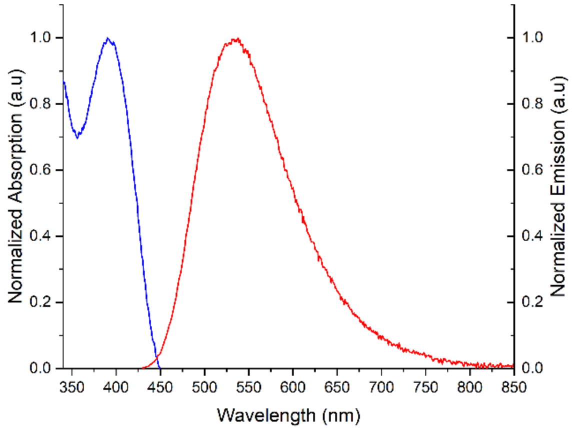

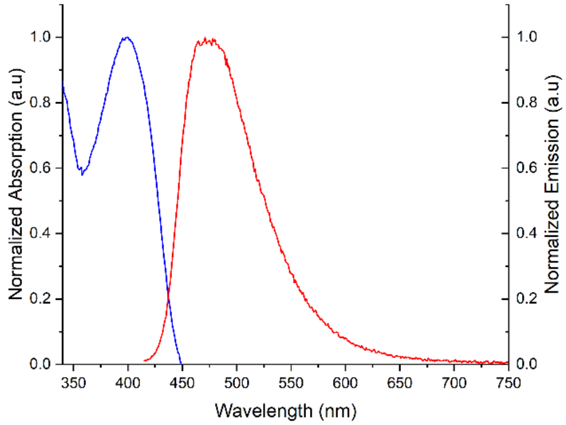

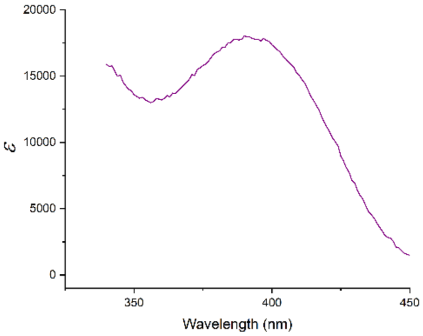

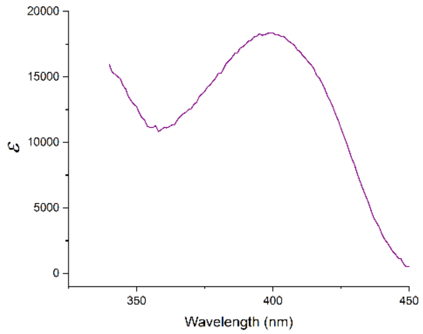

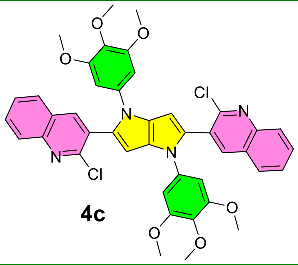

4c

**Ab**/**Em** and ***ε*** ($M^{-1}.cm^{-1}$) *in DCM*	**Ab**/**Em** and ***ε*** ($M^{-1}.cm^{-1}$) *in Toluene*

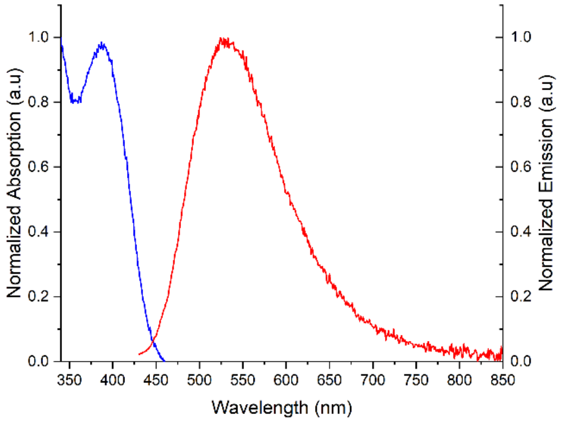

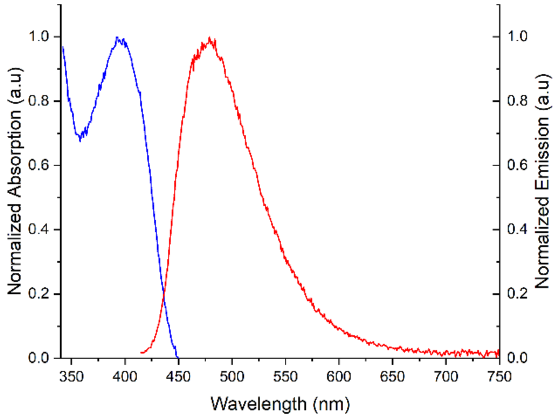

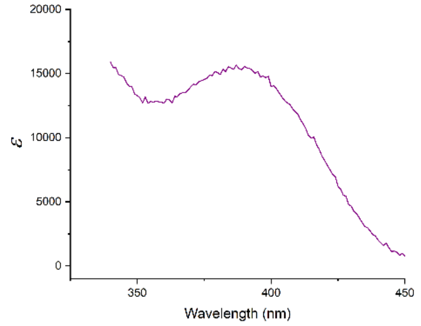

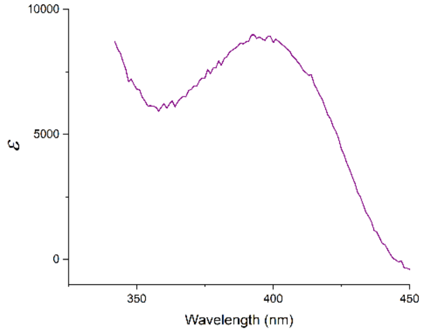

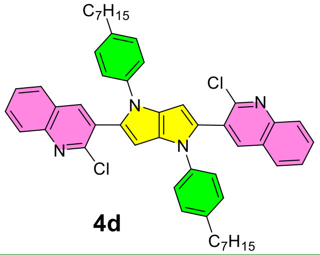

4d

**Ab**/**Em** and ***ε*** ($M^{-1}.cm^{-1}$) *in DCM*	**Ab**/**Em** and ***ε*** ($M^{-1}.cm^{-1}$) *in Toluene*

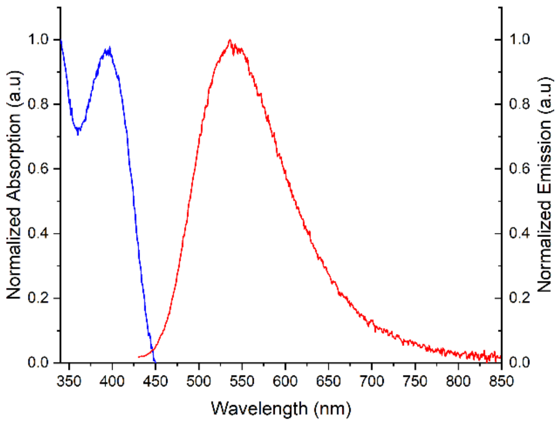

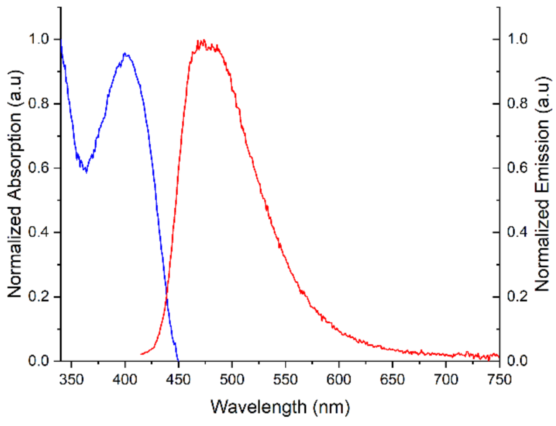

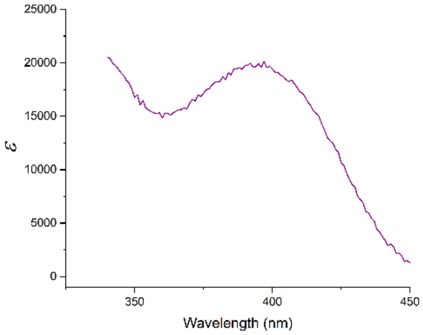

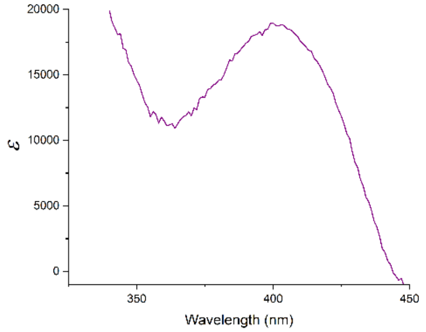

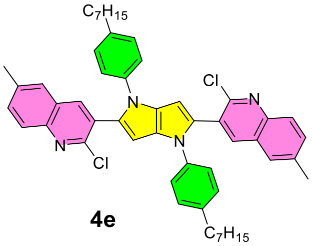

4e

Ab/**Em** and *ε* ($M^{-1}.cm^{-1}$) *in DCM*

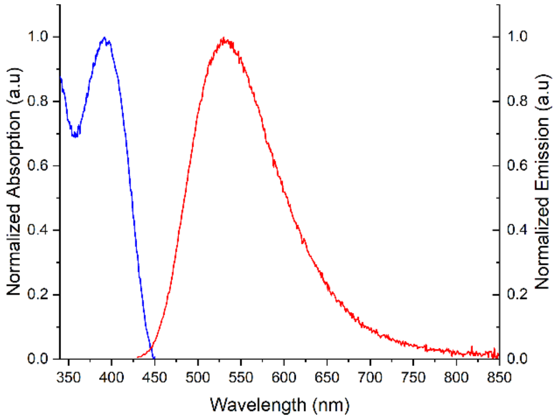

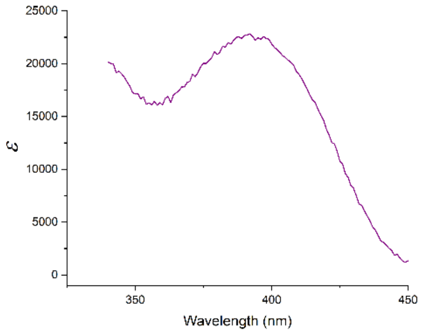

Ab/**Em** and *ε* ($M^{-1}.cm^{-1}$) *in Toluene*

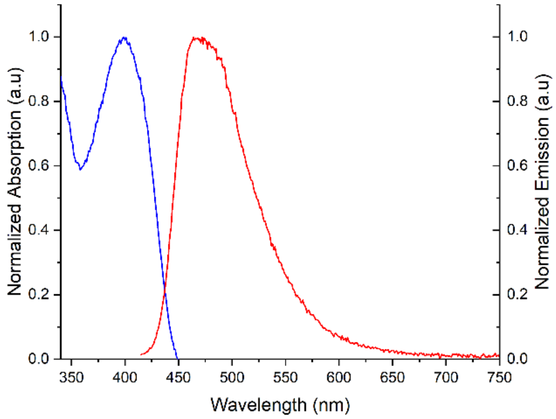

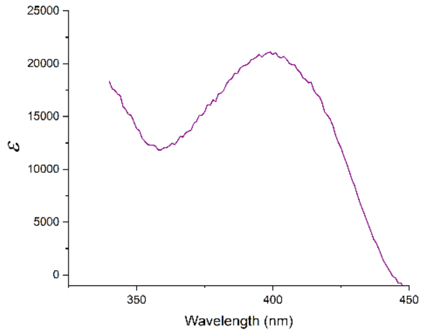

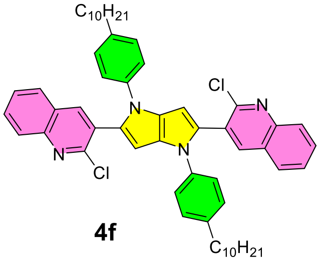

4f

Ab/**Em** and *ε* ($M^{-1}.cm^{-1}$) *in DCM*

Ab/**Em** and *ε* ($M^{-1}.cm^{-1}$) *in Toluene*

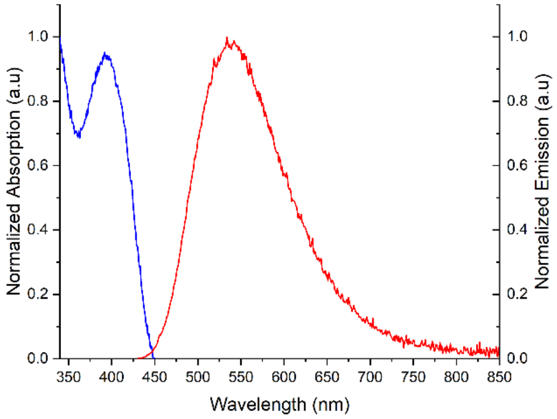

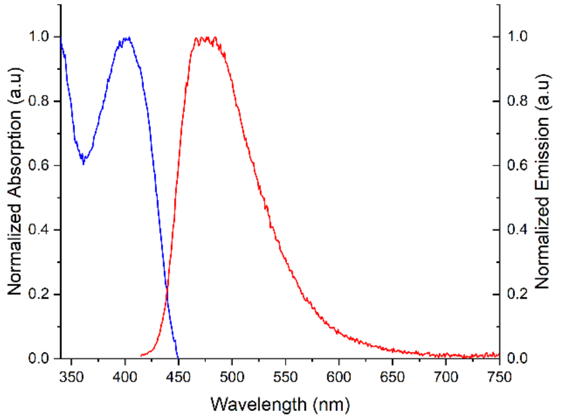

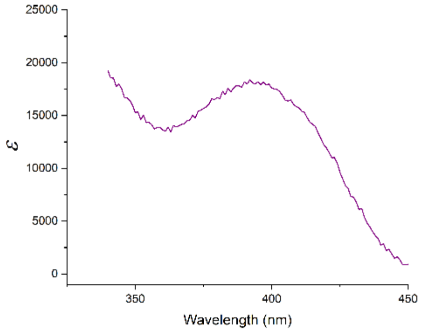

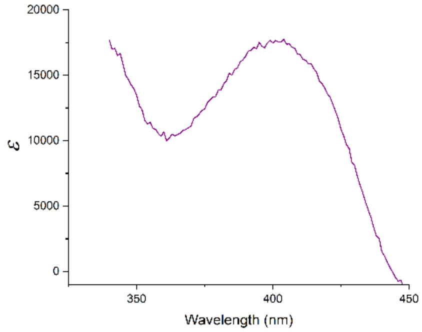

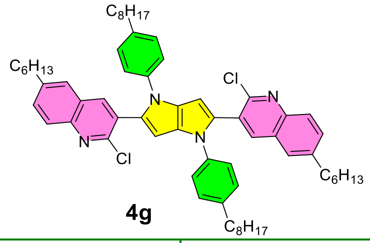

4g

**Ab/Em and ε ($M^{-1}.cm^{-1}$)** *in DCM*	**Ab/Em and ε ($M^{-1}.cm^{-1}$)** *in Toluene*

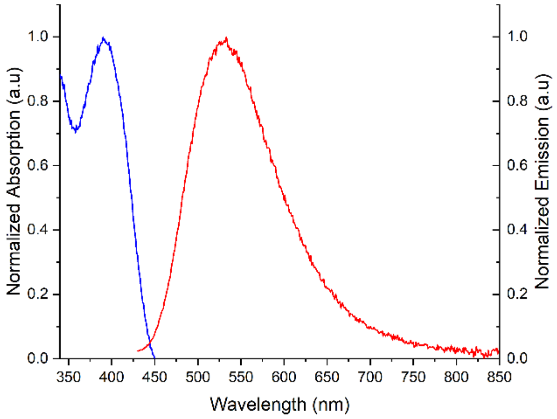

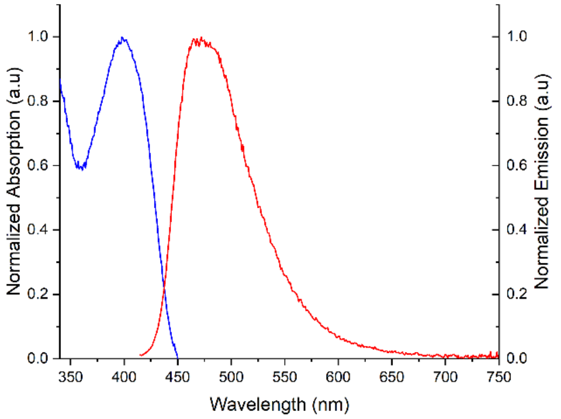

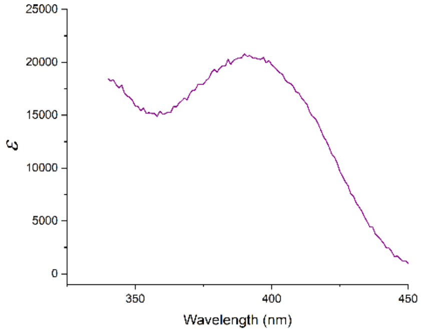

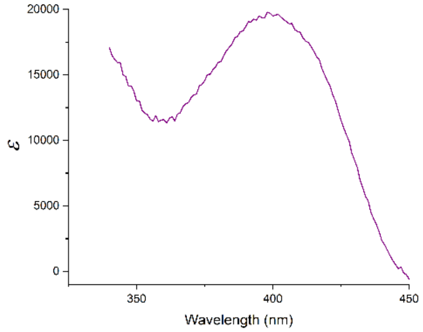

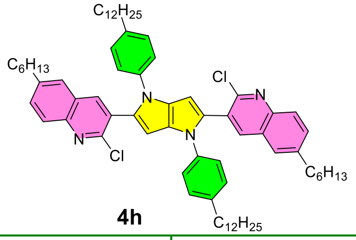

4h

**Ab**/**Em** and ***ε*** ($M^{-1}.cm^{-1}$) *in DCM*	**Ab**/**Em** and ***ε*** ($M^{-1}.cm^{-1}$) *in Toluene*

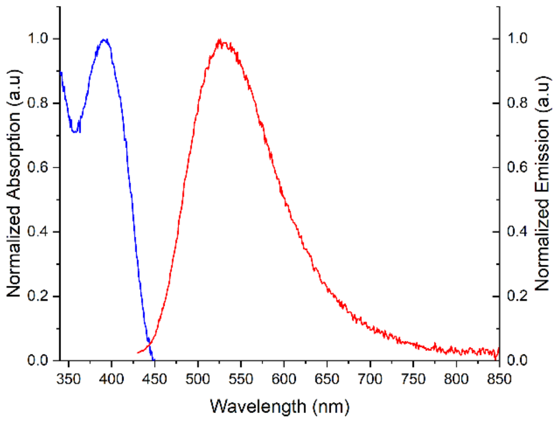

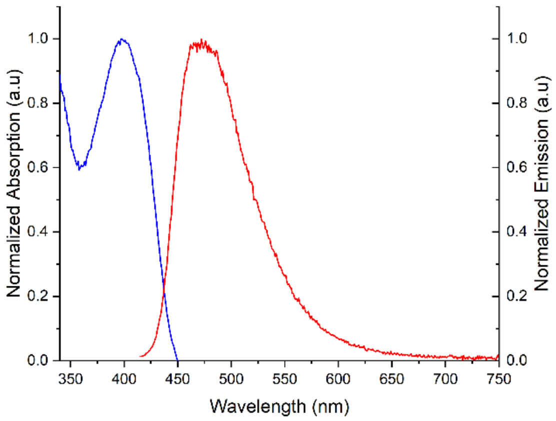

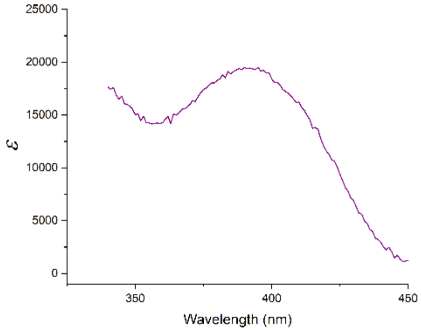

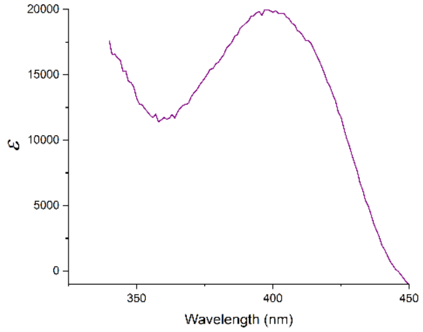

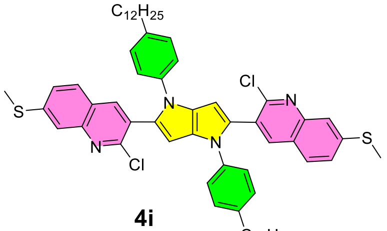

4i

**Ab**/**Em** and *ε* (M^{-1}.cm^{-1}) *in DCM*	**Ab**/**Em** and *ε* (M^{-1}.cm^{-1}) *in Toluene*

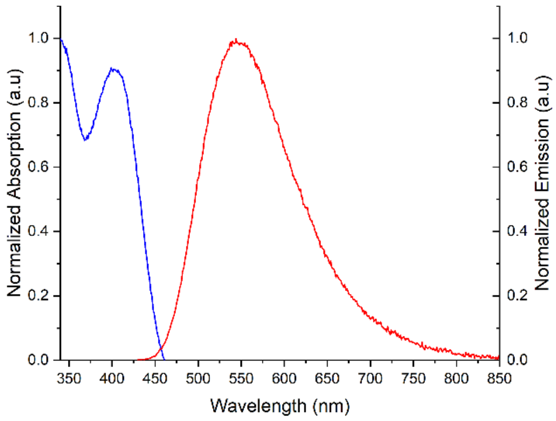

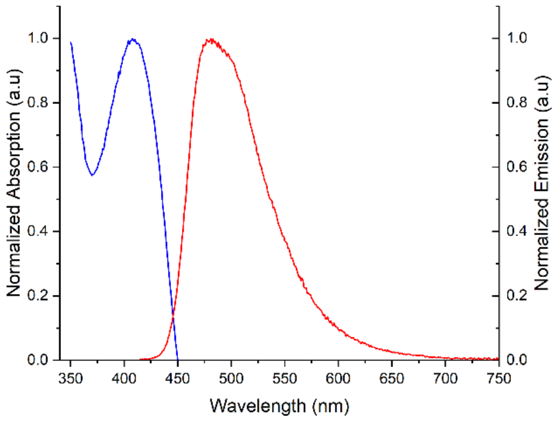

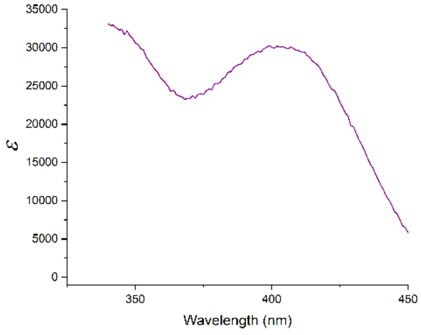

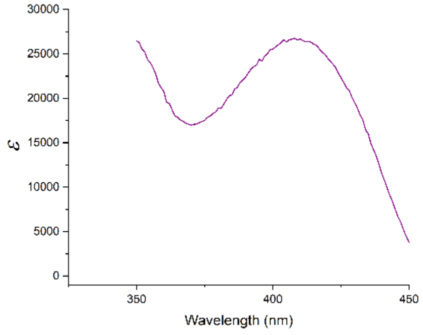

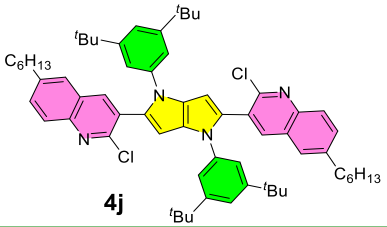

4j

**Ab**/**Em** and *ε* (M^{-1}.cm^{-1}) *in DCM*	**Ab**/**Em** and *ε* (M^{-1}.cm^{-1}) *in Toluene*

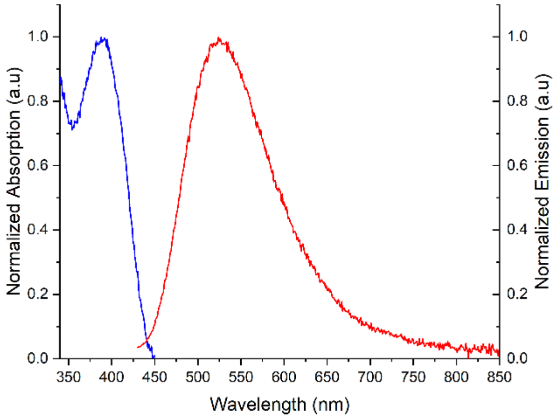

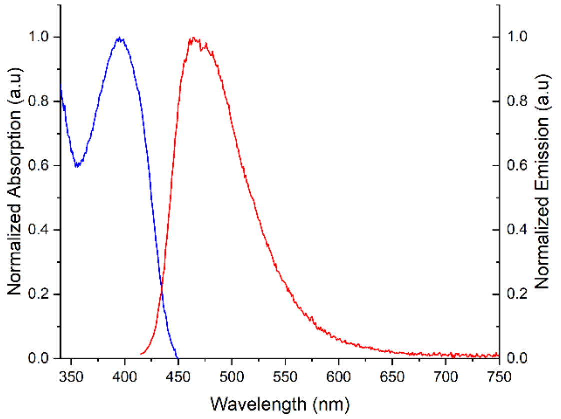

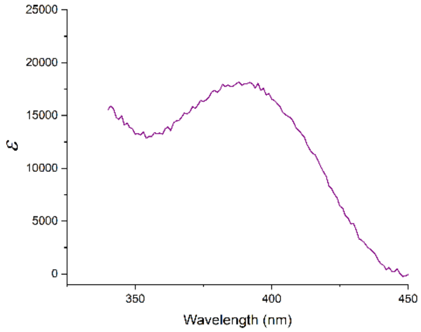

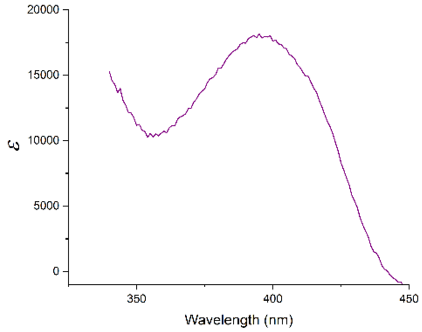

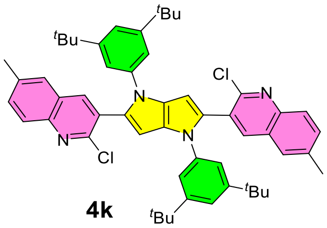

4k

**Ab**/**Em** and ***ε*** ($M^{-1}.cm^{-1}$) *in DCM*	**Ab**/**Em** and ***ε*** ($M^{-1}.cm^{-1}$) *in Toluene*

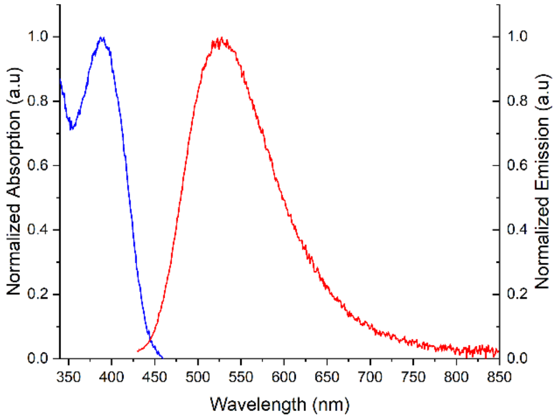

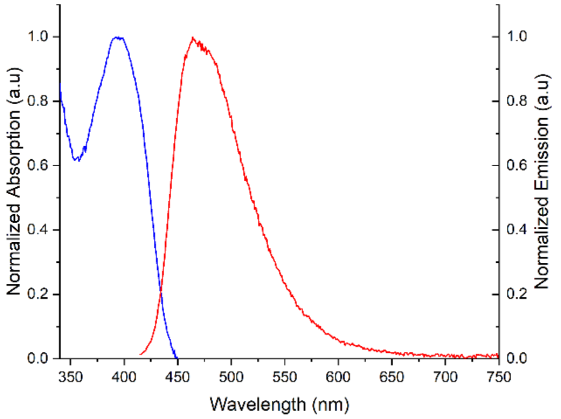

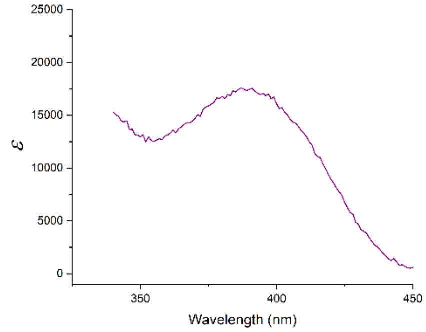

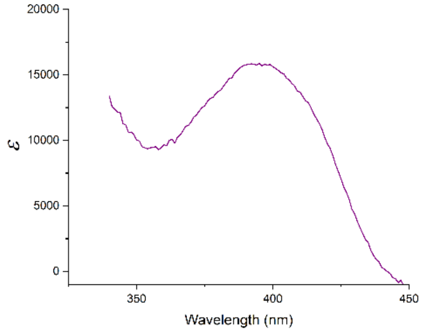

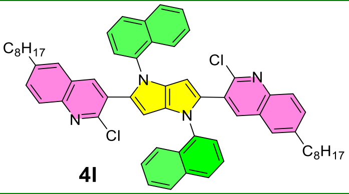

4l

Ab/**Em** and ***ε*** ($M^{-1}.cm^{-1}$)
in DCM

Ab/**Em** and ***ε*** ($M^{-1}.cm^{-1}$)
in Toluene

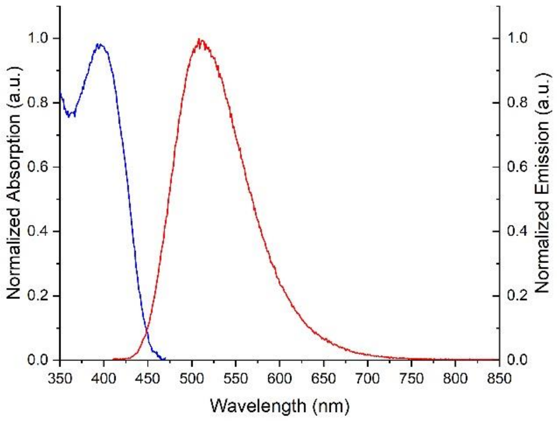

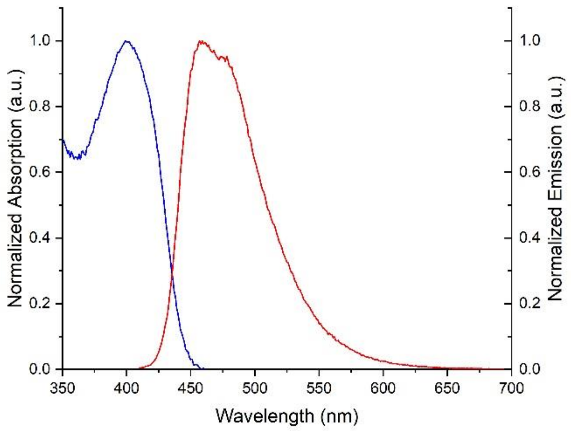

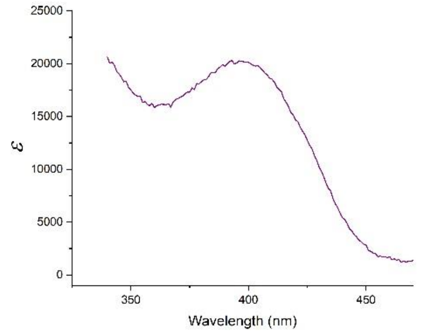

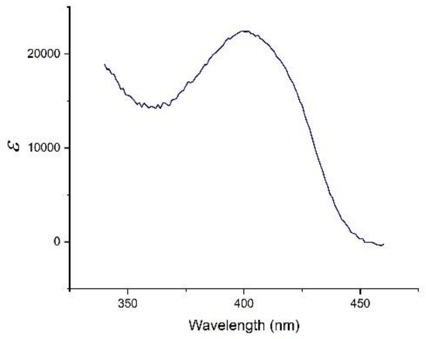

Table S1. Photophysical data for dyes **4a-4l**.

Entry	*in* DCM					*in* Toluene				
	$\lambda_{max\,(Ab)}$ (nm)	ε @ λ_{max} ($M^{-1}cm^{-1}$)	$\lambda_{max\,(Em)}$ (nm)	Stokes Shift (cm^{-1})	Φ_{fl}	$\lambda_{max\,(Ab)}$ (nm)	ε @ λ_{max} ($M^{-1}cm^{-1}$)	$\lambda_{max\,(Em)}$ (nm)	Stokes Shift (cm^{-1})	Φ_{fl}
**4a**	397	14700	540	6700	0.13	399	15100	480	4200	0.19
**4b**	390	18000	538	7000	0.12	399	18300	471	3800	0.20
**4c**	387	15700	524	6800	0.12	393	9000	479	4600	0.17
**4d**	397	20100	536	6500	0.15	400	18900	474	3900	0.26
**4e**	392	22800	530	6600	0.15	399	21100	464	3500	0.19
**4f**	392	18400	534	6800	0.15	404	17700	476	3700	0.21
**4g**	390	20800	533	6900	0.15	398	19800	472	3900	0.20
**4h**	395	19500	526	6300	0.15	398	20000	472	3900	0.20
**4i**	399	30300	544	6700	0.20	408	26800	481	3700	0.33
**4j**	388	18200	524	6700	0.13	395	18200	464	3800	0.18
**4k**	387	17600	528	6900	0.13	395	15900	464	3800	0.18
**4l**	396	20200	512	5700	0.18	399	22500	457	3200	0.13

Optical properties of 6a-6r

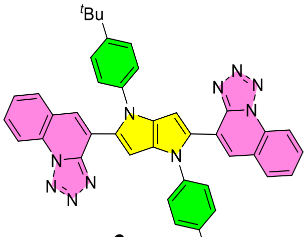

6a

**Ab**/**Em** and ε ($M^{-1}.cm^{-1}$) *in DCM*	**Ab**/**Em** and ε ($M^{-1}.cm^{-1}$) *in Toluene*

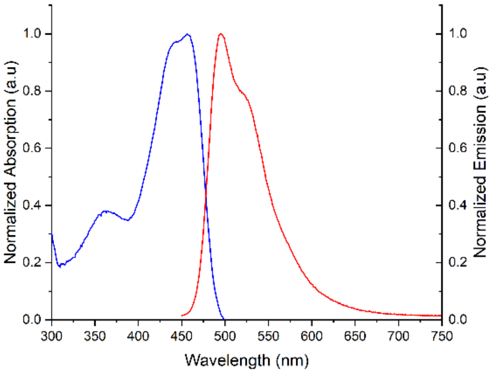

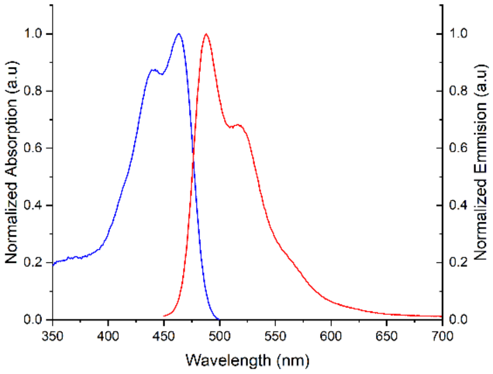

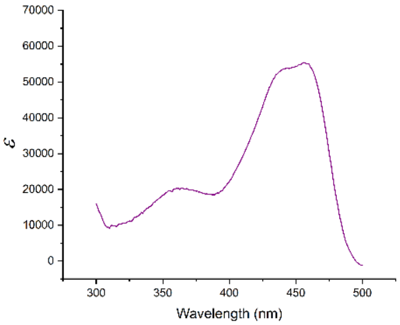

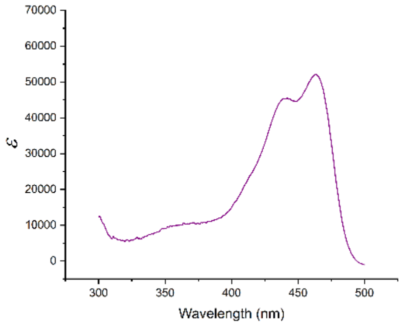

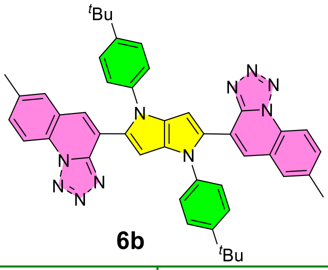

6b

**Ab**/**Em** and ε ($M^{-1}.cm^{-1}$) *in DCM*	**Ab**/**Em** and ε ($M^{-1}.cm^{-1}$) *in Toluene*

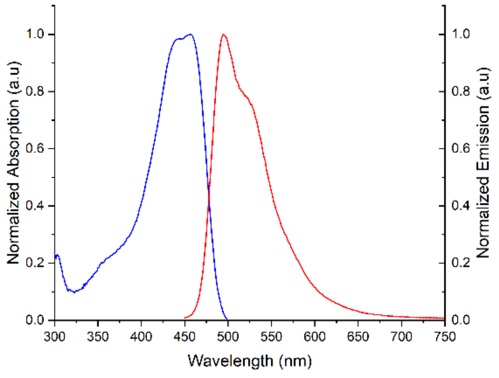

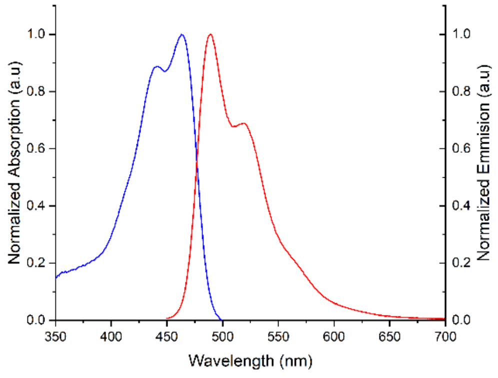

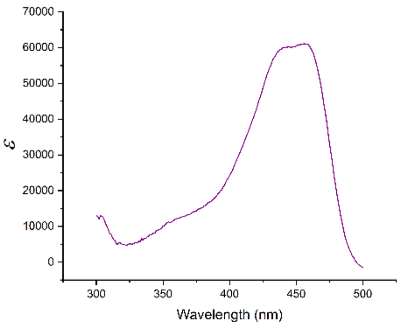

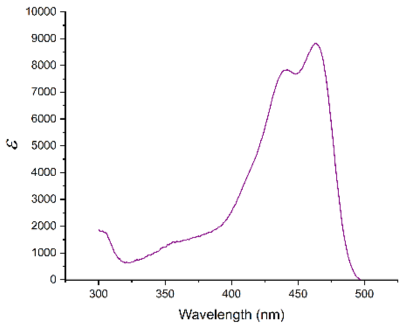

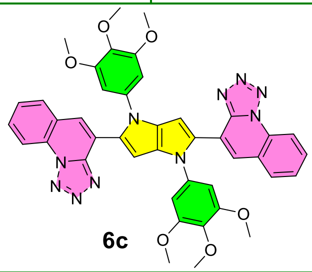

6c

**Ab**/**Em** and ***ε*** ($M^{-1}.cm^{-1}$) *in DCM*	**Ab**/**Em** and ***ε*** ($M^{-1}.cm^{-1}$) *in Toluene*

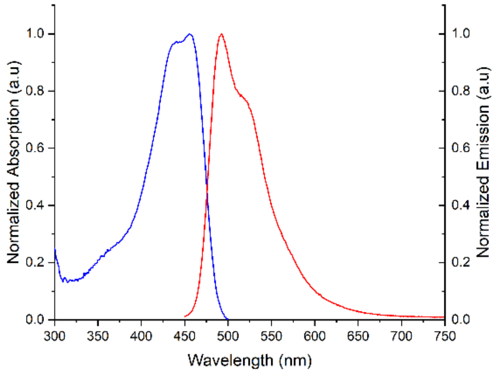

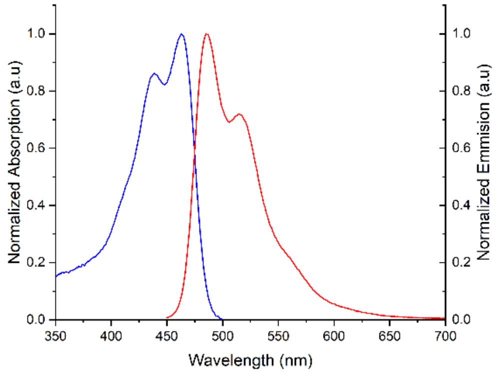

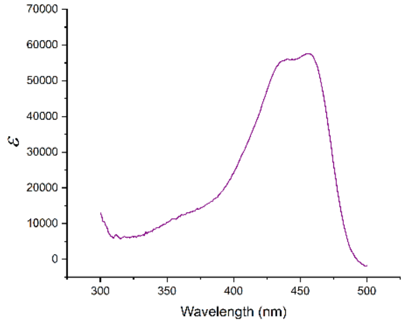

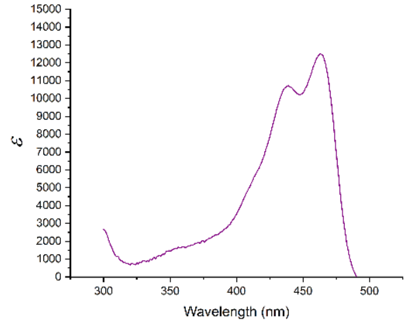

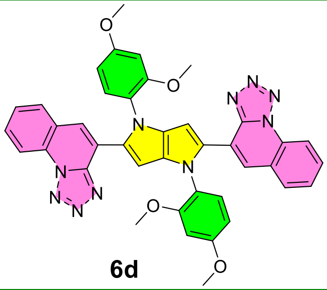

6d

**Ab**/**Em** and *ε* ($M^{-1}.cm^{-1}$) *in DCM*	**Ab**/**Em** and *ε* ($M^{-1}.cm^{-1}$) *in Toluene*

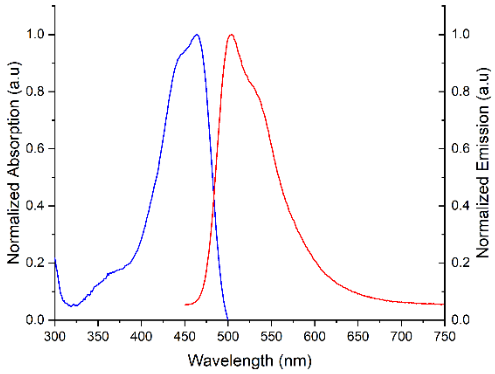

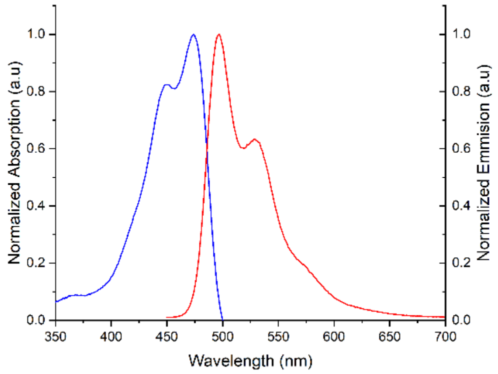

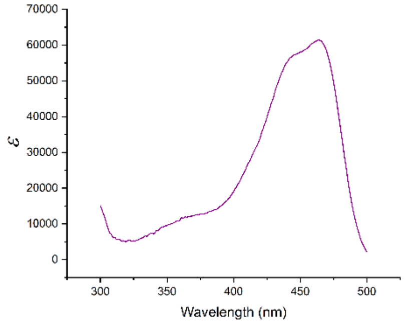

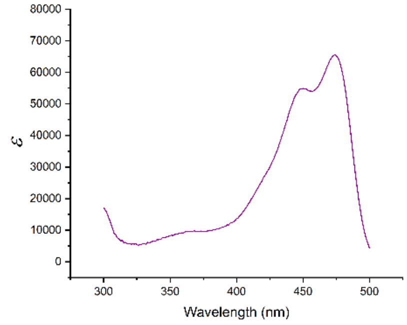

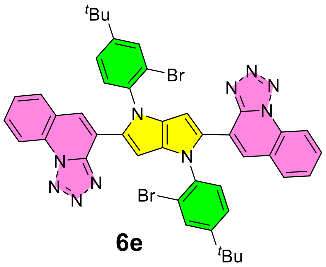

6e

**Ab/Em** and ***ε*** ($M^{-1}.cm^{-1}$) *in DCM*	**Ab/Em** and ***ε*** ($M^{-1}.cm^{-1}$) *in Toluene*
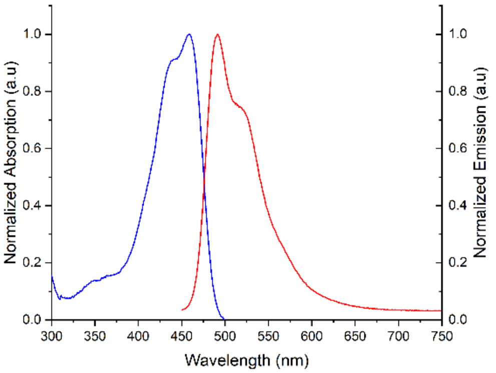	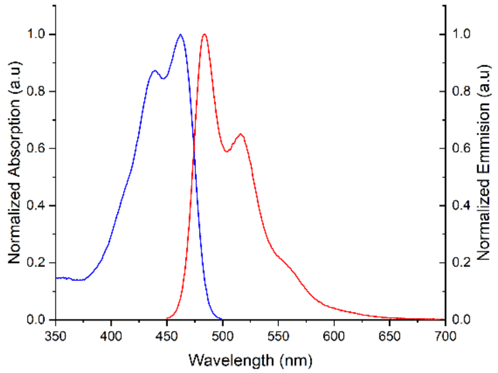
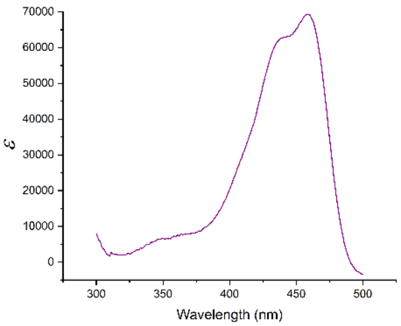	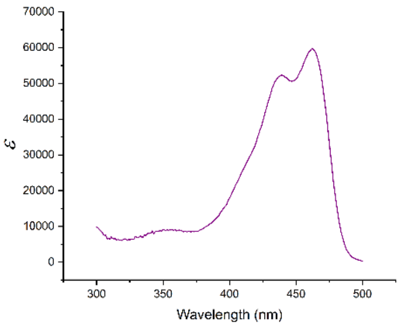

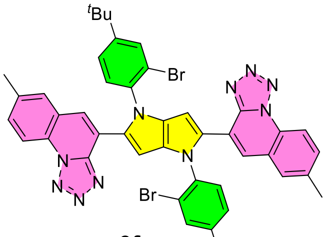

6f

**Ab/Em** and ***ε*** ($M^{-1}.cm^{-1}$) *in DCM*	**Ab/Em** and ***ε*** ($M^{-1}.cm^{-1}$) *in Toluene*

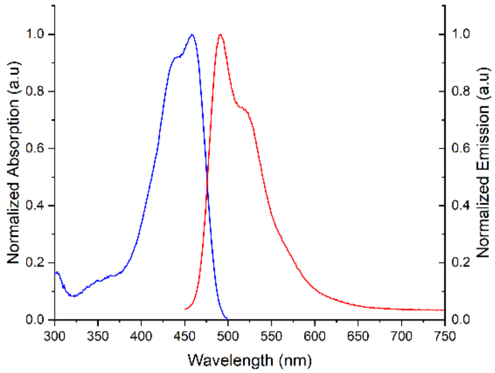

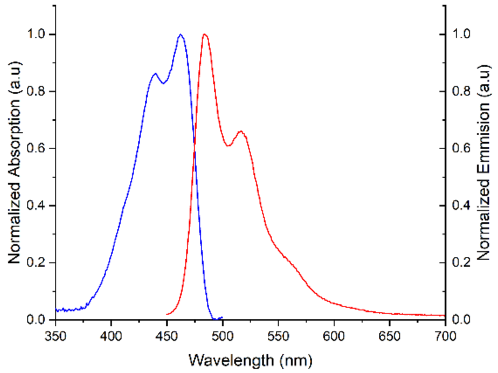

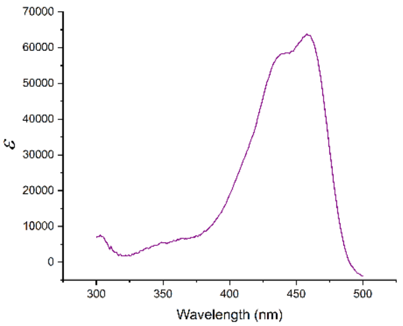

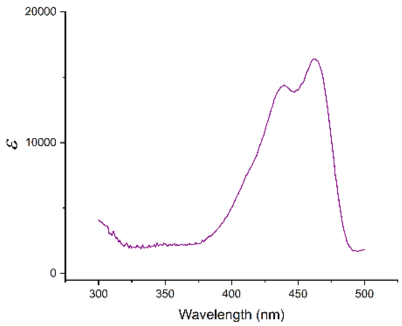

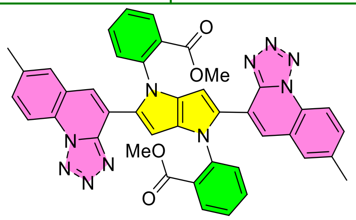

6g

**Ab**/**Em** and ***ε*** ($M^{-1}.cm^{-1}$) *in DCM*	**Ab**/**Em** and ***ε*** ($M^{-1}.cm^{-1}$) *in Toluene*

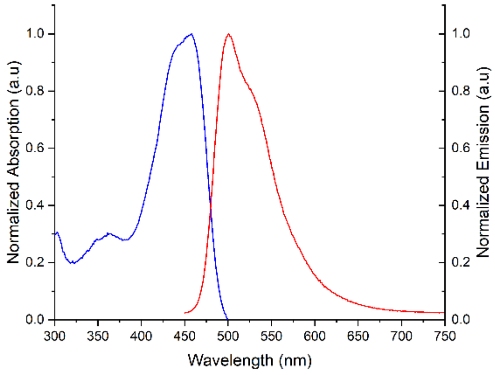

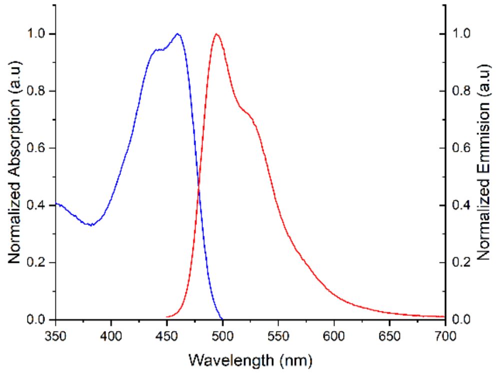

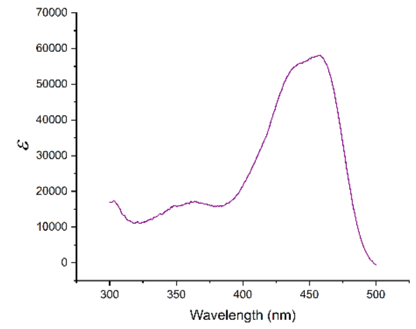

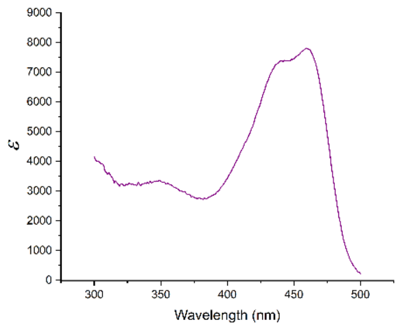

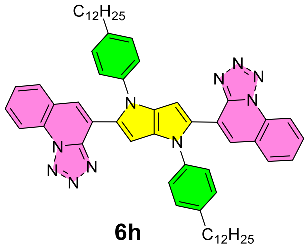

6h

**Ab**/**Em** and *ε* ($M^{-1}.cm^{-1}$) *in DCM*	**Ab**/**Em** and *ε* ($M^{-1}.cm^{-1}$) *in Toluene*

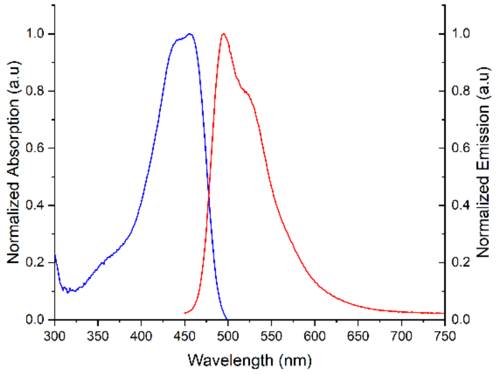

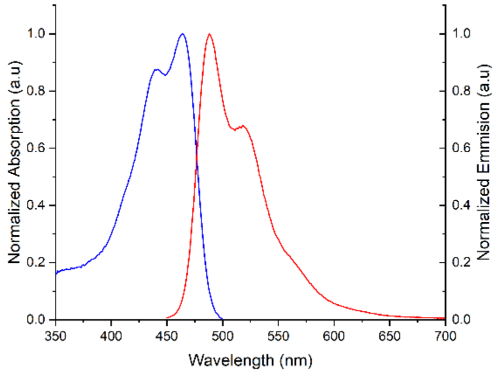

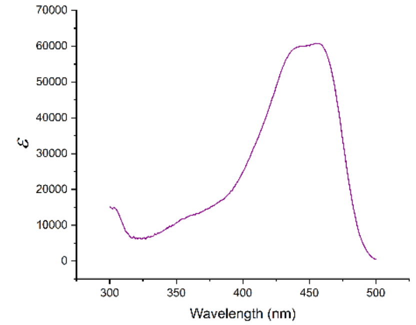

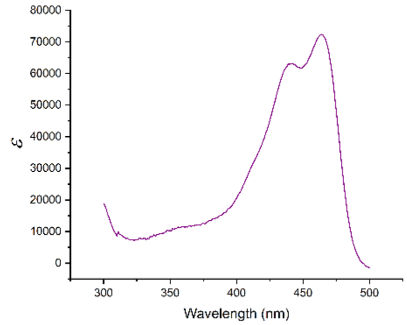

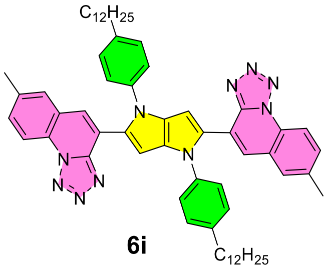

6i

**Ab**/**Em** and *ε* ($M^{-1}.cm^{-1}$) *in DCM*	**Ab**/**Em** and *ε* ($M^{-1}.cm^{-1}$) *in Toluene*

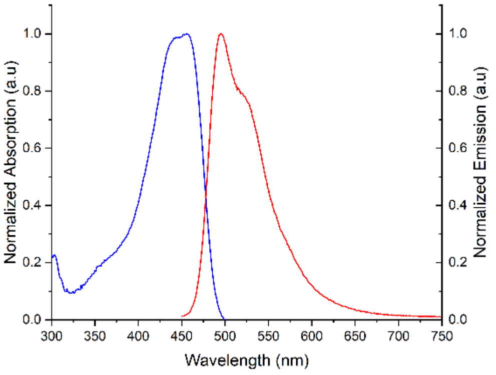

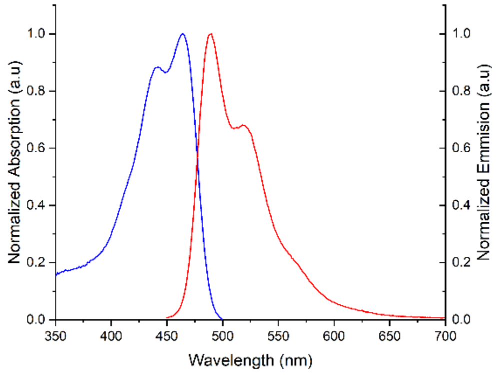

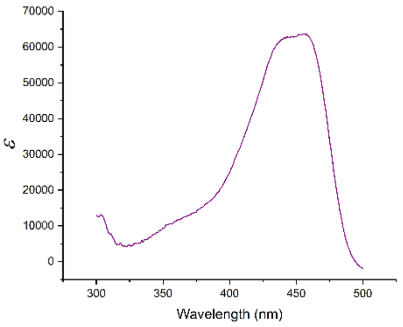

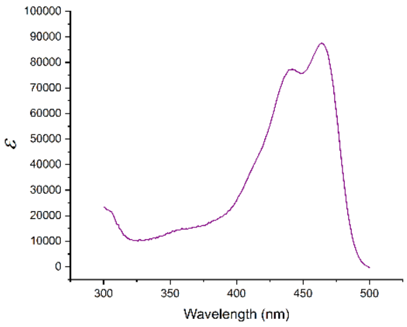

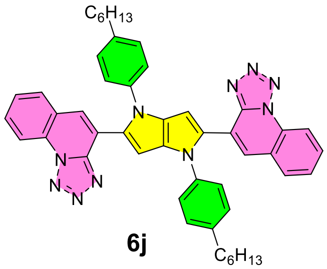

6j

**Ab**/**Em** and *ε* ($M^{-1}.cm^{-1}$) *in DCM*	**Ab**/**Em** and *ε* ($M^{-1}.cm^{-1}$) *in Toluene*

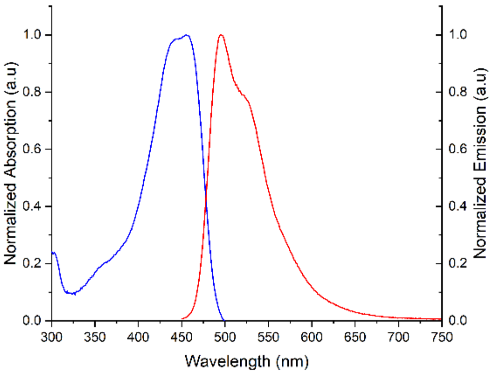

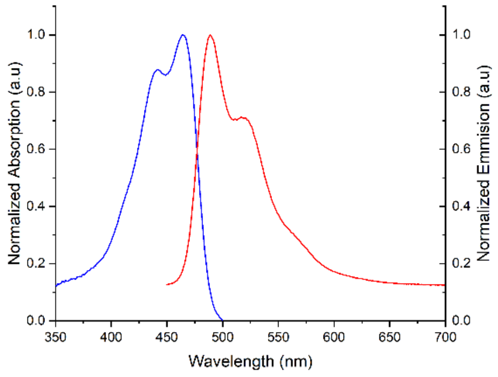

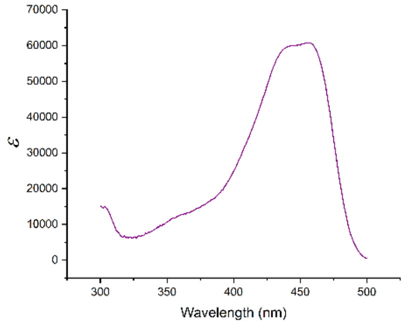

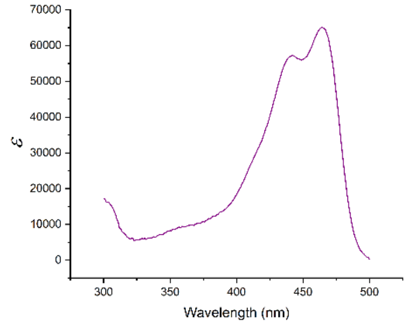

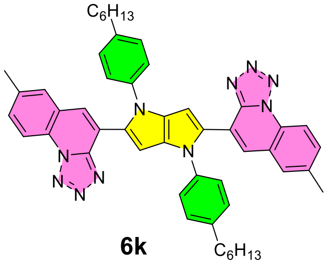

6k

**Ab**/**Em** and ε $(M^{-1}.cm^{-1})$ *in DCM*	**Ab**/**Em** and ε $(M^{-1}.cm^{-1})$ *in Toluene*

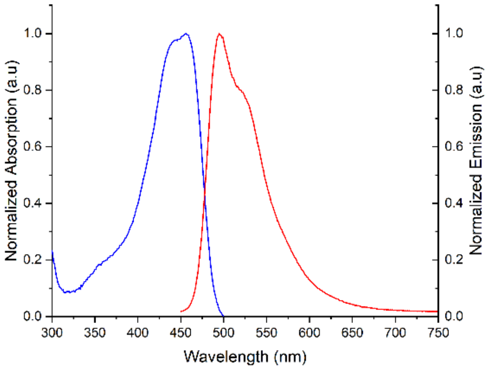

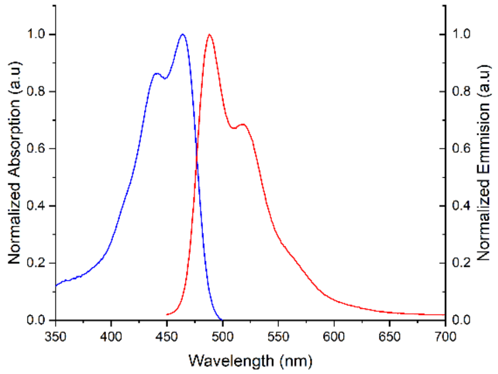

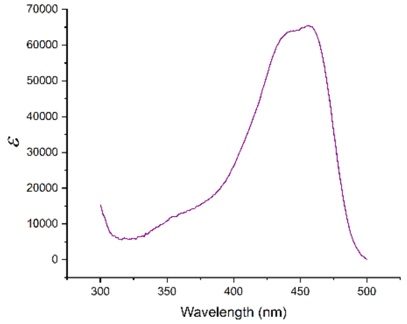

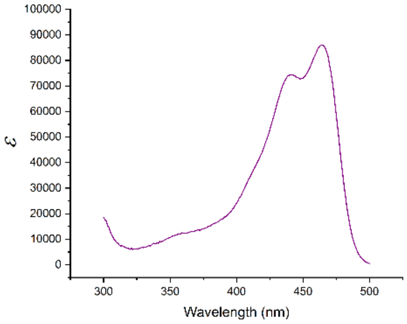

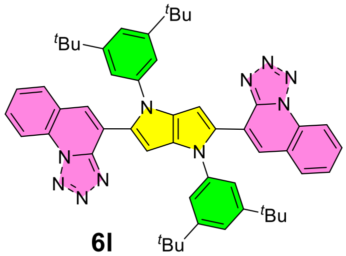

6l

**Ab**/**Em** and *ε* (M^{-1}.cm^{-1}) *in DCM*	**Ab**/**Em** and *ε* (M^{-1}.cm^{-1}) *in Toluene*
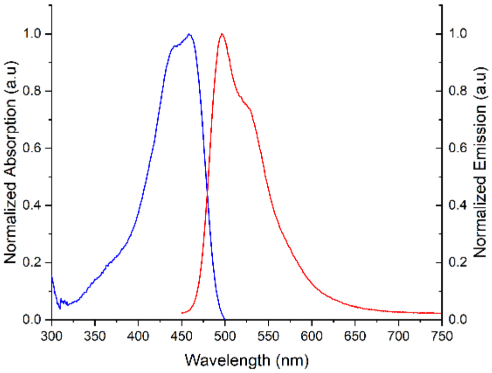	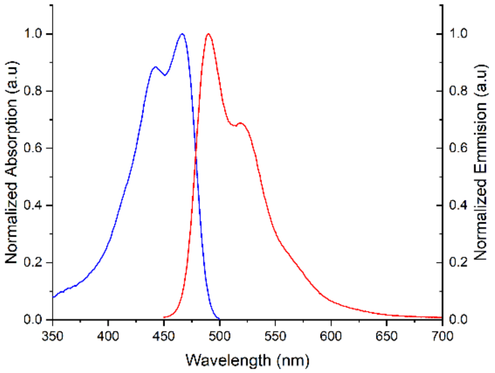
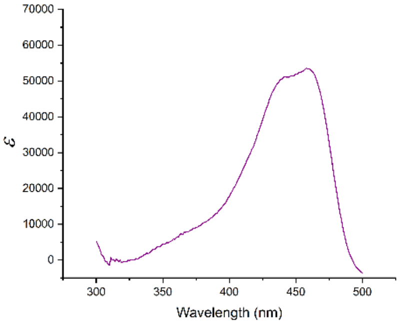	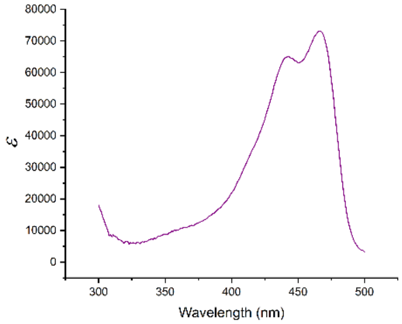

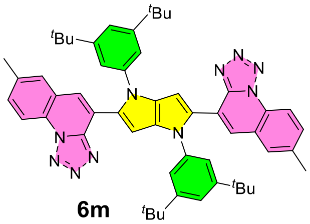

6m

**Ab**/**Em** and *ε* (M^{-1}.cm^{-1}) *in DCM*	**Ab**/**Em** and *ε* (M^{-1}.cm^{-1}) *in Toluene*

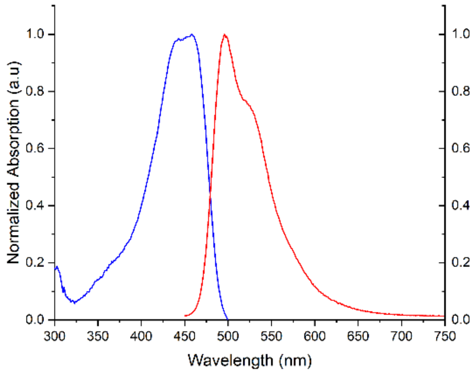

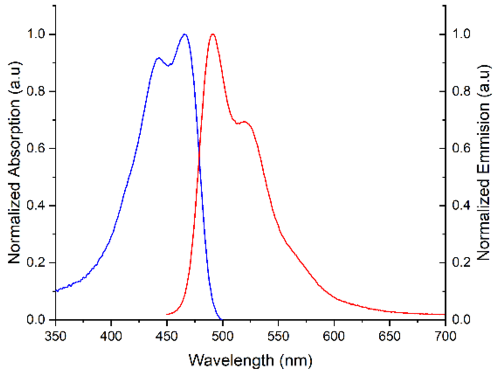

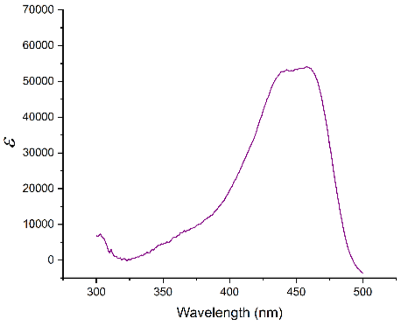

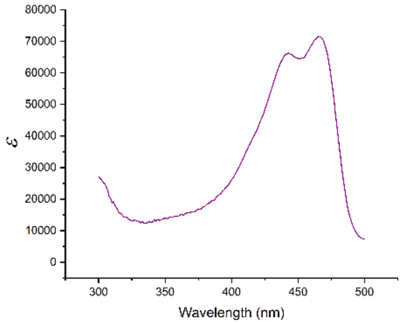

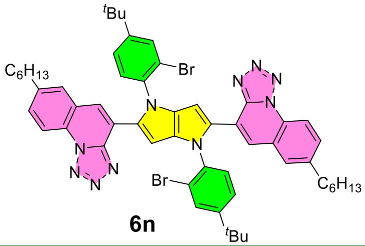

6n

Ab/**Em** and ***ε*** ($M^{-1}.cm^{-1}$)
in DCM

Ab/**Em** and ***ε*** ($M^{-1}.cm^{-1}$)
in Toluene

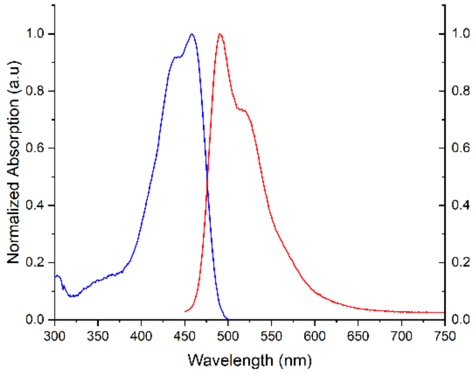

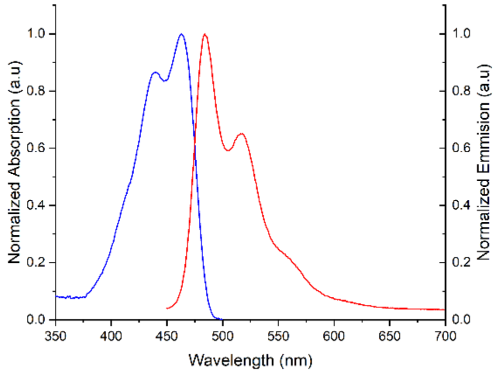

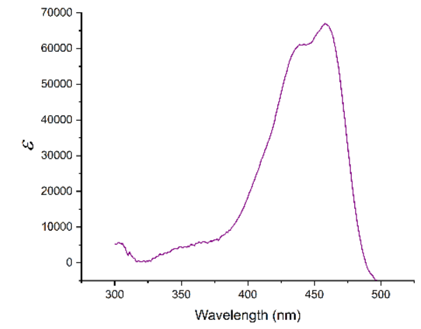

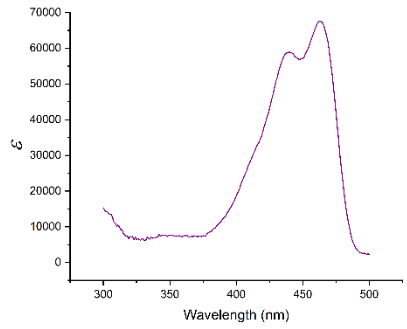

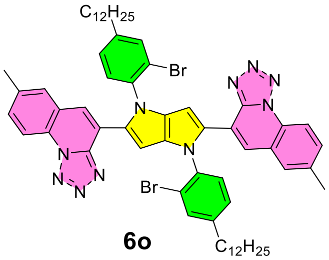

6o

**Ab**/**Em** and *ε* ($M^{-1}.cm^{-1}$) *in DCM*	**Ab**/**Em** and *ε* ($M^{-1}.cm^{-1}$) *in Toluene*

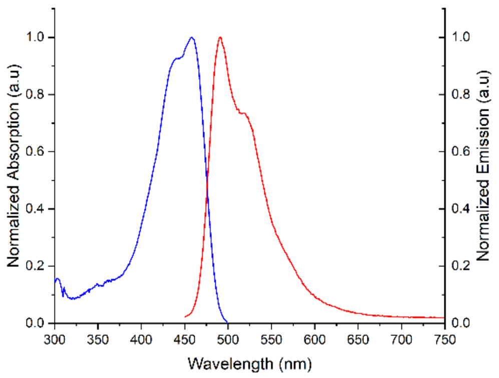

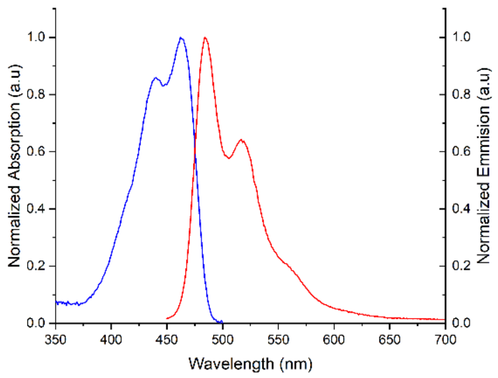

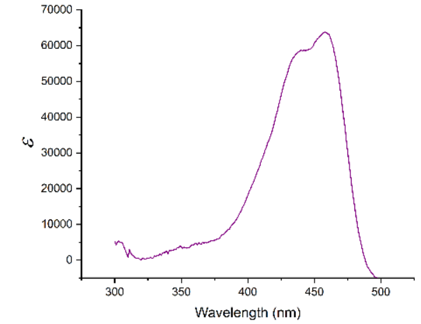

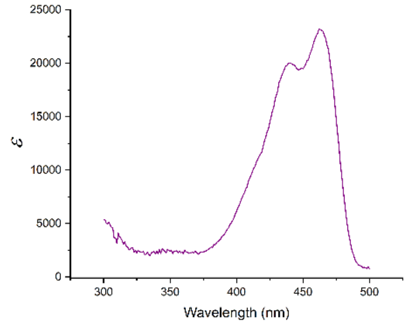

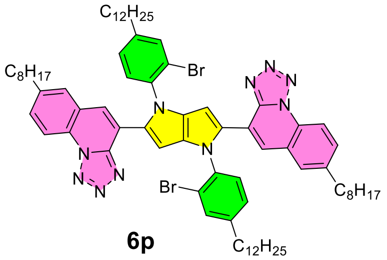

6p

Ab/Em and ε ($M^{-1}.cm^{-1}$) *in DCM*	Ab/Em and ε ($M^{-1}.cm^{-1}$) *in Toluene*
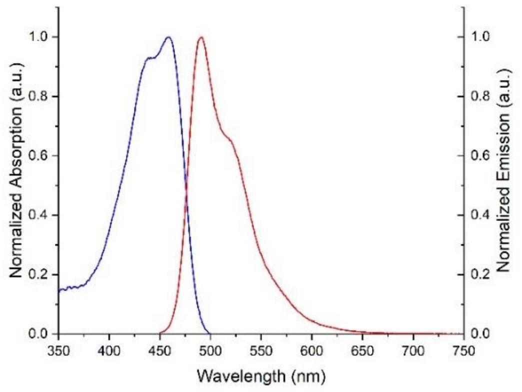	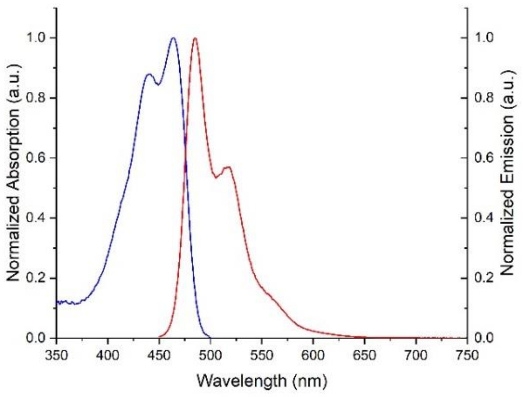
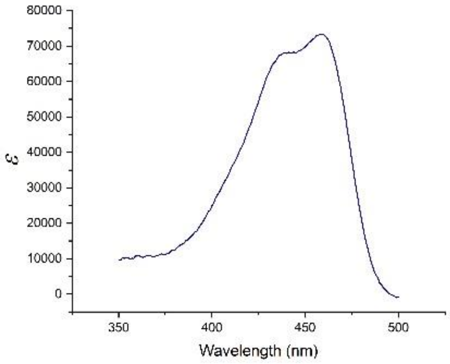	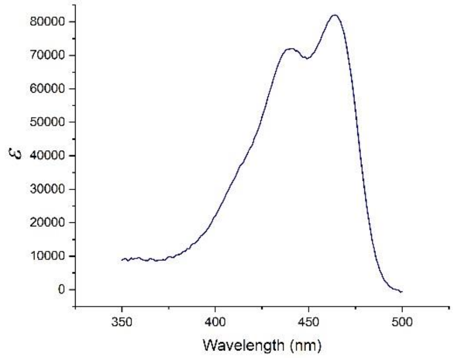

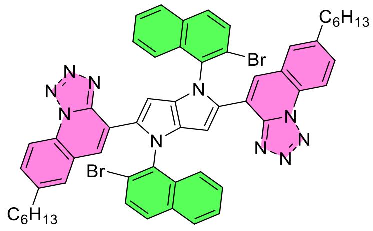

6q

Ab/Em and ε ($M^{-1}.cm^{-1}$) *in DCM*	Ab/Em and ε ($M^{-1}.cm^{-1}$) *in Toluene*
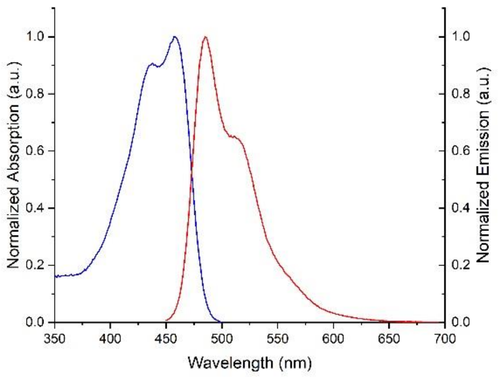	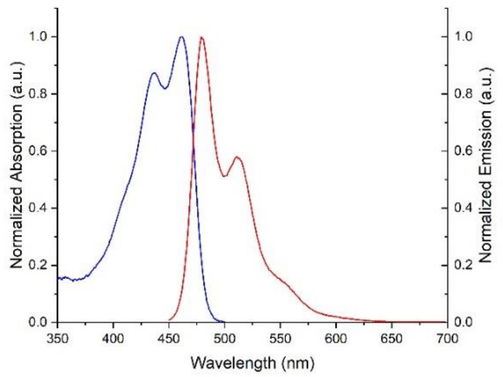

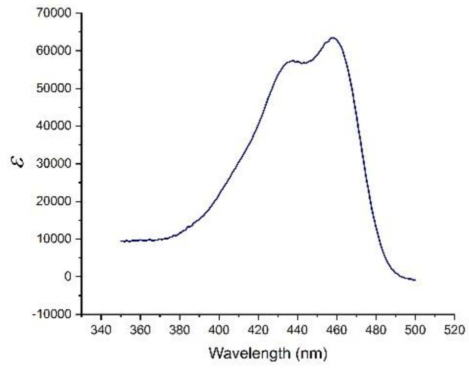

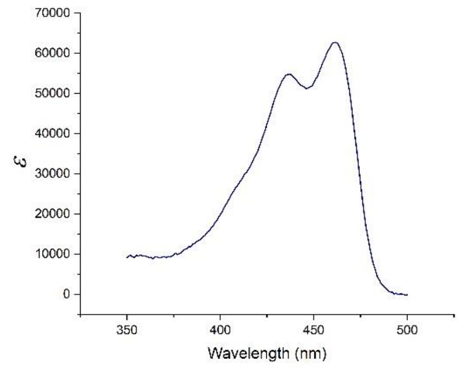

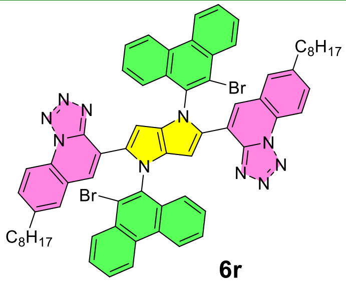

6r

**Ab**/**Em** and ***ε*** ($M^{-1}.cm^{-1}$) *in DCM*	**Ab**/**Em** and ***ε*** ($M^{-1}.cm^{-1}$) *in Toluene*

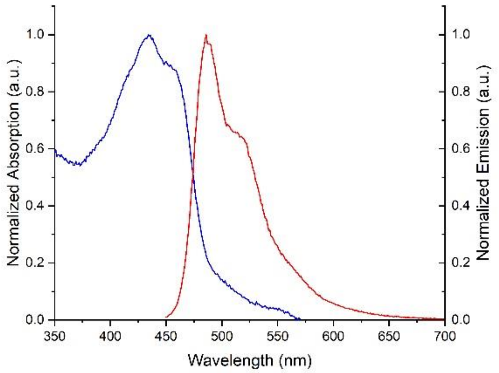

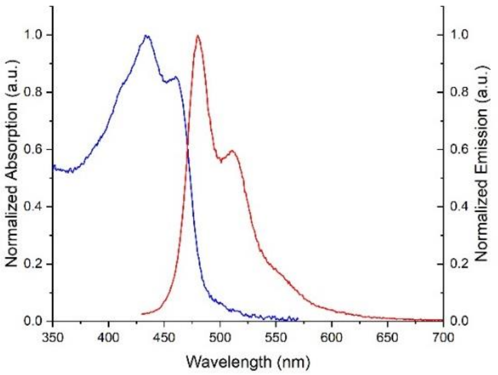

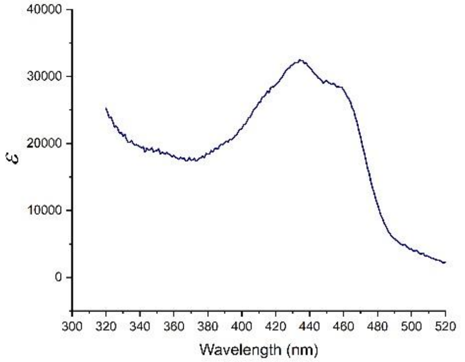

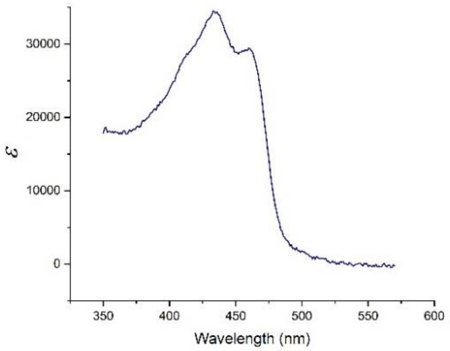

Table S2. Photophysical data for dyes **6a-6r**.

Entry	*in* DCM					*in* Toluene				
	$\lambda_{max\ (Ab)}$ (nm)	ε @ λ_{max} ($M^{-1}cm^{-1}$)	$\lambda_{max\ (Em)}$ (nm)	Stokes Shift (cm^{-1})	Φ_{fl}	$\lambda_{max\ (Ab)}$ (nm)	ε @ λ_{max} ($M^{-1}cm^{-1}$)	$\lambda_{max\ (Em)}$ (nm)	Stokes Shift (cm^{-1})	Φ_{fl}
**6a**	456	55400	495	1700	0.56	463	52100	488	1100	0.55
**6b**	456	61100	495	1700	0.57	463	8800	489	1100	0.63
**6c**	455	57700	493	1700	0.56	463	12500	485	1000	0.80
**6d**	464	61400	504	1700	0.52	474	65700	497	1000	0.59
**6e**	459	69300	491	1400	0.40	462	59800	484	1000	0.41
**6f**	458	63800	492	1500	0.40	462	16400	483	900	0.40
**6g**	458	58100	501	1900	0.55	459	7800	494	1500	0.51
**6h**	456	66900	495	1700	0.55	464	72300	488	1000	0.59

**6i**	456	63600	495	1700	0.57	464	87500	490	1100	0.58
**6j**	455	60900	495	1800	0.57	464	65100	489	1100	0.60
**6k**	456	65500	496	1800	0.55	464	86000	488	1100	0.58
**6l**	458	53600	496	1700	0.59	467	73100	490	1000	0.57
**6m**	458	54100	496	1700	0.59	465	71500	492	1200	0.54
**6n**	458	67000	490	1400	0.42	463	67700	484	900	0.42
**6o**	458	63800	491	1500	0.40	462	23200	484	1000	0.42
**6p**	459	73300	492	1500	0.37	464	82000	485	900	0.45
**6q**	458	63400	485	1200	0.17	461	62700	479	800	0.31
**6r**	434	32500	486	2500	0.13	460	34500	480	900	0.15

Optical properties of 10a-10c and 11a-11d

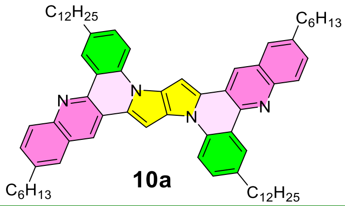

10a

**Ab**/**Em** and ε ($M^{-1}.cm^{-1}$) *in DCM*	**Ab**/**Em** and ε ($M^{-1}.cm^{-1}$) *in Toluene*

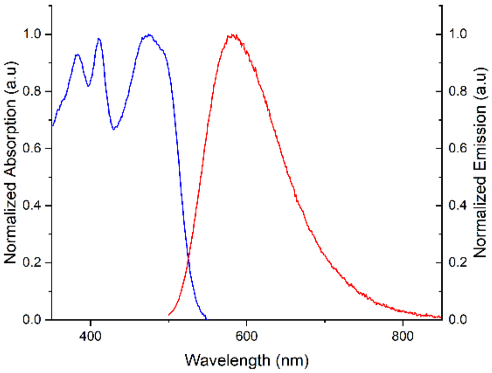

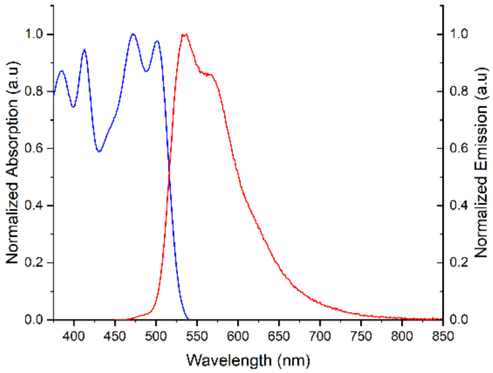

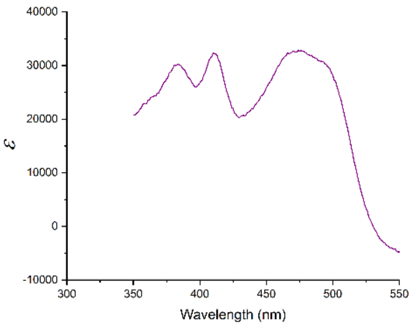

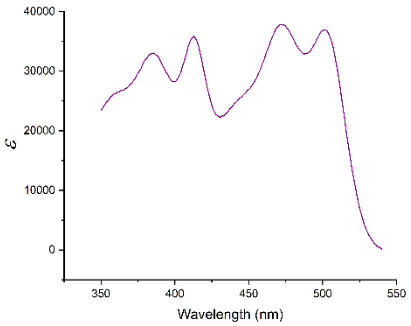

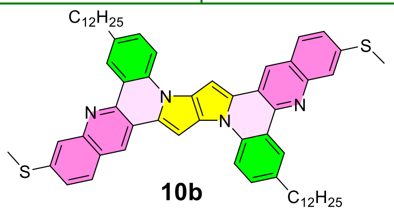

10b

**Ab**/**Em** and ε ($M^{-1}.cm^{-1}$) *in DCM*	**Ab**/**Em** and ε ($M^{-1}.cm^{-1}$) *in Toluene*

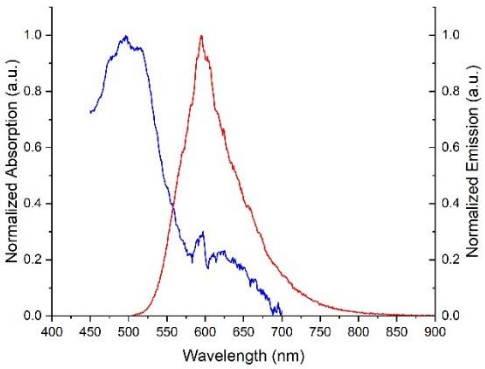

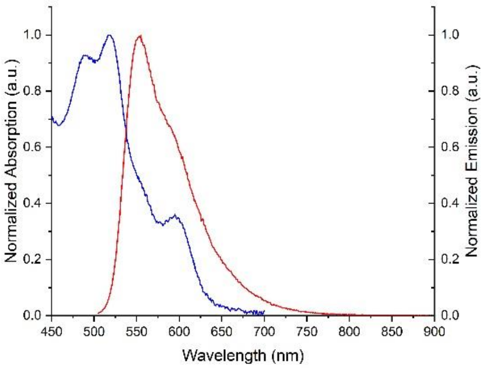

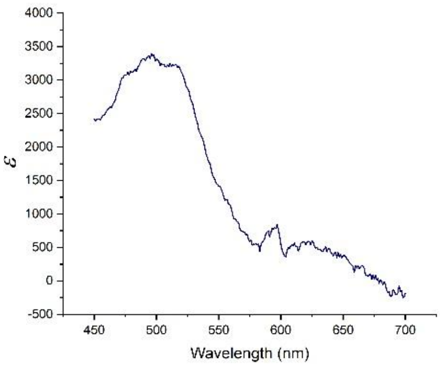

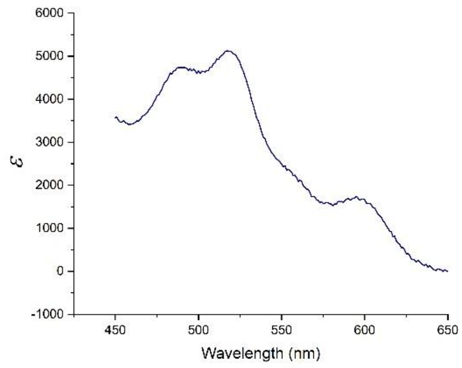

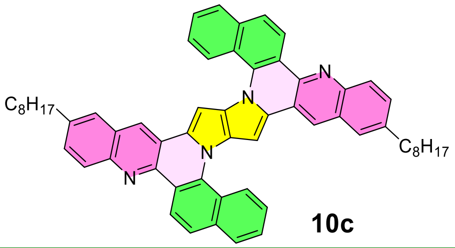

10c

**Ab**/**Em** and ***ε*** ($M^{-1}.cm^{-1}$) *in DCM*	**Ab**/**Em** and ***ε*** ($M^{-1}.cm^{-1}$) *in Toluene*

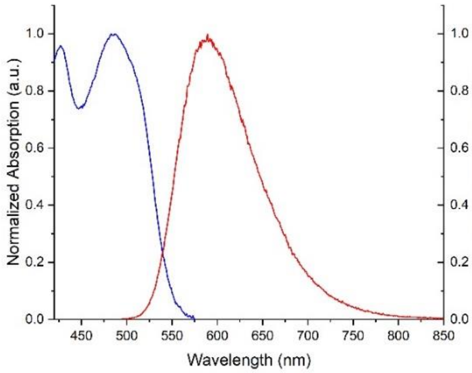

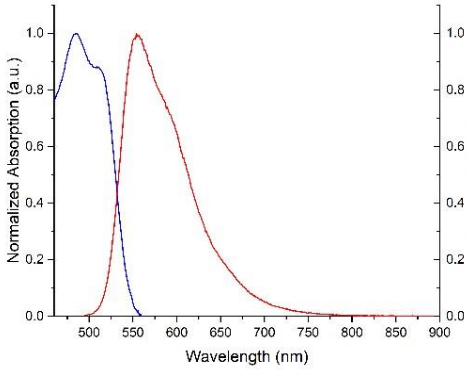

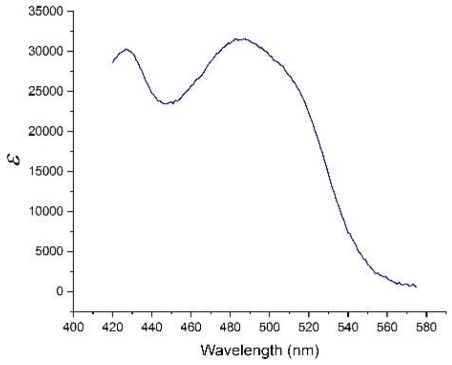

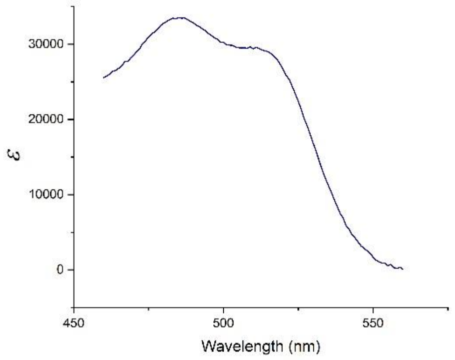

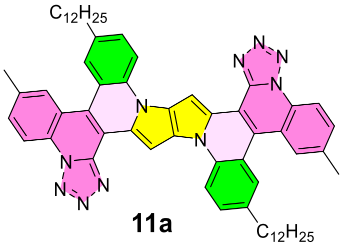

11a

Ab/Em and *ε* ($M^{-1}.cm^{-1}$) *in DCM*	Ab/Em and *ε* ($M^{-1}.cm^{-1}$) *in Toluene*

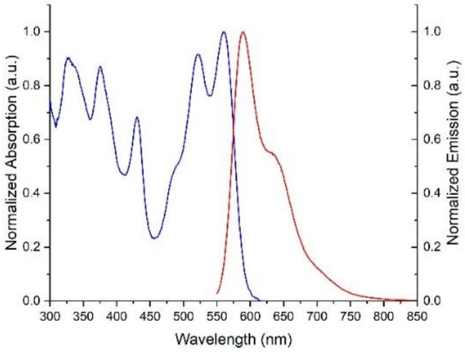

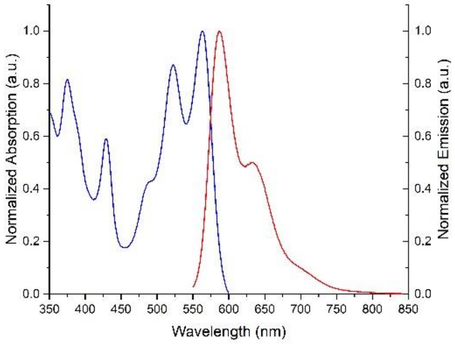

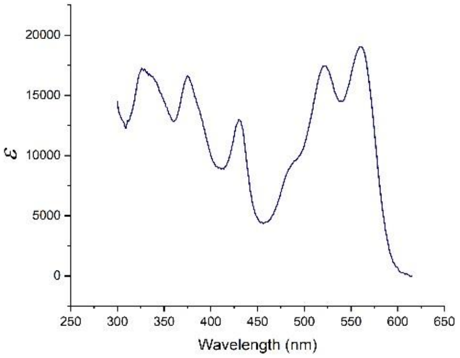

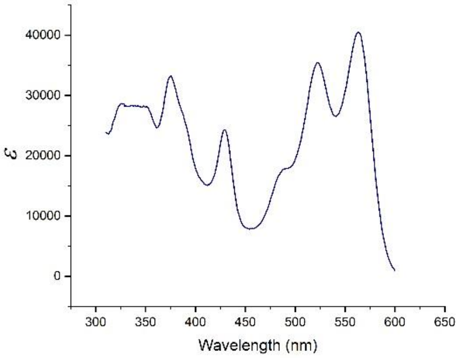

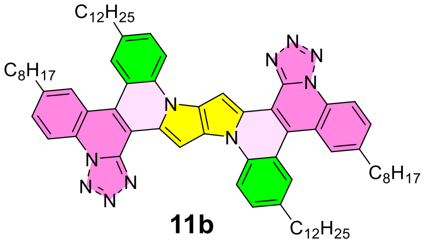

11b

Ab/Em and *ε* ($M^{-1}.cm^{-1}$) *in DCM*	Ab/Em and *ε* ($M^{-1}.cm^{-1}$) *in Toluene*

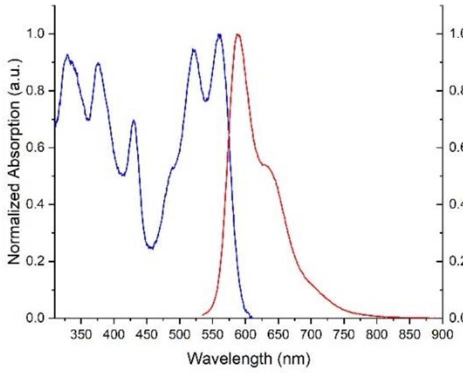

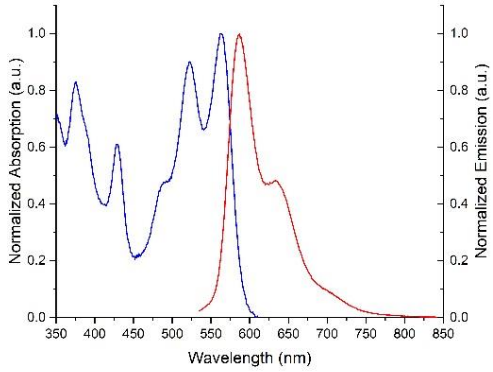

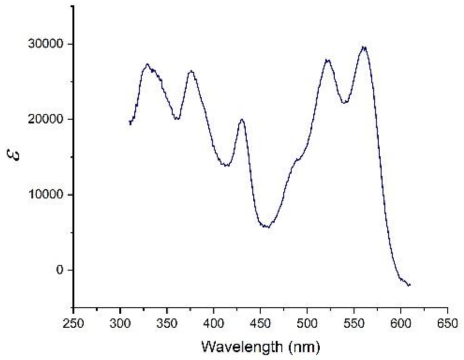

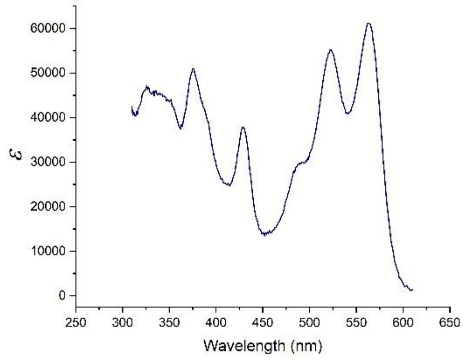

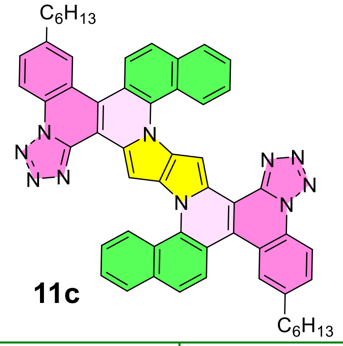

11c

**Ab**/**Em** and ***ε*** ($M^{-1}.cm^{-1}$) *in DCM*	**Ab**/**Em** and ***ε*** ($M^{-1}.cm^{-1}$) *in Toluene*

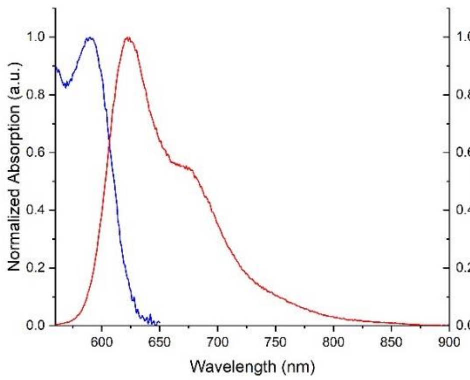

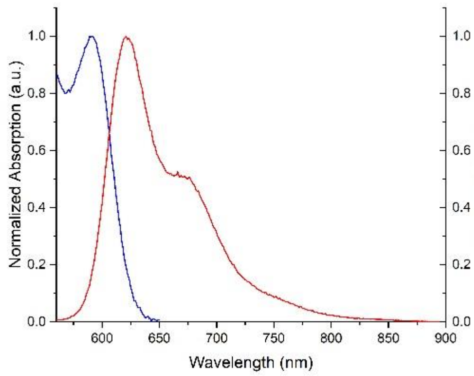

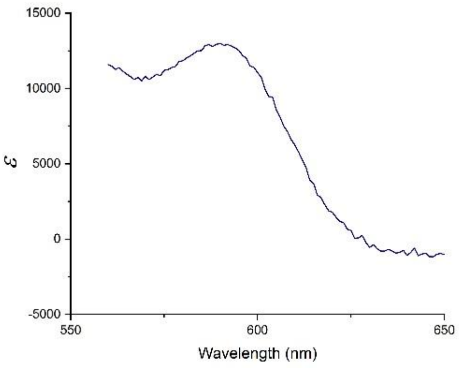

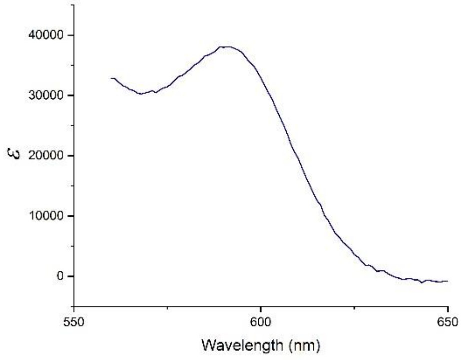

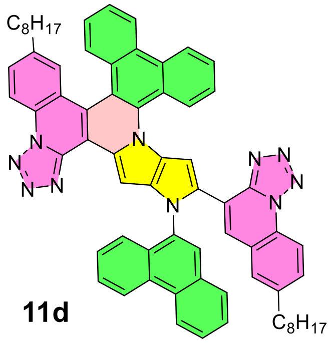

11d

**Ab**/**Em** and ***ε*** ($M^{-1}.cm^{-1}$) *in DCM*	**Ab**/**Em** and ***ε*** ($M^{-1}.cm^{-1}$) *in Toluene*

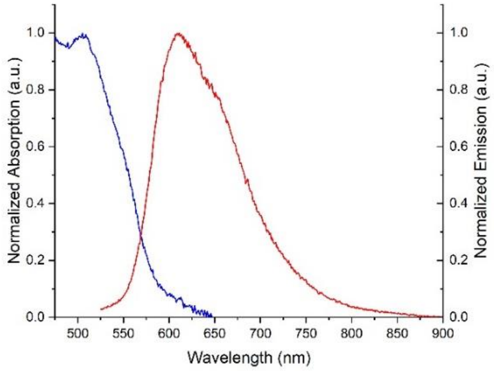

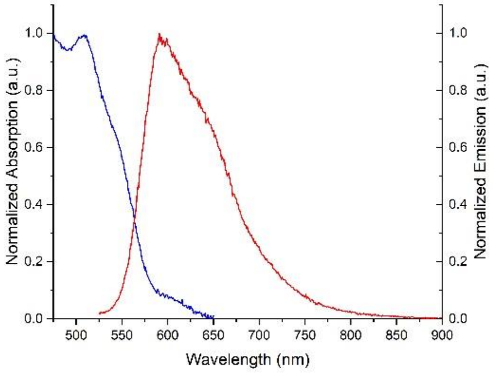

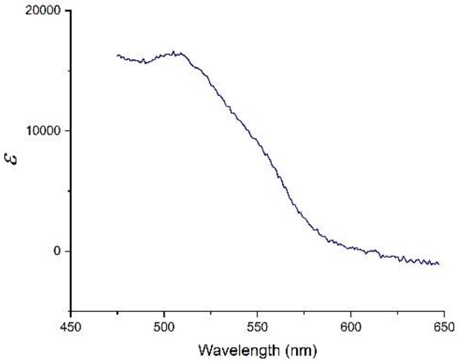

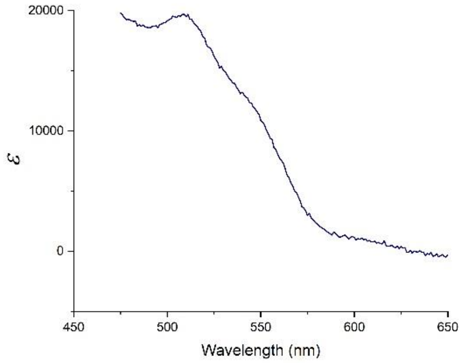

Table S3. Photophysical data for dyes **10a-10c** and **11a-11d**.

Entry	*in* DCM					*in* Toluene				
	λ_{max} (Ab) (nm)	ε @ λ_{max} ($M^{-1}cm^{-1}$)	λ_{max} (Em) (nm)	Stokes Shift (cm^{-1})	Φ_{fl}	λ_{max} (Ab) (nm)	ε @ λ_{max} ($M^{-1}cm^{-1}$)	λ_{max} (Em) (nm)	Stokes Shift (cm^{-1})	Φ_{fl}
**10a**	474	32800	581	3900	0.64	501	36900	532	1200	0.52
**10b**	496	3400	595	3400	0.04	517	5100	555	1300	0.02
**10c**	483	31600	589	3700	0.26	485	33500	554	2600	0.49
**11a**	561	19100	590	900	1.0	563	40500	586	700	0.79
**11b**	559	29700	587	900	0.80	562	61200	587	800	0.61
**11c**	590	13000	622	900	0.27	591	38100	621	800	0.45
**11d**	505	16600	611	3400	0.21	509	19700	591	2700	0.16

X-Ray crystallography analysis for compounds 4k, 6j and 10a:

The crystals of compounds **4k**, **6j** and **10a** were obtained from slow diffusion of acetonitrile into the dilute solution of these compounds in chloroform (vapor diffusion technique) after 4-7 days. The crystals selected for X-ray analysis had shapes, respectively, for compound **4k** with the formula $C_{54}H_{58}Cl_2N_4$ - pale yellow cubes with dimensions of 0.185 mm × 0.256 mm × 0.325 mm, for compound **6j** with the formula $C_{62}H_{74}N_{10}$- yellow parallelogram base prisms with dimensions

of 0.120 mm × 0.227 mm × 0.320 mm, and for compound **10a** with the formula $C_{72}H_{92}N_4$ - orange parallelogram base prisms with dimensions of 0.094 mm × 0.178 mm × 0.230 mm.

Crystal crystallographic data for samples **6j** and **10a** were collected at room temperature (T = 296(2) K), and the X-ray measurement of **4k** was performed at 149(2) K using a Bruker X8 APEXII diffractometer with Cu-Kα radiation (λ = 1.54178 Å). Frames were integrated with the Bruker SAINT [5] software package using a narrow-frame algorithm. The structures ware solved and refined using the Bruker SHELXTL Software Package [6, 7]. All obtained data were corrected for absorption effects using the face-indexed numerical method (SADABS) [8]. Same hydrogens were found from the difference electron density maps and refined with an anisotropic thermal motion model. Other hydrogen atoms were placed in calculated positions and refined as riding on their parent atoms with Uiso = 1.2 Ueq. The structure was solved by direct methods SHELXS-2014 [9] and refined with full-matrix least-squares calculations on F2 using SHELX-2014 [9]. All non-hydrogen atoms were refined anisotropically.

Crystallographic data have been deposited at the Cambridge Crystallographic Data Centre, 12 Union Road, 129 Cambridge CB2 1EZ, UK, and copies can be obtained on request, free of charge, by quoting the publication citation and the deposition number.

The details concerning the crystal data and structural parameters of **4k**, **6j** and **10a** are collected in **Table S4**.

Table S4. Data collection and structure refinement parameters for **4k, 6j** and **10a**.

	**4k**	**6j**	**10a**
**Identification code**	**MBT0001_Hex27Bu_1_B**	**TZDoDEC**	**DA2_A**
**CCDC Number**	**2297032**	**2297034**	**2297398**
**Chemical formula**	$C_{54}H_{58}Cl_2N_4$	$C_{62}H_{74}N_{10}$	$C_{72}H_{92}N_4$
**Formula weight**	833.94 g/mol	959.31 g/mol	1013.49 g/mol
**Temperature**	149(2) K	296(2) K	296(2) K
**Wavelength**	1.54178 Å	1.54178 Å	1.54178 Å
**Crystal size [mm]**	0.185×0.256×0.325	0.120×0.227×0.320	0.094×0.178×0.230
**Crystal habit**	colourless-yellow cubic	yellow plate	orange plate

**Crystal system**	monoclinic	triclinic	monoclinic
**Space group**	P 1 21/n 1	$P\bar{1}$	P 1 21/c 1
**Unit cell dimensions**			
a **[Å]**<br>***b*** **[Å]**<br>***c*** **[Å]**	11.1622(2)<br>18.3700(4)<br>11.8712(2)	8.8290(12)<br>11.0358(16)<br>15.492(2)	26.2982(15)<br>9.3916(5)<br>12.6474(7)
α **[°]**<br>***β*** **[°]**<br>***γ*** **[°]**	90<br>109.8350(10)<br>90	76.637(8)<br>88.102(8)<br>70.107(8)	90<br>102.618(4)<br>90
**Volume [Å^3]**	2289.77(8)	1379.3(3)	3048.2(3)
**Z**	2	1	2
**Density (calculated) [g/cm^3]**	1.210	1.155	1.104
**Absorption coefficient [mm^{-1}]**	1.578	0.533	0.474
**F(000)**	888	516	1104
**Theta range [°]**	4.63 to 68.58	2.94 to 64.29	1.72 to 52.34
**Index ranges**	-13<=h<=12,<br>-22<=k<=21,<br>-14<=l<=14	-9<=h<=10,<br>-11<=k<=12,<br>-16<=l<=17	-26<=h<=19,<br>-7<=k<=7,<br>-10<=l<=11
**Reflections collected**	32275	27781	14790
**Independent reflections**	4190 [R(int) = 0.0882]	4175 [R(int) = 0.0671]	2290 [R(int) = 0.0888]
**Coverage of independent reflections**	99.4%	90.3%	66.1%
**Absorption correction**	numerical	numerical	numerical
**Max. and min. transmission**	0.7590 and 0.6280	0.9390 and 0.8480	0.9570 and 0.8990
**Structure solution technique**	direct methods		
**Structure solution program**	SHELXL-2014 (Sheldrick, 2014)		
**Refinement method**	Full-matrix least-squares on F^2		
**Refinement program**	SHELXL-2014 (Sheldrick, 2014)		
**Function minimized**	$\Sigma\ w(F_o^2 - F_c^2)^2$		
**Data / restraints / parameters**	4190 / 0 / 365	4175 / 0 / 355	2290 / 2 / 346
**Goodness-of-fit on F_2**	1.027	1.026	1.032
**Δ/σ_{max}**	-	-	0.004
**Final R indices**<br>**R_1,**<br>**wR_2**	3265 data; I>2σ(I)<br>0.0498,<br>0.1025	1985 data; I>2σ(I)<br>0.0715<br>0.1277	1008 data; I>2σ(I)<br>0.0953,<br>0.1986
**R indices (all data):**	R1 = 0.0695,<br>wR2 = 0.1117	R1 = 0.1729,<br>wR2 = 0.1635	R1 = 0.2280,<br>wR2 = 0.2757
**Weighting scheme**	$w=1/[\sigma^2(F_o^2)+(0.0325P)^2+2.1377P]$	$w=1/[\sigma^2(F_o^2)+(0.0483P)^2+0.6715P]$	$w=1/[\sigma^2(F_o^2)+(0.1456P)^2]$
	where $P=(F_o^2+2F_c^2)/3$		

**Extinction coefficient**	-	-	0.0041(6)
**Largest diff. peak and hole** [eÅ^{-3}]	0.261 and -0.261	0.202 and -0.218	0.393 and -0.533
**R.M.S. deviation from mean** [eÅ^{-3}]	0.051	0.037	0.144

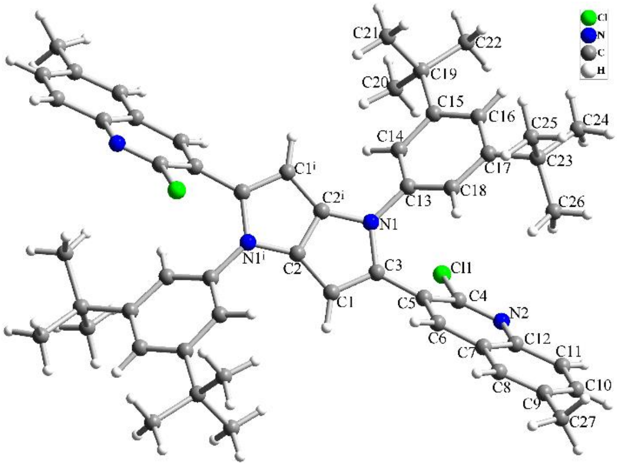

Fig S1. X-ray crystal structure of compound **4k** (CCDC Number: **2297032**) with atom labeling scheme.

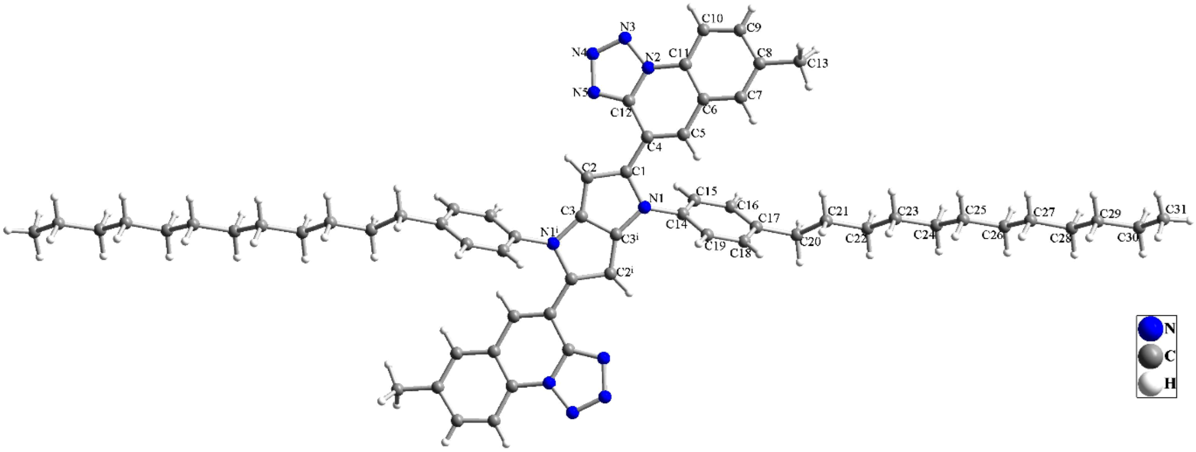

Fig S2. X-ray crystal structure of compound **6j** (CCDC Number: **2297034**) with atom labeling scheme.

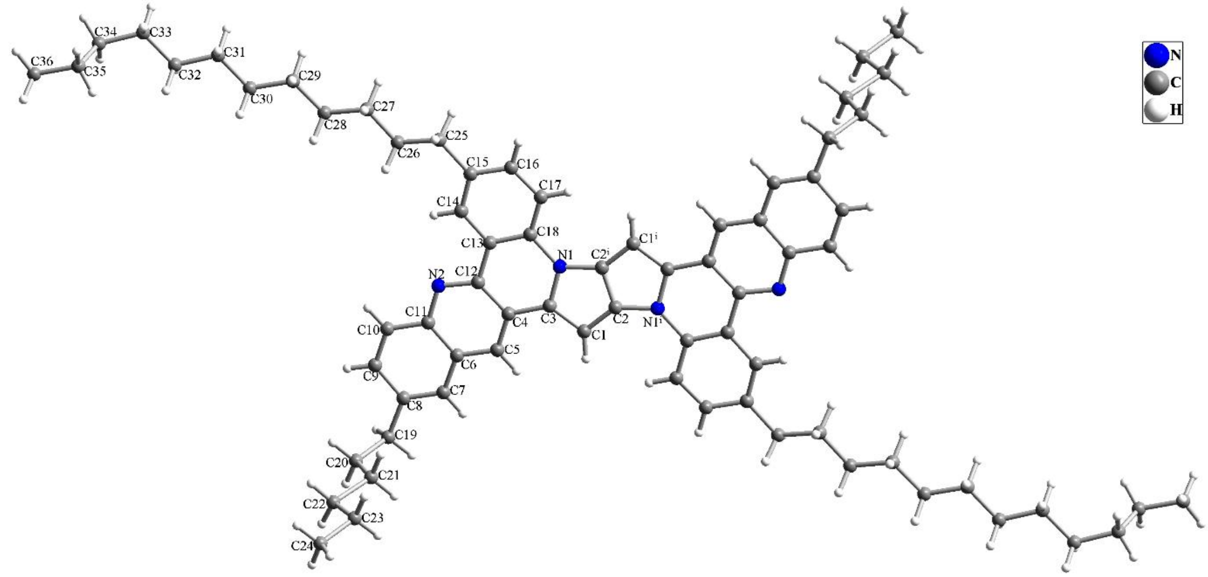

Fig S3. X-ray crystal structure of compound **10a** (CCDC Number: **2297398**) with atom labeling scheme.

Details of single crystal X-ray analysis of 11d:

The red parallelogram base prism shape crystals of compound **11d** were obtained from slow diffusion of acetonitrile into the dilute solution of **11d** in chloroform (vapor diffusion technique) after 5 days. The X-ray measurement of **11d** was performed at 130.0(5) K on a Bruker D8 Venture PhotonII diffractometer equipped with a INCOATEC IμS micro-focus source (CuKα, $\lambda = 1.54178$ Å) and a mirror monochromator. A total of 6171 frames were collected with Bruker APEX3 program [10]. The frames were integrated with the Bruker SAINT software package [11] using a narrow-frame algorithm. The integration of the data using a triclinic unit cell yielded a total of 46151 reflections to a maximum θ angle of 66.67° (0.84 Å resolution), of which 9404 were independent (average redundancy 4.908, completeness = 99.5%, $R_{int} = 4.92\%$, $R_{sig} = 3.50\%$) and 7557 (80.36%) were greater than $2\sigma(F^2)$. The final cell constants of $a = 12.7150(4)$ Å, $b = 13.0817(4)$ Å, $c = 17.2329(6)$ Å, $\alpha = 69.324(2)°$, $\beta = 87.123(2)°$, $\gamma = 84.459(2)°$, $V = 2668.86(15)$ Å^3, are based upon the refinement of the XYZ-centroids of 9891 reflections above 20 $\sigma(I)$ with $5.482° < 2\theta < 133.1°$. Data were corrected for absorption effects using the Multi-Scan method (SADABS) [12]. The ratio of minimum to maximum apparent transmission was 0.893. The calculated minimum and maximum transmission coefficients (based on crystal size) are 0.921 and 0.980.

The structure was solved and refined using SHELXTL Software Package [7] using the space group $P\bar{1}$, with $Z = 2$ for the formula unit, $C_{68}H_{60}N_{10}$. The final anisotropic full-matrix least-squares refinement on F^2 with 1076 variables converged at $R1 = 5.17\%$, for the observed data and $wR2 = 12.91\%$ for all data. The goodness-of-fit was 1.064. The largest peak in the final difference electron density synthesis was 0.325 $e^-/Å^3$ and the largest hole was -0.356 $e^-/Å^3$ with an RMS deviation of 0.037 $e^-/Å^3$. On the basis of the final model, the calculated density was 1.266 g/cm^3 and $F(000)$, 1076 e^-. All the details concerning the crystal data and structural parameters of **11d** are presented in **Table S5**.

The crystal lattice of **11d** as an asymmetric part contains fully disordered organic molecule. There are no additional solvent species present. Fragment of the molecule consisting of aromatic rings and one of the alkyl chains is disordered over two positions with refined occupancy ratio yielding 0.829(2):0.171(2) for main and B residues respectively. The second alkyl chain is disordered over four positions with refined occupancies equal 0.663(2), 0.166(2), 0.098(2) and 0.072(2) for main residue, D, B and C moieties respectively. The disorder of the molecule together with the occupancies of alternative positions is presented in **Figure S4**.

To preserve reasonable bonds and angles values in the disordered fragments a number of geometry restraints was used including restrains for atomic displacement parameters. All ordered non-hydrogen atoms and major component (occupancy higher than 0.5) disordered moieties were refined anisotropically. All hydrogen atoms were placed in calculated positions and refined within the riding model. Their temperature factors were not refined and were set to be either 1.2 (C_{ar}-H, CH_2 moieties) or 1.5 (methyl groups) times larger than U_{eq} of the corresponding heavy atom. The atomic scattering factors were taken from the International Tables [13]. Molecular graphics was prepared using program Mercury 4.1 [14]. Thermal ellipsoids parameters are presented at 50% probability level in **Figure S5** whereas packing of the molecules in the crystal lattice is given in **Figure S6**.

The structural data have been deposited at the Cambridge Crystallographic Data Center (CCDC No 2297035). These data can be obtained free of charge from The Cambridge Crystallographic Data Center via www.ccdc.cam.ac.uk/structures.

Table S5. Data collection and structure refinement for **11d** (CCDC No 2297035).

**Formula**	$C_{68}H_{60}N_{10}$	
**M_x/ g·mol^{-1}**	1017.26	
**T/ K**	130.0(5)	
**λ/ Å**	1.54178	
**Crystal size**	0.034 × 0.091 × 0.142 mm	
**Space group**	$P\bar{1}$	
**Unit cell dimensions**	a = 12.7150(4) Å	α = 69.324(2)°
	b = 13.0817(4) Å	β = 87.123(2)°
	c = 17.2329(6) Å	γ = 84.459(2)°
**V/ Å^3, Z**	2668.86(15), 2	
**D_x/ g·cm^{-3}**	1.266	
**μ/ mm^{-1}**	0.591	
**F(000)**	1076	
**θ_{min}, θ_{max}**	2.74°, 66.67°	
**Index ranges (merged data)**	$-15 \leq h \leq 15$, $-15 \leq k \leq 15$, $-20 \leq l \leq 20$	
**Reflections collected/ independent**	46151/ 9404	
	R_{int} = 0.0492	
**Completeness**	99.5%	
**Absorption correction**	Multi-Scan	
**T_{max}, T_{min}**	0.980, 0.921	
**Structure solution technique**	direct methods	
**Refinement method**	Full-matrix LSQ on F^2	
**Data / restraints / parameters**	9404 / 458 / 1076	
**GOF on F^2**	1.064	
**Final R indices**	7557 data; $I>2\sigma(I)$	
	$R1$ = 0.0517, $wR2$ = 0.1217	
	all data	
	$R1$ = 0.0665, $wR2$ = 0.1291	
**$\Delta\rho_{max}$, $\Delta\rho_{min}$**	0.325, -0.356 e·Å^{-3}	

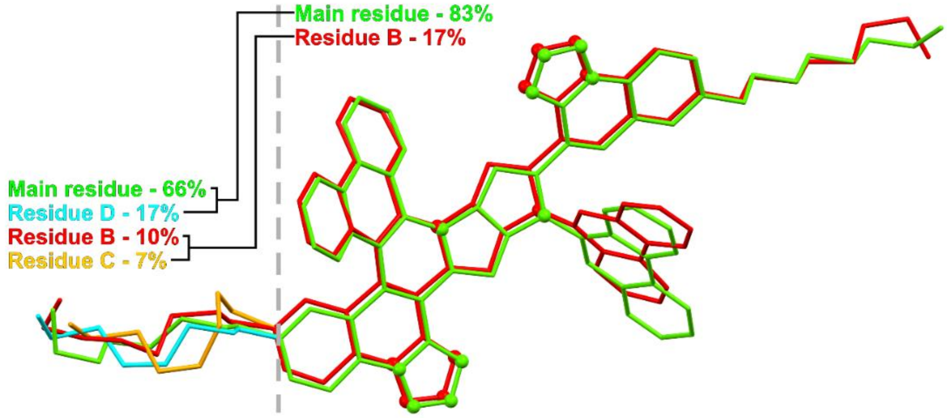

Fig S4. Disorder in the **11d** crystal structure. H atoms omitted; N atoms presented as spheres.

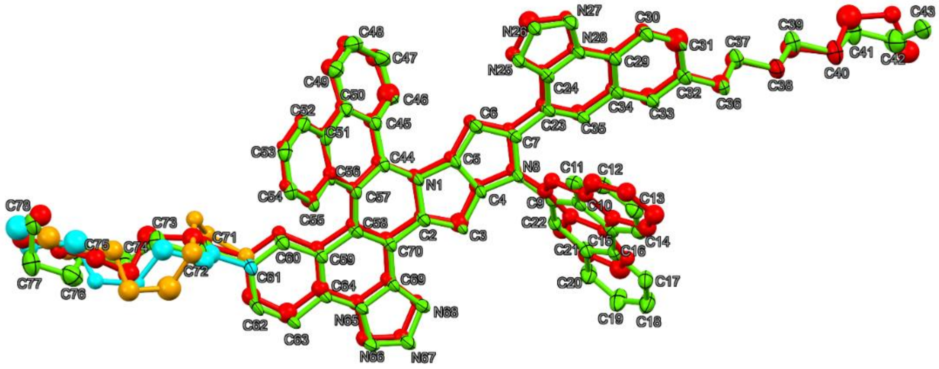

Fig S5. Thermal ellipsoid plot at 50% probability level together with numbering scheme of main residue atoms in the structure of **11d**. H atoms omitted for clarity. Color codes the same as in **Fig S4**.

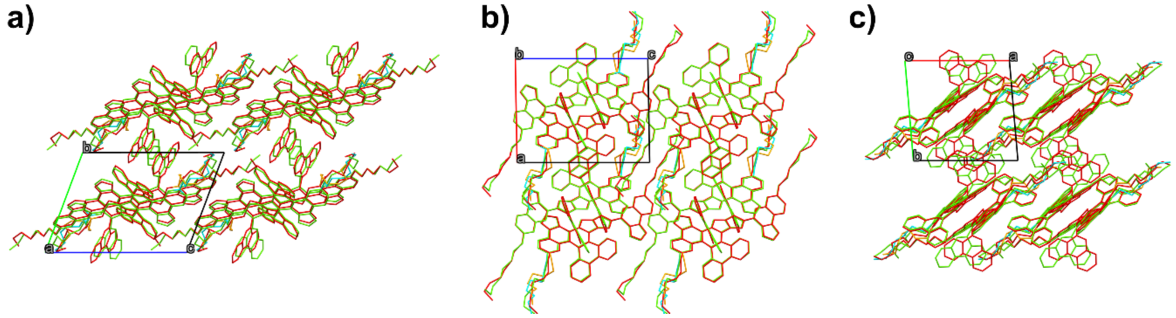

Fig S6. Packing diagram of **11d**, view along [100] a), [010] b) and [001] c). Color codes the same as in **Fig S4**.

Theoretical calculations

Table S6. The energies and oscillator strengths of the S_0->S_i and S_1->S_0 transitions calculated with use of the different functionals.

molecule	solvent	abs		flu		Stokes
		[nm]	f	[nm]	f	[cm^{-1}]
**M06**						
**6**	TOL	470.6	1.7056	531.4	1.9298	2429
	$CHCl_3$	477.7	1.7983	550.9	2.0604	2779
	ACN	484.0	1.8948	573.9	2.1953	3236
**4**	TOL	421.7	0.7415	483.1	1.0236	3012
	$CHCl_3$	423.6	0.8149	492.2	1.2886	3288
	ACN	424.6	0.8857	505.3	1.5498	3764
**CAM-B3LYP**						
**6**	TOL	396.7	1.8279	479.5	2.0959	4356
	DCM	399.4	1.9626	504.8	2.2849	5228
**4**	TOL	343.5	1.1010	423.4	1.7554	5493
	DCM	345.0	1.2519	441.6	1.9812	6340
**B3LYP**						
**6**	HEX	478.3	1.6397	528.2	1.7751	1975
	ACN	495.7	1.8124	575.9	2.1211	2809
**4**	HEX	446.3	0.6383	610.9	0.0026	6037
	ACN	452.6	0.8033	582.9	0.0258	4938

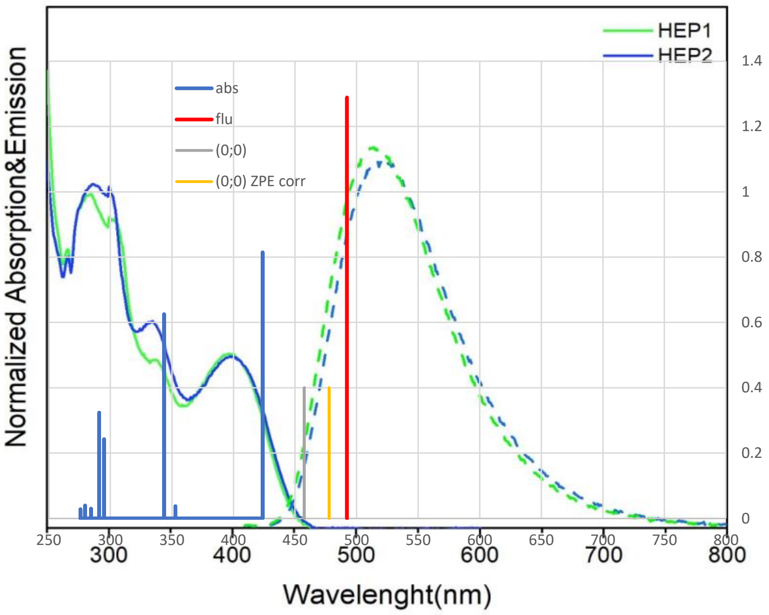

Fig. S7. Experimental absorption and fluorescence spectra of NCl-PP-NCl in chloroform and the results of TDDFT M06/6-31G(d,p) calculations - absorption S_o->S_i (blue lines) and fluorescence S_1->S_0 (red line).

Table S7. Characteristics of molecules - components of ADA systems, considered in the work. Results of M06/6-31G(d,p) calculations. the energies and shape of the HOMO and LUMO orbitals, the energies, and the oscillator strength for electronic excitation

	ε_{HOMO}	ε_{LUMO}	S_0->S_1		
	[eV]	[eV]	[eV]	[nm]	f
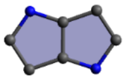	-5.11<br>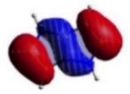	1.00<br>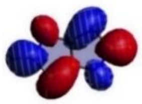	5343	232.1	0.4251
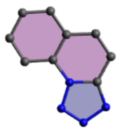	-7.07<br>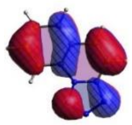	-1.73<br>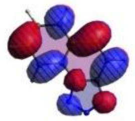	4.34	282.2	0.1824
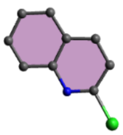	-6.88<br>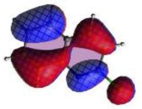	-1.55<br>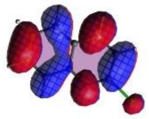	4.358	284.5	0.0568

The shapes of the orbitals of consided ADA sysyems can be described as:

$$\Psi_{LUMO} \approx a^{*}\varphi_{LUMO}(A_1, A_2) + d^{*}\varphi_{LUMO}(D) + c^{*}\phi(R_1, R_2) + ...$$

$$\Psi_{HOMO} \approx a\varphi_{HOMO}(A_1, A_2) + d\varphi_{HOMO}(D) + c\varphi(R_1, R_2) + ...$$

$$M_{HOMO/LUMO} = \langle \Psi_{HOMO} | R | \Psi_{LUMO} \rangle \approx aa^{*}M(A_1, A_2) + dd^{*}(D) + cc^{*}M(R_1, R_2) + ...$$

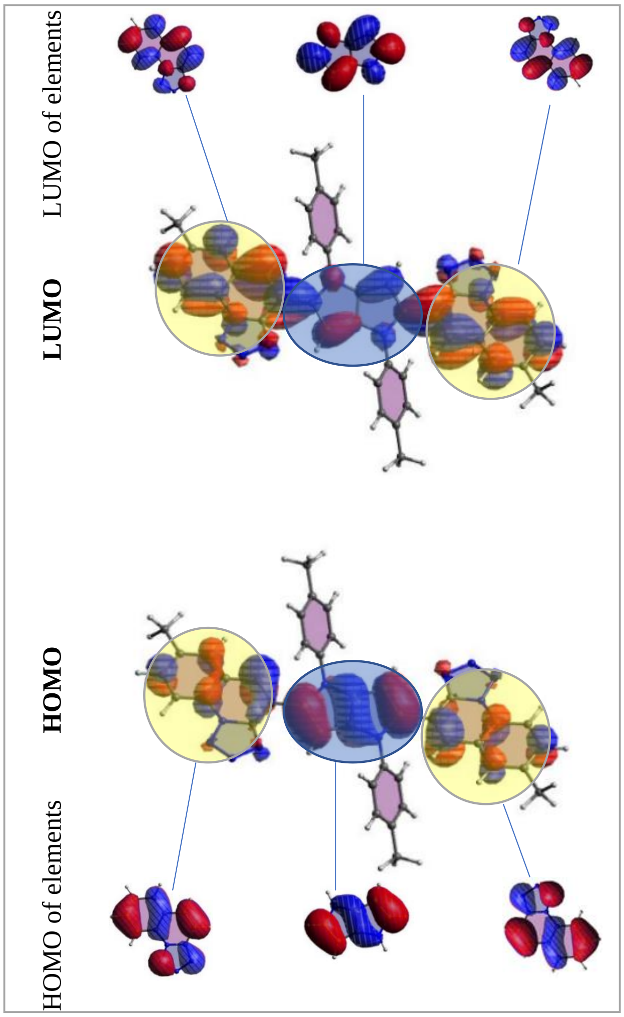

Fig. S8. Structure of HOMO and LUMO orbitals of compound **4.** In this case *c* and *c** are 0.

Table S8. Comparison of properties of isomers **10** and **(10-x)**

	**10**<br>Abs. 489 nm f=1.203<br>Flu. 563 nm f= 1.286	**(10-x)**<br>Abs. 510 nm f= 0.748<br>Flu. 603 nm f = 0.783
	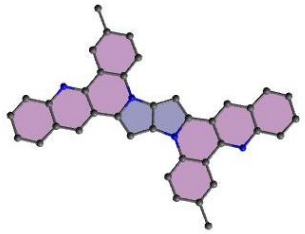	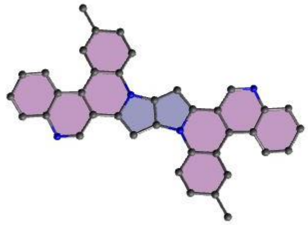
**LUMO**	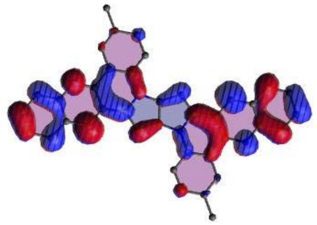	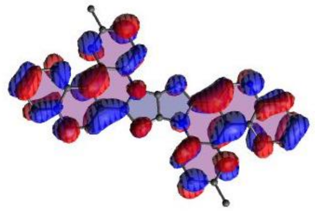
**HOMO**	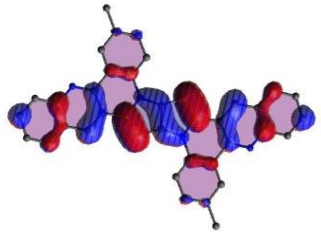	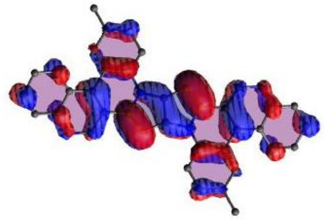

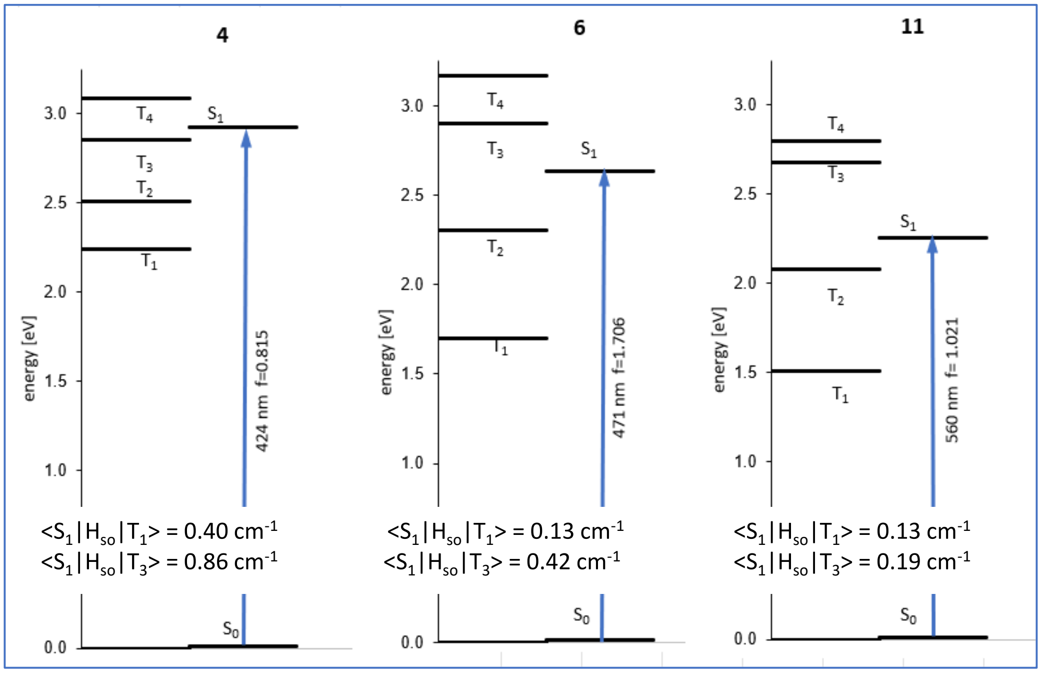

Fig. S9. Diagrams of electronic states and SOC elements.

Optical properties of compounds 4d, 4h, 4i, 4k, 6a, 6e, 6i, and 6o (in solid state)

Compound	Structure	Spectrum
**4d**	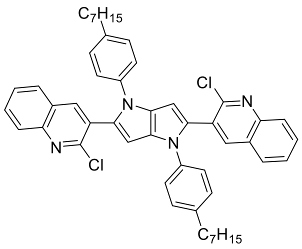	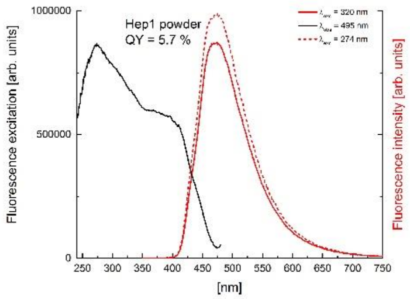
**4h**	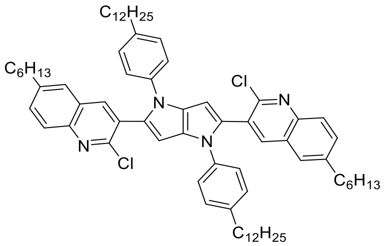	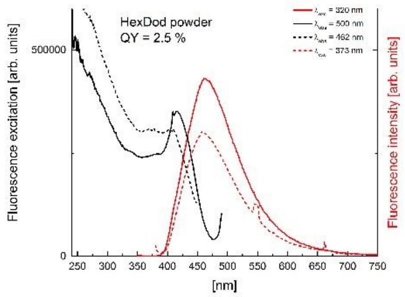
**4i**	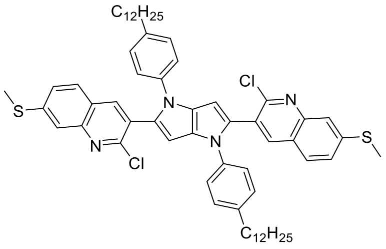	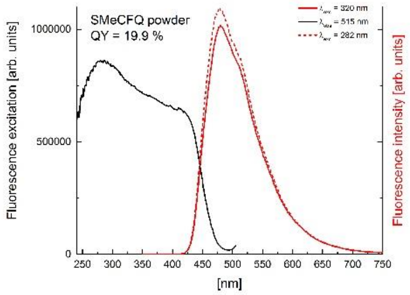
**4k**	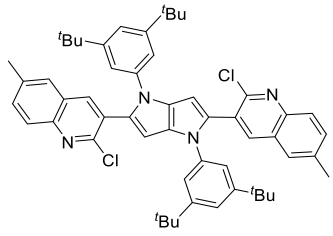	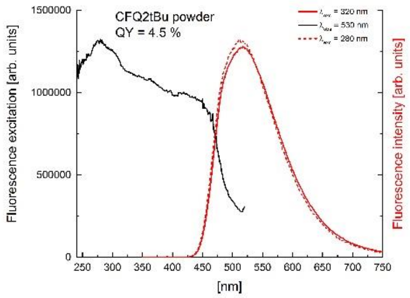

**6a**	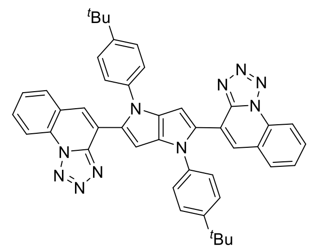	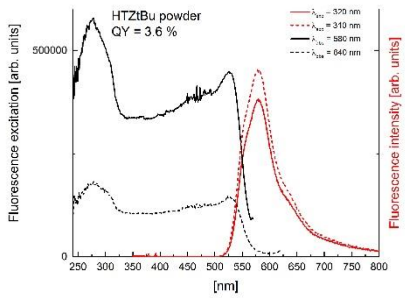HTZtBu powder<br>QY = 3.6 %<br>Fluorescence excitation [arb. units]<br>Fluorescence intensity [arb. units]<br>[nm]
**6e**	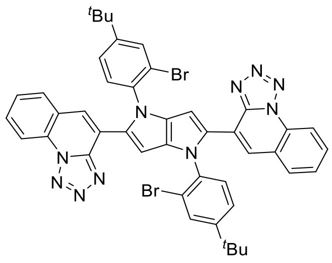	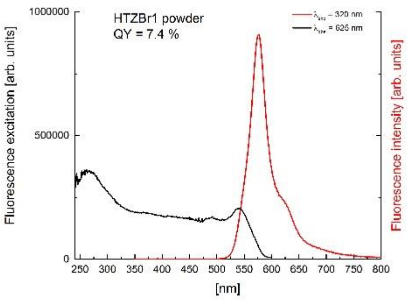HTZBr1 powder<br>QY = 7.4 %<br>Fluorescence excitation [arb. units]<br>Fluorescence intensity [arb. units]<br>[nm]
**6i**	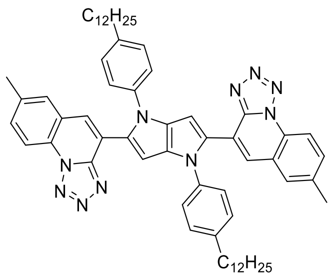	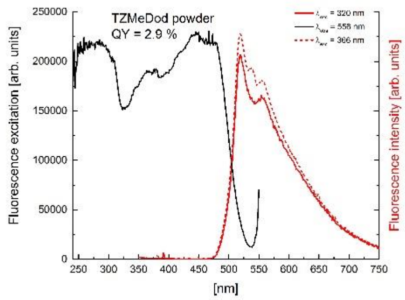TZMeDod powder<br>QY = 2.9 %<br>Fluorescence excitation [arb. units]<br>Fluorescence intensity [arb. units]<br>[nm]
**6o**	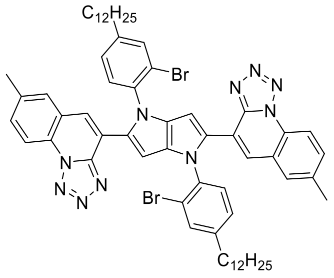	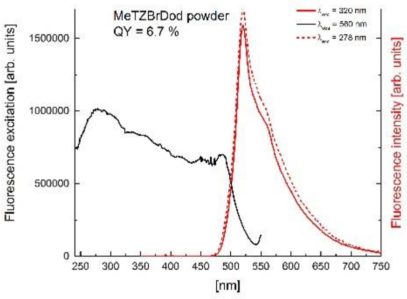MeTZBrDod powder<br>QY = 6.7 %<br>Fluorescence excitation [arb. units]<br>Fluorescence intensity [arb. units]<br>[nm]

References

[1] (a) Meth-Cohn, O.; Narine, B.; Tarnowski, B. *Tet. Lett.* **1979**, *20*, 3111-3114. (b) Meth-Cohn, O.; Narine, B.; Tarnowski, B. *J.* Chem. *Soc., Perkin Trans. 1,* **1981**,1531-1536.

[2] Sonar, S. S.; Sadaphal, S. A.; Pokalwar, R. U.; Shingate, B. B.; Shingare, M. S. *J. Heterocyclic Chem.* **2010**, *47*, 441-445.

[3] Izawa, T.; Miyazaki, E.; Takimiya, K. *Chem. Mater.* **2009**, *21,* 903-912.

[4] De Ridder, R.; Martin, R. H. *Bull. Soc. Chim. Belg.* **1960**, *69*, 534-548.

[5] Bruker, 2004, APEX2 and SAINT. Bruker AXS Inc., Madison, Wisconsin, USA.

[6] Sheldrick, G. M. *Acta Cryst., Sect. A: Found. Crystallogr.* **2008**, *64*, 112.

[7] Sheldrick, G. M. *Acta Cryst.* **2015**, *C71*, 3-8.

[8] Bruker, 2008, SADABS. Bruker AXS Inc., Madison, Wisconsin, USA.).

[9] Sheldrick, G. M. SHELXL-2014. Program for the Refinement of Crystal Structures from Diffraction Data, University of Göttingen, Germany (2014).

[10] APEX3 V2019, Bruker Nano, Inc., **2019**.

[11] SAINT V8.40A, Bruker Nano, Inc., **2019**.

[12] SADABS V2016/2, Bruker Nano, Inc., **2019**.

[13] *International Tables for Crystallography*, Ed. A. J. C. Wilson, Kluwer: Dordrecht, **1992**, Vol. C.

[14] Macrae, C. F.; Sovago, I.; Cottrell, S. J.; Galek, P. T. A.; McCabe, P.; Pidcock, E.; Platings, M.; Shields, G. P.; Stevens, J. S.; Towler, M.; Wood, P. A. Mercury 4.0: from visualization to analysis, design and prediction. *J. Appl. Cryst.*, **2020**, *53*, 226-235.
